# Identification of a venetoclax-resistance prognostic signature base on 6-senescence genes and its clinical significance for acute myeloid leukemia

**DOI:** 10.3389/fonc.2023.1302356

**Published:** 2023-11-30

**Authors:** Peng Ke, Jundan Xie, Ting Xu, Meiyu Chen, Yusha Guo, Ying Wang, Huiying Qiu, Depei Wu, Zhao Zeng, Suning Chen, Xiebing Bao

**Affiliations:** ^1^ National Clinical Research Center for Hematologic Diseases, Jiangsu Institute of Hematology, The First Affiliated Hospital of Soochow University, Suzhou, China; ^2^ Institute of Blood and Marrow Transplantation, Collaborative Innovation Center of Hematology, Soochow University, Suzhou, China

**Keywords:** venetoclax resistance, senescence, prognosis, acute myeloid leukemia, immunotherapy

## Abstract

**Background:**

Satisfactory responses can be obtained for acute myeloid leukemia (AML) treated by Venetoclax (VEN)-based therapy. However, there are still quite a few AML patients (AMLs) resistant to VEN, and it is critical to understand whether VEN-resistance is regulated by senescence.

**Methods:**

Here, we established and validated a signature for predicting AML prognosis based on VEN resistance-related senescence genes (VRSGs). In this study, 51 senescence genes were identified with VEN-resistance in AML. Using LASSO algorithms and multiple AML cohorts, a VEN-resistance senescence prognostic model (VRSP-M) was developed and validated based on 6-senescence genes.

**Results:**

According to the median score of the signature, AMLs were classified into two subtypes. A worse prognosis and more adverse features occurred in the high-risk subtype, including older patients, non-*de novo* AML, poor cytogenetics, adverse risk of European LeukemiaNet (ELN) 2017 recommendation, and *TP53* mutation. Patients in the high-risk subtype were mainly involved in monocyte differentiation, senescence, NADPH oxidases, and PD1 signaling pathway. The model’s risk score was significantly associated with VEN-resistance, immune features, and immunotherapy response in AML. *In vitro*, the IC50 values of ABT-199 (VEN) rose progressively with increasing expression of *G6PD* and *BAG3* in AML cell lines.

**Conclusions:**

The 6-senescence genes prognostic model has significant meaning for the prediction of VEN-resistance, guiding personalized molecularly targeted therapies, and improving AML prognosis.

## Introduction

Acute myeloid leukemia (AML) is one of the most common hematological malignant cancers, which is far more common in elderly patients (1, 2). Traditional chemotherapy era, elderly AML patients (AMLs) have a much poorer prognosis, with a 5-year survival rate of only 5% after the diagnosis (3). With the recent advent of molecularly targeted therapies, such as B-cell lymphoma 2 (*BCL-2*) inhibitor, survival of older AMLs has been improved (4, 5).

The *BCL-2* protein is a key regulator of the mitochondrial apoptotic pathway and plays an important role in the survival and persistence of AML blasts (6, 7). Targeting *BCL-2*, Venetoclax (VEN) showed an efficient strategy to promote caspase-dependent cell death in AML (4, 8). In accordance with these studies, VEN has been approved for the treatment of newly-diagnosed elderly AMLs. VEN-based therapy can induce approximately 70% therapeutic responses in older AMLs. However, a significant minority of AMLs lack therapeutic response to initial induction or re-induction of VEN Monotherapy (9). The short duration of response and development of resistance have become major concerns. Previous studies have found that key contributing factors to VEN resistance include dependencies on alternative anti-apoptotic BCL-2 family proteins, selection of the activating kinase mutations, *TP53* mutation, and BAX variants (10–14). More research is needed to explore the mechanisms of VEN resistance in AML and try to find strategies to overcome the resistance.

Senescence is the natural consequence of telomere shortening at the chromosome ends upon extensive replication, but it can also be induced by DNA damage and imbalances in cellular signaling networks (15, 16). Cellular senescence response may suppress cancer progression *in vivo* (17–19), but could also variously stimulate tumor progression in some conditions, as well as associated with various age-related diseases (20–22). The elimination of senescent cells can delay multiple age-related symptoms, and reduce incidences of spontaneous tumorigenesis and cancer-related mortality (23). Therefore, tumor cells can undergo senescence as an evolutionary process, including both tumor-intrinsic characteristics and extrinsic immune pressure (24, 25). However, a comprehensive understanding of the influences of senescence on VEN-resistance in AML is still lacking. In this study, we developed a VEN-resistance senescence prognostic model (VRSP-M) across multiple AML cohorts. The prognostic signature has significant meaning for the prediction of VEN-resistance, guiding personalized molecularly targeted therapies, and improving AML prognosis.

## Materials and methods

### Data source

The profiles of TCGA AML (n=151) were downloaded from the website of UCSC Xena (https://gdc.xenahubs.net) and exploited to build a prognostic signature for AML based on VRSGs (VEN resistance-related senescence genes). Human-related senescence genes (HRSGs, n=279, **Supplementary Table S1**) were acquired from the HAGR website (https://genomics.senescence.info/cells/). Ex vivo data from Beat AML cohort (26) was used to identify VRSGs and a total of 343 AMLs were enrolled to validate the relationship between prognostic signature and clinical manifestation. To test the applicability, we further verified the effects of a predictive model in non-APL (acute promyelocytic leukemia) AML (GSE106291, n=250) and normal karyotype AML (GSE71014, n=104). The inclusion criteria of Beat AML contained: expression profiles at time of the initial diagnosis, complete data of survival and ELN stratification, but excluding duplicated cases. For other three datasets, all patients with survival and expressed information were included in this study. All the data used in this study were obtained from the public program, and all processes complied with the publication guidelines. Therefore, ethical approval of local ethics committees is not required.

### Identification of VRSGs

From the drug response of beat AML in Ex vivo, samples with the lowest 20% of area-under-the-curve values (AUCs) were deemed to be sensitive to VEN, while those with the highest 20% AUCs were considered as VEN-resistance. A total of 3023 differential expression genes (DEGs) were identified between VEN-resistant and -sensitive samples through the DEseq2 method (|log2FC| ≥1.0 and adjusted P value < 0.05, **Supplementary Table S2**). The intersections of DEGs with HRSGs were identified as VRSGs (n=51).

### Prognostic model generated from VRSGs

According to the median expressed levels of VRSGs and univariate Cox analysis, 18 of 51 VRSGs were proved to be associated with AML prognosis in the modeling set (**Supplementary Table S3**, P<0.05). Of 18 VRSGs, the Least Absolute Shrinkage and Selection Operator (LASSO) algorithm was performed to screen the optimal senescence genes to develop VRSP-M in TCGA-AML through “glmnet” R packet. A 10-fold cross-validation method was employed to hold stability, and the minimum criteria was chosen as the optimal penalty value (λ) (set.seed, 2021). The risk score was calculated using the expression of model’s genes as follows:


$$Risk\;score=\sum_{i=1}^{n}Coef(i)\times x(i)$$

To evaluate and validate VRSP-M in the modeling and validation datasets, AMLs could be divided into high- and low-risk subtypes according to the median score. Survival analyses were applied to distinguish the difference between these two groups by “survival” package. Curves of receiver operating characteristic (ROC) and AUCs were used to assess the accuracy of the prognostic model using the “timeROC” package. Then Beat AML and GSE106291 were combined after removing batch effects by the “SVA” package. A subset containing 80% samples of the combined dataset was re-sampled 100 times and used to examine the robustness of the VRSP-M for predicting OS of AML patients.

### Functional analyses

A previous study demonstrated that a monocytic clone of AML could confer VEN-resistance (27), so its markers were obtained to assess the enrichment of monocyte differentiation. Moreover, the SenMayo set (28) was also used to estimate the degree of enrichment in the senescence pathway. Therefore, four gene sets were acquired to inquire into the biological function of VRSP-M, including c2.cp.kegg.v7.5.1.symbols.gmt, c2.cp.reactome.v7.5.1.symbols.gmt, monocyte differentiation (**Supplementary Table S4**), and the SenMayo gene set (**Supplementary Table S5**). A value of false discovery rate (FDR) < 0.05, adjusted P < 0.05, and |NES| (normalized enrichment score) >1.5 were considered as significant enrichment in gene set enrichment analysis (GSEA).

### Components analysis of immune cells

Using the “GSVA” and “GSEABase” R packages, the different components of immune cells between the high- and low-risk subtypes were reckoned and compared using single-sample gene set enrichment analysis (ssGSEA). The gene set was collected from the previous study (29), containing 28 types of immune cells. Then xCell (30) and ESTIMATE (31) algorithms were further utilized to impute the weights of M2 macrophages, immune and stromal score, respectively.

### Predicted response of immunotherapy

The correlation with Spearman method was performed to check the link between the risk score of VRSP-M and eight immune checkpoints (*SIGLEC15*, *TIGIT*, *CD274*, *HAVCR2*, *PDCD1*, *CTLA4*, *LAG3*, and *PDCD1LG2*). We also predicted whether high-risk subtype could benefit from blockade therapy of immune checkpoints using the algorithm of Tumor Immune Dysfunction and Exclusion (TIDE) (32).

### Screening marker genes of VEN-resistance

Using one dataset of CRISPR-Cas9 screens (GSE216087) (33), we checked whether model’s VRSGs dysregulated in AML cells after single-VEN treatment. When sgRNAs depleted significantly on OCI-AML2 cells after VEN treatment, it meant that knockdown of these genes increased sensitivity to VEN, and high expression of these genes could decrease sensitivity to VEN and contribute to resistance.

To identify which model’s VRSGs could be an effective biomarker of VEN-resistance, we first applied ABT-199 (venetoclax) to search molecular biomarkers of treatment response to VEN using a software of computational analysis of resistance (CARE) (34), the score of which indicates the correlation between gene alteration and drug efficacy. A higher positive score suggests better drug response, while a negative score demonstrates drug resistance. Furthermore, we also got data on CRISPR loss-of-function from previous VEN-resistance research (13). The negatively selected gene indicated that higher expression confers resistance to VEN. Then we verified *in vitro*.

### Cell lines and RT-qPCR

AML cell lines, including HL-60, MOLM13, MV4-11, THP-1 OCI-AML3, and K562, were purchased from the American Type Culture Collection (Manassas, United States). All of them were cultured in RPMI 1640 with 10% FBS (Gibco, United States) as well as antibiotics (1% penicillin-streptomycin). All cells were kept at 37°C in an incubator with 5% CO2.

The total RNA was extracted using RNA Isolator Total RNA Extraction Reagent (R401-01, Vazyme, China), and reverse transcribed to cDNAs using the PrimeScript™ RT Master Kit (RR036A, Takara, Japan). Quantitative real-time PCR (qRT-PCR) was performed using 2x SYBR Green qPCR Master Mix (B21202, Bimake, United States) with 7500 real-time PCR system (Applied Biosystems, United States). The sequences of gene-specific primers are summarized in **Supplementary Table S6**. Gene expression levels were quantified with the 2-ΔCt method and GAPDH was used as endogenous control.

### Western blot

Protein preparation and western blot assay were performed as described previously (35). BAG3 Ab (sc-136467, United States) was purchased from Santa Cruz Biotechnology, and GAPDH Ab (ab8245, United States) was obtained from Abcam. G6PD Ab (AF6945, China) and secondary Abs, such as HRP goat anti-mouse IgG (A0216, China) and goat anti-rabbit IgG (A0208, China), were bought from Beyotime Biotechnology.

### Cell viability assay

The cytotoxic effects of ABT-199 on AML cell lines were determined by a Cell Counting Kit-8 (CCK-8; B34304, Bimake, United States) assay. ABT-199 was diluted in 100 µl of growth medium to designated doses, and leukemia cells were added to the 96-well plate (1×10^4^ cells per well in 100 µl). Cultured leukemia cells were incubated in the presence of the drug for 48 hours at 37°C in a humidified 5% CO2-95% air incubator. Then, 10 μL of the CCK-8 reagent was added into each well, and optical densities at the wavelength of 450 nm were measured using the Synergy HTX Multimode Reader (BioTek, United States). The percentage of surviving cells was calculated according to the absorbance ratio of the test well and control well. IC50 values were calculated and visualized using GraphPad Prism software v9.0 (GraphPad, La Jolla, CA) based on the percent of live cells.

### Statistical analysis

Categorical variables were compared by Chi-square or Fisher’s exact test, and continuous variables were checked using the non-parametric test. Survival differences were quantified through the log-rank method or Cox regression analysis, and visualized using Kaplan-Meier curves. All analyses were conducted using the statistical software R (version 4.1) (http://www.R-project.org). The P-value of two-sided was set at the 0.05 significance level.

## Results

### Construction of VEN-resistance senescence prognostic model

**Figure 1:**
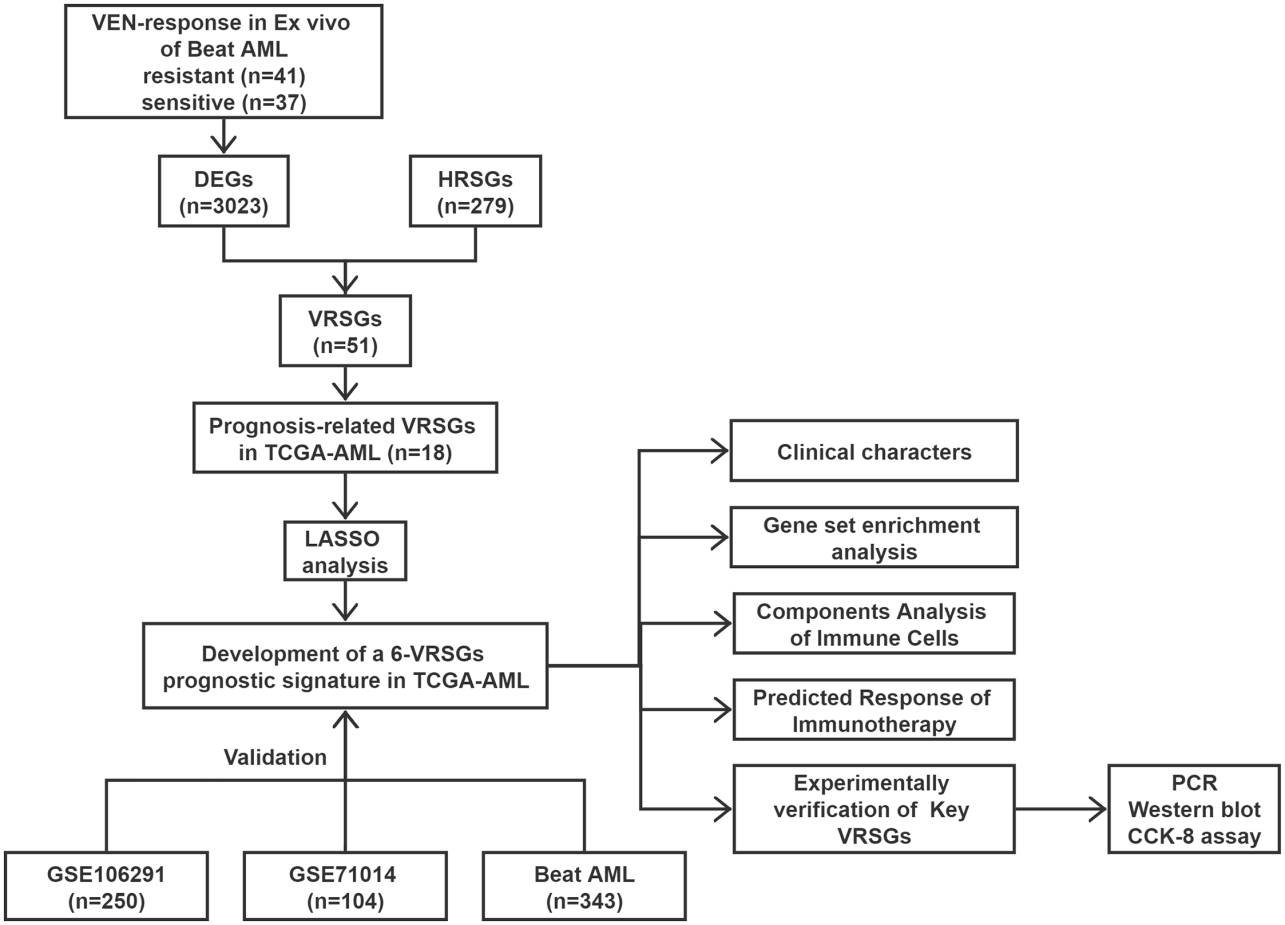
Work flow of the current study.

**Figure 2:**
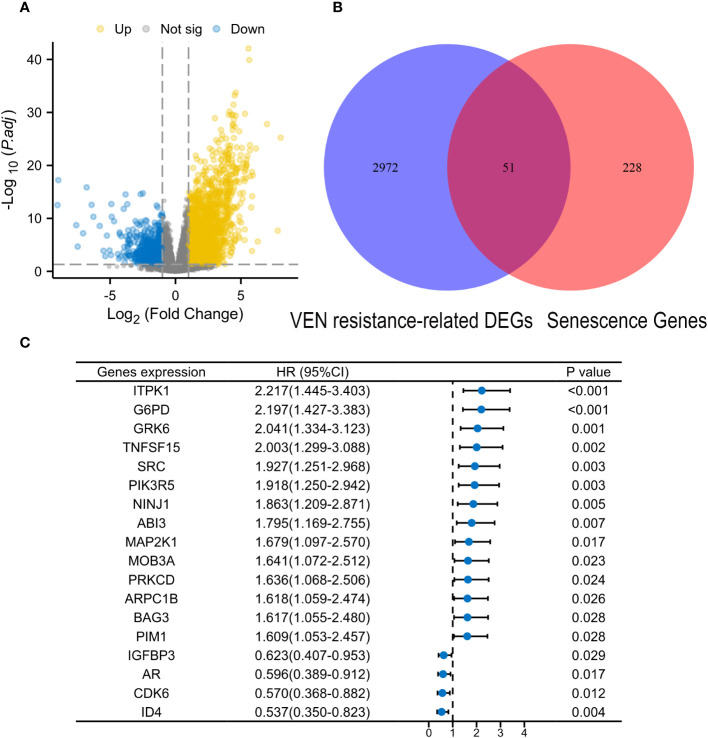
Identification of Venetoclax (VEN) resistance-related senescence genes (VRSGs). **(A)** Volcano map of differential expression genes (DEGs) between VEN-resistant and -sensitive samples through DEseq2 method. **(B)** Intersections of VEN-resistant related DEGs and Human related senescence genes (HRSGs). **(C)** Eighteen VRSGs were confirmed with AML prognosis in the modeling set using univariate Cox regression analysis.

**Figure 3:**
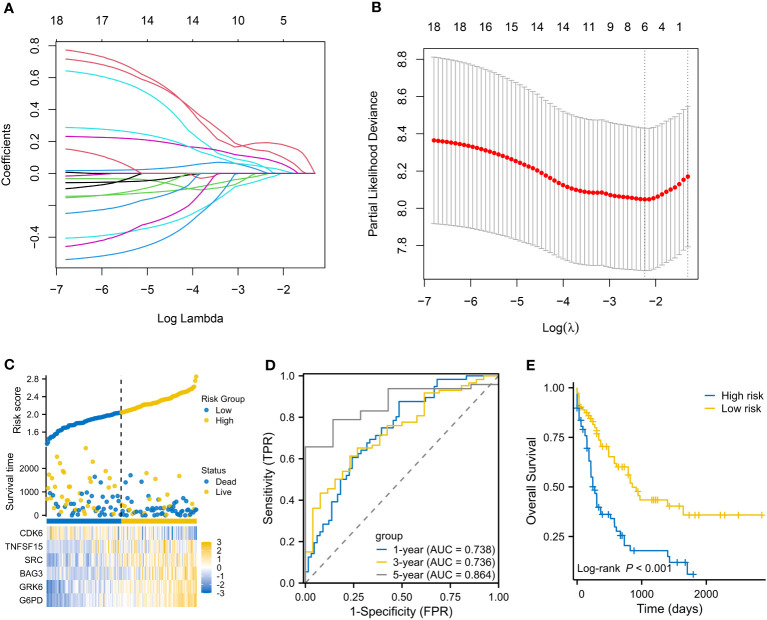
Establishment of a VEN-resistance senescence prognostic model (VRSP-M). **(A, B)** Selecting key genes of the eighteen VRSGs for constructing VRSP-M using LASSO (least absolute shrinkage and selection operator) analysis. **(C)** The distribution of risk score, survival status and heatmap of 6-VRSGs in VRSP-M. **(D)** Curves of ROC (receiver operator characteristic) based on the VRSP-M in the modeling set. AUC, the area under the curve. **(E)** Curve of overall survival (OS) analysis with Kaplan-Meier method in the modeling set.


The flow chart is presented in **Figure 1**. A total of 3023 DEGs were found differently between VEN-sensitivity and -resistance (**Figure 2A**). Fifty-one genes were identified as VRSGs (**Figure 2B**), and 18 of which were verified with AML prognosis (**Figure 2C**). Through the LASSO method, 6 of 18 prognosis-related VRSGs were selected to construct VRSP-M to predict overall survival (OS) in TCGA AML (**Figures 3A, B**). The risk score of each patient was determined based on the following formula (EXP indicated the expression of each gene):

Risk Score=0.1893656×G6PD EXP+0.078429364 ×BAG3 EXP+0.032736642×SRC EXP+0.005747792×TNFSF15 EXP+0.127731446×GRK6 EXP−0.02047377×CDK6 EXP

The distribution of risk score, survival status, and expression of 6-VRSGs are shown in **Figure 3C**. Associated with the elevated risk scores, mortality risk increased, while the survival time decreased. AUCs at 1, 3, and 5 years were 0.738, 0.736, and 0.864 (**Figure 3D**), which indicates that VRSP-M had better accuracy than random choice. Moreover, AMLs with high-risk scores presented a worse OS (P < 0.001, **Figure 3E**).

### VRSP-M correlates with adverse features and monocyte differentiation in AMLs

**Figure 4:**
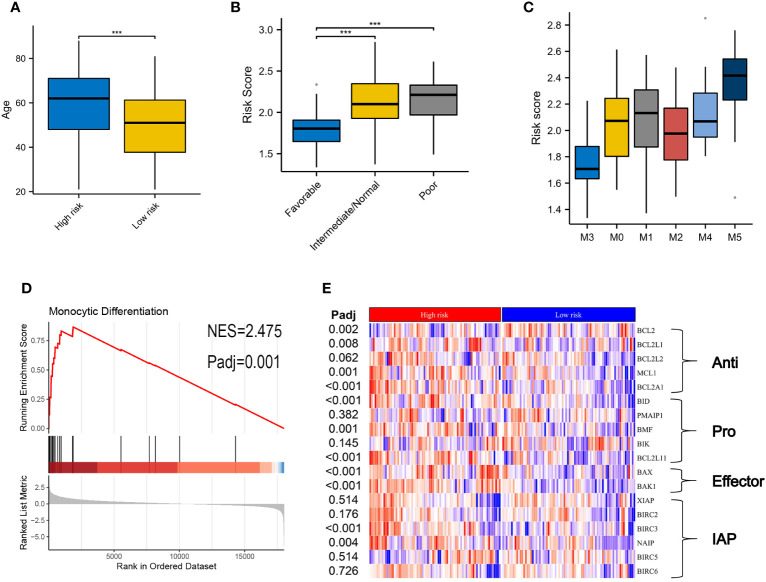
Correlation of clinical features and VRSP-M in the modeling set. **(A)** Difference of age distribution between high- and low-risk subtypes based on VRSP-M. **(B, C)** Distribution of risk score between different risk of cytogenetics and French-American-British (FAB) subtype, including M0, M1, M2, M3, M4 and M5. *** indicate P<0.001. **(D)** Curve of gene set enrichment analysis (GSEA) between high- and low-risk subtypes in monocyte differentiation. **(E)** Coexpression of key genes in apoptosis pathways, the difference was compared by non-parametric test. IAP, inhibitor of apoptosis proteins.

**Table 1** summarizes the clinical characteristics in high- and low-risk subtypes in TCGA-AML. AMLs with high risk were older than those in the low-risk subtype, with median ages of 62 and 51 years respectively (**Figure 4A**, P < 0.001). More AMLs with intermediate or poor cytogenetic risk were located in the high-risk subtype (**Figure 4B**, P<0.001). In the distribution of the French-American-British (FAB) subtype, M5 possessed the highest risk score, while M3 had the lowest score (**Figure 4C**). Furthermore, GSEA analysis also confirmed the enrichment of monocyte differentiation for the high-risk subtype (**Figure 4D**, NES = 2.475).

**Table 1:** Clinical characteristics of AML patients in TCGA cohort.

	Level	Low risk (n=76)	High risk (n=75)	P value
Age (median [IQR]), years		51 [38, 61]	62 [48, 71]	**<0.001**
Gender (%)	Female	36 (47.4)	32 (42.7)	0.677
	Male	40 (52.6)	43 (57.3)	
WBC (median [IQR]) (×10^9^/L)		19.0 [4.0, 42.5]	16.0 [5.0, 70.0]	0.239
HB (median [IQR]) (g/L)		9.0 [9.0, 11.0]	9.0 [9.0, 10.0]	0.276
PLT (median [IQR]) (×10^9^/L)		45.0 [24.5, 83.0]	50.0 [32.0, 87.0]	0.565
BM blast (median [IQR]) %		44.0 [10.0, 68.5]	29.0 [6.0, 59.0]	0.291
FAB (%)	M0	7 (9.2)	8 (10.7)	**0.001**
	M1	15 (19.7)	20 (26.7)	
	M2	24 (31.6)	14 (18.7)	
	M3	14 (18.4)	1 (1.3)	
	M4	14 (18.4)	15 (20.0)	
	M5	2 (2.6)	13 (17.3)	
	M6	0 (0.0)	2 (2.7)	
	M7	0 (0.0)	1 (1.3)	
	Not Classified	0 (0.0)	1 (1.3)	
Cytogenetics risk (%)	Favorable	26 (34.2)	5 (6.7)	**<0.001**
	Intermediate	36 (47.4)	46 (61.3)	
	Poor	14 (18.4)	22 (29.3)	
	NA	0 (0.0)	2 (2.7)	
*FLT3* mutation (%)	Neg	51 (68.0)	51 (70.8)	0.846
	Pos	24 (32.0)	21 (29.2)	
*RAS* mutation (%)	Neg	71 (93.4)	71 (95.9)	0.745
	Pos	5 (6.6)	3 (4.1)	
*NPM1* mutation (%)	Neg	62 (81.6)	55 (74.3)	0.381
	Pos	14 (18.4)	19 (25.7)	
*IDH* mutation (%)	Neg	64 (85.3)	57 (79.2)	0.445
	Pos	11 (14.7)	15 (20.8)	

IQR, inter quartile range; WBC, white blood cells; HB, hemoglobin; PLT, platelet; BM, blast; NA, not available; Pos, positive; Neg, negative.

The meaning of the bold value is P<0.05.

The different expressions of key genes in apoptosis pathways may help to explain why the high-risk subtype was more likely to be resistant to VEN (**Figure 4E** **).** The *BCL2* expression in the high-risk subtype was significantly lower than that in the low-risk subtype, but opposite results occurred in the expression of *BCL2L1*, *BCL2A1*, and *MCL1*. In addition, although the high-risk subtype possessed a high expression of pro-apoptotic genes (*BID*, *BMF*, *BCL2L11*) and apoptosis receptor (*BAX*, *BAK1*), the inhibitor of apoptotic genes (*BIRC3, NAIP*) was also higher in high-risk subtype than those in the low-risk group (adjusted P < 0.05).

### Validating the clinical significance of VRSP-M in AML patients

Three AML cohorts were used to verify the performance of 6-genes VRSP-M. In the Beat AML cohort, the clinical features are depicted in **Table 2**. A higher proportion of M5 subtype, non-*de novo* AML, and adverse risk of ELN recommendation was presented in AMLs with high-risk scores (P < 0.05). When taking genetic mutations into account, patients with no mutation data were excluded from the analysis. AMLs with high-risk scores were frequently accompanied by mutations of *DNMT3A* (P = 0.005), *NRAS* (P = 0.007), *KARS* (P = 0.066), and *TP53* (P = 0.040), while the proportion of *FLT3-ITD* mutation in low-risk subtype was significantly higher than that in high-risk subtype (P = 0.002).

**Table 2:** Clinical characteristics of AML patients in Beat cohort.

	Level	Low risk (n=171)	High risk (n=172)	P value
Age (median [IQR]), years		58 [37, 69]	63 [52, 72]	**0.001**
Gender (%)	Female	79 (46.2)	74 (43.0)	0.629
	Male	92 (53.8)	98 (57.0)	
WBC (median [IQR]) (×10^9^/L)		18.7 [4.9, 52.5]	19.8 [6.5, 48.4]	0.675
HB (median [IQR]) (g/L)		8.5 [7.2, 9.9]	8.5 [7.5, 9.5]	0.724
PLT (median [IQR]) (×10^9^/L)		34.0 [23.0, 63.0]	39.0 [22.0, 90.8]	0.140
PB blast (median [IQR]) %		57.5 [25.0, 83.5]	29.5 [8.0, 61.3]	**<0.001**
BM blast (median [IQR]) %		76.0 [56.0, 90.0]	51.0 [24.0, 76.8]	**<0.001**
FAB (%)	M0	4 (8.7)	2 (4.9)	**0.004**
	M1	5 (10.9)	2 (4.9)	
	M2	6 (13.0)	2 (4.9)	
	M3	9 (19.6)	0 (0.0)	
	M4	13 (28.3)	11 (26.8)	
	M5	8 (17.4)	21 (51.2)	
	M7	0 (0.0)	2 (4.9)	
	NOS	1 (2.2)	1 (2.4)	
De novo AML (%)	No	63 (36.8)	93 (54.1)	**0.002**
	Yes	108 (63.2)	79 (45.9)	
ELN 2017 risk (%)	Favorable	68 (39.8)	32 (18.6)	**<0.001**
	Intermediate	55 (32.2)	61 (35.5)	
	Adverse	48 (28.1)	79 (45.9)	
*FLT3-ITD* mutation (%)	Neg	128 (74.9)	152 (88.4)	**0.002**
	Pos	43 (25.1)	20 (11.6)	
*NPM1* mutation (%)	Neg	124 (72.9)	140 (81.4)	0.083
	Pos	46 (27.1)	32 (18.6)	
*ASXL1* mutation (%)	Neg	3 (23.1)	1 (6.2)	0.444
	Pos	10 (76.9)	15 (93.8)	
*CEBPA* mutation (%)	Neg	77 (81.1)	64 (92.8)	0.057
	Pos	18 (18.9)	5 (7.2)	
*DNMT3A* mutation (%)	Neg	55 (80.9)	37 (56.9)	**0.005**
	Pos	13 (19.1)	28 (43.1)	
*FLT3* mutation (%)	Neg	80 (86.0)	71 (83.5)	0.800
	Pos	13 (14.0)	14 (16.5)	
*IDH1* mutation (%)	Neg	75 (87.2)	73 (90.1)	0.727
	Pos	11 (12.8)	8 (9.9)	
*IDH2* mutation (%)	Neg	69 (83.1)	65 (81.2)	0.913
	Pos	14 (16.9)	15 (18.8)	
*KIT* mutation (%)	Neg	73 (91.2)	61 (98.4)	0.144
	Pos	7 (8.8)	1 (1.6)	
*KRAS* mutation (%)	Neg	65 (94.2)	46 (82.1)	**0.066**
	Pos	4 (5.8)	10 (17.9)	
*NRAS* mutation (%)	Neg	61 (81.3)	39 (59.1)	**0.007**
	Pos	14 (18.7)	27 (40.9)	
*TP53* mutation (%)	Neg	61 (89.7)	43 (74.1)	**0.040**
	Pos	7 (10.3)	15 (25.9)	
*RUNX1* mutation (%)	Neg	23 (69.7)	15 (46.9)	0.106
	Pos	10 (30.3)	17 (53.1)	

PB, Peripheral Blood; ELN, European LeukemiaNet. When taking genetic mutations into account, cases without genetic mutations data were excluded from analysis.

The meaning of the bold value is P<0.05.

**Figure 5:**
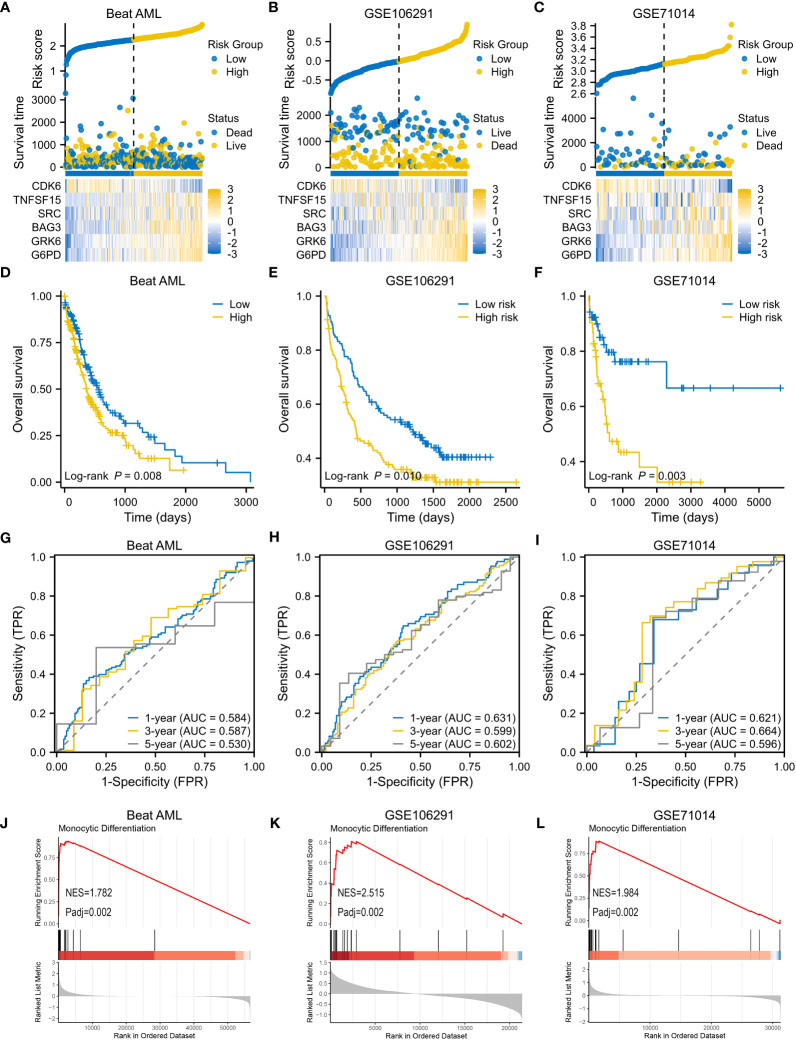
Validation of VRSP-M in three independent AML cohorts. **(A–C)** The distribution of risk score, survival status and heatmap of 6-VRSGs in three validation sets. **(D–F)** Curves of survival analysis in Beat AML, GSE106291, and GSE71014. **(G–I)** Curves of ROC analysis in Beat AML, GSE106291, and GSE71014. **(J–L)** GSEA analysis of monocyte differentiation between high- and low-risk subtypes.

Consistent with the results in the modeling set, as risk scores increased, death risk also went up in three validation cohorts (**Figures 5A–C**). A worse OS was confirmed for the high-risk subtype (**Figures 5D–F**), and the VRSP-M also had the ability to predict AML prognosis (**Figures 5G–I**). Similarly, patients with high-risk scores were mainly involved in monocyte differentiation (**Figures 5J–L**). Leukemia heterogeneity may limit the practicability of prognostic signatures. Thus, we combined Beat AML and GSE106291 after removing batch effects by the SVA package. To check the robustness of this 6-genes prognostic signature, a total of 100 re-sampling tests were conducted randomly in 80% samples of the combined dataset. The results indicated that the P values were less than 0.05 in each Kaplan-Meier and univariate Cox analysis (**Table S7**), indicating a high performance for OS prediction in AML.

### Functional signaling pathways

**Figure 6:**
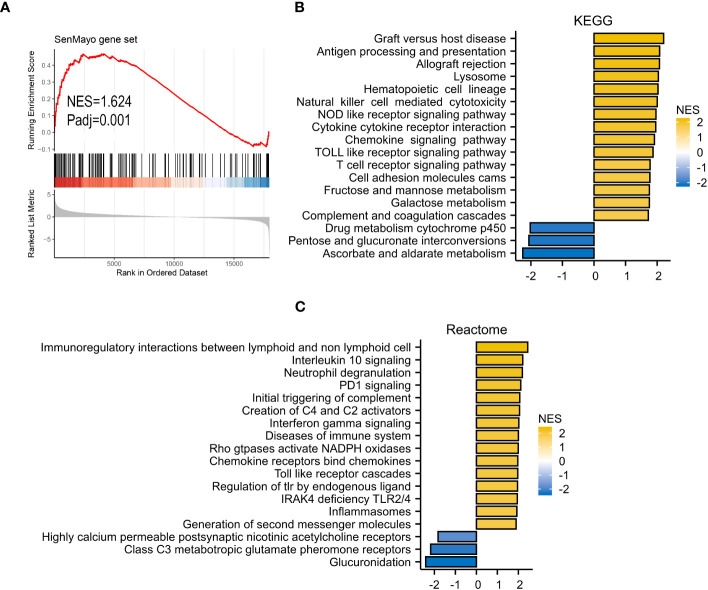
GSEA analysis. **(A)** SenMayo gene set. **(B)**, KEGG (Kyoto Encyclopedia of Genes and Genomes) gene set. **(C)** Reactome gene set.

GSEA analyses were performed to better investigate the potential function of VRSP-M. The high-risk subtype was highly enriched in the senescence pathway (**Figure 6A**). KEGG results revealed that high-risk subtype enriched in Lysosome, Hematopoietic cell lineage, Cell adhesion molecules, and many immune-related pathways, such as cytotoxicity mediated by natural killer cell, interaction of cytokine and cytokine receptor, chemokine and T cell receptor signaling pathway (**Figure 6B**). Moreover, the main items of Reactome analysis were interactions between lymphoid and non-lymphoid cell, NADPH oxidases, interleukin 10 and PD1 signaling (**Figure 6C**).

### VRSP-M correlates with immune features in AMLs

**Figure 7:**
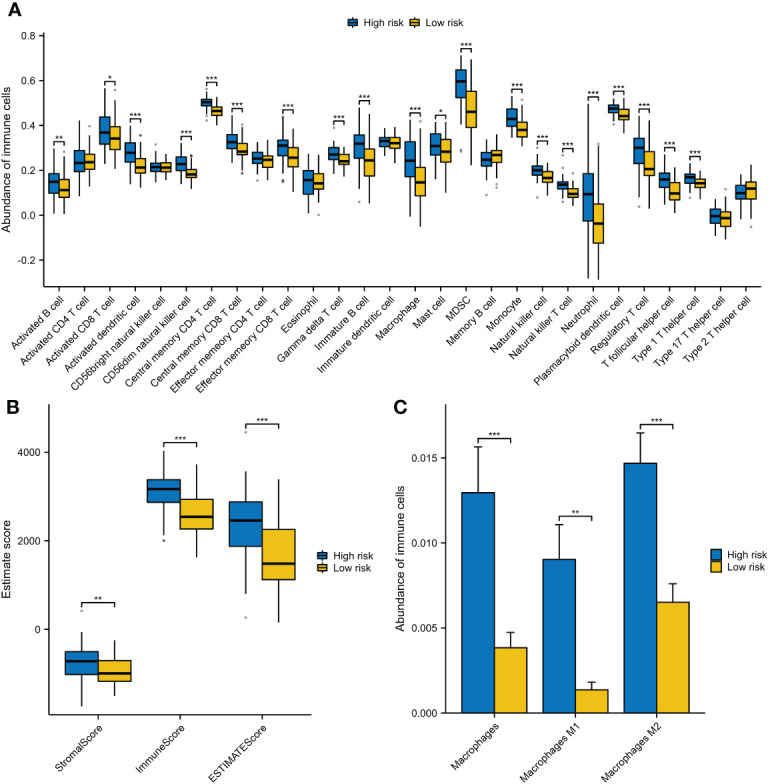
The different components of immune cells between high- and low-risk subtypes of VRSP-M. **(A)** The difference of 28 types of immune cells, evaluating by single-sample gene set enrichment analysis (ssGSEA). **(B)** The difference of immune and stromal score, calculated by ESTIMATE method. **(C)** Subtypes of macrophages between high- and low-risk AMLs, estimated by xCell algorithm. * indicate P<0.05, ** indicate P<0.01, *** indicate P<0.001.

Many enriched items of immune-related pathways prompted us to explore the immune features associated with VRSP-M. We found that patients with high-risk score were often accompanied with a higher immune cell infiltration, including various subtypes of immune activated and immunosuppressive cells (**Figure 7A**). The high-risk AMLs both had a higher immune and stromal score (**Figure 7B**). In addition, as well as Myeloid-derived suppressor cells (MDSCs) (**Figure 7A**), a higher proportion of M2 macrophage cells was also observed in patients with high-risk score (**Figure 7C**).

### VRSP-M associated with immunotherapy response of AMLs

**Figure 8:**
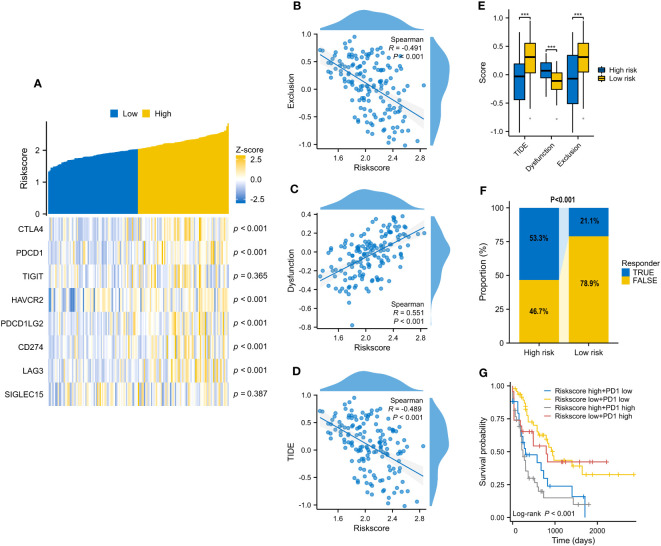
The correlation of VRSP-M’s risk score with 8 immune checkpoints and predicted response of immunotherapy. **(A–D)** The relationship of VRSP-M’s risk score with expression of 8 immune checkpoints, Exclusion, Dysfunction, and TIDE score, tested by Spearman method. **(E, F)** The difference of Exclusion, Dysfunction, TIDE score, and predicted response of immunotherapy between high- and low-risk subtypes. **(G)** Survival analysis of classification based on *PDCD1* expression and VRSP-M’s risk score. *** indicate P<0.001.

On account of the above discoveries, we speculated that PD1 plays a vital role in VRSP-M. The results showed that the risk score of the VRSP-M was positively correlated with *CTLA4* (R=0.402), *PDCD1* (R=0.398), *HAVCR2* (R=0.328), *PDCD1LG2* (R=0.283), *CD274* (R=0.281), *LAG3* (R=0.324), and T cell dysfunction (R=0.551), but negatively related with TIDE score (R= -0.489) and T cell exclusion (R= -0.491) (**Figures 8A–D**, P < 0.001). With a lower TIDE score (**Figure 8E**, P < 0.001), the high-risk subtype had a higher number of responders from immunotherapy (53.3% vs. 21.1%, P < 0.001) (**Figure 8F**). According to the expression of *PDCD1* and VRSP-M’s risk score, AMLs were divided into four groups. Patients both with a high level of risk score and *PDCD1* presented a worse prognosis (**Figure 8G**, P < 0.001). These results indicated that patients with high-risk scores may benefit from the blocking therapy of immune checkpoints.

### Verification of the maker genes of VRSP-M *in vitro*

In this signature, five senescence genes (except *CDK6*) were positively associated with higher VRSP-M scores. We then checked whether these five genes were dysregulated in AML cells using another dataset of CRISPR-Cas9 screens (GSE216087). The results indicated that four sgRNAs (except *GRK6*) were depleted significantly on OCI-AML2 cells after single-VEN treatment (**Figure S1**), suggesting that overexpression of these genes could confer VEN-resistance.

**Figure 9:**
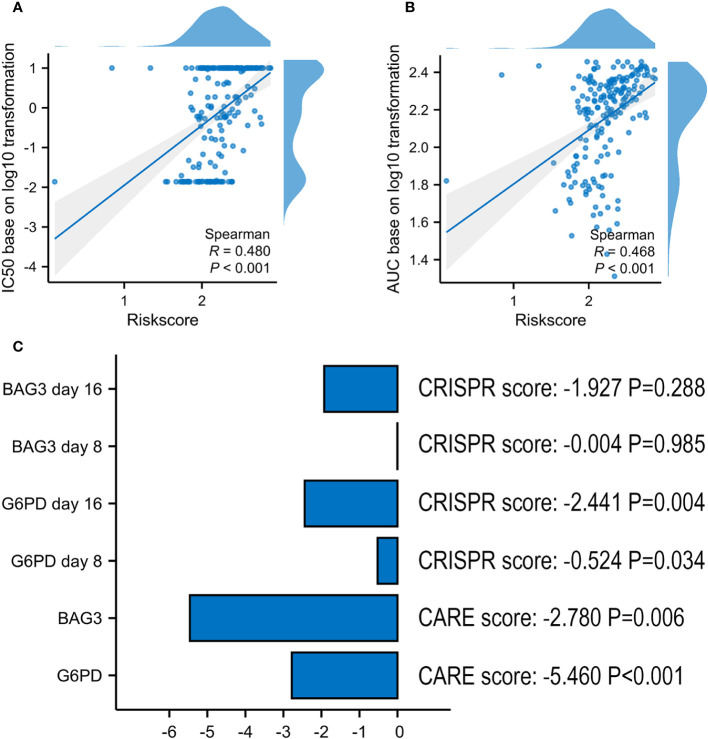
Screening Marker genes from VRSP-M. **(A, B)** The correlation analysis of VRSP-M’s risk score with IC50 values (log10 transformation) and AUC values (log10 transformation) of VEN in Ex vivo data from Beat AML sample, tested by Spearman method. **(C)** The importance of *G6PD* and *BAG3* in VEN-resistance, and the scores were evaluated using by CARE algorithm and CRISPR data.

In ex vivo data from the Beat AML sample, the risk score was proved to be positively associated with VEN-resistance (**Figures 9A, B**). Using CARE software, 2 of 6 model’s genes had a negative score as follows: *G6PD* (-5.460, P < 0.001) and *BAG3* (-2.780, P = 0.006) (**Figure 9C**). Additionally, *G6PD* also presented a negative CRISPR score both at day 8 (-0.524, P = 0.034) and day 16 (-2.441, P = 0.004) post co-culture of VEN, compared with control.

**Figure 10:**
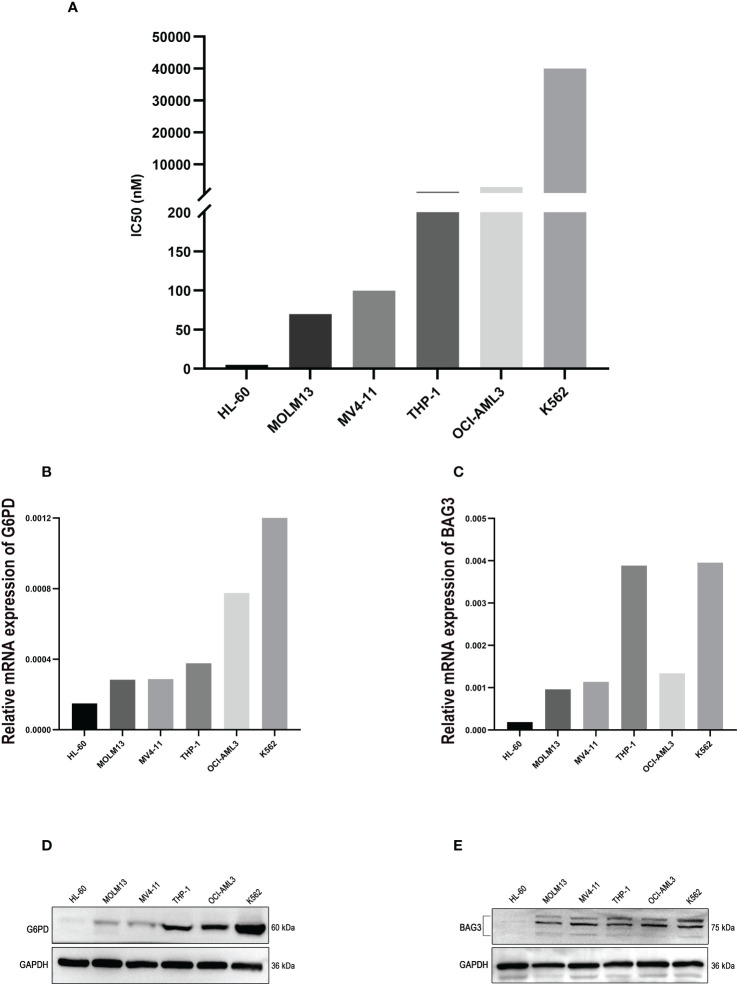
The experimental verification of the Key Markers. **(A)** The IC50 values of ABT-199 (Venetoclax) in different AML cell lines, tested by CCK-8 assay. **(B, C)** The mRNA levels of *G6PD* and *BAG3* expression in different AML cell lines, using PCR method. **(D, E)** Protein levels of *G6PD* and *BAG3* in different AML cell lines, tested by Western blot.

Whereafter, a method of CCK-8 assay was used to examine the viability of AML cell lines treated with ABT-199 for 48 hours and determined the IC50 values. The IC50 values ranged from <10 nmol/L to >1000 nmol/L (**Figure 10A**). The expression levels of *G6PD* and *BAG3* were further tested in these AML cell lines, such as HL-60, MOLM13, MV4-11, and so on. The results indicated that *G6PD* expression was positively correlated with the IC50 of ABT-199 (**Figure 10B**). Except for OCI-AML3, the expression of *BAG3* was also positively correlated with VEN-resistance (**Figure 10C**). The expression of *G6PD* and *BAG3* were also verified in these AML cell lines using Western blot. As shown in **Figures 10D, E**, the IC50 levels of ABT-199 rose progressively with increasing protein levels of *G6PD* and *BAG3*. These findings indicate that *G6PD* and *BAG3* may be effective markers for VEN-resistance in AML.

## Discussion

Senescence is a complex stress response that can be grouped into different categories including genome-based failures and signaling dysfunction. However, the role of cellular senescence in cancer is controversial. In some conditions, the response of cellular senescence suppresses cancer progression (17, 18), conversely, which variously stimulates tumor progression in other ways (20–22). However, it is not quite clear whether senescence could induce VEN-resistance in AML. To better understand it, a VRSP-M was developed and validated using multiple AML cohorts, which can distinguish the prognosis of AMLs.

In this prognostic model, *G6PD*, *BAG3*, *SRC*, *TNFSF15*, and *GRK6* act as risk factors, whereas *CDK6* is a protective factor. Moreover, *G6PD* possesses the highest weight on AML prognosis, and was also proven to be an effective molecular marker of VEN-resistance. Previous studies have indicated that *G6PD* overexpression was associated with a poor prognosis in certain types of cancer, including AML, hepatocellular carcinoma, invasive breast carcinoma, and mesothelioma (36). *G6PD* could promote cancer progression through its effects on some metabolic pathways (37, 38). Decreasing proliferation of leukemia and other cancer cells, knockdown of *G6PD* significantly increased apoptosis of tumor cells which are also more susceptible to oxidative stress (39, 40). Acting as an effector of ATM (Ataxia telangiectasia mutated), *G6PD* often participates in the development of various cancers through metabolic programming and DNA repair pathways (41). As an essential enzyme in the pentose phosphate pathway (PPP), *G6PD* could produce more materials by this pathway to meet the high anabolic needs of tumor cells, which may make the cancers more resistant to chemotherapy. Playing an important role in AML resistance to the FLT3 inhibitor, the inactivation of *G6PD* increases the sensitivity of AML to FLT3 inhibitors (42). Under various stresses, multiple tumors turn metabolism to the PPP to get enough reductants to fight against reactive oxygen species (ROS) by activating *G6PD* rapidly. Otherwise, *G6PD* can also affect cancers by regulating ROS. Modulation of *G6PD* was proven to affect bladder cancer via ROS accumulation and the AKT pathway *in vitro* (43), and knockdown of *G6PD* reduces ROS accumulation and enhances apoptosis of bladder cancer cells. In addition, *G6PD* also facilitates clear cell renal cell carcinoma invasion through the ROS−MAPK axis pathway (44).

Up-regulated ROS level induced by *G6PD* activation not only leads to out of control of cell growth and apoptosis of cancer, by also affects the immune microenvironment. In our study, high-risk AMLs also activate the signal of NADPH oxidases, and excessive activation of NOX (non-phagocytic cell oxidase) in cancer cells results in a large amount of ROS production. Accumulation of ROS leads to apoptosis in normal cells and mediates cellular senescence (45), which may be one of the reasons for the activation of NADPH oxidases in the high-risk subtype. Otherwise, excessive ROS also maintains the proliferation of tumor cells due to the protection of anti-oxidative stress with NADPH (39, 46–48). In fact, overexpression of *G6PD* could protect leukemic cells against oxidative stress by increasing NADPH production. On the other hand, not only leading to DNA damage and genomic instability, excessive of ROS could regulate signal transduction in the tumor environment, all of these are beneficial to the growth and progress of tumors (49, 50). Previous study has demonstrated that elevated ROS level induced by VEN can enhance the anti-leukemia effect of T cells (51). However, many studies also confirmed that ROS could induce the polarization of macrophages to M2 subtypes (52–56). Acting as a double-edged sword, ROS not only enhances T cells’ anti-leukemia effect, but stimulates other immune cells, such as M2 macrophages and MDSCs. Hence, cellular senescence often triggers an immune response in the tumor microenvironment, facilitating tumor formation and progression (57, 58), which might lead to a higher immune and stromal score in our high-risk AMLs. Moreover, a higher infiltration of MDSCs and M2 Macrophages in the high-risk subtype could prevent immune clearance of leukemia cells, and lead to poorer prognosis (59).

Shaping an unfavorable immune microenvironment, immunosenescence is also an urgent problem to be solved in the treatment of cancers. Mainly involved in PD1 signaling and strongly related to many immune checkpoints, a high-risk score of the VRSP-M was positively correlated with T cell dysfunction but negatively with T cell exclusion. T-cell dysfunction in cancer displays functional unresponsiveness, including senescence, exhaustion, anergy, and self-tolerance that is increasingly recognized as major hurdles for the success of cancer immunotherapy (60–62). So, potential approach to enhance anti-leukemia is to improve T and NK dysfunction, such as PD-1 inhibitor, chimeric antigen receptor T-cell therapy, and NK or γδ T-based adoptive immunotherapies (63–65). While the blocking-up of immune checkpoints has led to breakthroughs in several solid cancer therapies, research in AML remains limited (66). In the real world, AMLs have limited benefits from anti-PD-1 therapy (67, 68), which may be due to many AMLs often accompanied by T cell exclusion. The resistance to immunotherapy in AML, such as PD-1 blockade, remains one of the major challenges impeding its application in the future. So, a higher predictive response of immunotherapy in the high-risk subtype may bring a new perspective towards AML therapy, and VEN combined with blocking therapy of immune checkpoints is worthy of further exploration.

Considering VEN-resistance from the perspective of gene interaction, patients in the high-risk group presented a lower *BCL2* expression, but higher levels of *BCL2L1*, *BCL2A1*, and *MCL1*. This means that the high-risk score often causes VEN-resistance by interacting with anti-apoptosis proteins, which is one of the main causes of VEN-resistance (9, 69, 70). Of other 5 senescence genes, as the co-variants of *BCL-2*, *BAG3* works together with *BAG1* to maintain the viability of myeloid cells, dysregulation of which could lead to physiological abnormalities (71). Notably, previous studies have indicated that *BAG3* is associated with a poor AML prognosis (72), and involved in resistance to chemotherapy (73). Bound to the DR3 receptor, *TNFSF15* often plays pro-inflammatory roles and regulates cytokine release (74). Furthermore, the *TNFSF15/DR3* axis is involved in promoting apoptosis through Caspase pathways, but could also activate inhibitors of apoptosis proteins by regulating *NF-κB* pathways and inhibiting apoptosis (75). It is noteworthy that persistent inflammation could also promote the progression and resistance of tumors (76). Abnormal activation of *SRC* protein, one of the non-receptor tyrosine kinases, is closely related to the tumors’ progression. Overexpression of which could lead to increased Src kinase activity, and play an important role in human cancer, including cell proliferation, differentiation, survival, and mortality (77). Besides, up-regulated *GRK6* level is also associated with the progression and prognosis of colorectal carcinoma (78), and its role in AML is worth further exploration. Interestingly, AMLs sensitive to VEN-therapy are enriched in various gene sets of leukemic stem cells (LSCs), while the major enrichment of VEN-resistant AMLs is monocytic differentiation (27). As an essential regulatory molecule for activating LSCs (79), *CDK6* is up-regulated significantly in VEN-sensitive AMLs. Consistent with these results, *CDK6* levels are negatively associated with the risk score of VRSP-M, and high-risk AMLs are also enriched in the monocytic phenotype. In both mice and humans, aging is often accompanied by alteration of the monocyte function and increased production of classical monocytes expressing MHC II, which may help to explain why our high-risk subtype was mainly involved in monocyte differentiation and resistant to VEN-therapy (80).

In the context of oncogenes and clinical characteristics, patients in the high-risk subtype were frequently accompanied by mutations of *NRAS*, *KARS*, and *TP53*. Of note, AML with *RAS* mutation was associated with VEN-resistance and monocytic phenotype (27, 81). As one of the most common proto-oncogenes in AML, a gain of function in *KRAS/NRAS* could activate the pathway of *RAS/MAPK*, and further lead to overexpression and increased stability of *MCL-1* protein, which also plays a major role in VEN-resistance. Furthermore, *RAS* regulatory genes such as *PTPN11* usually co-mutate with *KRAS/NRAS* mutation, which have been reported refractory to VEN monotherapy in AML (12, 82). In addition, a higher *TP53* mutation may also help explain why the high-risk AML subtype exhibited a lower response to VEN and a poor prognosis (4, 83, 84). In general, our results demonstrate that more adverse prognostic features were presented in the high-risk group, including older patients, non-*de novo* AML, poor cytogenetics or adverse ELN risk, and *TP53* mutation. All these results support why high-risk patients in our results have worse survival.

Our study provides a new perspective and potential therapeutic targets based on senescence aid to explore pathogenesis of VEN-resistance in AML; however, there are still several limitations in the current study. More independent AML cohorts are needed to validate it. Moreover, further investigation is needed to explore the underlying mechanisms. The risk score of the prognostic model is significantly associated with VEN-resistance, immune features, and immunotherapy response in AML. We also verified that *G6PD* and *BAG3* could be effective biomarkers of VEN-resistance *in vitro*. In conclusion, the 6-senescence genes prognostic model has significant meaning for the prediction of VEN-resistance, guiding personalized molecularly targeted therapies, and improving AML prognosis.

## Data availability statement

The original contributions presented in the study are included in the article/**Supplementary Material**. Further inquiries can be directed to the corresponding authors. The GDC TCGA-LAML cohort for this study can besought out on the UCSC Xena website (https://gdc.xenahubs.net), datasets of GSE71014 and GSE106291, GSE71014, and GSE216087 can be downloaded from the GEO website (https://www.ncbi.nlm.nih.gov/geo/), and Beat AML cohort can be obtained from the Beat AML program.

## Ethics statement

Ethical approval was not required for this study in accordance with the local legislation and institutional requirements because only commercially available established cell lines were used.

## Author contributions

PK: Data curation, Writing – original draft. JX: Data curation, Writing – original draft. TX: Data curation, Writing – original draft. MC: Formal Analysis, Software, Writing – original draft. YG: Formal Analysis, Software, Writing – original draft. YW: Formal Analysis, Software, Writing – original draft. HQ: Formal Analysis, Software, Writing – original draft. DW: Formal Analysis, Software, Writing – original draft. ZZ: Project administration, Writing – review & editing. SC: Project administration, Writing – review & editing. XB: Project administration, Writing – review & editing.

## References

[1] Kelly LM Gilliland DG. Genetics of myeloid leukemias. Annu Rev Genomics Hum Genet (2002) 3:179–98. doi: 10.1146/annurev.genom.3.032802.115046 12194988

[2] Almeida AM Ramos F. Acute myeloid leukemia in the older adults. Leuk Res Rep (2016) 6:1–7. doi: 10.1016/j.lrr.2016.06.001 27408788 PMC4927655

[3] Alibhai SM Leach M Minden MD Brandwein J. Outcomes and quality of care in acute myeloid leukemia over 40 years. Cancer (2009) 115(13):2903–11. doi: 10.1002/cncr.24373 19452536

[4] Dinardo CD Pratz K Pullarkat V Jonas BA Arellano M Becker PS. Venetoclax combined with decitabine or azacitidine in treatment-naive, elderly patients with acute myeloid leukemia. Blood (2019) 133(1):7–17. doi: 10.1182/blood-2018-08-868752 30361262 PMC6318429

[5] Dombret H Seymour JF Butrym A Wierzbowska A Selleslag D Jang JH. International phase 3 study of azacitidine vs conventional care regimens in older patients with newly diagnosed AML with >30% blasts. Blood (2015) 126(3):291–9. doi: 10.1182/blood-2015-01-621664 PMC4504945 25987659

[6] Vo TT Ryan J Carrasco R Neuberg D Rossi DJ Stone RM. Relative mitochondrial priming of myeloblasts and normal HSCs determines chemotherapeutic success in AML. Cell (2012) 151(2):344–55. doi: 10.1016/j.cell.2012.08.038 PMC3534747 23063124

[7] Konopleva M Letai A. BCL-2 inhibition in AML: an unexpected bonus? Blood (2018) 132(10):1007–12. doi: 10.1182/blood-2018-03-828269 PMC6235069 30037885

[8] Pan R Hogdal LJ Benito JM Bucci D Han L Borthakur G. Selective BCL-2 inhibition by ABT-199 causes on-target cell death in acute myeloid leukemia. Cancer Discovery (2014) 4:362–75. doi: 10.1158/2159-8290.CD-13-0609 PMC3975047 24346116

[9] Konopleva M Pollyea DA Potluri J Chyla B Hogdal L Busman T. Efficacy and biological correlates of response in a phase II study of venetoclax monotherapy in patients with acute myelogenous leukemia. Cancer Discov (2016) 6:1106–17. doi: 10.1158/2159-8290.CD-16-0313 PMC5436271 27520294

[10] Bisaillon R Moison C Thiollier C Krosl J Bordeleau ME Lehnertz B. Genetic characterization of ABT-199 sensitivity in human AML. Leukemia (2020) 34:63–74. doi: 10.1038/s41375-019-0485-x 31300747

[11] Maiti A Rausch CR Cortes JE Pemmaraju N Daver NG Ravandi F. Outcomes of relapsed or refractory acute myeloid leukemia after frontline hypomethylating agent and venetoclax regimens. Haematologica (2021) 106:894–8. doi: 10.3324/haematol.2020.252569 PMC7927994 32499238

[12] Chyla B Daver N Doyle K McKeegan E Huang X Ruvolo V. Genetic biomarkers of sensitivity and resistance to venetoclax monotherapy in patients with relapsed acute myeloid leukemia. Am J Hematol (2018) 93(8):E202–205. doi: 10.1002/ajh.25146 PMC6120451 29770480

[13] Chen X Glytsou C Zhou H Narang S Reyna DE Lopez A. Targeting mitochondrial structure sensitizes acute myeloid leukemia to venetoclax treatment. Cancer Discovery (2019) 9:890–909. doi: 10.1158/2159-8290.CD-19-0117 31048321 PMC6606342

[14] Nechiporuk T Kurtz SE Nikolova O Liu T Jones CL D’Alessandro A. The TP53 apoptotic network is a primary mediator of resistance to BCL2 inhibition in AML cells. Cancer Discovery (2019) 9:910–25. doi: 10.1158/2159-8290.CD-19-0125 PMC6606338 31048320

[15] Birch J Gil J. Senescence and the SASP: many therapeutic avenues. Genes Dev (2020) 34:1565–76. doi: 10.1101/gad.343129.120 PMC7706700 33262144

[16] Gorgoulis V Adams PD Alimonti A Bennett DC Bischof O Bishop C. Cellular senescence: defining a path forward. Cell (2019) 179:813–27. doi: 10.1016/j.cell.2019.10.005 31675495

[17] He S Sharpless NE. Senescence in health and disease. Cell (2017) 169:1000–11. doi: 10.1016/j.cell.2017.05.015 PMC5643029 28575665

[18] Kowald A Passos JF Kirkwood TBL. On the evolution of cellular senescence. Aging Cell (2020) 19:e13270. doi: 10.1111/acel.13270 33166065 PMC7744960

[19] Lee S Schmitt CA. The dynamic nature of senescence in cancer. Nat Cell Biol (2019) 21:94–101. doi: 10.1038/s41556-018-0249-2 30602768

[20] Wang B Kohli J Demaria M. Senescent cells in cancer therapy: friends or foes? Trends Cancer (2020) 6:838–57. doi: 10.1016/j.trecan.2020.05.004 32482536

[21] Salama R Sadaie M Hoare M Narita M. Cellular senescence and its effector programs. Genes Dev (2014) 28(2):99–114. doi: 10.1101/gad.235184.113 24449267 PMC3909793

[22] Pérez-Mancera PA Young AR Narita M. Inside and out: the activities of senescence in cancer. Nat Rev Cancer (2014) 14(8):547–58. doi: 10.1038/nrc3773 25030953

[23] Baker DJ Childs BG Durik M Wijers ME Sieben CJ Zhong J. Naturally occurring p16(Ink4a)-positive cells shorten healthy lifespan. Nature (2016) 530:184–9. doi: 10.1038/nature16932 PMC4845101 26840489

[24] Lasry A Ben-Neriah Y. Senescence-associated inflammatory responses: aging and cancer perspectives. Trends Immunol (2015) 36(4):217–28. doi: 10.1016/j.it.2015.02.009 25801910

[25] Berben L Floris G Wildiers H Hatse S. Cancer and aging: two tightly interconnected biological processes. Cancers (Basel) (2021) 13(6):1400. doi: 10.3390/cancers13061400 33808654 PMC8003441

[26] Tyner JW Tognon CE Bottomly D Wilmot B Kurtz SE Savage SL. Functional genomic landscape of acute myeloid leukaemia. Nature (2018) 562(7728):526–31. doi: 10.1038/s41586-018-0623-z PMC6280667 30333627

[27] Pei S Pollyea DA Gustafson A Stevens BM Minhajuddin M Fu R. Monocytic subclones confer resistance to venetoclax-based therapy in patients with acute myeloid leukemia. Cancer Discov (2020) 10(4):536–51. doi: 10.1158/2159-8290.CD-19-0710 PMC7124979 31974170

[28] Saul D Kosinsky RL Atkinson EJ Doolittle ML Zhang X LeBrasseur NK. A new gene set identifies senescent cells and predicts senescence-associated pathways across tissues. Nat Commun (2022) 13(1):4827. doi: 10.1038/s41467-022-32552-1 35974106 PMC9381717

[29] Charoentong P Finotello F Angelova M Mayer C Efremova M Rieder D. Pan-cancer immunogenomic analyses reveal genotype-immunophenotype relationships and predictors of response to checkpoint blockade. Cell Rep (2017) 18(1):248–62. doi: 10.1016/j.celrep.2016.12.019 28052254

[30] Aran D Hu Z Butte AJ. xCell: digitally portraying the tissue cellular heterogeneity landscape. Genome Biol (2017) 18(1):220. doi: 10.1186/s13059-017-1349-1 29141660 PMC5688663

[31] Yoshihara K Shahmoradgoli M Martínez E Vegesna R Kim H Torres-Garcia W. Inferring tumour purity and stromal and immune cell admixture from expression data. Nat Commun (2013) 4:2612. doi: 10.1038/ncomms3612 24113773 PMC3826632

[32] Jiang P Gu S Pan D Fu J Sahu A Hu X. Signatures of T cell dysfunction and exclusion predict cancer immunotherapy response. Nat Med (2018) 24(10):1550–8. doi: 10.1038/s41591-018-0136-1 PMC6487502 30127393

[33] Eide CA Kurtz SE Kaempf A Long N Joshi SK Nechiporuk T. Clinical correlates of venetoclax-based combination sensitivities to augment acute myeloid leukemia therapy. Blood Cancer Discov (2023) 4(6):452–67. doi: 10.1158/2643-3230 PMC10618724 37698624

[34] Jiang P Lee W Li X Johnson C Liu JS Brown M. Genome-scale signatures of gene interaction from compound screens predict clinical efficacy of targeted cancer therapies. Cell Syst (2018) 6(3):343–354.e5. doi: 10.1016/j.cels.2018.01.009 29428415 PMC5876130

[35] Li J Shen Z Wang Z Chao H Xu Y Zeng Z. CTCF: A novel fusion partner of ETO2 in a multiple relapsed acute myeloid leukemia patient. J Leukoc Biol (2022) 111(5):981–7. doi: 10.1002/JLB.2A0720-441RR 34622967

[36] Li R Wang W Yang Y Gu C. Exploring the role of glucose-6-phosphate dehydrogenase in cancer (Review). Oncol Rep (2020) 44(6):2325–36. doi: 10.3892/or.2020.7803 33125150

[37] Spencer NY Stanton RC. Glucose 6-phosphate dehydrogenase and the kidney. Curr Opin Nephrol Hypertens (2017) 26(1):43–9. doi: 10.1097/MNH.0000000000000294 27755120

[38] Peiró C Romacho T Azcutia V Villalobos L Fernández E Bolaños JP. Inflammation, glucose, and vascular cell damage: the role of the pentose phosphate pathway. Cardiovasc Diabetol (2017) 16(1):25. doi: 10.1186/s12933-017-0502-1 28209202 PMC5314630

[39] Xu SN Wang TS Li X Wang YP. SIRT2 activates G6PD to enhance NADPH production and promote leukaemia cell proliferation. Sci Rep (2016) 6:32734. doi: 10.1038/srep32734 27586085 PMC5009355

[40] Gottschalk S Anderson N Hainz C Eckhardt SG Serkova NJ. Imatinib (STI571)-mediated changes in glucose metabolism in human leukemia BCR-ABL-positive cells. Clin Cancer Res (2004) 10:6661–8. doi: 10.1158/1078-0432.CCR-04-0039 15475456

[41] Williams RM Yates LA Zhang X. Structures and regulations of ATM and ATR, master kinases in genome integrity. Curr Opin Struct Biol (2020) 61:98–105. doi: 10.1016/j.sbi.2019.12.010 31924595

[42] Gregory MA D’Alessandro A Alvarez-Calderon F Kim J Nemkov T Adane B. ATM/G6PD-driven redox metabolism promotes FLT3 inhibitor resistance in acute myeloid leukemia. Proc Natl Acad Sci USA (2016) 113(43):E6669–78. doi: 10.1073/pnas.1603876113 PMC5086999 27791036

[43] Chen X Xu Z Zhu Z Chen A Fu G Wang Y. Modulation of G6PD affects bladder cancer via ROS accumulation and the AKT pathway in vitro. Int J Oncol (2018) 53(4):1703–12. doi: 10.3892/ijo.2018.4501 30066842

[44] Zhang Q Han Q Yang Z Ni Y Agbana YL Bai H. G6PD facilitates clear cell renal cell carcinoma invasion by enhancing MMP2 expression through ROS−MAPK axis pathway. Int J Oncol (2020) 57(1):197–212. doi: 10.3892/ijo.2020.5041 32319593 PMC7252464

[45] Tsai IC Pan ZC Cheng HP Liu CH Lin BT Jiang MJ. Reactive oxygen species derived from NADPH oxidase 1 and mitochondria mediate angiotensin II-induced smooth muscle cell senescence. J Mol Cell Cardiol (2016) 98:18–27. doi: 10.1016/j.yjmcc.2016.07.001 27381955

[46] Cairns RA Harris IS Mak TW. Regulation of cancer cell metabolism. Nat Rev Cancer (2011) 11:85–95. doi: 10.1038/nrc2981 21258394

[47] Hamanaka RB Chandel NS. Mitochondrial reactive oxygen species regulate cellular signaling and dictate biological outcomes. Trends Biochem Sci (2010) 35:505–13. doi: 10.1016/j.tibs.2010.04.002 PMC2933303 20430626

[48] Maryanovich M Gross A. A ROS rheostat for cell fate regulation. Trends Cell Biol (2013) 23(3):129–34. doi: 10.1016/j.tcb.2012.09.007 23117019

[49] Meitzler JL Antony S Wu Y Juhasz A Liu H Jiang G. NADPH oxidases:a perspective on reactive oxygen species production in tumor biology. Antioxid Redox Signal (2014) 20:2873–89. doi: 10.1089/ars.2013.5603 PMC4026372 24156355

[50] Weyemi U Redon CE Parekh PR Dupuy C Bonner WM. NADPH oxidases NOXs and DUOXs as putative targets for cancer therapy. Anticancer Agents Med Chem (2013) 13:502–14. PMC6365101 22931418

[51] Lee JB Khan DH Hurren R Xu M Na Y Kang H. Venetoclax enhances T cell-mediated antileukemic activity by increasing ROS production. Blood (2021) 138(3):234–45. doi: 10.1182/blood.2020009081 PMC8310428 34292323

[52] Zhang Y Choksi S Chen K Pobezinskaya Y Linnoila I Liu ZG. ROS play a critical role in the differentiation of alternatively activated macrophages and the occurrence of tumor-associated macrophages. Cell Res (2013) 23(7):898–914. doi: 10.1038/cr.2013.75 23752925 PMC3698641

[53] Kapoor N Niu J Saad Y Kumar S Sirakova T Becerra E. Transcription factors STAT6 and KLF4 implement macrophage polarization via the dual catalytic powers of MCPIP. J Immunol (2015) 194(12):6011–23. doi: 10.4049/jimmunol.1402797 PMC4458412 25934862

[54] Shan M Qin J Jin F Han X Guan H Li X. Autophagy suppresses isoprenaline-induced M2 macrophage polarization via the ROS/ERK and mTOR signaling pathway. Free Radic Biol Med (2017) 110:432–43. doi: 10.1016/j.freeradbiomed.2017.05.021 28647611

[55] Aerbajinai W Ghosh MC Liu J Kumkhaek C Zhu J Chin K. Glia maturation factor-γ regulates murine macrophage iron metabolism and M2 polarization through mitochondrial ROS. Blood Adv (2019) 3(8):1211–25. doi: 10.1182/bloodadvances.2018026070 PMC6482362 30971398

[56] Griess B Mir S Datta K Teoh-Fitzgerald M. Scavenging reactive oxygen species selectively inhibits M2 macrophage polarization and their pro-tumorigenic function in part, via Stat3 suppression. Free Radic Biol Med (2020) 147:48–60. doi: 10.1016/j.freeradbiomed.2019.12.018 31863907 PMC10035558

[57] Duy C Li M Teater M Meydan C Garrett-Bakelman FE Lee TC. Chemotherapy induces senescence-like resilient cells capable of initiating AML recurrence. Cancer Discov (2021) 11(6):1542–61. doi: 10.1158/2159-8290.CD-20-1375 PMC8178167 33500244

[58] Sarode P Schaefer MB Grimminger F Seeger W Savai R. Macrophage and tumor cell cross-talk is fundamental for lung tumor progression: we need to talk. Front Oncol (2020) 10:324. doi: 10.3389/fonc.2020.00324 32219066 PMC7078651

[59] Yan H Qu J Cao W Liu Y Zheng G Zhang E. Identification of prognostic genes in the acute myeloid leukemia immune microenvironment based on TCGA data analysis. Cancer Immunol Immunother (2019) 68(12):1971–8. doi: 10.1007/s00262-019-02408-7 PMC11028253 31650199

[60] Thommen DS Schumacher TN. T cell dysfunction in cancer. Cancer Cell (2018) 33(4):547–62. doi: 10.1016/j.ccell.2018.03.012 PMC7116508 29634943

[61] Wherry EJ Kurachi M. Molecular and cellular insights into T cell exhaustion. Nat Rev Immunol (2015) 15(8):486–99. doi: 10.1038/nri3862 PMC4889009 26205583

[62] Schietinger A Philip M Krisnawan VE Chiu EY Delrow JJ Basom RS. Tumor-specific T cell dysfunction is a dynamic antigen-driven differentiation program initiated early during tumorigenesis. Immunity (2016) 45(2):389–401. doi: 10.1016/j.immuni.2016.07.011 27521269 PMC5119632

[63] Kasakovski D Xu L Li Y. T cell senescence and CAR-T cell exhaustion in hematological Malignancies. J Hematol Oncol (2018) 11(1):91. doi: 10.1186/s13045-018-0629-x 29973238 PMC6032767

[64] Lichtenegger FS Krupka C Haubner S Köhnke T Subklewe M. Recent developments in immunotherapy of acute myeloid leukemia. J Hematol Oncol (2017) 10(1):142. doi: 10.1186/s13045-017-0505-0 28743264 PMC5526264

[65] Lee JB Chen B Vasic D Law AD Zhang L. Cellular immunotherapy for acute myeloid leukemia: How specific should it be? Blood Rev (2019) 35:18–31. doi: 10.1016/j.blre.2019.02.001 30826141

[66] Topalian SL Taube JM Anders RA Pardoll DM. Mechanism-driven biomarkers to guide immune checkpoint blockade in cancer therapy. Nat Rev Cancer (2016) 16(5):275–87. doi: 10.1038/nrc.2016.36 PMC5381938 27079802

[67] Ravandi F Assi R Daver N Benton CB Kadia T Thompson PA. Idarubicin, cytarabine, and nivolumab in patients with newly diagnosed acute myeloid leukaemia or high-risk myelodysplastic syndrome: a single-arm, phase 2 study. Lancet Haematol (2019) 6(9):e480–8. doi: 10.1016/S2352-3026(19)30114-0 PMC6778960 31400961

[68] Daver N Garcia-Manero G Basu S Boddu PC Alfayez M Cortes JE. Efficacy, safety, and biomarkers of response to azacitidine and nivolumab in relapsed/refractory acute myeloid leukemia: A nonrandomized, open-label, phase II study. Cancer Discov (2019) 9(3):370–83. doi: 10.1158/2159-8290.CD-18-0774 PMC6397669 30409776

[69] Konopleva M Contractor R Tsao T Samudio I Ruvolo PP Kitada S. Mechanisms of apoptosis sensitivity and resistance to the BH3 mimetic ABT-737 in acute myeloid leukemia. Cancer Cell (2006) 10(5):375–88. doi: 10.1016/j.ccr.2006.10.006 17097560

[70] Kasper S Breitenbuecher F Heidel F Hoffarth S Markova B Schuler M. Targeting MCL-1 sensitizes FLT3-ITD-positive leukemias to cytotoxic therapies. Blood Cancer J (2012) 2(3):e60. doi: 10.1038/bcj.2012.5 22829255 PMC3317524

[71] Liu Q Liu J Huang X. Unraveling the mystery: How bad is BAG3 in hematological Malignancies? Biochim Biophys Acta Rev Cancer (2022) 1877(5):188781. doi: 10.1016/j.bbcan.2022.188781 35985611

[72] Fu D Zhang B Wu S Zhang Y Xie J Ning W. Prognosis and characterization of immune microenvironment in acute myeloid leukemia through identification of an autophagy-related signature. Front Immunol (2021) 12:695865. doi: 10.3389/fimmu.2021.695865 34135913 PMC8200670

[73] Valdez BC Murray D Ramdas L de Lima M Jones R Kornblau S. Altered gene expression in busulfan-resistant human myeloid leukemia. Leuk Res (2008) 32(11):1684–97. doi: 10.1016/j.leukres.2008.01.016 PMC2633244 18339423

[74] Xu WD Li R Huang AF. Role of TL1A in inflammatory autoimmune diseases: A comprehensive review. Front Immunol (2022) 13:891328. doi: 10.3389/fimmu.2022.891328 35911746 PMC9329929

[75] Bittner S Ehrenschwender M. Multifaceted death receptor 3 signalingPromoting survival and triggering death. FEBS Lett (2017) 591(17):2543–55. doi: 10.1002/1873-3468.12747 28686297

[76] Greten FR Grivennikov SI. Inflammation and cancer: triggers, mechanisms, and consequences. Immunity (2019) 51(1):27–41. doi: 10.1016/j.immuni.2019.06.025 31315034 PMC6831096

[77] Dehm SM Bonham K. SRC gene expression in human cancer: the role of transcriptional activation. Biochem Cell Biol (2004) 82(2):263–74. doi: 10.1139/o03-077 15060621

[78] Tao R Li Q Gao X Ma L. Overexpression of GRK6 associates with the progression and prognosis of colorectal carcinoma. Oncol Lett (2018) 15(4):5879–86. doi: 10.3892/ol.2018.8030 PMC5840531 29552218

[79] Scheicher R Hoelbl-Kovacic A Bellutti F Tigan AS Prchal-Murphy M Heller G. CDK6 as a key regulator of hematopoietic and leukemic stem cell activation. Blood (2015) 125(1):90–101. doi: 10.1182/blood-2014-06-584417 25342715 PMC4281832

[80] Barman PK Shin JE Lewis SA Kang S Wu D Wang Y. Production of MHCII-expressing classical monocytes increases during aging in mice and humans. Aging Cell (2022) 21(10):e13701. doi: 10.1111/acel.13701 36040389 PMC9577948

[81] Hang Q Riley-Gillis B Han L Jia Y Lodi A Zhang H. Activation of RAS/MAPK pathway confers MCL-1 mediated acquired resistance to BCL-2 inhibitor venetoclax in acute myeloid leukemia. Signal Transduct Target Ther (2022) 7(1):51. doi: 10.1038/s41392-021-00870-3 35185150 PMC8858957

[82] Chen L Chen W Mysliwski M Serio J Ropa J Abulwerdi FA. Mutated Ptpn11 alters leukemic stem cell frequency and reduces the sensitivity of acute myeloid leukemia cells to Mcl1 inhibition. Leukemia (2015) 29(6):1290–300. doi: 10.1038/leu.2015.18 PMC4456293 25650089

[83] Wei AH Strickland SA Jr Hou JZ Fiedler W Lin TL Walter RB. Venetoclax combined with low-dose cytarabine for previously untreated patients with acute myeloid leukemia: results from a phase Ib/II study. J Clin Oncol (2019) 37(15):1277–84. doi: 10.1200/JCO.18.01600 PMC6524989 30892988

[84] Schwartz J Niu X Walton E Hurley L Lin H Edwards H. Synergistic anti-leukemic interactions between ABT-199 and panobinostat in acute myeloid leukemia ex vivo. Am J Transl Res (2016) 8(9):3893–902. PMC5040686 27725868

