# Supplementary material for: Identification of a venetoclax-resistance prognostic signature base on 6-senescence genes and its clinical significance for acute myeloid leukemia
Source: Front Oncol. 2023 Nov 30;13:1302356. doi: 10.3389/fonc.2023.1302356 (PMC10720639; doi:10.3389/fonc.2023.1302356)
Supplement: Supplementary file 1 [file DataSheet_1.doc]

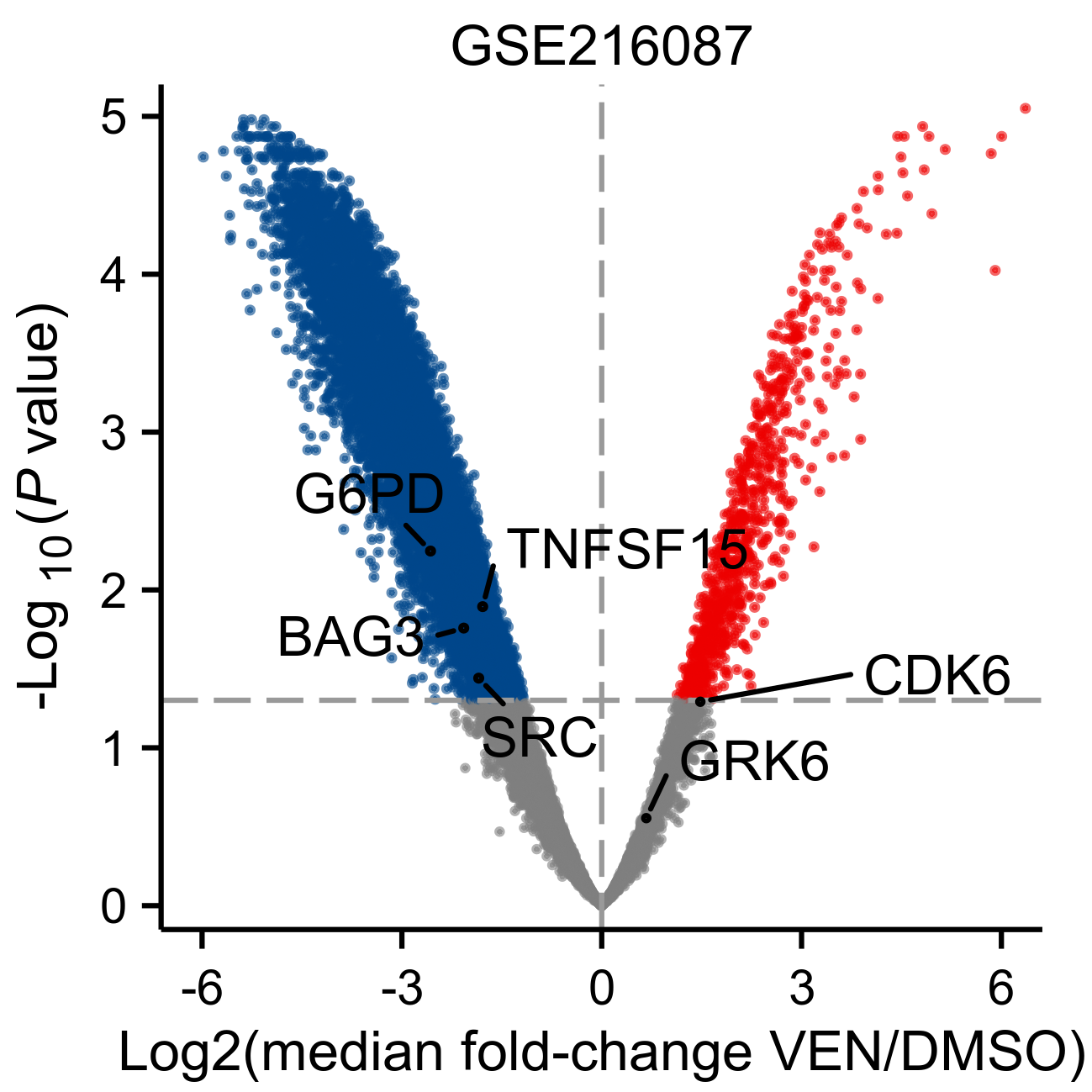


**Supplementary Figure S1. Differentially expressed levels of six-sgRNAs (*G6PD*, *BAG3*, *SRC*, *TNFSF15*, *GRK6* and *CDK6*) for single-VEN treatment relative to DMSO control in OCI-AML2 cells.** The results showed that four sgRNAs (except GRK6) were depleted significantly on OCI-AML2 cells after single-VEN treatment. Select genes highlighted in blue in the left indicated that knockdown of these four genes increased sensitivity to VEN. Conversely, high expression of these genes could decrease sensitivity to VEN and contribute to resistance.

**Supplementary Table S1. The list of 279 human senescence-related genes.**

| **Genes** | **Effect** | **Genes** | **Effect** | **Genes** | **Effect** |
| --- | --- | --- | --- | --- | --- |
| *AAK1* | Induces | *ACLY* | Inhibits | *KDM4A* | Unclear |
| *ABI3* | Induces | *AKR1B1* | Inhibits | *KSR2* | Unclear |
| *ADCK5* | Induces | *ASPH* | Inhibits | *LEO1* | Unclear |
| *AGT* | Induces | *ATF7IP* | Inhibits | *NR2E1* | Unclear |
| *AKT1* | Induces | *ATM* | Inhibits | *PIK3C2A* | Unclear |
| *ALOX15B* | Induces | *AURKA* | Inhibits |  |  |
| *AR* | Induces | *BAG3* | Inhibits |  |  |
| *ARPC1B* | Induces | *BCL6* | Inhibits |  |  |
| *ASF1A* | Induces | *BLVRA* | Inhibits |  |  |
| *AXL* | Induces | *BMI1* | Inhibits |  |  |
| *BHLHE40* | Induces | *BRCA1* | Inhibits |  |  |
| *BLK* | Induces | *BTG3* | Inhibits |  |  |
| *BRAF* | Induces | *C11orf31* | Inhibits |  |  |
| *BRD7* | Induces | *CBX7* | Inhibits |  |  |
| *CAV1* | Induces | *CBX8* | Inhibits |  |  |
| *CCND1* | Induces | *CDK1* | Inhibits |  |  |
| *CDK18* | Induces | *CDK2AP1* | Inhibits |  |  |
| *CDKN1A* | Induces | *CDK6* | Inhibits |  |  |
| *CDKN1C* | Induces | *CDK4* | Inhibits |  |  |
| *CDKN1B* | Induces | *CENPA* | Inhibits |  |  |
| *CDKN2A* | Induces | *CSNK1A1* | Inhibits |  |  |
| *CDKN2AIP* | Induces | *CTNNAL1* | Inhibits |  |  |
| *CDKN2B* | Induces | *CSNK2A1* | Inhibits |  |  |
| *CEBPB* | Induces | *DEK* | Inhibits |  |  |
| *CHEK1* | Induces | *DGCR8* | Inhibits |  |  |
| *CKB* | Induces | *DLX2* | Inhibits |  |  |
| *CPEB1* | Induces | *DHX9* | Inhibits |  |  |
| *CXCL1* | Induces | *DPY30* | Inhibits |  |  |
| *DDB2* | Induces | *DUSP3* | Inhibits |  |  |
| *CYR61* | Induces | *DUSP16* | Inhibits |  |  |
| *DHCR24* | Induces | *ENDOG* | Inhibits |  |  |
| *E2F1* | Induces | *EPHA3* | Inhibits |  |  |
| *EHF* | Induces | *EZH2* | Inhibits |  |  |
| *ERRFI1* | Induces | *FOXM1* | Inhibits |  |  |
| *ETS1* | Induces | *FOS* | Inhibits |  |  |
| *ETS2* | Induces | *FOXO3* | Inhibits |  |  |
| *EWSR1* | Induces | *FXR1* | Inhibits |  |  |
| *FASTK* | Induces | *G6PD* | Inhibits |  |  |
| *FBXO31* | Induces | *GAPDH* | Inhibits |  |  |
| *GKN1* | Induces | *GLB1* | Inhibits |  |  |
| *GATA4* | Induces | *HDAC4* | Inhibits |  |  |
| *GNG11* | Induces | *HJURP* | Inhibits |  |  |
| *GRK6* | Induces | *HIVEP1* | Inhibits |  |  |
| *HDAC1* | Induces | *HMGB1* | Inhibits |  |  |
| *HEPACAM* | Induces | *HSPA5* | Inhibits |  |  |
| *HK3* | Induces | *HSPB2* | Inhibits |  |  |
| *HRAS* | Induces | *ID1* | Inhibits |  |  |
| *ID4* | Induces | *IGFBP6* | Inhibits |  |  |
| *IGFBP1* | Induces | *KCNJ12* | Inhibits |  |  |
| *IFNG* | Induces | *KDM5B* | Inhibits |  |  |
| *IGFBP3* | Induces | *KIAA1524* | Inhibits |  |  |
| *IGFBP5* | Induces | *KL* | Inhibits |  |  |
| *IL1A* | Induces | *LATS1* | Inhibits |  |  |
| *IL8* | Induces | *LGALS3* | Inhibits |  |  |
| *ING1* | Induces | *LIMA1* | Inhibits |  |  |
| *ING2* | Induces | *MAGEA2* | Inhibits |  |  |
| *IRF3* | Induces | *MAGOH* | Inhibits |  |  |
| *IRF5* | Induces | *MAD2L1* | Inhibits |  |  |
| *IRF7* | Induces | *MAGOHB* | Inhibits |  |  |
| *ITPK1* | Induces | *MAP2K3* | Inhibits |  |  |
| *ITGB4* | Induces | *MAP4K1* | Inhibits |  |  |
| *ITPKB* | Induces | *MARCH5* | Inhibits |  |  |
| *ITSN2* | Induces | *MCL1* | Inhibits |  |  |
| *LIMK1* | Induces | *MDH1* | Inhibits |  |  |
| *MAP2K1* | Induces | *MECP2* | Inhibits |  |  |
| *MAP2K2* | Induces | *MMP9* | Inhibits |  |  |
| *MAP2K6* | Induces | *MYC* | Inhibits |  |  |
| *MAP3K6* | Induces | *MYLK* | Inhibits |  |  |
| *MAP2K7* | Induces | *NANOG* | Inhibits |  |  |
| *MAP3K7* | Induces | *NEK1* | Inhibits |  |  |
| *MAPK12* | Induces | *NEK6* | Inhibits |  |  |
| *MAPKAPK5* | Induces | *NFE2L2* | Inhibits |  |  |
| *MAPK14* | Induces | *NTN4* | Inhibits |  |  |
| *MAST1* | Induces | *P3H1* | Inhibits |  |  |
| *MATK* | Induces | *PATZ1* | Inhibits |  |  |
| *MCRS1* | Induces | *POT1* | Inhibits |  |  |
| *MOB3A* | Induces | *PPM1B* | Inhibits |  |  |
| *MORC3* | Induces | *PPM1D* | Inhibits |  |  |
| *MORF4* | Induces | *PRMT6* | Inhibits |  |  |
| *MXD4* | Induces | *PRPF19* | Inhibits |  |  |
| *MVK* | Induces | *PSMB5* | Inhibits |  |  |
| *NADK* | Induces | *PSMD14* | Inhibits |  |  |
| *NDRG1* | Induces | *RAD21* | Inhibits |  |  |
| *NEK4* | Induces | *RBP2* | Inhibits |  |  |
| *NINJ1* | Induces | *RBX1* | Inhibits |  |  |
| *NOTCH3* | Induces | *RSL1D1* | Inhibits |  |  |
| *NOX4* | Induces | *RUVBL2* | Inhibits |  |  |
| *NUAK1* | Induces | *SENP1* | Inhibits |  |  |
| *OTX2* | Induces | *SENP2* | Inhibits |  |  |
| *PAK4* | Induces | *SENP7* | Inhibits |  |  |
| *PBRM1* | Induces | *SGK1* | Inhibits |  |  |
| *PCGF2* | Induces | *SIRT1* | Inhibits |  |  |
| *PDCD10* | Induces | *SIRT6* | Inhibits |  |  |
| *PDIK1L* | Induces | *SIX1* | Inhibits |  |  |
| *PDZD2* | Induces | *SLC16A7* | Inhibits |  |  |
| *PDPK1* | Induces | *SMARCA4* | Inhibits |  |  |
| *PEBP1* | Induces | *SMG1* | Inhibits |  |  |
| *PEX19* | Induces | *SNAI1* | Inhibits |  |  |
| *PIAS4* | Induces | *SOD1* | Inhibits |  |  |
| *PIK3R5* | Induces | *SRC* | Inhibits |  |  |
| *PIM1* | Induces | *SUPT5H* | Inhibits |  |  |
| *PLA2R1* | Induces | *TACC3* | Inhibits |  |  |
| *PKM* | Induces | *TERC* | Inhibits |  |  |
| *PML* | Induces | *TBX2* | Inhibits |  |  |
| *PNPT1* | Induces | *TERF2* | Inhibits |  |  |
| *PMVK* | Induces | *TERT* | Inhibits |  |  |
| *POU5F1* | Induces | *TFDP1* | Inhibits |  |  |
| *PRKCH* | Induces | *TMSB4X* | Inhibits |  |  |
| *PRKCD* | Induces | *TNFSF13* | Inhibits |  |  |
| *PROX1* | Induces | *TPR* | Inhibits |  |  |
| *PTRF* | Induces | *TRPM8* | Inhibits |  |  |
| *PTTG1* | Induces | *TXN* | Inhibits |  |  |
| *RAF1* | Induces | *USP1* | Inhibits |  |  |
| *RB1* | Induces | *VEGFA* | Inhibits |  |  |
| *RNASEL* | Induces | *WNT2* | Inhibits |  |  |
| *RPS6KA6* | Induces | *WRN* | Inhibits |  |  |
| *RUNX1* | Induces | *WT1* | Inhibits |  |  |
| *SERPINE1* | Induces | *WWP1* | Inhibits |  |  |
| *SFN* | Induces | *YAP1* | Inhibits |  |  |
| *SIK1* | Induces | *ZMAT3* | Inhibits |  |  |
| *SIN3B* | Induces | *ZNF148* | Inhibits |  |  |
| *SLC13A3* | Induces |  | Inhibits |  |  |
| *SMARCB1* | Induces |  | Inhibits |  |  |
| *SMURF2* | Induces |  | Inhibits |  |  |
| *SOCS1* | Induces |  | Inhibits |  |  |
| *SORBS2* | Induces |  | Inhibits |  |  |
| *SOX2* | Induces |  | Inhibits |  |  |
| *SPIN1* | Induces |  | Inhibits |  |  |
| *SOX5* | Induces |  | Inhibits |  |  |
| *SP1* | Induces |  | Inhibits |  |  |
| *SPOP* | Induces |  | Inhibits |  |  |
| *SREBF1* | Induces |  | Inhibits |  |  |
| *SRSF1* | Induces |  | Inhibits |  |  |
| *STAT5B* | Induces |  | Inhibits |  |  |
| *STK32C* | Induces |  | Inhibits |  |  |
| *STK40* | Induces |  | Inhibits |  |  |
| *SYK* | Induces |  | Inhibits |  |  |
| *TFAP4* | Induces |  | Inhibits |  |  |
| *TGFB1I1* | Induces |  | Inhibits |  |  |
| *TLR3* | Induces |  | Inhibits |  |  |
| *TNFSF15* | Induces |  | Inhibits |  |  |
| *TOP1* | Induces |  | Inhibits |  |  |
| *TP63* | Induces |  | Inhibits |  |  |
| *TP53* | Induces |  | Inhibits |  |  |
| *TRIM28* | Induces |  | Inhibits |  |  |
| *TXNIP* | Induces |  | Inhibits |  |  |
| *UBTD1* | Induces |  | Inhibits |  |  |
| *TYK2* | Induces |  | Inhibits |  |  |
| *VENTX* | Induces |  | Inhibits |  |  |
| *WNT16* | Induces |  | Inhibits |  |  |
| *XAF1* | Induces |  | Inhibits |  |  |
| *YPEL3* | Induces |  | Inhibits |  |  |
| *ZFP36* | Induces |  | Inhibits |  |  |

**Supplementary Table S2. Differential expression genes between VEN-sensitive and -resistant samples.**

| **Genes** | **log2FoldChange** | **lfcSE** | **stat** | **P value** | **P adj** |
| --- | --- | --- | --- | --- | --- |
| *CCL2* | 8.045077794 | 0.718760377 | 11.19299012 | 4.41E-29 | 5.82E-26 |
| *IGFN1* | 7.818529134 | 1.268006322 | 6.166001699 | 7.00E-10 | 2.15E-08 |
| *DAAM2* | 7.004182143 | 0.596487265 | 11.74238338 | 7.73E-32 | 1.63E-28 |
| *CCL7* | 6.292083315 | 1.193824495 | 5.270526229 | 1.36E-07 | 2.34E-06 |
| *TRPV4* | 6.157611114 | 0.573687163 | 10.73339532 | 7.09E-27 | 6.24E-24 |
| *FN1* | 5.913225165 | 0.624623629 | 9.466861145 | 2.88E-21 | 7.74E-19 |
| *OLAH* | 5.870740715 | 0.700245471 | 8.383832471 | 5.12E-17 | 6.62E-15 |
| *DUSP13* | 5.861068377 | 0.607988152 | 9.640102943 | 5.41E-22 | 1.66E-19 |
| *MUCL1* | 5.854417693 | 1.357483206 | 4.312699906 | 1.61E-05 | 0.000147121 |
| *HS3ST5* | 5.795837264 | 0.75004042 | 7.727366562 | 1.10E-14 | 8.89E-13 |
| *IL6* | 5.7143132 | 0.60656824 | 9.420726022 | 4.48E-21 | 1.17E-18 |
| *RPH3A* | 5.693898761 | 0.534884131 | 10.64510692 | 1.84E-26 | 1.42E-23 |
| *FAM20A* | 5.693271188 | 0.523256294 | 10.88046384 | 1.43E-27 | 1.51E-24 |
| *CD14* | 5.644334066 | 0.401745507 | 14.04952628 | 7.76E-45 | 1.23E-40 |
| *MAFB* | 5.589354817 | 0.386952082 | 14.44456583 | 2.71E-47 | 8.59E-43 |
| *LTF* | 5.500649842 | 0.529683006 | 10.38479578 | 2.91E-25 | 1.67E-22 |
| *OLFM4* | 5.448126147 | 0.547936367 | 9.942990608 | 2.71E-23 | 1.08E-20 |
| *THBS1* | 5.435345066 | 0.53165195 | 10.22350254 | 1.56E-24 | 8.01E-22 |
| *CHI3L1* | 5.42272152 | 0.544394092 | 9.961021984 | 2.26E-23 | 9.28E-21 |
| *LCN2* | 5.404260706 | 0.537225897 | 10.0595685 | 8.34E-24 | 3.67E-21 |
| *SASH1* | 5.37639116 | 0.575975537 | 9.334408873 | 1.02E-20 | 2.51E-18 |
| *SLC46A2* | 5.370783149 | 0.551409751 | 9.740094625 | 2.03E-22 | 6.71E-20 |
| *DEFA3* | 5.33301202 | 0.541740146 | 9.844225236 | 7.26E-23 | 2.64E-20 |
| *MARCO* | 5.311148434 | 0.43646332 | 12.16860201 | 4.57E-34 | 1.81E-30 |
| *TRHDE* | 5.307898282 | 0.846843001 | 6.2678658 | 3.66E-10 | 1.23E-08 |
| *DEFA1B* | 5.294717505 | 0.576811949 | 9.179278469 | 4.34E-20 | 9.47E-18 |
| *PGA3* | 5.287016238 | 0.979016008 | 5.400336863 | 6.65E-08 | 1.23E-06 |
| *ATP2C2* | 5.278926813 | 0.547667397 | 9.638928371 | 5.48E-22 | 1.67E-19 |
| *CYP1B1* | 5.199183829 | 0.558613368 | 9.307302921 | 1.31E-20 | 3.22E-18 |
| *CAMP* | 5.189638958 | 0.516638738 | 10.04500548 | 9.66E-24 | 4.13E-21 |
| *DEFA1* | 5.171846606 | 0.603195872 | 8.574074936 | 9.99E-18 | 1.50E-15 |
| *NRG1* | 5.135604263 | 0.623854862 | 8.232049748 | 1.84E-16 | 2.10E-14 |
| *CHIT1* | 5.087160169 | 0.472806 | 10.75950849 | 5.35E-27 | 4.98E-24 |
| *NECAB1* | 5.021449244 | 0.742324613 | 6.764492452 | 1.34E-11 | 6.16E-10 |
| *ARG1* | 4.991530977 | 0.478701475 | 10.42723123 | 1.86E-25 | 1.14E-22 |
| *STAB2* | 4.97041748 | 0.623818774 | 7.967726672 | 1.62E-15 | 1.57E-13 |
| *ARHGAP29* | 4.941126899 | 0.501911321 | 9.844621335 | 7.23E-23 | 2.64E-20 |
| *IL31RA* | 4.934906695 | 0.578998577 | 8.523175855 | 1.55E-17 | 2.16E-15 |
| *PGLYRP1* | 4.913601206 | 0.482032317 | 10.19350993 | 2.12E-24 | 9.87E-22 |
| *AMPH* | 4.889922141 | 0.683151911 | 7.157884013 | 8.19E-13 | 4.66E-11 |
| *PLBD1* | 4.878304928 | 0.426183481 | 11.44648994 | 2.45E-30 | 3.66E-27 |
| *MMP27* | 4.876987294 | 0.710670974 | 6.862510885 | 6.77E-12 | 3.30E-10 |
| *LSAMP* | 4.872416245 | 0.874353688 | 5.572591859 | 2.51E-08 | 5.24E-07 |
| *LRRC32* | 4.856484316 | 0.532917377 | 9.1130155 | 8.01E-20 | 1.63E-17 |
| *PROKR2* | 4.842910315 | 0.847078299 | 5.71719323 | 1.08E-08 | 2.47E-07 |
| *MECOM* | 4.837451262 | 0.784203383 | 6.1686182 | 6.89E-10 | 2.12E-08 |
| *PLA2G7* | 4.796119397 | 0.488212948 | 9.823826707 | 8.89E-23 | 3.13E-20 |
| *CRISP3* | 4.721485526 | 0.606535116 | 7.784356427 | 7.01E-15 | 5.98E-13 |
| *IL1R2* | 4.705597684 | 0.48260326 | 9.750447371 | 1.84E-22 | 6.17E-20 |
| *MMP8* | 4.701515578 | 0.509716724 | 9.223781282 | 2.87E-20 | 6.63E-18 |
| *SMPDL3A* | 4.683448062 | 0.43434137 | 10.78287354 | 4.15E-27 | 3.98E-24 |
| *CD300E* | 4.641008323 | 0.473544101 | 9.800583128 | 1.12E-22 | 3.81E-20 |
| *SIGLEC1* | 4.639934406 | 0.483012002 | 9.60625074 | 7.52E-22 | 2.25E-19 |
| *VCAN* | 4.617108117 | 0.48363125 | 9.54675306 | 1.34E-21 | 3.82E-19 |
| *G0S2* | 4.615707911 | 0.502565086 | 9.184298795 | 4.14E-20 | 9.23E-18 |
| *TMTC1* | 4.615012964 | 0.601602234 | 7.671203174 | 1.70E-14 | 1.34E-12 |
| *C5AR1* | 4.611692666 | 0.355511599 | 12.97198931 | 1.76E-38 | 1.86E-34 |
| *S100A8* | 4.608112974 | 0.381696697 | 12.07270855 | 1.47E-33 | 3.88E-30 |
| *VSIG4* | 4.57477802 | 0.501437072 | 9.123334269 | 7.28E-20 | 1.50E-17 |
| *TRPC6* | 4.556929327 | 0.780046805 | 5.84186654 | 5.16E-09 | 1.29E-07 |
| *WIF1* | 4.55039081 | 0.839047589 | 5.423280955 | 5.85E-08 | 1.11E-06 |
| *ABCC3* | 4.546840444 | 0.390762831 | 11.63580587 | 2.71E-31 | 4.77E-28 |
| *SLC25A21* | 4.542128343 | 0.849322913 | 5.347940431 | 8.90E-08 | 1.61E-06 |
| *SLC7A7* | 4.527452622 | 0.352041332 | 12.86057121 | 7.50E-38 | 5.94E-34 |
| *PDK4* | 4.521455648 | 0.470724376 | 9.605314447 | 7.59E-22 | 2.25E-19 |
| *DEFA5* | 4.51089821 | 0.866119285 | 5.208172 | 1.91E-07 | 3.16E-06 |
| *CD163* | 4.489599038 | 0.507382357 | 8.848551742 | 8.87E-19 | 1.57E-16 |
| *ANKRD34B* | 4.468340347 | 0.579619962 | 7.709086371 | 1.27E-14 | 1.02E-12 |
| *HMOX1* | 4.467241144 | 0.374846321 | 11.91752699 | 9.59E-33 | 2.17E-29 |
| *LRP1* | 4.462876986 | 0.399283185 | 11.17722247 | 5.27E-29 | 6.67E-26 |
| *ADGRL2* | 4.461592128 | 0.574441734 | 7.766831453 | 8.05E-15 | 6.74E-13 |
| *OLR1* | 4.45312826 | 0.448386012 | 9.931461149 | 3.04E-23 | 1.20E-20 |
| *C1QA* | 4.449032396 | 0.451124381 | 9.862096987 | 6.08E-23 | 2.29E-20 |
| *ADORA1* | 4.444759503 | 0.68712868 | 6.468598432 | 9.89E-11 | 3.75E-09 |
| *SLC16A6* | 4.442531797 | 0.355098911 | 12.51068832 | 6.53E-36 | 3.44E-32 |
| *TMEM176B* | 4.41498864 | 0.507974767 | 8.691354235 | 3.58E-18 | 5.87E-16 |
| *S100A9* | 4.390480833 | 0.374192834 | 11.73320394 | 8.61E-32 | 1.70E-28 |
| *CHST15* | 4.390058716 | 0.362352056 | 12.11545139 | 8.75E-34 | 2.77E-30 |
| *GPBAR1* | 4.38420054 | 0.375356951 | 11.68008353 | 1.61E-31 | 3.00E-28 |
| *S100A12* | 4.375017622 | 0.418960626 | 10.44255081 | 1.58E-25 | 1.00E-22 |
| *TGFBI* | 4.371647568 | 0.442892326 | 9.870678063 | 5.58E-23 | 2.15E-20 |
| *CXCL16* | 4.371056231 | 0.380152811 | 11.49815577 | 1.35E-30 | 2.13E-27 |
| *CEACAM5* | 4.366086893 | 0.942750619 | 4.631221456 | 3.64E-06 | 4.12E-05 |
| *TMEM176A* | 4.342295593 | 0.506660523 | 8.570424179 | 1.03E-17 | 1.53E-15 |
| *HTRA4* | 4.332433873 | 0.456041222 | 9.500092675 | 2.10E-21 | 5.77E-19 |
| *GJB6* | 4.330103133 | 0.649947507 | 6.662235158 | 2.70E-11 | 1.14E-09 |
| *RTN1* | 4.326968786 | 0.42963088 | 10.07136354 | 7.39E-24 | 3.30E-21 |
| *FOLR3* | 4.324878094 | 0.424199441 | 10.19538849 | 2.08E-24 | 9.82E-22 |
| *CPLX2* | 4.294091694 | 0.921892356 | 4.657910076 | 3.19E-06 | 3.69E-05 |
| *OR6N1* | 4.293941845 | 0.740832181 | 5.796105996 | 6.79E-09 | 1.64E-07 |
| *PGM5* | 4.286393157 | 0.575364527 | 7.449873873 | 9.34E-14 | 6.32E-12 |
| *LILRA5* | 4.280354795 | 0.35356439 | 12.10629498 | 9.78E-34 | 2.81E-30 |
| *CAMK1G* | 4.276954416 | 1.041646148 | 4.10595712 | 4.03E-05 | 0.000320415 |
| *CRISP2* | 4.269102175 | 0.644852683 | 6.62027512 | 3.59E-11 | 1.49E-09 |
| *DMRTC2* | 4.266683493 | 0.822581956 | 5.186940293 | 2.14E-07 | 3.48E-06 |
| *PLPP3* | 4.265584799 | 0.448342559 | 9.514119753 | 1.83E-21 | 5.13E-19 |
| *RBP7* | 4.263953466 | 0.467293004 | 9.124796283 | 7.19E-20 | 1.49E-17 |
| *CSRNP3* | 4.262062164 | 1.019966853 | 4.178628112 | 2.93E-05 | 0.000243906 |
| *CEACAM8* | 4.253703335 | 0.505803023 | 8.409802113 | 4.11E-17 | 5.35E-15 |
| *SLC9A4* | 4.228310062 | 0.866095213 | 4.882038371 | 1.05E-06 | 1.41E-05 |
| *C1QC* | 4.22700366 | 0.480212107 | 8.802367957 | 1.34E-18 | 2.32E-16 |
| *TFF3* | 4.220914566 | 0.639990674 | 6.59527511 | 4.24E-11 | 1.74E-09 |
| *CTSL* | 4.219438771 | 0.467111144 | 9.033050959 | 1.67E-19 | 3.26E-17 |
| *MSR1* | 4.216709148 | 0.5585156 | 7.549850262 | 4.36E-14 | 3.12E-12 |
| *KCNK13* | 4.200866258 | 0.472525541 | 8.890241678 | 6.10E-19 | 1.11E-16 |
| *RAPH1* | 4.187564327 | 0.385340069 | 10.86719151 | 1.65E-27 | 1.69E-24 |
| *CSF3* | 4.181668873 | 0.675118726 | 6.193975535 | 5.87E-10 | 1.84E-08 |
| *AQP9* | 4.178435401 | 0.453602158 | 9.21167443 | 3.21E-20 | 7.31E-18 |
| *CA4* | 4.167719003 | 0.528843357 | 7.880819427 | 3.25E-15 | 2.93E-13 |
| *STC1* | 4.162021465 | 1.119721787 | 3.717013916 | 0.000201591 | 0.001261196 |
| *WLS* | 4.154976462 | 0.486900975 | 8.533514351 | 1.42E-17 | 2.02E-15 |
| *DEFA4* | 4.151718966 | 0.567013879 | 7.322076439 | 2.44E-13 | 1.53E-11 |
| *RGL1* | 4.147975327 | 0.387076905 | 10.71615297 | 8.55E-27 | 7.31E-24 |
| *OLFM1* | 4.144956688 | 0.751356281 | 5.516632777 | 3.46E-08 | 6.96E-07 |
| *C1QB* | 4.142872079 | 0.475422363 | 8.714087511 | 2.93E-18 | 4.86E-16 |
| *SLC15A3* | 4.130045101 | 0.336757495 | 12.26415199 | 1.41E-34 | 6.38E-31 |
| *ENHO* | 4.130022281 | 0.60919589 | 6.779465109 | 1.21E-11 | 5.60E-10 |
| *FOLR2* | 4.118918626 | 0.384604636 | 10.70948773 | 9.19E-27 | 7.65E-24 |
| *PDE2A* | 4.115283992 | 0.453281032 | 9.078879763 | 1.10E-19 | 2.18E-17 |
| *CLEC4E* | 4.110779501 | 0.431965243 | 9.51645895 | 1.79E-21 | 5.06E-19 |
| *SERPINB2* | 4.109083837 | 0.503299454 | 8.164292254 | 3.23E-16 | 3.45E-14 |
| *MMP9* | 4.102041531 | 0.47904327 | 8.562987501 | 1.10E-17 | 1.62E-15 |
| *CLEC7A* | 4.089102022 | 0.337418699 | 12.11877717 | 8.40E-34 | 2.77E-30 |
| *CPAMD8* | 4.076107115 | 0.439311698 | 9.278394219 | 1.72E-20 | 4.18E-18 |
| *COL17A1* | 4.06153997 | 0.529887452 | 7.664910646 | 1.79E-14 | 1.40E-12 |
| *ALOX12* | 4.058056754 | 0.493559328 | 8.222024229 | 2.00E-16 | 2.24E-14 |
| *SDC3* | 4.056556229 | 0.388132998 | 10.4514593 | 1.44E-25 | 9.32E-23 |
| *LILRA6* | 4.055253844 | 0.393394947 | 10.30835265 | 6.46E-25 | 3.59E-22 |
| *TENM4* | 4.042854529 | 0.573853627 | 7.04509711 | 1.85E-12 | 9.91E-11 |
| *LRRK2* | 4.038690059 | 0.385515809 | 10.4760686 | 1.11E-25 | 7.49E-23 |
| *CYP19A1* | 4.034810257 | 0.544520079 | 7.409846608 | 1.26E-13 | 8.34E-12 |
| *EPHB2* | 4.023817901 | 0.471518009 | 8.533752313 | 1.42E-17 | 2.02E-15 |
| *FGD5* | 4.022378612 | 0.615108987 | 6.539294166 | 6.18E-11 | 2.44E-09 |
| *QPCT* | 4.017562225 | 0.391989766 | 10.24915081 | 1.19E-24 | 6.41E-22 |
| *GLT1D1* | 4.011829458 | 0.408554439 | 9.819571326 | 9.27E-23 | 3.19E-20 |
| *FCN1* | 4.010221071 | 0.369478995 | 10.85371868 | 1.91E-27 | 1.89E-24 |
| *ORM1* | 4.008788503 | 0.531360213 | 7.54438967 | 4.54E-14 | 3.25E-12 |
| *CXCL1* | 4.003492867 | 0.435985846 | 9.182621188 | 4.21E-20 | 9.31E-18 |
| *TRIM7* | 3.999147365 | 0.397754052 | 10.05432213 | 8.79E-24 | 3.81E-21 |
| *SEPTIN10* | 3.99379561 | 0.449320682 | 8.888519423 | 6.19E-19 | 1.12E-16 |
| *HFE* | 3.993303907 | 0.398006534 | 10.03326218 | 1.09E-23 | 4.59E-21 |
| *CEACAM3* | 3.99312605 | 0.454649064 | 8.782875331 | 1.59E-18 | 2.74E-16 |
| *RGL3* | 3.987517635 | 0.553099772 | 7.20940025 | 5.62E-13 | 3.31E-11 |
| *CDKN2B* | 3.982879085 | 0.458561738 | 8.685589656 | 3.77E-18 | 6.15E-16 |
| *LYVE1* | 3.979893902 | 0.506230231 | 7.861825819 | 3.79E-15 | 3.37E-13 |
| *CCL20* | 3.973410393 | 0.654044895 | 6.07513402 | 1.24E-09 | 3.60E-08 |
| *IFI30* | 3.968861398 | 0.383808119 | 10.34074372 | 4.61E-25 | 2.61E-22 |
| *HPR* | 3.967710858 | 0.547716413 | 7.244097065 | 4.35E-13 | 2.61E-11 |
| *MYBPH* | 3.964432152 | 0.556969508 | 7.117862099 | 1.10E-12 | 6.07E-11 |
| *SIGLEC11* | 3.963161104 | 0.470300849 | 8.426863601 | 3.55E-17 | 4.72E-15 |
| *DENND2C* | 3.96191243 | 0.48094368 | 8.237788741 | 1.75E-16 | 2.00E-14 |
| *IL10* | 3.957013677 | 0.484926908 | 8.16002084 | 3.35E-16 | 3.56E-14 |
| *TRNP1* | 3.955801532 | 0.478451117 | 8.267932488 | 1.36E-16 | 1.60E-14 |
| *NMNAT2* | 3.927128188 | 0.505600824 | 7.767250372 | 8.02E-15 | 6.73E-13 |
| *SLC22A1* | 3.919833981 | 0.502708229 | 7.797433487 | 6.32E-15 | 5.48E-13 |
| *SIRPD* | 3.917370817 | 0.416851143 | 9.39752927 | 5.59E-21 | 1.45E-18 |
| *CLEC1A* | 3.916269904 | 0.425310894 | 9.208016902 | 3.32E-20 | 7.51E-18 |
| *HSD11B1* | 3.912120331 | 0.562215944 | 6.958394498 | 3.44E-12 | 1.76E-10 |
| *IER3* | 3.911775252 | 0.382963749 | 10.2144792 | 1.71E-24 | 8.45E-22 |
| *VEPH1* | 3.900189954 | 0.534502666 | 7.296857812 | 2.95E-13 | 1.83E-11 |
| *TGM3* | 3.899841315 | 0.600912619 | 6.48986424 | 8.59E-11 | 3.30E-09 |
| *C15orf48* | 3.894685054 | 0.524358196 | 7.427527758 | 1.11E-13 | 7.42E-12 |
| *CDA* | 3.892580038 | 0.367153694 | 10.60204514 | 2.92E-26 | 2.15E-23 |
| *IFITM10* | 3.889565146 | 0.406610486 | 9.56582598 | 1.11E-21 | 3.23E-19 |
| *VNN3* | 3.887935629 | 0.430741609 | 9.026143636 | 1.78E-19 | 3.45E-17 |
| *APOBEC3A* | 3.886114804 | 0.348699814 | 11.14458525 | 7.61E-29 | 9.27E-26 |
| *CDH6* | 3.881845721 | 0.753363225 | 5.152688099 | 2.57E-07 | 4.11E-06 |
| *EPB41L3* | 3.878983825 | 0.49440052 | 7.845832812 | 4.30E-15 | 3.79E-13 |
| *CABP5* | 3.878596245 | 0.889881127 | 4.358555459 | 1.31E-05 | 0.000123459 |
| *RAB19* | 3.877092003 | 0.465937644 | 8.321053364 | 8.72E-17 | 1.07E-14 |
| *HK3* | 3.858994144 | 0.368583926 | 10.46978413 | 1.19E-25 | 7.84E-23 |
| *NECAB2* | 3.855567408 | 0.548960218 | 7.023400393 | 2.17E-12 | 1.15E-10 |
| *CEACAM1* | 3.852145137 | 0.453129668 | 8.501198249 | 1.88E-17 | 2.56E-15 |
| *MYO7A* | 3.84253994 | 0.411236874 | 9.343860388 | 9.29E-21 | 2.32E-18 |
| *PRDM5* | 3.837145932 | 0.602211233 | 6.371760807 | 1.87E-10 | 6.74E-09 |
| *HP* | 3.829099543 | 0.418926169 | 9.140272981 | 6.23E-20 | 1.31E-17 |
| *WNT5A* | 3.822293084 | 0.73656895 | 5.189321498 | 2.11E-07 | 3.45E-06 |
| *P2RY12* | 3.805727628 | 0.490420268 | 7.760135282 | 8.48E-15 | 7.05E-13 |
| *NPFFR1* | 3.803538308 | 0.672289973 | 5.65758595 | 1.54E-08 | 3.37E-07 |
| *SEMG1* | 3.79913066 | 0.75817968 | 5.010857925 | 5.42E-07 | 7.90E-06 |
| *PCOLCE2* | 3.797131412 | 0.565267404 | 6.717407342 | 1.85E-11 | 8.21E-10 |
| *FPR1* | 3.792507774 | 0.417760291 | 9.078191149 | 1.10E-19 | 2.18E-17 |
| *RRAD* | 3.781478641 | 0.436032729 | 8.672465134 | 4.23E-18 | 6.86E-16 |
| *HIF3A* | 3.779948074 | 1.559675349 | 2.423548001 | 0.015369722 | 0.044941388 |
| *TWIST2* | 3.77161168 | 0.546254117 | 6.904500234 | 5.04E-12 | 2.51E-10 |
| *PCSK9* | 3.770065443 | 0.611657447 | 6.163687631 | 7.11E-10 | 2.18E-08 |
| *ARFGEF3* | 3.762389422 | 0.539084745 | 6.979217014 | 2.97E-12 | 1.53E-10 |
| *SGK1* | 3.759007659 | 0.356948842 | 10.53094229 | 6.22E-26 | 4.28E-23 |
| *C1QTNF1* | 3.747884457 | 0.694292862 | 5.398131917 | 6.73E-08 | 1.25E-06 |
| *FAM160A1* | 3.746258713 | 0.501407569 | 7.471484174 | 7.93E-14 | 5.43E-12 |
| *BPI* | 3.743791813 | 0.487513286 | 7.679363669 | 1.60E-14 | 1.26E-12 |
| *ADM* | 3.730836436 | 0.457469344 | 8.15538021 | 3.48E-16 | 3.68E-14 |
| *NR5A2* | 3.729377184 | 0.622126405 | 5.994565018 | 2.04E-09 | 5.67E-08 |
| *DNAAF1* | 3.725708423 | 0.435509961 | 8.554817932 | 1.18E-17 | 1.72E-15 |
| *GGTA1* | 3.723012044 | 0.431250022 | 8.633070957 | 5.97E-18 | 9.41E-16 |
| *KCNH3* | 3.712776274 | 0.380811888 | 9.749633337 | 1.85E-22 | 6.17E-20 |
| *PPP1R1B* | 3.706629902 | 0.626195907 | 5.919281586 | 3.23E-09 | 8.49E-08 |
| *PVALB* | 3.706419366 | 0.696579012 | 5.320888659 | 1.03E-07 | 1.84E-06 |
| *FABP4* | 3.698442485 | 0.986833451 | 3.747787919 | 0.000178401 | 0.001137455 |
| *STX1A* | 3.691424776 | 0.46852032 | 7.87890006 | 3.30E-15 | 2.97E-13 |
| *TLR8* | 3.687134448 | 0.462947722 | 7.964472609 | 1.66E-15 | 1.60E-13 |
| *MOGAT1* | 3.66545342 | 0.686271874 | 5.341109785 | 9.24E-08 | 1.67E-06 |
| *NWD1* | 3.657596036 | 0.530504667 | 6.894559577 | 5.40E-12 | 2.69E-10 |
| *PGA5* | 3.650139936 | 1.257223883 | 2.903333276 | 0.003692135 | 0.014007457 |
| *SECTM1* | 3.641743412 | 0.374166338 | 9.73295308 | 2.18E-22 | 7.12E-20 |
| *OR6N2* | 3.630332163 | 0.669519129 | 5.422297893 | 5.88E-08 | 1.11E-06 |
| *OASL* | 3.620107114 | 0.393440905 | 9.201145761 | 3.54E-20 | 7.95E-18 |
| *HEY1* | 3.61962241 | 0.542597676 | 6.670913956 | 2.54E-11 | 1.09E-09 |
| *CLEC6A* | 3.61487198 | 0.526562813 | 6.865034701 | 6.65E-12 | 3.26E-10 |
| *ALPL* | 3.612514153 | 0.522019684 | 6.920264239 | 4.51E-12 | 2.26E-10 |
| *PI3* | 3.612178349 | 0.565361793 | 6.389144779 | 1.67E-10 | 6.06E-09 |
| *PF4V1* | 3.608925063 | 0.677402439 | 5.327593846 | 9.95E-08 | 1.78E-06 |
| *PRG3* | 3.604175407 | 0.665256029 | 5.417726782 | 6.04E-08 | 1.14E-06 |
| *CD1B* | 3.600909626 | 0.691969938 | 5.203852695 | 1.95E-07 | 3.23E-06 |
| *MT1H* | 3.599104517 | 0.956166879 | 3.764096618 | 0.000167152 | 0.001078316 |
| *CD1D* | 3.583881236 | 0.402028535 | 8.914494684 | 4.90E-19 | 9.13E-17 |
| *P2RY13* | 3.581585758 | 0.317039133 | 11.29698318 | 1.36E-29 | 1.87E-26 |
| *ADAMTS5* | 3.57869112 | 0.766546824 | 4.668587757 | 3.03E-06 | 3.53E-05 |
| *SLC11A1* | 3.574920475 | 0.359346149 | 9.948403475 | 2.56E-23 | 1.04E-20 |
| *LIPN* | 3.568148224 | 0.342211255 | 10.4267413 | 1.87E-25 | 1.14E-22 |
| *FMO3* | 3.567383294 | 1.035668927 | 3.444520928 | 0.000572072 | 0.003015851 |
| *LCE5A* | 3.565652305 | 0.835277545 | 4.268823373 | 1.97E-05 | 0.000174003 |
| *CD177* | 3.554907087 | 0.572925008 | 6.204838396 | 5.48E-10 | 1.74E-08 |
| *STEAP4* | 3.554141944 | 0.528974866 | 6.718924043 | 1.83E-11 | 8.15E-10 |
| *ANXA3* | 3.551020709 | 0.443650355 | 8.00409752 | 1.20E-15 | 1.21E-13 |
| *PPEF1* | 3.529006621 | 0.43672551 | 8.080605647 | 6.44E-16 | 6.58E-14 |
| *LILRB2* | 3.522899264 | 0.316646274 | 11.12566152 | 9.41E-29 | 1.10E-25 |
| *TMEM38A* | 3.519196607 | 0.354778592 | 9.919416474 | 3.43E-23 | 1.34E-20 |
| *GFAP* | 3.513098769 | 0.580776065 | 6.048973056 | 1.46E-09 | 4.15E-08 |
| *ARHGAP24* | 3.506344796 | 0.343485149 | 10.2081409 | 1.82E-24 | 8.88E-22 |
| *CNR1* | 3.504641954 | 0.480564054 | 7.292767582 | 3.04E-13 | 1.87E-11 |
| *DNER* | 3.502836662 | 0.71004661 | 4.933248907 | 8.09E-07 | 1.12E-05 |
| *TRPM6* | 3.501692618 | 0.556963932 | 6.287108404 | 3.23E-10 | 1.10E-08 |
| *PENK* | 3.501527395 | 0.650735829 | 5.380873831 | 7.41E-08 | 1.36E-06 |
| *NLRC4* | 3.499931321 | 0.342429714 | 10.22087506 | 1.60E-24 | 8.03E-22 |
| *EDNRB* | 3.497341454 | 0.567345837 | 6.164390793 | 7.08E-10 | 2.17E-08 |
| *LILRB3* | 3.488772519 | 0.360813834 | 9.669176164 | 4.08E-22 | 1.28E-19 |
| *ZMYND15* | 3.485712665 | 0.383472627 | 9.08986046 | 9.92E-20 | 1.99E-17 |
| *CACNA1E* | 3.483588537 | 0.582470893 | 5.980708352 | 2.22E-09 | 6.12E-08 |
| *NPL* | 3.483264161 | 0.302666793 | 11.50857724 | 1.19E-30 | 1.99E-27 |
| *CPNE4* | 3.468823147 | 0.678551117 | 5.112102923 | 3.19E-07 | 4.94E-06 |
| *ROBO1* | 3.462247354 | 0.608070683 | 5.693823842 | 1.24E-08 | 2.79E-07 |
| *CXCL9* | 3.45770672 | 0.499216082 | 6.926272703 | 4.32E-12 | 2.17E-10 |
| *MRO* | 3.457374168 | 0.53481533 | 6.464613058 | 1.02E-10 | 3.84E-09 |
| *LORICRIN* | 3.445543372 | 1.031770039 | 3.339448949 | 0.000839448 | 0.004127511 |
| *CD5L* | 3.444544218 | 0.815221148 | 4.225288103 | 2.39E-05 | 0.000205103 |
| *ASGR2* | 3.426355185 | 0.431223258 | 7.945664162 | 1.93E-15 | 1.84E-13 |
| *NRP2* | 3.422874886 | 0.462811362 | 7.395831586 | 1.41E-13 | 9.17E-12 |
| *CYP27A1* | 3.421893867 | 0.53875169 | 6.35152322 | 2.13E-10 | 7.62E-09 |
| *IGSF6* | 3.419120959 | 0.394839585 | 8.659519177 | 4.74E-18 | 7.61E-16 |
| *LILRB1* | 3.412724711 | 0.355963965 | 9.587275818 | 9.04E-22 | 2.65E-19 |
| *HRK* | 3.410108513 | 0.538168407 | 6.336508176 | 2.35E-10 | 8.33E-09 |
| *CYB5R2* | 3.4057854 | 0.709773152 | 4.798413957 | 1.60E-06 | 2.03E-05 |
| *GLDN* | 3.404397192 | 0.543263863 | 6.266562938 | 3.69E-10 | 1.24E-08 |
| *PPP1R17* | 3.404240138 | 0.69643702 | 4.888080387 | 1.02E-06 | 1.37E-05 |
| *PKD2L1* | 3.403375142 | 0.388762052 | 8.75439133 | 2.05E-18 | 3.49E-16 |
| *CFI* | 3.402130385 | 0.711645375 | 4.78065411 | 1.75E-06 | 2.19E-05 |
| *MMP25* | 3.401910042 | 0.412150792 | 8.254042224 | 1.53E-16 | 1.78E-14 |
| *SLCO2B1* | 3.395191448 | 0.561607627 | 6.04548671 | 1.49E-09 | 4.23E-08 |
| *NAGS* | 3.390464442 | 0.404504478 | 8.381772324 | 5.21E-17 | 6.71E-15 |
| *INHBA* | 3.386738722 | 0.503288804 | 6.729215298 | 1.71E-11 | 7.64E-10 |
| *DCST1* | 3.375806302 | 0.509253527 | 6.628930628 | 3.38E-11 | 1.41E-09 |
| *GRIN3A* | 3.371928469 | 0.431100852 | 7.821669686 | 5.21E-15 | 4.56E-13 |
| *SERPINA1* | 3.37051333 | 0.393103287 | 8.574116367 | 9.99E-18 | 1.50E-15 |
| *CLEC4A* | 3.370120018 | 0.315610072 | 10.67811303 | 1.29E-26 | 1.02E-23 |
| *CPXM2* | 3.369755345 | 0.957136498 | 3.520663304 | 0.000430469 | 0.002391527 |
| *CEACAM6* | 3.365894915 | 0.534889496 | 6.292692112 | 3.12E-10 | 1.07E-08 |
| *MEFV* | 3.359785918 | 0.388255748 | 8.653538118 | 4.99E-18 | 7.98E-16 |
| *IL1RN* | 3.349715668 | 0.39095397 | 8.568056409 | 1.05E-17 | 1.56E-15 |
| *DGAT2* | 3.349324016 | 0.350534202 | 9.554913611 | 1.24E-21 | 3.56E-19 |
| *F5* | 3.349281482 | 0.393264979 | 8.516602447 | 1.64E-17 | 2.25E-15 |
| *CYP4F3* | 3.348370423 | 0.482082622 | 6.945636018 | 3.77E-12 | 1.92E-10 |
| *TMEM45B* | 3.342541457 | 0.551886682 | 6.056572059 | 1.39E-09 | 3.98E-08 |
| *KRT36* | 3.328082889 | 0.743785671 | 4.474518692 | 7.66E-06 | 7.81E-05 |
| *BEND7* | 3.325616296 | 0.692387927 | 4.803111329 | 1.56E-06 | 1.98E-05 |
| *TNNT1* | 3.323994395 | 0.561000276 | 5.925120785 | 3.12E-09 | 8.23E-08 |
| *CHRNA2* | 3.316425547 | 0.776165727 | 4.272831732 | 1.93E-05 | 0.000171431 |
| *SLC34A1* | 3.315635361 | 0.57290312 | 5.787427658 | 7.15E-09 | 1.71E-07 |
| *GPR84* | 3.31430438 | 0.424695882 | 7.803947526 | 6.00E-15 | 5.22E-13 |
| *BMP5* | 3.313528375 | 1.238088067 | 2.676326882 | 0.007443401 | 0.02496856 |
| *CKAP4* | 3.309684016 | 0.356822476 | 9.275435929 | 1.77E-20 | 4.24E-18 |
| *SLC37A2* | 3.304173867 | 0.411339662 | 8.032714022 | 9.53E-16 | 9.67E-14 |
| *CSF2* | 3.302583305 | 0.84890179 | 3.890418589 | 0.000100071 | 0.000697921 |
| *DRGX* | 3.287340722 | 0.902821802 | 3.64118447 | 0.000271387 | 0.001621252 |
| *MUC17* | 3.282047643 | 1.189888398 | 2.75828191 | 0.005810606 | 0.02036092 |
| *PCK1* | 3.278217578 | 1.035092697 | 3.167076328 | 0.001539799 | 0.006820187 |
| *VIPR1* | 3.275903691 | 0.298376142 | 10.97910733 | 4.82E-28 | 5.26E-25 |
| *ANKRD22* | 3.269459272 | 0.359462851 | 9.09540239 | 9.42E-20 | 1.90E-17 |
| *ACOD1* | 3.262170886 | 1.364481512 | 2.390776905 | 0.016812766 | 0.048283127 |
| *LPIN3* | 3.260091901 | 0.511392735 | 6.374928066 | 1.83E-10 | 6.61E-09 |
| *TENM1* | 3.258916472 | 0.411230895 | 7.92478511 | 2.29E-15 | 2.15E-13 |
| *ISL2* | 3.256376352 | 0.423902157 | 7.681905599 | 1.57E-14 | 1.24E-12 |
| *SCN5A* | 3.252952 | 0.527071072 | 6.171752105 | 6.75E-10 | 2.09E-08 |
| *PECR* | 3.244576012 | 0.390383866 | 8.311245147 | 9.47E-17 | 1.16E-14 |
| *TBC1D12* | 3.241072767 | 0.49207897 | 6.586489094 | 4.50E-11 | 1.84E-09 |
| *NLRP12* | 3.238147687 | 0.375227399 | 8.629827386 | 6.14E-18 | 9.63E-16 |
| *PNMA6E* | 3.232291102 | 0.723101984 | 4.470034897 | 7.82E-06 | 7.95E-05 |
| *SLC51A* | 3.222739745 | 0.469459941 | 6.864781128 | 6.66E-12 | 3.26E-10 |
| *GIMAP4* | 3.212875506 | 0.388067881 | 8.279158521 | 1.24E-16 | 1.47E-14 |
| *CLEC4D* | 3.210681459 | 0.472132552 | 6.800381478 | 1.04E-11 | 4.89E-10 |
| *CCL8* | 3.209899902 | 0.874017133 | 3.672582358 | 0.000240112 | 0.001462781 |
| *CXCL8* | 3.201865515 | 0.49306324 | 6.49382322 | 8.37E-11 | 3.23E-09 |
| *RPGRIP1* | 3.200562603 | 0.432672283 | 7.397198125 | 1.39E-13 | 9.10E-12 |
| *CRYBG2* | 3.199639024 | 0.380081753 | 8.418291589 | 3.82E-17 | 5.02E-15 |
| *TIMD4* | 3.198601274 | 0.503640686 | 6.350958852 | 2.14E-10 | 7.64E-09 |
| *SEMA6B* | 3.185499456 | 0.429248203 | 7.42111308 | 1.16E-13 | 7.73E-12 |
| *TMEM169* | 3.183271549 | 0.457442958 | 6.958838241 | 3.43E-12 | 1.76E-10 |
| *TXNDC2* | 3.177082569 | 0.411388013 | 7.722837 | 1.14E-14 | 9.19E-13 |
| *FPR2* | 3.174693001 | 0.454071371 | 6.991616739 | 2.72E-12 | 1.41E-10 |
| *ID4* | 3.16815958 | 1.136710993 | 2.787128479 | 0.005317739 | 0.018952753 |
| *MYCL* | 3.16782877 | 0.458679084 | 6.906416449 | 4.97E-12 | 2.48E-10 |
| *SHOC1* | 3.167812782 | 0.810037333 | 3.910699733 | 9.20E-05 | 0.000652092 |
| *ZNF703* | 3.162714753 | 0.455510033 | 6.943238396 | 3.83E-12 | 1.95E-10 |
| *IFI44L* | 3.162384299 | 0.38161689 | 8.286803818 | 1.16E-16 | 1.39E-14 |
| *SIRPB1* | 3.162045093 | 0.328428861 | 9.627793025 | 6.10E-22 | 1.84E-19 |
| *WNK2* | 3.162044699 | 0.441798298 | 7.157213403 | 8.23E-13 | 4.67E-11 |
| *TKTL2* | 3.158760497 | 0.48046299 | 6.574409608 | 4.88E-11 | 1.98E-09 |
| *IL18RAP* | 3.158365868 | 0.425560078 | 7.421668604 | 1.16E-13 | 7.72E-12 |
| *PID1* | 3.153023563 | 0.592708883 | 5.319683328 | 1.04E-07 | 1.85E-06 |
| *LGALS2* | 3.152312393 | 0.54490269 | 5.785092372 | 7.25E-09 | 1.73E-07 |
| *OVCH1* | 3.150953156 | 0.648909008 | 4.855770407 | 1.20E-06 | 1.58E-05 |
| *KCNK10* | 3.150204766 | 0.529007452 | 5.954934578 | 2.60E-09 | 7.03E-08 |
| *VWA2* | 3.145868147 | 0.685781905 | 4.587272026 | 4.49E-06 | 4.92E-05 |
| *PBX1* | 3.144593012 | 0.504197332 | 6.236829932 | 4.47E-10 | 1.45E-08 |
| *SOWAHC* | 3.143976577 | 0.397028125 | 7.918775477 | 2.40E-15 | 2.24E-13 |
| *PNPLA1* | 3.140876207 | 0.3643971 | 8.619377609 | 6.73E-18 | 1.04E-15 |
| *RNASE1* | 3.139271625 | 0.484307627 | 6.481978496 | 9.05E-11 | 3.47E-09 |
| *CRISPLD2* | 3.122804343 | 0.366137164 | 8.529055907 | 1.48E-17 | 2.09E-15 |
| *FGR* | 3.121392852 | 0.296207016 | 10.53787616 | 5.78E-26 | 4.07E-23 |
| *GASK1B* | 3.117365581 | 0.394728125 | 7.897500545 | 2.85E-15 | 2.60E-13 |
| *ECE1* | 3.114927971 | 0.28100485 | 11.08496159 | 1.48E-28 | 1.68E-25 |
| *CD200R1* | 3.114036345 | 0.356204606 | 8.742268613 | 2.28E-18 | 3.87E-16 |
| *MET* | 3.113963798 | 0.669981124 | 4.647838104 | 3.35E-06 | 3.85E-05 |
| *C5AR2* | 3.105780586 | 0.331698912 | 9.363252265 | 7.73E-21 | 1.99E-18 |
| *ISLR* | 3.103288678 | 0.78346587 | 3.960974939 | 7.46E-05 | 0.000546598 |
| *KCTD12* | 3.102864538 | 0.392699408 | 7.9013731 | 2.76E-15 | 2.54E-13 |
| *PLOD2* | 3.097302763 | 0.580171578 | 5.338597887 | 9.37E-08 | 1.69E-06 |
| *XCR1* | 3.094931952 | 0.50977817 | 6.071134734 | 1.27E-09 | 3.69E-08 |
| *ORM2* | 3.090634452 | 0.582750509 | 5.303529391 | 1.14E-07 | 2.00E-06 |
| *CES1* | 3.090158637 | 0.654850092 | 4.718879441 | 2.37E-06 | 2.85E-05 |
| *ICAM1* | 3.089339286 | 0.314094457 | 9.835701397 | 7.90E-23 | 2.84E-20 |
| *RIPK4* | 3.088498662 | 0.593187368 | 5.206615699 | 1.92E-07 | 3.18E-06 |
| *CXCL5* | 3.085967129 | 0.571085945 | 5.4036825 | 6.53E-08 | 1.21E-06 |
| *CCR2* | 3.085164457 | 0.492517075 | 6.264076138 | 3.75E-10 | 1.25E-08 |
| *KRT12* | 3.080758386 | 1.11805798 | 2.75545494 | 0.005861058 | 0.020501397 |
| *MPDZ* | 3.080733289 | 0.59762632 | 5.154949147 | 2.54E-07 | 4.07E-06 |
| *PTGES* | 3.07676173 | 0.499285772 | 6.162326072 | 7.17E-10 | 2.19E-08 |
| *C7* | 3.076622703 | 0.697792275 | 4.40908106 | 1.04E-05 | 0.000101364 |
| *GABRB3* | 3.076619631 | 1.047223089 | 2.937883688 | 0.00330461 | 0.012819942 |
| *BCL2A1* | 3.073667368 | 0.35743005 | 8.599353537 | 8.02E-18 | 1.23E-15 |
| *SIGLEC9* | 3.066142511 | 0.386068591 | 7.941963109 | 1.99E-15 | 1.89E-13 |
| *IGF2R* | 3.060209214 | 0.315501923 | 9.699494663 | 3.03E-22 | 9.79E-20 |
| *TLR5* | 3.059467729 | 0.456258212 | 6.705561994 | 2.01E-11 | 8.81E-10 |
| *SEMA6A* | 3.055031352 | 0.564188709 | 5.414910474 | 6.13E-08 | 1.15E-06 |
| *IL4I1* | 3.053780747 | 0.401028131 | 7.614879128 | 2.64E-14 | 1.98E-12 |
| *VPS37D* | 3.051832104 | 0.677682494 | 4.503336195 | 6.69E-06 | 6.95E-05 |
| *RBM47* | 3.051809028 | 0.358207196 | 8.519675369 | 1.60E-17 | 2.20E-15 |
| *EBI3* | 3.051456579 | 0.416087259 | 7.333693859 | 2.24E-13 | 1.41E-11 |
| *KCNJ2* | 3.045509701 | 0.423690249 | 7.18805709 | 6.57E-13 | 3.80E-11 |
| *COL15A1* | 3.044004805 | 0.50826614 | 5.988997825 | 2.11E-09 | 5.84E-08 |
| *MKX* | 3.043578633 | 0.930089117 | 3.272351626 | 0.001066568 | 0.005027291 |
| *FGL2* | 3.041308452 | 0.39701519 | 7.660433473 | 1.85E-14 | 1.44E-12 |
| *OXGR1* | 3.036034924 | 0.694843183 | 4.369381467 | 1.25E-05 | 0.000118164 |
| *TMEM40* | 3.032608601 | 0.587148423 | 5.16497785 | 2.40E-07 | 3.87E-06 |
| *TMEM108* | 3.032189522 | 0.710694777 | 4.266514433 | 1.99E-05 | 0.000175519 |
| *A2M* | 3.031985517 | 0.511514192 | 5.927470961 | 3.08E-09 | 8.14E-08 |
| *FPR3* | 3.031828638 | 0.416945215 | 7.271527598 | 3.55E-13 | 2.18E-11 |
| *ABLIM3* | 3.022508697 | 0.500668025 | 6.03695172 | 1.57E-09 | 4.44E-08 |
| *SCIMP* | 3.021026725 | 0.470848716 | 6.41613032 | 1.40E-10 | 5.16E-09 |
| *CTSS* | 3.019556804 | 0.252250806 | 11.97045452 | 5.07E-33 | 1.24E-29 |
| *GPR4* | 3.017586141 | 0.595116095 | 5.070583984 | 3.97E-07 | 5.98E-06 |
| *SGCE* | 3.016534225 | 0.507147839 | 5.948037223 | 2.71E-09 | 7.29E-08 |
| *FCER2* | 3.014046347 | 0.464486532 | 6.488985453 | 8.64E-11 | 3.32E-09 |
| *HCRTR1* | 3.010052649 | 0.788106085 | 3.819349588 | 0.000133804 | 0.000893796 |
| *GCM1* | 3.008516104 | 0.500224494 | 6.01433184 | 1.81E-09 | 5.05E-08 |
| *MPEG1* | 3.006361852 | 0.465555174 | 6.457584451 | 1.06E-10 | 4.01E-09 |
| *FCGR3A* | 3.00269498 | 0.338231396 | 8.877635294 | 6.83E-19 | 1.23E-16 |
| *LY6G6F* | 3.001140653 | 0.623204565 | 4.815658967 | 1.47E-06 | 1.88E-05 |
| *GIMAP8* | 2.993346564 | 0.41328551 | 7.242805492 | 4.39E-13 | 2.62E-11 |
| *NFIB* | 2.988251483 | 0.573266727 | 5.212672117 | 1.86E-07 | 3.10E-06 |
| *PKIB* | 2.984167226 | 0.409562251 | 7.286236015 | 3.19E-13 | 1.96E-11 |
| *SLC1A2* | 2.980797261 | 0.6687469 | 4.457287592 | 8.30E-06 | 8.36E-05 |
| *ADGRE3* | 2.977392137 | 0.399089251 | 7.460466873 | 8.62E-14 | 5.84E-12 |
| *ALDH1A2* | 2.974540741 | 0.401516508 | 7.408265115 | 1.28E-13 | 8.42E-12 |
| *NDNF* | 2.974501492 | 0.844946767 | 3.520341882 | 0.000430991 | 0.002393587 |
| *DOK6* | 2.974281874 | 0.775354998 | 3.836025925 | 0.000125041 | 0.000845611 |
| *ANKRD55* | 2.971361638 | 0.374599945 | 7.932093098 | 2.15E-15 | 2.03E-13 |
| *ADAP2* | 2.951611484 | 0.370893754 | 7.958105112 | 1.75E-15 | 1.67E-13 |
| *NEBL* | 2.950314674 | 0.561213685 | 5.257025541 | 1.46E-07 | 2.50E-06 |
| *KNDC1* | 2.943419597 | 0.498952896 | 5.899193334 | 3.65E-09 | 9.44E-08 |
| *LY96* | 2.940613755 | 0.288262833 | 10.20115469 | 1.96E-24 | 9.40E-22 |
| *PADI2* | 2.940401191 | 0.368570422 | 7.977854466 | 1.49E-15 | 1.45E-13 |
| *TMEM132C* | 2.93922565 | 0.818573543 | 3.590667784 | 0.000329832 | 0.00191265 |
| *CLEC4G* | 2.936902303 | 0.506052038 | 5.803557896 | 6.49E-09 | 1.57E-07 |
| *CPNE6* | 2.930752748 | 0.855053348 | 3.427567126 | 0.000609016 | 0.003175066 |
| *TCN1* | 2.922349245 | 0.489530588 | 5.969696924 | 2.38E-09 | 6.50E-08 |
| *CASP5* | 2.911712663 | 0.405142926 | 7.1868777 | 6.63E-13 | 3.83E-11 |
| *CARD14* | 2.906471476 | 0.4308103 | 6.746522718 | 1.51E-11 | 6.85E-10 |
| *VIP* | 2.903853503 | 1.005007849 | 2.889383903 | 0.003859975 | 0.014525841 |
| *SCIN* | 2.899029315 | 0.622462278 | 4.657357427 | 3.20E-06 | 3.70E-05 |
| *SMIM34A* | 2.897525065 | 0.530603198 | 5.460813419 | 4.74E-08 | 9.19E-07 |
| *ARSI* | 2.897049777 | 0.781117429 | 3.708853071 | 0.0002082 | 0.00129485 |
| *FLVCR2* | 2.891936856 | 0.309494748 | 9.344057942 | 9.27E-21 | 2.32E-18 |
| *CNTNAP3* | 2.891878133 | 0.528890134 | 5.467823933 | 4.56E-08 | 8.89E-07 |
| *SLPI* | 2.882237115 | 0.47245666 | 6.100532297 | 1.06E-09 | 3.12E-08 |
| *BAG3* | 2.878217582 | 0.364780991 | 7.890261983 | 3.02E-15 | 2.74E-13 |
| *RND3* | 2.875973445 | 0.892390166 | 3.222775817 | 0.001269549 | 0.005814118 |
| *ADORA2A* | 2.875838481 | 0.35068195 | 8.200702892 | 2.39E-16 | 2.62E-14 |
| *FCAR* | 2.875616377 | 0.475994208 | 6.041284394 | 1.53E-09 | 4.33E-08 |
| *RIMS1* | 2.874783902 | 1.035048563 | 2.777438669 | 0.005478918 | 0.0194104 |
| *CTLA4* | 2.870893744 | 0.396273354 | 7.24473073 | 4.33E-13 | 2.60E-11 |
| *NINJ1* | 2.867901562 | 0.325110017 | 8.821326356 | 1.13E-18 | 1.97E-16 |
| *CMTM5* | 2.867825489 | 0.543881887 | 5.272882874 | 1.34E-07 | 2.31E-06 |
| *CCR1* | 2.867707736 | 0.386787807 | 7.414162721 | 1.22E-13 | 8.11E-12 |
| *DNAJB5* | 2.867348766 | 0.394507142 | 7.268179613 | 3.64E-13 | 2.23E-11 |
| *SBK3* | 2.865088647 | 0.7270721 | 3.940583949 | 8.13E-05 | 0.000587871 |
| *NCF1* | 2.864247748 | 0.381122814 | 7.515288094 | 5.68E-14 | 3.98E-12 |
| *FLT1* | 2.859183668 | 0.49201612 | 5.811158521 | 6.20E-09 | 1.51E-07 |
| *CABP1* | 2.856713815 | 0.590340068 | 4.839098632 | 1.30E-06 | 1.70E-05 |
| *GNG8* | 2.853967224 | 0.641453702 | 4.449217796 | 8.62E-06 | 8.63E-05 |
| *EFCAB1* | 2.850738172 | 0.664166045 | 4.292207039 | 1.77E-05 | 0.00015914 |
| *ALPK2* | 2.850491172 | 0.500762163 | 5.692305413 | 1.25E-08 | 2.80E-07 |
| *C5orf47* | 2.845836534 | 0.616504493 | 4.61608401 | 3.91E-06 | 4.39E-05 |
| *ZBP1* | 2.845158271 | 0.325742153 | 8.734387739 | 2.45E-18 | 4.13E-16 |
| *CYBB* | 2.844877769 | 0.333912212 | 8.519837457 | 1.60E-17 | 2.20E-15 |
| *S1PR3* | 2.844527855 | 0.455723918 | 6.24177872 | 4.33E-10 | 1.41E-08 |
| *PPP4R4* | 2.840304062 | 0.457217045 | 6.212156988 | 5.23E-10 | 1.66E-08 |
| *LILRB5* | 2.839986872 | 0.397907338 | 7.137307102 | 9.52E-13 | 5.32E-11 |
| *ARHGAP6* | 2.836461194 | 0.48326964 | 5.869313854 | 4.38E-09 | 1.11E-07 |
| *THBD* | 2.833124081 | 0.452860352 | 6.256065624 | 3.95E-10 | 1.31E-08 |
| *BARX1* | 2.832124485 | 0.840869788 | 3.36808924 | 0.000756911 | 0.003788863 |
| *FCN2* | 2.831825946 | 0.40928572 | 6.918946368 | 4.55E-12 | 2.28E-10 |
| *PLAAT1* | 2.830568516 | 0.784517256 | 3.608038567 | 0.000308521 | 0.001804606 |
| *CD86* | 2.829464689 | 0.377655533 | 7.492183864 | 6.77E-14 | 4.69E-12 |
| *SPOCD1* | 2.829454315 | 0.732993851 | 3.860133767 | 0.000113325 | 0.000777507 |
| *TNFRSF1B* | 2.828898136 | 0.304907607 | 9.277886376 | 1.73E-20 | 4.18E-18 |
| *SEC14L5* | 2.828192169 | 0.529461076 | 5.341643221 | 9.21E-08 | 1.66E-06 |
| *C19orf38* | 2.826714524 | 0.271790858 | 10.40032968 | 2.47E-25 | 1.45E-22 |
| *CD83* | 2.823744408 | 0.359898559 | 7.845945296 | 4.30E-15 | 3.79E-13 |
| *ARSG* | 2.822270113 | 0.265294441 | 10.63825574 | 1.98E-26 | 1.49E-23 |
| *OIT3* | 2.8181867 | 0.497780373 | 5.661506259 | 1.50E-08 | 3.30E-07 |
| *LGMN* | 2.818148724 | 0.399743276 | 7.049896512 | 1.79E-12 | 9.61E-11 |
| *ACOX2* | 2.816269608 | 0.527271248 | 5.341215967 | 9.23E-08 | 1.67E-06 |
| *NEURL3* | 2.815943517 | 0.92896022 | 3.031285361 | 0.00243515 | 0.009977621 |
| *LRRC25* | 2.815120989 | 0.339293035 | 8.29701967 | 1.07E-16 | 1.29E-14 |
| *MCEMP1* | 2.810686903 | 0.395565518 | 7.105490182 | 1.20E-12 | 6.57E-11 |
| *PPBP* | 2.808581314 | 0.537109395 | 5.229067557 | 1.70E-07 | 2.86E-06 |
| *OR6K3* | 2.793880846 | 0.834986547 | 3.346018993 | 0.000819808 | 0.004045493 |
| *KLF5* | 2.790030747 | 0.396831902 | 7.030762227 | 2.05E-12 | 1.09E-10 |
| *OTOF* | 2.785772377 | 0.466070473 | 5.977148395 | 2.27E-09 | 6.24E-08 |
| *VIT* | 2.783301174 | 0.628157776 | 4.430895043 | 9.38E-06 | 9.28E-05 |
| *VNN2* | 2.780860276 | 0.39677574 | 7.008644916 | 2.41E-12 | 1.26E-10 |
| *ST6GALNAC3* | 2.776740569 | 0.43074639 | 6.446346701 | 1.15E-10 | 4.30E-09 |
| *PADI4* | 2.773802164 | 0.358543653 | 7.73630252 | 1.02E-14 | 8.37E-13 |
| *TINAGL1* | 2.773712121 | 0.59869181 | 4.632954846 | 3.60E-06 | 4.09E-05 |
| *KLHDC8A* | 2.7714903 | 0.66459508 | 4.170193829 | 3.04E-05 | 0.000251677 |
| *CRYM* | 2.770887387 | 0.549810802 | 5.039710713 | 4.66E-07 | 6.91E-06 |
| *KREMEN1* | 2.770770226 | 0.42823835 | 6.470159034 | 9.79E-11 | 3.72E-09 |
| *DGKI* | 2.76971432 | 0.58894768 | 4.702818971 | 2.57E-06 | 3.05E-05 |
| *SLC9A9* | 2.764900083 | 0.305563373 | 9.048532407 | 1.45E-19 | 2.85E-17 |
| *PPFIBP2* | 2.763623049 | 0.310416696 | 8.902945907 | 5.44E-19 | 1.00E-16 |
| *MEIS3* | 2.763128809 | 0.602319365 | 4.587481279 | 4.49E-06 | 4.92E-05 |
| *RAB31* | 2.762711664 | 0.327968751 | 8.423703946 | 3.65E-17 | 4.81E-15 |
| *CLEC1B* | 2.761240071 | 0.506929256 | 5.446992924 | 5.12E-08 | 9.83E-07 |
| *AFAP1* | 2.757829668 | 0.384826811 | 7.166417693 | 7.70E-13 | 4.38E-11 |
| *CD300LD* | 2.757630022 | 0.538159654 | 5.124185732 | 2.99E-07 | 4.68E-06 |
| *ODAD3* | 2.755126717 | 0.359920939 | 7.654810876 | 1.94E-14 | 1.48E-12 |
| *ZNF536* | 2.751130055 | 1.066616567 | 2.579305572 | 0.009899917 | 0.031626983 |
| *PKHD1L1* | 2.747887607 | 0.524068968 | 5.2433702 | 1.58E-07 | 2.67E-06 |
| *KLF4* | 2.74550989 | 0.331325491 | 8.286443292 | 1.17E-16 | 1.39E-14 |
| *ITGB3* | 2.744978069 | 0.517881688 | 5.30039608 | 1.16E-07 | 2.03E-06 |
| *GPRIN3* | 2.74415369 | 0.350397584 | 7.831542834 | 4.82E-15 | 4.23E-13 |
| *PTK2* | 2.743187376 | 0.390486132 | 7.025057106 | 2.14E-12 | 1.14E-10 |
| *UPB1* | 2.742614623 | 0.400780805 | 6.843178591 | 7.75E-12 | 3.73E-10 |
| *NBPF4* | 2.735277701 | 1.089344589 | 2.510938897 | 0.012041054 | 0.036955719 |
| *HS3ST2* | 2.734811985 | 0.913435196 | 2.993985779 | 0.002753588 | 0.011025496 |
| *LYNX1* | 2.734103492 | 0.40568348 | 6.739499208 | 1.59E-11 | 7.15E-10 |
| *PDE4A* | 2.730834528 | 0.369152165 | 7.397585018 | 1.39E-13 | 9.09E-12 |
| *ACSM5* | 2.72945558 | 0.632730815 | 4.31377059 | 1.60E-05 | 0.000146626 |
| *BEX1* | 2.721255697 | 0.560239173 | 4.857310639 | 1.19E-06 | 1.57E-05 |
| *UPK3A* | 2.720700535 | 0.554157118 | 4.909619401 | 9.13E-07 | 1.25E-05 |
| *EDN1* | 2.719108723 | 0.359143318 | 7.571096504 | 3.70E-14 | 2.67E-12 |
| *CDH5* | 2.71568328 | 0.502945471 | 5.399558073 | 6.68E-08 | 1.24E-06 |
| *PCDH12* | 2.715213589 | 0.460053719 | 5.901949007 | 3.59E-09 | 9.30E-08 |
| *B4GALNT3* | 2.714063722 | 0.40462613 | 6.707583915 | 1.98E-11 | 8.70E-10 |
| *BCL2L15* | 2.713324251 | 0.343031842 | 7.909832039 | 2.58E-15 | 2.39E-13 |
| *LBP* | 2.706248253 | 0.488581121 | 5.538994724 | 3.04E-08 | 6.21E-07 |
| *PLIN4* | 2.706235645 | 0.438219659 | 6.175523144 | 6.59E-10 | 2.04E-08 |
| *SDC2* | 2.702784036 | 0.454135607 | 5.951491122 | 2.66E-09 | 7.16E-08 |
| *SULF1* | 2.702277256 | 0.925799645 | 2.918857521 | 0.003513168 | 0.013465654 |
| *MS4A6E* | 2.697172173 | 0.540153775 | 4.993341342 | 5.93E-07 | 8.54E-06 |
| *LILRA1* | 2.695749999 | 0.362094297 | 7.444883897 | 9.70E-14 | 6.55E-12 |
| *DOCK9* | 2.693857768 | 0.34470842 | 7.8148882 | 5.50E-15 | 4.80E-13 |
| *GPR65* | 2.692601332 | 0.299609467 | 8.987036875 | 2.54E-19 | 4.81E-17 |
| *MS4A4A* | 2.689750823 | 0.443574243 | 6.063812005 | 1.33E-09 | 3.82E-08 |
| *ITGAM* | 2.685801405 | 0.292925595 | 9.168886053 | 4.78E-20 | 1.04E-17 |
| *CXCL12* | 2.685600415 | 0.764657223 | 3.51216249 | 0.000444476 | 0.002459849 |
| *MXRA5* | 2.685551119 | 1.112718287 | 2.413504973 | 0.015799912 | 0.045970774 |
| *FABP3* | 2.684170265 | 0.596626855 | 4.498909568 | 6.83E-06 | 7.08E-05 |
| *SNX25* | 2.682046966 | 0.358480645 | 7.481706493 | 7.34E-14 | 5.06E-12 |
| *CD101* | 2.678409677 | 0.314081756 | 8.52774676 | 1.49E-17 | 2.09E-15 |
| *PHF24* | 2.675716235 | 0.583098306 | 4.588790961 | 4.46E-06 | 4.90E-05 |
| *NPHS1* | 2.674661001 | 0.547074798 | 4.889022507 | 1.01E-06 | 1.37E-05 |
| *TUBA4B* | 2.67152654 | 0.458677659 | 5.82440956 | 5.73E-09 | 1.41E-07 |
| *ICOS* | 2.666059513 | 0.396369506 | 6.726197326 | 1.74E-11 | 7.79E-10 |
| *TNF* | 2.665883308 | 0.395690519 | 6.737293867 | 1.61E-11 | 7.24E-10 |
| *ENTPD2* | 2.663456107 | 0.541708665 | 4.916768514 | 8.80E-07 | 1.21E-05 |
| *SLC22A31* | 2.66326694 | 0.519738202 | 5.124247035 | 2.99E-07 | 4.68E-06 |
| *CLEC10A* | 2.660148092 | 0.579594587 | 4.589670353 | 4.44E-06 | 4.89E-05 |
| *ZFP36L1* | 2.658715717 | 0.325417088 | 8.170178578 | 3.08E-16 | 3.33E-14 |
| *CLMP* | 2.658391318 | 0.807453263 | 3.292316027 | 0.000993659 | 0.004740809 |
| *EXT1* | 2.656501831 | 0.269320427 | 9.863722053 | 5.98E-23 | 2.28E-20 |
| *BCL6* | 2.655775975 | 0.29494914 | 9.004182803 | 2.17E-19 | 4.14E-17 |
| *TRAPPC3L* | 2.652118111 | 0.728815118 | 3.638944977 | 0.000273757 | 0.001632949 |
| *DMRTA1* | 2.651360523 | 0.767408791 | 3.454951982 | 0.000550391 | 0.002924332 |
| *ABCA8* | 2.650062908 | 0.854735233 | 3.100448896 | 0.001932276 | 0.00824146 |
| *PAPSS2* | 2.649640577 | 0.429740395 | 6.165677247 | 7.02E-10 | 2.15E-08 |
| *HMSD* | 2.647802029 | 0.92065347 | 2.876002877 | 0.00402746 | 0.015049798 |
| *HPCAL4* | 2.644780099 | 0.341266761 | 7.749890713 | 9.20E-15 | 7.60E-13 |
| *ART3* | 2.642250885 | 0.781330734 | 3.381731667 | 0.000720305 | 0.003632043 |
| *NCKAP5* | 2.634537438 | 0.75561076 | 3.486633038 | 0.000489142 | 0.002660736 |
| *DCN* | 2.634293046 | 0.826072084 | 3.188938468 | 0.001427963 | 0.006426441 |
| *LHX4* | 2.634279159 | 0.512930903 | 5.135738838 | 2.81E-07 | 4.44E-06 |
| *IL24* | 2.631234962 | 0.694587629 | 3.788197272 | 0.000151744 | 0.00099495 |
| *RETN* | 2.630069176 | 0.474531227 | 5.542457543 | 2.98E-08 | 6.12E-07 |
| *JAML* | 2.628930234 | 0.371269542 | 7.080920831 | 1.43E-12 | 7.75E-11 |
| *UGCG* | 2.627534814 | 0.328573581 | 7.99679271 | 1.28E-15 | 1.27E-13 |
| *ZP1* | 2.626660116 | 0.776002393 | 3.384860843 | 0.000712143 | 0.003598917 |
| *SPIC* | 2.626577061 | 0.863882997 | 3.040431484 | 0.002362394 | 0.009722303 |
| *MEGF9* | 2.625215624 | 0.244480351 | 10.73794116 | 6.75E-27 | 6.11E-24 |
| *CDHR5* | 2.624169756 | 0.425229792 | 6.171180399 | 6.78E-10 | 2.09E-08 |
| *RUNX1T1* | 2.62321205 | 0.882699743 | 2.97180561 | 0.002960541 | 0.011723652 |
| *PF4* | 2.622890379 | 0.572202066 | 4.583853385 | 4.56E-06 | 4.99E-05 |
| *DEFB124* | 2.619931617 | 0.705090885 | 3.715736043 | 0.000202613 | 0.001266071 |
| *ATRNL1* | 2.619811748 | 0.859698176 | 3.047362226 | 0.002308593 | 0.009541824 |
| *CDH10* | 2.617664375 | 0.788956619 | 3.317881253 | 0.00090703 | 0.00440856 |
| *CYSTM1* | 2.615365399 | 0.319847434 | 8.176915372 | 2.91E-16 | 3.16E-14 |
| *LLCFC1* | 2.614000878 | 0.580408316 | 4.503727477 | 6.68E-06 | 6.95E-05 |
| *MGP* | 2.612222496 | 0.469477647 | 5.564104096 | 2.64E-08 | 5.48E-07 |
| *NCF2* | 2.607616811 | 0.305782032 | 8.527697953 | 1.49E-17 | 2.09E-15 |
| *PI4K2A* | 2.606014624 | 0.305617431 | 8.527048397 | 1.50E-17 | 2.09E-15 |
| *RGL4* | 2.601203164 | 0.389612638 | 6.676382932 | 2.45E-11 | 1.05E-09 |
| *GCKR* | 2.600629835 | 0.569695835 | 4.564944438 | 5.00E-06 | 5.41E-05 |
| *TDRP* | 2.598359616 | 0.676207458 | 3.842548 | 0.000121764 | 0.000826447 |
| *SNAP91* | 2.597818379 | 0.999493648 | 2.599134456 | 0.009345916 | 0.030167667 |
| *STX11* | 2.597388025 | 0.283826465 | 9.15132429 | 5.62E-20 | 1.19E-17 |
| *DNASE1L3* | 2.597278228 | 0.555689615 | 4.67397295 | 2.95E-06 | 3.45E-05 |
| *BEND2* | 2.595609961 | 0.77902092 | 3.331887365 | 0.000862592 | 0.004222008 |
| *CCDC80* | 2.590150078 | 0.677379761 | 3.823778373 | 0.000131422 | 0.000879927 |
| *MS4A6A* | 2.589917483 | 0.431719726 | 5.999071456 | 1.98E-09 | 5.53E-08 |
| *CMTM2* | 2.589002755 | 0.399500864 | 6.480593636 | 9.14E-11 | 3.49E-09 |
| *TLR7* | 2.588764975 | 0.475586332 | 5.443312391 | 5.23E-08 | 1.00E-06 |
| *NUAK2* | 2.588700105 | 0.346578711 | 7.469299245 | 8.06E-14 | 5.51E-12 |
| *KRT7* | 2.58600846 | 0.458184665 | 5.644031016 | 1.66E-08 | 3.61E-07 |
| *BMP2* | 2.58490077 | 0.491015705 | 5.2643953 | 1.41E-07 | 2.41E-06 |
| *RAB11FIP5* | 2.581755917 | 0.444830371 | 5.803911079 | 6.48E-09 | 1.57E-07 |
| *LGALSL* | 2.580764162 | 0.368260065 | 7.007993563 | 2.42E-12 | 1.26E-10 |
| *HPSE* | 2.578473949 | 0.357733434 | 7.207808118 | 5.69E-13 | 3.35E-11 |
| *CDH1* | 2.575754572 | 0.529768446 | 4.86203848 | 1.16E-06 | 1.53E-05 |
| *CD1C* | 2.572008972 | 0.545363624 | 4.716135909 | 2.40E-06 | 2.89E-05 |
| *FFAR2* | 2.569411826 | 0.440021973 | 5.839280726 | 5.24E-09 | 1.31E-07 |
| *GPR20* | 2.56629591 | 0.811987893 | 3.160510068 | 0.001574932 | 0.006955362 |
| *LRCH2* | 2.566208808 | 0.836610694 | 3.067387051 | 0.002159391 | 0.009032461 |
| *CEBPB* | 2.564940258 | 0.284279294 | 9.02260668 | 1.84E-19 | 3.55E-17 |
| *DACT3* | 2.564461739 | 0.45828091 | 5.59582929 | 2.20E-08 | 4.66E-07 |
| *ACVR2A* | 2.56425921 | 0.342152373 | 7.494494885 | 6.66E-14 | 4.62E-12 |
| *MTSS1* | 2.562755516 | 0.331383093 | 7.733513173 | 1.05E-14 | 8.51E-13 |
| *IL10RA* | 2.559801329 | 0.306480554 | 8.352247123 | 6.70E-17 | 8.35E-15 |
| *CAMK1* | 2.559726281 | 0.433582372 | 5.903667789 | 3.56E-09 | 9.23E-08 |
| *LIMCH1* | 2.556908909 | 0.508206899 | 5.031236127 | 4.87E-07 | 7.20E-06 |
| *VDR* | 2.555017122 | 0.34782504 | 7.345696346 | 2.05E-13 | 1.29E-11 |
| *CNTNAP3B* | 2.552058395 | 0.644646756 | 3.958847803 | 7.53E-05 | 0.000550772 |
| *CPLX1* | 2.547476667 | 0.520652692 | 4.892852192 | 9.94E-07 | 1.35E-05 |
| *WNT5B* | 2.54249643 | 0.41074461 | 6.189969059 | 6.02E-10 | 1.88E-08 |
| *SEC14L2* | 2.541716785 | 0.451162954 | 5.633700118 | 1.76E-08 | 3.82E-07 |
| *RFPL4A* | 2.541695075 | 1.05056391 | 2.419362639 | 0.015547732 | 0.045374665 |
| *FXYD6* | 2.539922872 | 0.35710557 | 7.112526614 | 1.14E-12 | 6.27E-11 |
| *RCN3* | 2.539477127 | 0.366242689 | 6.933864362 | 4.09E-12 | 2.07E-10 |
| *ANXA5* | 2.534128273 | 0.341898556 | 7.411930309 | 1.24E-13 | 8.23E-12 |
| *ARHGAP8* | 2.532711336 | 0.5282288 | 4.794724061 | 1.63E-06 | 2.06E-05 |
| *THEM5* | 2.53055968 | 0.790883501 | 3.199661742 | 0.00137589 | 0.006228394 |
| *PALMD* | 2.529436605 | 0.888851461 | 2.845736004 | 0.004430891 | 0.016264412 |
| *SIGLEC7* | 2.527564454 | 0.402218582 | 6.284056894 | 3.30E-10 | 1.12E-08 |
| *ALOX5AP* | 2.526523276 | 0.304762469 | 8.29013915 | 1.13E-16 | 1.36E-14 |
| *MME* | 2.526176225 | 0.628039951 | 4.022317723 | 5.76E-05 | 0.000437372 |
| *MARCHF1* | 2.522906202 | 0.394041669 | 6.402638098 | 1.53E-10 | 5.60E-09 |
| *ARHGEF10L* | 2.521268933 | 0.530407558 | 4.753455892 | 2.00E-06 | 2.46E-05 |
| *PRELP* | 2.52103966 | 1.004965216 | 2.508583999 | 0.012121614 | 0.037149949 |
| *ZSWIM2* | 2.520002558 | 0.611849298 | 4.118665437 | 3.81E-05 | 0.000305941 |
| *CD93* | 2.518821718 | 0.356159215 | 7.072178984 | 1.53E-12 | 8.24E-11 |
| *SYNDIG1* | 2.518045972 | 0.985086409 | 2.55616761 | 0.010583213 | 0.033322278 |
| *TAAR1* | 2.516642638 | 0.641845705 | 3.920946448 | 8.82E-05 | 0.000629209 |
| *RBM11* | 2.516374708 | 0.489346479 | 5.142316977 | 2.71E-07 | 4.31E-06 |
| *FGD2* | 2.514714626 | 0.423008491 | 5.944832501 | 2.77E-09 | 7.42E-08 |
| *SUSD4* | 2.514490338 | 0.426316963 | 5.898170979 | 3.68E-09 | 9.48E-08 |
| *SULF2* | 2.510226604 | 0.352612181 | 7.118944669 | 1.09E-12 | 6.04E-11 |
| *CXCR1* | 2.509046694 | 0.501314384 | 5.004936569 | 5.59E-07 | 8.13E-06 |
| *SIGLEC14* | 2.508634265 | 0.325630492 | 7.703929227 | 1.32E-14 | 1.05E-12 |
| *THY1* | 2.508461914 | 0.687562631 | 3.64833951 | 0.000263941 | 0.001583104 |
| *CDC42EP5* | 2.50528339 | 0.710454307 | 3.526311779 | 0.000421391 | 0.00235058 |
| *APLNR* | 2.504383619 | 0.747198738 | 3.351696802 | 0.000803179 | 0.003980812 |
| *FAM9C* | 2.500818364 | 0.562661742 | 4.444621302 | 8.80E-06 | 8.79E-05 |
| *CALD1* | 2.498844641 | 0.472989326 | 5.283088859 | 1.27E-07 | 2.20E-06 |
| *CC2D2B* | 2.493040045 | 0.438405155 | 5.686612062 | 1.30E-08 | 2.89E-07 |
| *PEAK3* | 2.491738996 | 0.381236669 | 6.535937381 | 6.32E-11 | 2.49E-09 |
| *PDGFB* | 2.491311126 | 0.36873992 | 6.756282664 | 1.42E-11 | 6.48E-10 |
| *IL15* | 2.48737648 | 0.39458192 | 6.303827802 | 2.90E-10 | 1.00E-08 |
| *SGSH* | 2.482154641 | 0.325928035 | 7.615652462 | 2.62E-14 | 1.98E-12 |
| *TUSC3* | 2.471258766 | 0.854078024 | 2.893481272 | 0.00380997 | 0.014368408 |
| *FRZB* | 2.470577488 | 0.90639292 | 2.725724609 | 0.006416051 | 0.022112847 |
| *ABCA13* | 2.46913679 | 0.497963008 | 4.958474323 | 7.10E-07 | 1.00E-05 |
| *CR1* | 2.468183227 | 0.398752323 | 6.18976513 | 6.03E-10 | 1.88E-08 |
| *HTRA1* | 2.465486026 | 0.502893033 | 4.902605252 | 9.46E-07 | 1.29E-05 |
| *NHS* | 2.463912946 | 0.352523162 | 6.989364704 | 2.76E-12 | 1.43E-10 |
| *GRHL1* | 2.462339123 | 0.381406585 | 6.455942878 | 1.08E-10 | 4.05E-09 |
| *PFKFB2* | 2.460603154 | 0.326410173 | 7.538377656 | 4.76E-14 | 3.39E-12 |
| *INSYN2A* | 2.460528485 | 0.748330963 | 3.288021752 | 0.00100894 | 0.004800694 |
| *SUSD3* | 2.458561229 | 0.308595653 | 7.966934094 | 1.63E-15 | 1.57E-13 |
| *CPM* | 2.458518311 | 0.401016982 | 6.13070872 | 8.75E-10 | 2.63E-08 |
| *SOCS3* | 2.457567943 | 0.373441562 | 6.580863504 | 4.68E-11 | 1.90E-09 |
| *FFAR4* | 2.456564192 | 0.581706322 | 4.22303162 | 2.41E-05 | 0.000206895 |
| *FCER1G* | 2.455822224 | 0.264945842 | 9.269147999 | 1.88E-20 | 4.47E-18 |
| *CD207* | 2.453342721 | 0.855440906 | 2.867927761 | 0.004131699 | 0.015353099 |
| *HS3ST4* | 2.452860405 | 0.868424871 | 2.824493503 | 0.004735541 | 0.017187274 |
| *AP3B2* | 2.452687619 | 0.508268893 | 4.825570976 | 1.40E-06 | 1.80E-05 |
| *PDE5A* | 2.451114183 | 0.385791818 | 6.353463364 | 2.11E-10 | 7.55E-09 |
| *MGAM* | 2.448406408 | 0.425958095 | 5.747998305 | 9.03E-09 | 2.10E-07 |
| *ST14* | 2.447944907 | 0.372846078 | 6.565564314 | 5.18E-11 | 2.08E-09 |
| *CDH20* | 2.447084579 | 0.660920212 | 3.702541597 | 0.00021345 | 0.001320497 |
| *TYMP* | 2.446712135 | 0.324896977 | 7.530732221 | 5.05E-14 | 3.57E-12 |
| *HLA-DQB1* | 2.444404196 | 0.51902258 | 4.709629774 | 2.48E-06 | 2.96E-05 |
| *RNF222* | 2.44358618 | 0.551149425 | 4.43361831 | 9.27E-06 | 9.18E-05 |
| *SNX21* | 2.443381866 | 0.308701742 | 7.915024547 | 2.47E-15 | 2.29E-13 |
| *CLCF1* | 2.440987129 | 0.339821774 | 7.183139262 | 6.81E-13 | 3.93E-11 |
| *PLD2* | 2.440803202 | 0.304399554 | 8.018419124 | 1.07E-15 | 1.08E-13 |
| *MAP2* | 2.43670916 | 0.446440266 | 5.458085543 | 4.81E-08 | 9.31E-07 |
| *BTNL3* | 2.43652987 | 0.671909022 | 3.626279436 | 0.000287534 | 0.001700071 |
| *COL3A1* | 2.428902399 | 0.821959717 | 2.955013914 | 0.00312655 | 0.012255324 |
| *CHST8* | 2.428693476 | 0.724685816 | 3.35137438 | 0.000804115 | 0.003984826 |
| *MAN1C1* | 2.428437891 | 0.319129966 | 7.609557699 | 2.75E-14 | 2.04E-12 |
| *FCGR3B* | 2.427939869 | 0.54073433 | 4.490079015 | 7.12E-06 | 7.34E-05 |
| *OLFM3* | 2.423121348 | 0.952477551 | 2.54401938 | 0.0109585 | 0.03424755 |
| *VMO1* | 2.421521961 | 0.339296036 | 7.136900235 | 9.55E-13 | 5.33E-11 |
| *C4BPB* | 2.419720225 | 0.506415781 | 4.77812958 | 1.77E-06 | 2.22E-05 |
| *TMEM89* | 2.419610524 | 0.440597786 | 5.491653837 | 3.98E-08 | 7.89E-07 |
| *KCNE1* | 2.41640567 | 0.430719444 | 5.610161565 | 2.02E-08 | 4.33E-07 |
| *SMOC2* | 2.411984684 | 0.842146469 | 2.864091668 | 0.00418207 | 0.015496617 |
| *IPCEF1* | 2.411112824 | 0.286205278 | 8.424417751 | 3.63E-17 | 4.80E-15 |
| *HSPA6* | 2.409734578 | 0.38547972 | 6.25126162 | 4.07E-10 | 1.35E-08 |
| *GOLGA7B* | 2.409353311 | 0.385640962 | 6.247659216 | 4.17E-10 | 1.37E-08 |
| *ARHGEF4* | 2.409188873 | 0.424448056 | 5.676051148 | 1.38E-08 | 3.05E-07 |
| *TMEM158* | 2.406365643 | 0.377991546 | 6.366189055 | 1.94E-10 | 6.97E-09 |
| *CD36* | 2.404295577 | 0.389383942 | 6.1746141 | 6.63E-10 | 2.05E-08 |
| *KLK1* | 2.404265651 | 0.481446593 | 4.99383667 | 5.92E-07 | 8.52E-06 |
| *LYZ* | 2.403217147 | 0.342937768 | 7.007735421 | 2.42E-12 | 1.27E-10 |
| *RBM24* | 2.399206554 | 0.744222985 | 3.223773793 | 0.001265133 | 0.005799167 |
| *IGSF11* | 2.399192949 | 0.583931266 | 4.108690679 | 3.98E-05 | 0.000317045 |
| *TMEM119* | 2.398741502 | 0.337381554 | 7.109877445 | 1.16E-12 | 6.38E-11 |
| *FGD4* | 2.392251608 | 0.330876247 | 7.230049393 | 4.83E-13 | 2.87E-11 |
| *NIPAL4* | 2.388305274 | 0.501159877 | 4.765555634 | 1.88E-06 | 2.33E-05 |
| *ACSM6* | 2.387260912 | 0.764711336 | 3.121780469 | 0.001797609 | 0.007767557 |
| *IFIT1* | 2.383751484 | 0.378822131 | 6.292534911 | 3.12E-10 | 1.07E-08 |
| *C19orf33* | 2.376772748 | 0.648053911 | 3.667554051 | 0.000244882 | 0.001487649 |
| *TSPAN9* | 2.375776787 | 0.411940424 | 5.767282475 | 8.06E-09 | 1.89E-07 |
| *CXCL10* | 2.373179254 | 0.455663859 | 5.208179688 | 1.91E-07 | 3.16E-06 |
| *AOC3* | 2.372311873 | 0.316515315 | 7.495093478 | 6.63E-14 | 4.61E-12 |
| *ABI3* | 2.371401437 | 0.319421064 | 7.424060925 | 1.14E-13 | 7.60E-12 |
| *TNC* | 2.368835069 | 0.844865241 | 2.803802255 | 0.005050387 | 0.018153179 |
| *SNTB2* | 2.365814929 | 0.28247831 | 8.375209151 | 5.51E-17 | 6.99E-15 |
| *NRGN* | 2.365751422 | 0.37905878 | 6.241120242 | 4.34E-10 | 1.42E-08 |
| *LGALS3* | 2.365289177 | 0.37469525 | 6.312567818 | 2.74E-10 | 9.57E-09 |
| *VENTX* | 2.364648894 | 0.474641675 | 4.981966439 | 6.29E-07 | 8.98E-06 |
| *GLB1L3* | 2.363939368 | 0.500595108 | 4.722258224 | 2.33E-06 | 2.81E-05 |
| *ASTL* | 2.363195513 | 0.460266472 | 5.13440726 | 2.83E-07 | 4.47E-06 |
| *PROZ* | 2.362177276 | 0.746701532 | 3.163482562 | 0.001558937 | 0.006894343 |
| *TUBA4A* | 2.361620647 | 0.306309891 | 7.709906601 | 1.26E-14 | 1.01E-12 |
| *NFIA* | 2.359654424 | 0.446938704 | 5.279592938 | 1.29E-07 | 2.24E-06 |
| *PAPPA* | 2.358847378 | 0.819922449 | 2.876915227 | 0.004015835 | 0.015014204 |
| *SIGLEC5* | 2.356113295 | 0.304529468 | 7.736897554 | 1.02E-14 | 8.35E-13 |
| *CLEC4F* | 2.356094252 | 0.728763439 | 3.233002817 | 0.001224964 | 0.005633001 |
| *ZNF385A* | 2.354575913 | 0.282942394 | 8.321750156 | 8.67E-17 | 1.07E-14 |
| *CAMK2D* | 2.352972163 | 0.364255549 | 6.459674166 | 1.05E-10 | 3.96E-09 |
| *RGS2* | 2.35297185 | 0.243074758 | 9.680033725 | 3.67E-22 | 1.16E-19 |
| *STAC* | 2.352496485 | 0.64122123 | 3.668775106 | 0.000243715 | 0.001481547 |
| *KRT23* | 2.352186992 | 0.527160413 | 4.46199474 | 8.12E-06 | 8.22E-05 |
| *PLIN5* | 2.35174949 | 0.397124031 | 5.921952105 | 3.18E-09 | 8.36E-08 |
| *POTEG* | 2.351062773 | 0.46565342 | 5.048954158 | 4.44E-07 | 6.62E-06 |
| *CPS1* | 2.350961288 | 0.491089276 | 4.787238091 | 1.69E-06 | 2.13E-05 |
| *DUSP8* | 2.34863459 | 0.465501252 | 5.045388339 | 4.53E-07 | 6.73E-06 |
| *LAMB3* | 2.348489003 | 0.515499968 | 4.555750043 | 5.22E-06 | 5.61E-05 |
| *TIMP3* | 2.346110164 | 0.452216118 | 5.188028618 | 2.13E-07 | 3.47E-06 |
| *GPNMB* | 2.343654857 | 0.476769043 | 4.915702671 | 8.85E-07 | 1.22E-05 |
| *FXYD7* | 2.342628828 | 0.413884953 | 5.660096628 | 1.51E-08 | 3.33E-07 |
| *FREM1* | 2.341637405 | 0.608078622 | 3.850879343 | 0.000117694 | 0.000802096 |
| *CHRD* | 2.339964179 | 0.559087021 | 4.185330888 | 2.85E-05 | 0.000237588 |
| *SATB2* | 2.339012574 | 0.400836779 | 5.835324245 | 5.37E-09 | 1.33E-07 |
| *SOD2* | 2.3378606 | 0.259222913 | 9.018726658 | 1.90E-19 | 3.65E-17 |
| *RNF144B* | 2.335984567 | 0.292210826 | 7.994175286 | 1.30E-15 | 1.29E-13 |
| *PGAP4* | 2.335282866 | 0.692772822 | 3.370921596 | 0.000749172 | 0.003757848 |
| *MAOB* | 2.333095751 | 0.532550466 | 4.380985281 | 1.18E-05 | 0.000113092 |
| *SLC22A3* | 2.330838359 | 0.770960594 | 3.023291175 | 0.002500415 | 0.010200148 |
| *RNF182* | 2.330514179 | 0.701650477 | 3.321474517 | 0.000895432 | 0.004361561 |
| *ESM1* | 2.329933414 | 0.574737494 | 4.053908854 | 5.04E-05 | 0.000389752 |
| *TMCC3* | 2.329890849 | 0.394223993 | 5.91006862 | 3.42E-09 | 8.92E-08 |
| *RAB3IL1* | 2.321762484 | 0.505513989 | 4.592874845 | 4.37E-06 | 4.83E-05 |
| *PCDH17* | 2.31869672 | 0.761514496 | 3.044849089 | 0.002327971 | 0.009609368 |
| *KCND3* | 2.311480229 | 0.619655562 | 3.730266252 | 0.000191278 | 0.001207394 |
| *IFI27* | 2.311381779 | 0.423266985 | 5.460812816 | 4.74E-08 | 9.19E-07 |
| *CUEDC1* | 2.311227158 | 0.302398256 | 7.64299103 | 2.12E-14 | 1.62E-12 |
| *TIAM1* | 2.31081623 | 0.368090149 | 6.277854039 | 3.43E-10 | 1.16E-08 |
| *FMN1* | 2.30996837 | 0.412006632 | 5.606629094 | 2.06E-08 | 4.42E-07 |
| *CEBPD* | 2.308115599 | 0.307743508 | 7.500127679 | 6.38E-14 | 4.45E-12 |
| *TREM1* | 2.305516837 | 0.370911423 | 6.215815131 | 5.11E-10 | 1.63E-08 |
| *ETV7* | 2.305133973 | 0.413473369 | 5.575048231 | 2.47E-08 | 5.18E-07 |
| *C4BPA* | 2.303551278 | 0.726980407 | 3.16865662 | 0.001531452 | 0.006790818 |
| *GCA* | 2.302168288 | 0.245904166 | 9.362054829 | 7.82E-21 | 2.00E-18 |
| *SH2D4A* | 2.301540579 | 0.516149371 | 4.459059161 | 8.23E-06 | 8.31E-05 |
| *MB21D2* | 2.301289939 | 0.419738735 | 5.482672307 | 4.19E-08 | 8.26E-07 |
| *GPAT2* | 2.299367444 | 0.626970179 | 3.66742713 | 0.000245003 | 0.001488071 |
| *TMEM63C* | 2.298117981 | 0.428469257 | 5.363553958 | 8.16E-08 | 1.49E-06 |
| *GRK5* | 2.297869887 | 0.250923254 | 9.157660175 | 5.30E-20 | 1.13E-17 |
| *LRRC4* | 2.297552989 | 0.364587174 | 6.301793234 | 2.94E-10 | 1.01E-08 |
| *WFS1* | 2.296789489 | 0.408774633 | 5.618718247 | 1.92E-08 | 4.14E-07 |
| *PROS1* | 2.296472244 | 0.395018676 | 5.813578915 | 6.12E-09 | 1.50E-07 |
| *CDS1* | 2.296060199 | 0.495991966 | 4.629228609 | 3.67E-06 | 4.16E-05 |
| *PRLR* | 2.295806637 | 0.602707429 | 3.809156028 | 0.000139442 | 0.000927348 |
| *PRKG1* | 2.291580991 | 0.737456608 | 3.107411292 | 0.001887336 | 0.008084643 |
| *PSAP* | 2.286588669 | 0.232816547 | 9.821418167 | 9.11E-23 | 3.17E-20 |
| *RUBCNL* | 2.286007386 | 0.301152295 | 7.590868228 | 3.18E-14 | 2.32E-12 |
| *TICAM2* | 2.285877869 | 0.363179921 | 6.294064564 | 3.09E-10 | 1.06E-08 |
| *TNFRSF8* | 2.28558671 | 0.356914321 | 6.403740554 | 1.52E-10 | 5.57E-09 |
| *DUSP4* | 2.284083972 | 0.490425973 | 4.657347079 | 3.20E-06 | 3.70E-05 |
| *RAI2* | 2.2825532 | 0.460056255 | 4.961465422 | 7.00E-07 | 9.87E-06 |
| *RAB36* | 2.282095747 | 0.320630451 | 7.117526557 | 1.10E-12 | 6.07E-11 |
| *ATP1B2* | 2.276126897 | 0.542099711 | 4.198723687 | 2.68E-05 | 0.000226231 |
| *SLC22A14* | 2.275792789 | 0.392769213 | 5.794223972 | 6.86E-09 | 1.65E-07 |
| *HAS1* | 2.275592399 | 0.81571652 | 2.789685316 | 0.005275929 | 0.018831302 |
| *KIR3DL2* | 2.275169596 | 0.567217755 | 4.011104337 | 6.04E-05 | 0.000455077 |
| *TDRD1* | 2.275067758 | 0.67671661 | 3.361920962 | 0.000774023 | 0.003859872 |
| *CMKLR1* | 2.274547654 | 0.349302382 | 6.511686631 | 7.43E-11 | 2.89E-09 |
| *PCSK5* | 2.273777565 | 0.380387339 | 5.977532189 | 2.27E-09 | 6.23E-08 |
| *PTAFR* | 2.269442063 | 0.329201048 | 6.893787478 | 5.43E-12 | 2.70E-10 |
| *CD300LB* | 2.267187829 | 0.418988184 | 5.411102071 | 6.26E-08 | 1.17E-06 |
| *SNX7* | 2.266425106 | 0.700500713 | 3.235435829 | 0.001214572 | 0.005596577 |
| *GBP1* | 2.264189592 | 0.370828919 | 6.105752478 | 1.02E-09 | 3.02E-08 |
| *DES* | 2.26365116 | 0.590848254 | 3.831188708 | 0.000127526 | 0.000859328 |
| *MTMR11* | 2.259217649 | 0.494527662 | 4.568435347 | 4.91E-06 | 5.33E-05 |
| *SPP1* | 2.259163282 | 0.564196494 | 4.004213614 | 6.22E-05 | 0.000466107 |
| *EXD1* | 2.258872881 | 0.418462875 | 5.398024575 | 6.74E-08 | 1.25E-06 |
| *CYP4F12* | 2.257145534 | 0.462140316 | 4.884113015 | 1.04E-06 | 1.40E-05 |
| *DYSF* | 2.256903498 | 0.365229321 | 6.179414865 | 6.43E-10 | 2.01E-08 |
| *ZNF483* | 2.256231516 | 0.389968017 | 5.785683483 | 7.22E-09 | 1.73E-07 |
| *IRAG1* | 2.253182116 | 0.311852697 | 7.225148717 | 5.01E-13 | 2.97E-11 |
| *RNF212B* | 2.252163413 | 0.768798358 | 2.929459187 | 0.003395524 | 0.013109978 |
| *NOS3* | 2.251955244 | 0.377978339 | 5.957894973 | 2.56E-09 | 6.91E-08 |
| *IQSEC1* | 2.251169838 | 0.222441185 | 10.12029242 | 4.49E-24 | 2.03E-21 |
| *CLC* | 2.250565488 | 0.498197108 | 4.517419818 | 6.26E-06 | 6.57E-05 |
| *PCSK6* | 2.249562162 | 0.489492476 | 4.595703248 | 4.31E-06 | 4.77E-05 |
| *CTSH* | 2.249528663 | 0.379563297 | 5.926623262 | 3.09E-09 | 8.16E-08 |
| *SLC5A9* | 2.248163287 | 0.536840958 | 4.187764089 | 2.82E-05 | 0.000235739 |
| *TNNI2* | 2.247669722 | 0.319634966 | 7.031989499 | 2.04E-12 | 1.09E-10 |
| *CYP24A1* | 2.242879501 | 0.717918195 | 3.124143553 | 0.001783234 | 0.00771492 |
| *PTPN3* | 2.242878225 | 0.677078532 | 3.312582098 | 0.00092439 | 0.004475755 |
| *AXIN2* | 2.2410989 | 0.348966963 | 6.422094752 | 1.34E-10 | 4.98E-09 |
| *PTGFR* | 2.238999208 | 0.744906202 | 3.005746497 | 0.002649298 | 0.010687673 |
| *CCL19* | 2.238964469 | 0.646470785 | 3.463365276 | 0.000533464 | 0.00284922 |
| *SLC37A3* | 2.237643889 | 0.299814283 | 7.46343325 | 8.43E-14 | 5.74E-12 |
| *SLC8A1* | 2.237567895 | 0.435938609 | 5.132759173 | 2.86E-07 | 4.50E-06 |
| *CD48* | 2.234985023 | 0.283047919 | 7.896136561 | 2.88E-15 | 2.62E-13 |
| *GPRC5A* | 2.234204399 | 0.729452071 | 3.06285291 | 0.002192378 | 0.009135437 |
| *SPHK1* | 2.231541765 | 0.334993264 | 6.661452648 | 2.71E-11 | 1.15E-09 |
| *HES5* | 2.231007466 | 0.701186055 | 3.181762458 | 0.001463818 | 0.006557044 |
| *RAB39A* | 2.230996169 | 0.444753103 | 5.016257682 | 5.27E-07 | 7.70E-06 |
| *NLRP6* | 2.225896055 | 0.35097125 | 6.342103669 | 2.27E-10 | 8.06E-09 |
| *HLA-DRB5* | 2.224938855 | 0.386667808 | 5.754135223 | 8.71E-09 | 2.03E-07 |
| *ALDH2* | 2.223937045 | 0.29967804 | 7.4210878 | 1.16E-13 | 7.73E-12 |
| *HLA-DQA1* | 2.223891255 | 0.462938611 | 4.803857799 | 1.56E-06 | 1.98E-05 |
| *RNASE4* | 2.223861537 | 0.558101927 | 3.984687079 | 6.76E-05 | 0.000500695 |
| *TPST1* | 2.223411431 | 0.437275729 | 5.08468978 | 3.68E-07 | 5.61E-06 |
| *FBXO17* | 2.22265291 | 0.681704331 | 3.260435368 | 0.001112413 | 0.005203096 |
| *CEMIP* | 2.221013751 | 0.712714825 | 3.116272701 | 0.001831528 | 0.007885061 |
| *ADA2* | 2.219556479 | 0.291341525 | 7.618400713 | 2.57E-14 | 1.94E-12 |
| *APOD* | 2.219375042 | 0.650231344 | 3.413208336 | 0.000642028 | 0.003311208 |
| *GIMAP7* | 2.218737419 | 0.435822369 | 5.090921383 | 3.56E-07 | 5.45E-06 |
| *LYPD3* | 2.217811405 | 0.320105774 | 6.928370502 | 4.26E-12 | 2.15E-10 |
| *BGN* | 2.216754992 | 0.64695333 | 3.426452712 | 0.00061152 | 0.003186549 |
| *COL8A2* | 2.216002885 | 0.435063404 | 5.093517096 | 3.51E-07 | 5.38E-06 |
| *WNT7A* | 2.215704512 | 0.407403277 | 5.438602574 | 5.37E-08 | 1.03E-06 |
| *PKDCC* | 2.213321491 | 0.549130639 | 4.030591871 | 5.56E-05 | 0.000424087 |
| *PMM1* | 2.211912013 | 0.247982198 | 8.919640313 | 4.68E-19 | 8.76E-17 |
| *PHLDA2* | 2.211055075 | 0.412863657 | 5.355412225 | 8.54E-08 | 1.55E-06 |
| *DHRS9* | 2.207032204 | 0.42014173 | 5.253065926 | 1.50E-07 | 2.54E-06 |
| *UBXN10* | 2.204189583 | 0.370837569 | 5.943814133 | 2.78E-09 | 7.46E-08 |
| *OR52K2* | 2.204129997 | 0.465824862 | 4.73167101 | 2.23E-06 | 2.70E-05 |
| *KIAA0513* | 2.20375596 | 0.291678039 | 7.555440113 | 4.17E-14 | 3.00E-12 |
| *PILRA* | 2.202584871 | 0.283012077 | 7.782653275 | 7.10E-15 | 6.03E-13 |
| *TNFRSF10C* | 2.200962976 | 0.377859163 | 5.824823614 | 5.72E-09 | 1.41E-07 |
| *CNN3* | 2.199750854 | 0.442489214 | 4.971309544 | 6.65E-07 | 9.42E-06 |
| *S100P* | 2.196860529 | 0.483447321 | 4.544157006 | 5.52E-06 | 5.88E-05 |
| *BASP1* | 2.195406428 | 0.388989593 | 5.643869316 | 1.66E-08 | 3.61E-07 |
| *EGR2* | 2.19342045 | 0.419618707 | 5.227175088 | 1.72E-07 | 2.89E-06 |
| *HAMP* | 2.19294406 | 0.384434012 | 5.704344549 | 1.17E-08 | 2.65E-07 |
| *CDHR2* | 2.191501507 | 0.312903724 | 7.003756558 | 2.49E-12 | 1.30E-10 |
| *COTL1* | 2.19076831 | 0.230401751 | 9.508470769 | 1.93E-21 | 5.37E-19 |
| *NFAM1* | 2.189473856 | 0.352937507 | 6.203573751 | 5.52E-10 | 1.75E-08 |
| *LILRA2* | 2.188893075 | 0.254774591 | 8.59148891 | 8.58E-18 | 1.31E-15 |
| *ANG* | 2.187247794 | 0.335425519 | 6.52081512 | 6.99E-11 | 2.73E-09 |
| *EGR3* | 2.186040772 | 0.457109088 | 4.782317455 | 1.73E-06 | 2.18E-05 |
| *SLCO4C1* | 2.184709412 | 0.319125379 | 6.845928141 | 7.60E-12 | 3.66E-10 |
| *COBL* | 2.184048049 | 0.70560078 | 3.095302768 | 0.001966121 | 0.008367777 |
| *CCM2L* | 2.178418929 | 0.49560122 | 4.395507598 | 1.11E-05 | 0.000106986 |
| *PRSS33* | 2.178044696 | 0.592130122 | 3.678321055 | 0.000234774 | 0.001436138 |
| *PFKFB4* | 2.174591292 | 0.225059211 | 9.662307439 | 4.36E-22 | 1.35E-19 |
| *EVC* | 2.173855144 | 0.601844586 | 3.611987539 | 0.000303859 | 0.00178228 |
| *EMCN* | 2.172212132 | 0.663635188 | 3.273202161 | 0.001063364 | 0.00501368 |
| *IQCA1* | 2.171315728 | 0.637263489 | 3.407249538 | 0.000656211 | 0.003367323 |
| *ZNF366* | 2.171166848 | 0.501997251 | 4.325057249 | 1.52E-05 | 0.000140451 |
| *BST1* | 2.171080346 | 0.285142382 | 7.614021923 | 2.66E-14 | 1.98E-12 |
| *SHISA4* | 2.170894651 | 0.366148647 | 5.928998156 | 3.05E-09 | 8.09E-08 |
| *CCDC158* | 2.169785739 | 0.794749914 | 2.730149071 | 0.006330569 | 0.021863463 |
| *KDR* | 2.169093969 | 0.68446721 | 3.169025393 | 0.00152951 | 0.006785059 |
| *PTPRN* | 2.167887679 | 0.490294291 | 4.421604978 | 9.80E-06 | 9.64E-05 |
| *FAM181B* | 2.166240841 | 0.727036757 | 2.979547899 | 0.002886741 | 0.011478793 |
| *CCR5* | 2.163220939 | 0.449268811 | 4.814981337 | 1.47E-06 | 1.88E-05 |
| *PHOSPHO1* | 2.162439124 | 0.485352631 | 4.455397965 | 8.37E-06 | 8.42E-05 |
| *H2AW* | 2.162081108 | 0.537258814 | 4.024282245 | 5.71E-05 | 0.000434258 |
| *GIPC2* | 2.160777269 | 0.534085772 | 4.045749546 | 5.22E-05 | 0.000400837 |
| *CR1L* | 2.15672547 | 0.426487887 | 5.056944252 | 4.26E-07 | 6.39E-06 |
| *ALKAL2* | 2.156444977 | 0.6411574 | 3.363362846 | 0.000769991 | 0.003842792 |
| *HCK* | 2.1520307 | 0.246929949 | 8.715146584 | 2.90E-18 | 4.84E-16 |
| *DEFA6* | 2.145870015 | 0.885395235 | 2.423629504 | 0.015366273 | 0.044940387 |
| *TMC1* | 2.144089263 | 0.688664939 | 3.113399769 | 0.001849453 | 0.007953578 |
| *GLTPD2* | 2.143807797 | 0.62461549 | 3.432204025 | 0.000598697 | 0.003130033 |
| *MYOF* | 2.142160155 | 0.439150873 | 4.877959462 | 1.07E-06 | 1.43E-05 |
| *IDO1* | 2.141638741 | 0.705544998 | 3.035438911 | 0.002401859 | 0.009860361 |
| *HBZ* | 2.140037508 | 0.623225615 | 3.433808649 | 0.000595164 | 0.00311362 |
| *SCML1* | 2.138021224 | 0.392068999 | 5.453175916 | 4.95E-08 | 9.54E-07 |
| *SH3D19* | 2.137457645 | 0.397560752 | 5.376430229 | 7.60E-08 | 1.39E-06 |
| *TREML1* | 2.135829428 | 0.494255117 | 4.321309697 | 1.55E-05 | 0.000142444 |
| *ADAMTSL5* | 2.133287 | 0.365183074 | 5.841691884 | 5.17E-09 | 1.29E-07 |
| *SPINK8* | 2.132264139 | 0.496532638 | 4.294308122 | 1.75E-05 | 0.00015791 |
| *ARNT2* | 2.129026182 | 0.665448737 | 3.199384211 | 0.001377215 | 0.00623083 |
| *PRTN3* | 2.128618245 | 0.484989616 | 4.388997564 | 1.14E-05 | 0.000109569 |
| *GUCY2C* | 2.127882392 | 0.604021917 | 3.52285626 | 0.000426923 | 0.002374212 |
| *FJX1* | 2.126242656 | 0.593342919 | 3.583497144 | 0.000339024 | 0.001955755 |
| *ACHE* | 2.122131135 | 0.578822705 | 3.666288687 | 0.000246096 | 0.001492703 |
| *CX3CR1* | 2.119805449 | 0.55382359 | 3.827582439 | 0.000129408 | 0.000869569 |
| *RIN2* | 2.119566419 | 0.345014935 | 6.143404834 | 8.08E-10 | 2.44E-08 |
| *CATIP* | 2.116751343 | 0.283295823 | 7.471876287 | 7.91E-14 | 5.43E-12 |
| *NRN1* | 2.115555514 | 0.524541384 | 4.033152729 | 5.50E-05 | 0.000419894 |
| *SLC43A2* | 2.112619641 | 0.31540309 | 6.698157719 | 2.11E-11 | 9.19E-10 |
| *TRIM40* | 2.112324976 | 0.650758234 | 3.245944293 | 0.001170617 | 0.005433587 |
| *NOD2* | 2.109254788 | 0.323870195 | 6.512654816 | 7.38E-11 | 2.87E-09 |
| *CLEC4C* | 2.109078223 | 0.587168953 | 3.591944384 | 0.00032822 | 0.001904698 |
| *DBH* | 2.10753133 | 0.831164938 | 2.535635507 | 0.011224349 | 0.034927554 |
| *IL18R1* | 2.106164832 | 0.40660949 | 5.179822129 | 2.22E-07 | 3.60E-06 |
| *COL1A1* | 2.10613876 | 0.47626984 | 4.422154381 | 9.77E-06 | 9.63E-05 |
| *APCDD1* | 2.105326448 | 0.502570767 | 4.18911442 | 2.80E-05 | 0.000234465 |
| *EFS* | 2.102100594 | 0.801944173 | 2.621255526 | 0.008760657 | 0.028567423 |
| *DMPK* | 2.101739111 | 0.296486321 | 7.088823203 | 1.35E-12 | 7.33E-11 |
| *DKK2* | 2.100933002 | 0.687225494 | 3.057123203 | 0.002234724 | 0.009272766 |
| *IDO2* | 2.097628986 | 0.634859921 | 3.304081604 | 0.000952881 | 0.004587009 |
| *GALR2* | 2.095558155 | 0.420438506 | 4.984220342 | 6.22E-07 | 8.88E-06 |
| *NOS1AP* | 2.09393065 | 0.475157616 | 4.406812773 | 1.05E-05 | 0.0001023 |
| *TMEM26* | 2.093544681 | 0.84420903 | 2.479888994 | 0.013142329 | 0.039588312 |
| *MYEOV* | 2.092451272 | 0.544447098 | 3.843259114 | 0.000121411 | 0.000824408 |
| *MANSC1* | 2.08836664 | 0.366867092 | 5.692433819 | 1.25E-08 | 2.80E-07 |
| *RRAS* | 2.088353564 | 0.297482803 | 7.020081639 | 2.22E-12 | 1.17E-10 |
| *KLHL29* | 2.088327401 | 0.411094954 | 5.079914948 | 3.78E-07 | 5.73E-06 |
| *CCR4* | 2.085763616 | 0.350065717 | 5.958205881 | 2.55E-09 | 6.91E-08 |
| *MMRN2* | 2.082969007 | 0.466866298 | 4.461596432 | 8.14E-06 | 8.23E-05 |
| *PELI3* | 2.082959816 | 0.285294163 | 7.301095113 | 2.85E-13 | 1.78E-11 |
| *SEMA3C* | 2.080950809 | 0.510371177 | 4.077328233 | 4.56E-05 | 0.000357139 |
| *NEIL1* | 2.080187222 | 0.314731517 | 6.609402326 | 3.86E-11 | 1.60E-09 |
| *LAMP3* | 2.077815524 | 0.357844089 | 5.806482734 | 6.38E-09 | 1.55E-07 |
| *CHST2* | 2.076032897 | 0.288051556 | 7.207157365 | 5.71E-13 | 3.35E-11 |
| *OPLAH* | 2.075518588 | 0.359105905 | 5.779683812 | 7.48E-09 | 1.78E-07 |
| *PIK3R5* | 2.075517856 | 0.23141266 | 8.968903676 | 2.99E-19 | 5.64E-17 |
| *THEMIS2* | 2.073312414 | 0.218887848 | 9.472030694 | 2.74E-21 | 7.43E-19 |
| *MX1* | 2.073109145 | 0.311426309 | 6.656820838 | 2.80E-11 | 1.18E-09 |
| *LTBP2* | 2.072953179 | 0.272256189 | 7.613980002 | 2.66E-14 | 1.98E-12 |
| *GPER1* | 2.072565676 | 0.577495076 | 3.588888913 | 0.00033209 | 0.001923984 |
| *TMEM151A* | 2.071990306 | 0.531099586 | 3.901321636 | 9.57E-05 | 0.000671954 |
| *IL1RL2* | 2.070800062 | 0.615798698 | 3.362787337 | 0.000771598 | 0.003849598 |
| *INSL4* | 2.068792906 | 0.582326464 | 3.552634193 | 0.000381394 | 0.002161372 |
| *FAM3B* | 2.06875985 | 0.847654705 | 2.440569063 | 0.014664142 | 0.043290877 |
| *SYN1* | 2.068292927 | 0.448704709 | 4.609474532 | 4.04E-06 | 4.51E-05 |
| *C3* | 2.066487511 | 0.348812835 | 5.924344819 | 3.14E-09 | 8.26E-08 |
| *CAMSAP2* | 2.065734942 | 0.340325489 | 6.069880175 | 1.28E-09 | 3.71E-08 |
| *EPSTI1* | 2.065249844 | 0.33114861 | 6.236625433 | 4.47E-10 | 1.45E-08 |
| *MS4A7* | 2.064797366 | 0.328271161 | 6.289913973 | 3.18E-10 | 1.08E-08 |
| *HES1* | 2.064407709 | 0.453599066 | 4.551172754 | 5.33E-06 | 5.71E-05 |
| *DUSP6* | 2.06423037 | 0.246921519 | 8.359864225 | 6.28E-17 | 7.89E-15 |
| *CDC42EP3* | 2.063661329 | 0.277286306 | 7.442348519 | 9.89E-14 | 6.66E-12 |
| *LRIG1* | 2.060304573 | 0.333968992 | 6.169149302 | 6.87E-10 | 2.12E-08 |
| *PYY* | 2.059006096 | 0.567770512 | 3.62647593 | 0.000287316 | 0.001699414 |
| *PLEKHS1* | 2.058382723 | 0.43246711 | 4.759628369 | 1.94E-06 | 2.39E-05 |
| *FKBP6* | 2.058086988 | 0.47838042 | 4.302197372 | 1.69E-05 | 0.000153305 |
| *DAPK2* | 2.057388306 | 0.380927047 | 5.40100348 | 6.63E-08 | 1.23E-06 |
| *SPOCK1* | 2.057154646 | 0.474145922 | 4.338653039 | 1.43E-05 | 0.000133554 |
| *SLC6A19* | 2.056779248 | 0.705552618 | 2.915132331 | 0.003555378 | 0.01359211 |
| *XKR7* | 2.055788627 | 0.360482763 | 5.702876358 | 1.18E-08 | 2.67E-07 |
| *SYNE1* | 2.054530911 | 0.2646298 | 7.763792717 | 8.24E-15 | 6.87E-13 |
| *TNFAIP6* | 2.054153477 | 0.469681574 | 4.373502372 | 1.22E-05 | 0.000116276 |
| *CH25H* | 2.050880122 | 0.616472662 | 3.32679817 | 0.0008785 | 0.004287003 |
| *TNFAIP3* | 2.048329089 | 0.343605835 | 5.961275632 | 2.50E-09 | 6.80E-08 |
| *KCNMA1* | 2.047501305 | 0.534429105 | 3.831193486 | 0.000127523 | 0.000859328 |
| *RASGEF1B* | 2.046021171 | 0.33343061 | 6.136272761 | 8.45E-10 | 2.54E-08 |
| *TLCD4* | 2.044316389 | 0.604929661 | 3.379428253 | 0.000726368 | 0.003658535 |
| *NEFM* | 2.041002757 | 0.638065339 | 3.198736291 | 0.001380314 | 0.006239499 |
| *OAS1* | 2.040843422 | 0.309163376 | 6.601181058 | 4.08E-11 | 1.68E-09 |
| *CXCL6* | 2.039754216 | 0.483410147 | 4.219510554 | 2.45E-05 | 0.000209585 |
| *HNMT* | 2.038886715 | 0.470355813 | 4.334775202 | 1.46E-05 | 0.000135412 |
| *KCNJ3* | 2.038041733 | 0.714888848 | 2.850851205 | 0.004360237 | 0.016053461 |
| *TSPAN18* | 2.037666809 | 0.297911051 | 6.839849676 | 7.93E-12 | 3.80E-10 |
| *NRP1* | 2.036781763 | 0.512804838 | 3.971845842 | 7.13E-05 | 0.000525643 |
| *IFIT2* | 2.036568992 | 0.324091682 | 6.283928605 | 3.30E-10 | 1.12E-08 |
| *HORMAD1* | 2.035763085 | 0.379433617 | 5.365268103 | 8.08E-08 | 1.47E-06 |
| *TNFRSF9* | 2.033561123 | 0.334329483 | 6.082506101 | 1.18E-09 | 3.46E-08 |
| *CACNA1I* | 2.029819368 | 0.369831281 | 5.488501026 | 4.05E-08 | 8.01E-07 |
| *ITPRIP* | 2.029726689 | 0.300575002 | 6.752812706 | 1.45E-11 | 6.59E-10 |
| *MNDA* | 2.026800871 | 0.336891125 | 6.016189574 | 1.79E-09 | 4.99E-08 |
| *DLEU7* | 2.026691435 | 0.43084278 | 4.70401624 | 2.55E-06 | 3.03E-05 |
| *FAM178B* | 2.026461858 | 0.673952958 | 3.006829829 | 0.002639875 | 0.010653733 |
| *GEM* | 2.025959544 | 0.484200036 | 4.184137532 | 2.86E-05 | 0.000238588 |
| *FZD9* | 2.025361637 | 0.486585379 | 4.162397239 | 3.15E-05 | 0.000259079 |
| *ADAM23* | 2.020148011 | 0.435117228 | 4.642767236 | 3.44E-06 | 3.93E-05 |
| *FNDC5* | 2.018456758 | 0.790152656 | 2.554514931 | 0.010633587 | 0.03343765 |
| *ZNF467* | 2.018300724 | 0.246479487 | 8.188513965 | 2.64E-16 | 2.88E-14 |
| *SLC2A6* | 2.016705705 | 0.334170752 | 6.034955756 | 1.59E-09 | 4.49E-08 |
| *PAQR5* | 2.016392362 | 0.435531042 | 4.629732826 | 3.66E-06 | 4.15E-05 |
| *HLA-F* | 2.016188738 | 0.262654476 | 7.676201705 | 1.64E-14 | 1.29E-12 |
| *LYPD2* | 2.014485211 | 0.68465571 | 2.94233318 | 0.003257492 | 0.012679107 |
| *NCMAP* | 2.014111039 | 0.795735269 | 2.531132046 | 0.011369504 | 0.035299432 |
| *ERICH2* | 2.01401776 | 0.77480397 | 2.599390089 | 0.009338958 | 0.030148282 |
| *BCL3* | 2.01309626 | 0.258988732 | 7.772910605 | 7.67E-15 | 6.46E-13 |
| *LY6K* | 2.008042968 | 0.609033451 | 3.29709799 | 0.000976894 | 0.004679867 |
| *CES4A* | 2.007782986 | 0.402198887 | 4.992015274 | 5.98E-07 | 8.59E-06 |
| *FLOT1* | 2.005867845 | 0.242922638 | 8.257228986 | 1.49E-16 | 1.74E-14 |
| *TMEM252* | 2.005764182 | 0.600766976 | 3.338672498 | 0.000841797 | 0.004135952 |
| *GPR15* | 2.004003376 | 0.435759819 | 4.598871414 | 4.25E-06 | 4.70E-05 |
| *BLVRA* | 2.003295376 | 0.259527726 | 7.719003321 | 1.17E-14 | 9.44E-13 |
| *CD68* | 2.002295363 | 0.309451911 | 6.470457257 | 9.77E-11 | 3.72E-09 |
| *OSBPL6* | 2.001914112 | 0.512309794 | 3.907624129 | 9.32E-05 | 0.000658616 |
| *SLC26A11* | 2.001849188 | 0.377766366 | 5.299172632 | 1.16E-07 | 2.04E-06 |
| *AGXT2* | 2.000701547 | 0.729648799 | 2.742006221 | 0.006106519 | 0.021228635 |
| *MCOLN2* | 1.998281097 | 0.41652851 | 4.797465353 | 1.61E-06 | 2.03E-05 |
| *STUM* | 1.996924338 | 0.611853767 | 3.263728107 | 0.001099566 | 0.005152905 |
| *EDAR* | 1.99657321 | 0.406938194 | 4.906330347 | 9.28E-07 | 1.27E-05 |
| *FBP1* | 1.995840226 | 0.434707851 | 4.59122195 | 4.41E-06 | 4.86E-05 |
| *PRG2* | 1.995309024 | 0.454289441 | 4.392153645 | 1.12E-05 | 0.000108352 |
| *MGAM2* | 1.993919996 | 0.39454876 | 5.05367194 | 4.33E-07 | 6.48E-06 |
| *MAPK13* | 1.993265691 | 0.232574873 | 8.570425799 | 1.03E-17 | 1.53E-15 |
| *KIAA0319* | 1.993063568 | 0.412623826 | 4.830219295 | 1.36E-06 | 1.76E-05 |
| *PELI1* | 1.992721144 | 0.341326064 | 5.838174564 | 5.28E-09 | 1.32E-07 |
| *LDLRAD3* | 1.992645385 | 0.45456707 | 4.383611389 | 1.17E-05 | 0.000111805 |
| *TPBGL* | 1.992210336 | 0.490381792 | 4.062569954 | 4.85E-05 | 0.000377317 |
| *CDKN2D* | 1.987922487 | 0.255214147 | 7.789233109 | 6.74E-15 | 5.79E-13 |
| *OR52K1* | 1.985618771 | 0.575343666 | 3.451187329 | 0.000558126 | 0.002956994 |
| *ACSL1* | 1.98358334 | 0.301755355 | 6.573481824 | 4.92E-11 | 1.98E-09 |
| *CHST1* | 1.980984941 | 0.605812915 | 3.269961554 | 0.001075621 | 0.005058663 |
| *TGM2* | 1.980434528 | 0.424843479 | 4.661562736 | 3.14E-06 | 3.65E-05 |
| *DOC2B* | 1.980234192 | 0.55306716 | 3.580458823 | 0.000342991 | 0.001974852 |
| *OTOA* | 1.979980369 | 0.443902737 | 4.460392341 | 8.18E-06 | 8.27E-05 |
| *SLC24A4* | 1.979945167 | 0.325527993 | 6.082257778 | 1.19E-09 | 3.46E-08 |
| *C1orf226* | 1.979473842 | 0.477049617 | 4.149408723 | 3.33E-05 | 0.000272101 |
| *ELAPOR1* | 1.977785687 | 0.422120074 | 4.685362788 | 2.79E-06 | 3.29E-05 |
| *GPR155* | 1.977538828 | 0.244932243 | 8.073819925 | 6.81E-16 | 6.94E-14 |
| *FCGRT* | 1.976071617 | 0.319056014 | 6.19349435 | 5.88E-10 | 1.84E-08 |
| *SH3BP5* | 1.974760316 | 0.32206776 | 6.131505722 | 8.71E-10 | 2.61E-08 |
| *LINGO2* | 1.974470095 | 0.538725387 | 3.665077126 | 0.000247264 | 0.001498639 |
| *HIF1A* | 1.973282897 | 0.303780922 | 6.495743335 | 8.26E-11 | 3.19E-09 |
| *PLCL1* | 1.972385203 | 0.416419859 | 4.736530115 | 2.17E-06 | 2.64E-05 |
| *BCL2L14* | 1.972275786 | 0.60406597 | 3.265000654 | 0.001094638 | 0.005135132 |
| *ZBTB32* | 1.970904154 | 0.369730143 | 5.330655868 | 9.79E-08 | 1.75E-06 |
| *NFKB2* | 1.96707718 | 0.248488824 | 7.916159555 | 2.45E-15 | 2.28E-13 |
| *CHRDL1* | 1.966732051 | 0.667858575 | 2.944833122 | 0.003231289 | 0.012598803 |
| *TLR4* | 1.965471029 | 0.322545717 | 6.093619985 | 1.10E-09 | 3.24E-08 |
| *KLF9* | 1.965025915 | 0.380381999 | 5.165927733 | 2.39E-07 | 3.85E-06 |
| *SPTBN5* | 1.963412412 | 0.329002449 | 5.967774459 | 2.41E-09 | 6.56E-08 |
| *NIBAN2* | 1.962239316 | 0.295054509 | 6.650429865 | 2.92E-11 | 1.23E-09 |
| *CALHM5* | 1.961238911 | 0.537971273 | 3.645620147 | 0.000266748 | 0.001597459 |
| *PRSS41* | 1.958893478 | 0.589866902 | 3.320907602 | 0.000897252 | 0.004369085 |
| *RLN3* | 1.958286679 | 0.607460695 | 3.223725741 | 0.001265345 | 0.0057993 |
| *DKK3* | 1.95812616 | 0.516324401 | 3.792433899 | 0.000149178 | 0.000979543 |
| *TPD52L1* | 1.957634143 | 0.630089573 | 3.106914045 | 0.001890514 | 0.008097158 |
| *CD1A* | 1.954809487 | 0.558480842 | 3.500226579 | 0.000464863 | 0.002554365 |
| *ASGR1* | 1.95221743 | 0.369165788 | 5.288186214 | 1.24E-07 | 2.15E-06 |
| *CCR7* | 1.951837562 | 0.358398182 | 5.446002965 | 5.15E-08 | 9.88E-07 |
| *PLA2G5* | 1.951566058 | 0.804423238 | 2.426043861 | 0.015264425 | 0.044700337 |
| *AGTRAP* | 1.949620007 | 0.254457905 | 7.661856696 | 1.83E-14 | 1.43E-12 |
| *TBC1D8* | 1.948068296 | 0.327448649 | 5.949232954 | 2.69E-09 | 7.25E-08 |
| *OTULINL* | 1.947581756 | 0.308800074 | 6.306934225 | 2.85E-10 | 9.87E-09 |
| *RSPO2* | 1.946512522 | 0.587177269 | 3.315033848 | 0.00091632 | 0.004447567 |
| *HMCN1* | 1.945232957 | 0.419165242 | 4.640730578 | 3.47E-06 | 3.97E-05 |
| *TNIP3* | 1.945214315 | 0.393552873 | 4.94270135 | 7.70E-07 | 1.08E-05 |
| *CD40* | 1.94354797 | 0.29722142 | 6.53905754 | 6.19E-11 | 2.44E-09 |
| *PLPP4* | 1.94251811 | 0.784487559 | 2.47616178 | 0.013280339 | 0.039955579 |
| *HLA-DQB2* | 1.942182031 | 0.402087152 | 4.830251401 | 1.36E-06 | 1.76E-05 |
| *OSR2* | 1.942027101 | 0.535214019 | 3.628505668 | 0.000285066 | 0.001688319 |
| *SGMS2* | 1.941341714 | 0.386702462 | 5.020246587 | 5.16E-07 | 7.57E-06 |
| *RPL3L* | 1.941242025 | 0.642346575 | 3.022110026 | 0.002510193 | 0.010234312 |
| *SHROOM1* | 1.94065898 | 0.449449759 | 4.317855199 | 1.58E-05 | 0.000144481 |
| *RCVRN* | 1.940077632 | 0.314625014 | 6.166317183 | 6.99E-10 | 2.15E-08 |
| *ETV3L* | 1.938862512 | 0.439095574 | 4.415581997 | 1.01E-05 | 9.88E-05 |
| *FAM20C* | 1.936250716 | 0.414889195 | 4.666910442 | 3.06E-06 | 3.56E-05 |
| *CPED1* | 1.935734575 | 0.488191638 | 3.965112113 | 7.34E-05 | 0.000538389 |
| *TNFAIP2* | 1.935692666 | 0.279426204 | 6.927384196 | 4.29E-12 | 2.16E-10 |
| *BICDL2* | 1.934680457 | 0.538341815 | 3.593777047 | 0.000325919 | 0.001892732 |
| *HLA-DRB1* | 1.932749407 | 0.351746636 | 5.494720377 | 3.91E-08 | 7.77E-07 |
| *FGFR2* | 1.929126295 | 0.586088167 | 3.291529163 | 0.000996443 | 0.004749796 |
| *S100A11* | 1.928067012 | 0.292573961 | 6.590015763 | 4.40E-11 | 1.80E-09 |
| *NAMPT* | 1.926443973 | 0.380249293 | 5.066265753 | 4.06E-07 | 6.10E-06 |
| *MSC* | 1.924188997 | 0.514374282 | 3.740834374 | 0.00018341 | 0.001164935 |
| *ZFR2* | 1.92241956 | 0.424798923 | 4.525481242 | 6.03E-06 | 6.36E-05 |
| *SAMD4A* | 1.918908307 | 0.278567853 | 6.888477216 | 5.64E-12 | 2.79E-10 |
| *CHDH* | 1.918250187 | 0.489825376 | 3.916191932 | 9.00E-05 | 0.000638784 |
| *AQP10* | 1.917970893 | 0.53890302 | 3.559027916 | 0.00037223 | 0.002117777 |
| *CD209* | 1.91736249 | 0.536062451 | 3.576752085 | 0.00034789 | 0.002000509 |
| *PTGIR* | 1.916580556 | 0.328118645 | 5.84112053 | 5.19E-09 | 1.30E-07 |
| *UBXN11* | 1.915928614 | 0.188905785 | 10.14224426 | 3.59E-24 | 1.65E-21 |
| *EPHX3* | 1.915871714 | 0.403303328 | 4.750448566 | 2.03E-06 | 2.49E-05 |
| *KCNQ1* | 1.913242597 | 0.331055231 | 5.779224784 | 7.50E-09 | 1.78E-07 |
| *UTF1* | 1.912066341 | 0.515197415 | 3.711327516 | 0.000206175 | 0.001285033 |
| *CABP4* | 1.910088002 | 0.403029958 | 4.739320151 | 2.14E-06 | 2.61E-05 |
| *SLAMF7* | 1.908334248 | 0.335237141 | 5.692490524 | 1.25E-08 | 2.80E-07 |
| *RORA* | 1.908088087 | 0.36023123 | 5.296842498 | 1.18E-07 | 2.06E-06 |
| *ATP1A4* | 1.907655003 | 0.653921598 | 2.9172534 | 0.003531288 | 0.013517103 |
| *SLC16A14* | 1.906602574 | 0.432352671 | 4.409831841 | 1.03E-05 | 0.000101075 |
| *SIRPA* | 1.906589714 | 0.238578648 | 7.99145159 | 1.33E-15 | 1.32E-13 |
| *HPD* | 1.90289267 | 0.500414158 | 3.802635552 | 0.000143165 | 0.00094733 |
| *CRYAB* | 1.900652333 | 0.736290737 | 2.581388353 | 0.009840381 | 0.031468544 |
| *DTX4* | 1.898237041 | 0.386921423 | 4.906001397 | 9.30E-07 | 1.27E-05 |
| *ANGPTL4* | 1.89623342 | 0.720922548 | 2.630287298 | 0.008531274 | 0.027936899 |
| *SEL1L2* | 1.894875312 | 0.658635439 | 2.876971387 | 0.00401512 | 0.015013305 |
| *ECM2* | 1.894151774 | 0.493491339 | 3.838267515 | 0.000123905 | 0.000838826 |
| *SORCS3* | 1.892528571 | 0.535756786 | 3.532439756 | 0.000411744 | 0.002304893 |
| *DOCK4* | 1.89243932 | 0.373342975 | 5.068902989 | 4.00E-07 | 6.03E-06 |
| *RBP1* | 1.892072497 | 0.754704057 | 2.507038989 | 0.012174728 | 0.037276641 |
| *LRG1* | 1.891522923 | 0.320356611 | 5.904429179 | 3.54E-09 | 9.20E-08 |
| *PRUNE2* | 1.891137657 | 0.38377928 | 4.927670036 | 8.32E-07 | 1.15E-05 |
| *GPR160* | 1.888647795 | 0.339699467 | 5.559760849 | 2.70E-08 | 5.60E-07 |
| *PAQR9* | 1.888235268 | 0.756802336 | 2.495017757 | 0.012595083 | 0.03826391 |
| *FOXO1* | 1.88656764 | 0.296493024 | 6.362941078 | 1.98E-10 | 7.11E-09 |
| *RDH12* | 1.885788574 | 0.647454503 | 2.912619441 | 0.003584111 | 0.013687854 |
| *KDM7A* | 1.885176621 | 0.236155553 | 7.982774908 | 1.43E-15 | 1.40E-13 |
| *CD5* | 1.883217333 | 0.318768545 | 5.907789098 | 3.47E-09 | 9.03E-08 |
| *DIO1* | 1.880320157 | 0.554894157 | 3.38861048 | 0.000702477 | 0.003559158 |
| *ABCA4* | 1.873595256 | 0.648275889 | 2.890120222 | 0.003850945 | 0.01449703 |
| *GJB2* | 1.87253026 | 0.71627746 | 2.614252666 | 0.008942288 | 0.02906037 |
| *FFAR1* | 1.872488329 | 0.564821168 | 3.315187947 | 0.000915815 | 0.00444648 |
| *VGF* | 1.871645454 | 0.570859004 | 3.278647512 | 0.001043058 | 0.004934109 |
| *FAM133A* | 1.870521303 | 0.703778961 | 2.657824981 | 0.007864672 | 0.026130548 |
| *ZNF98* | 1.868696233 | 0.654989368 | 2.85301766 | 0.004330621 | 0.015964848 |
| *MUC12* | 1.868172648 | 0.40836698 | 4.574739732 | 4.77E-06 | 5.19E-05 |
| *RGS7* | 1.868081327 | 0.603934803 | 3.093183761 | 0.001980215 | 0.008415315 |
| *PYDC1* | 1.866542495 | 0.765949346 | 2.436900699 | 0.014813747 | 0.043647056 |
| *PLAG1* | 1.864353502 | 0.417719037 | 4.463175812 | 8.08E-06 | 8.18E-05 |
| *AIM2* | 1.863846101 | 0.358438078 | 5.199910989 | 1.99E-07 | 3.29E-06 |
| *SORT1* | 1.862383196 | 0.372536241 | 4.999200056 | 5.76E-07 | 8.32E-06 |
| *PXN* | 1.862086271 | 0.226253639 | 8.23008318 | 1.87E-16 | 2.12E-14 |
| *EGFL6* | 1.857032275 | 0.771325485 | 2.407585786 | 0.016058389 | 0.046513346 |
| *GNGT2* | 1.85550111 | 0.313343621 | 5.921617627 | 3.19E-09 | 8.37E-08 |
| *BMP8B* | 1.85434557 | 0.404236272 | 4.587281492 | 4.49E-06 | 4.92E-05 |
| *SNN* | 1.854316829 | 0.223653815 | 8.291013646 | 1.12E-16 | 1.35E-14 |
| *DZIP1L* | 1.853498063 | 0.374492819 | 4.949355419 | 7.45E-07 | 1.05E-05 |
| *CRIP3* | 1.85342358 | 0.492094994 | 3.766393889 | 0.000165622 | 0.001070425 |
| *ACP3* | 1.851373864 | 0.303081797 | 6.108495734 | 1.01E-09 | 2.98E-08 |
| *IFIT3* | 1.85115689 | 0.321483764 | 5.758166026 | 8.50E-09 | 1.99E-07 |
| *AICDA* | 1.850643295 | 0.627516924 | 2.949152802 | 0.003186464 | 0.012444996 |
| *GIMAP1* | 1.850551578 | 0.320211852 | 5.779147662 | 7.51E-09 | 1.78E-07 |
| *TMEM236* | 1.850251493 | 0.601477024 | 3.076179834 | 0.002096713 | 0.008822752 |
| *CXCR2* | 1.848637453 | 0.446202685 | 4.143044213 | 3.43E-05 | 0.000279118 |
| *ENTPD1* | 1.845319483 | 0.333071305 | 5.540313608 | 3.02E-08 | 6.18E-07 |
| *SLA* | 1.845154563 | 0.28075271 | 6.572170099 | 4.96E-11 | 2.00E-09 |
| *DUSP26* | 1.844642872 | 0.746381429 | 2.471447976 | 0.013456713 | 0.040373965 |
| *VWA5B2* | 1.844181093 | 0.631518806 | 2.920231476 | 0.003497715 | 0.013424293 |
| *CPVL* | 1.843710391 | 0.339629404 | 5.42859473 | 5.68E-08 | 1.08E-06 |
| *LSP1* | 1.843256398 | 0.269156926 | 6.848259219 | 7.48E-12 | 3.61E-10 |
| *KAZALD1* | 1.841257214 | 0.354571097 | 5.192913996 | 2.07E-07 | 3.40E-06 |
| *TNFSF12* | 1.840736407 | 0.320705902 | 5.739639956 | 9.49E-09 | 2.20E-07 |
| *E2F2* | 1.840347149 | 0.312807722 | 5.883317508 | 4.02E-09 | 1.03E-07 |
| *ADAP1* | 1.839745736 | 0.272599998 | 6.748883889 | 1.49E-11 | 6.75E-10 |
| *SLC35E4* | 1.838123366 | 0.300069432 | 6.125660161 | 9.03E-10 | 2.70E-08 |
| *CADM1* | 1.838064725 | 0.483778578 | 3.799392551 | 0.000145051 | 0.000956413 |
| *SLC1A1* | 1.833056923 | 0.68804342 | 2.664158788 | 0.007718112 | 0.025726499 |
| *ST6GALNAC2* | 1.831502541 | 0.328562143 | 5.574295698 | 2.49E-08 | 5.20E-07 |
| *NFKBIA* | 1.830150287 | 0.284160912 | 6.440541992 | 1.19E-10 | 4.45E-09 |
| *PLEKHG1* | 1.829876164 | 0.335826865 | 5.448867722 | 5.07E-08 | 9.74E-07 |
| *LHB* | 1.829210209 | 0.727436567 | 2.51459755 | 0.011916833 | 0.036660764 |
| *CNN1* | 1.828397919 | 0.689655003 | 2.651177636 | 0.008021164 | 0.026560458 |
| *CSGALNACT1* | 1.828248785 | 0.354985077 | 5.150213077 | 2.60E-07 | 4.16E-06 |
| *IFNGR2* | 1.827086157 | 0.222821531 | 8.199773812 | 2.41E-16 | 2.63E-14 |
| *CATSPERB* | 1.827075205 | 0.417098248 | 4.380443249 | 1.18E-05 | 0.000113274 |
| *LILRA4* | 1.826361628 | 0.468771234 | 3.896061652 | 9.78E-05 | 0.00068443 |
| *GADD45G* | 1.825970064 | 0.289849564 | 6.299716439 | 2.98E-10 | 1.02E-08 |
| *SLC6A4* | 1.825292081 | 0.438473362 | 4.162834595 | 3.14E-05 | 0.000258714 |
| *YBX2* | 1.82168778 | 0.596299966 | 3.054985549 | 0.002250714 | 0.009329395 |
| *ZFP36* | 1.821145619 | 0.252595043 | 7.2097441 | 5.61E-13 | 3.31E-11 |
| *PTK6* | 1.820825625 | 0.428736275 | 4.246959571 | 2.17E-05 | 0.000188761 |
| *MAL* | 1.819675274 | 0.358344644 | 5.078003276 | 3.81E-07 | 5.79E-06 |
| *REM2* | 1.819427338 | 0.34499054 | 5.273847035 | 1.34E-07 | 2.30E-06 |
| *TARM1* | 1.818815088 | 0.574220921 | 3.167448316 | 0.00153783 | 0.006815282 |
| *CCIN* | 1.818603963 | 0.418652415 | 4.343947149 | 1.40E-05 | 0.000130836 |
| *IRAK2* | 1.818315761 | 0.417163858 | 4.358756696 | 1.31E-05 | 0.000123382 |
| *SERPINB10* | 1.817238308 | 0.446028081 | 4.074268834 | 4.62E-05 | 0.000361329 |
| *POU2F2* | 1.816800842 | 0.298298569 | 6.090544942 | 1.13E-09 | 3.30E-08 |
| *FNDC1* | 1.816488579 | 0.610080737 | 2.977456046 | 0.002906513 | 0.011549658 |
| *L1TD1* | 1.814888227 | 0.724705713 | 2.504310639 | 0.012269026 | 0.037492836 |
| *UPP1* | 1.813105661 | 0.397278735 | 4.563812515 | 5.02E-06 | 5.43E-05 |
| *ABCG2* | 1.810240418 | 0.428747767 | 4.222157079 | 2.42E-05 | 0.000207531 |
| *EPHA4* | 1.80943824 | 0.394236931 | 4.589722827 | 4.44E-06 | 4.89E-05 |
| *SCPEP1* | 1.809427706 | 0.332832332 | 5.436454124 | 5.44E-08 | 1.04E-06 |
| *IVNS1ABP* | 1.806211543 | 0.279166498 | 6.470015398 | 9.80E-11 | 3.72E-09 |
| *ASIP* | 1.805299802 | 0.595481658 | 3.031663152 | 0.002432104 | 0.009966433 |
| *TUBB1* | 1.803364546 | 0.479833603 | 3.758312327 | 0.000171063 | 0.001098859 |
| *SLAMF8* | 1.802902865 | 0.371721455 | 4.85014475 | 1.23E-06 | 1.62E-05 |
| *GP5* | 1.802305886 | 0.376322165 | 4.78926317 | 1.67E-06 | 2.11E-05 |
| *CCR3* | 1.800581362 | 0.417633849 | 4.311387514 | 1.62E-05 | 0.000147831 |
| *KRT86* | 1.800062241 | 0.562378836 | 3.200800114 | 0.001370466 | 0.006210057 |
| *FAM151B* | 1.79952074 | 0.269795909 | 6.66993338 | 2.56E-11 | 1.09E-09 |
| *SPDEF* | 1.799376233 | 0.549071519 | 3.277125419 | 0.001048698 | 0.004952807 |
| *PPARD* | 1.798928976 | 0.287719081 | 6.252379829 | 4.04E-10 | 1.34E-08 |
| *CD300C* | 1.798762228 | 0.368532552 | 4.880877468 | 1.06E-06 | 1.42E-05 |
| *GAL* | 1.798650192 | 0.522732139 | 3.440863987 | 0.00057986 | 0.003049956 |
| *C9orf72* | 1.798613649 | 0.206208183 | 8.72231949 | 2.73E-18 | 4.57E-16 |
| *COL1A2* | 1.798554042 | 0.741951805 | 2.424084733 | 0.015347024 | 0.044893161 |
| *MAB21L3* | 1.796429214 | 0.461111501 | 3.89586729 | 9.78E-05 | 0.000684828 |
| *JUNB* | 1.795957303 | 0.21772805 | 8.248626239 | 1.60E-16 | 1.85E-14 |
| *MAPK7* | 1.795392301 | 0.255499158 | 7.026998897 | 2.11E-12 | 1.12E-10 |
| *KCNK7* | 1.795151743 | 0.307523223 | 5.837450995 | 5.30E-09 | 1.32E-07 |
| *STOM* | 1.79340458 | 0.250223372 | 7.16721451 | 7.65E-13 | 4.37E-11 |
| *MFSD2A* | 1.793016203 | 0.354736084 | 5.054507511 | 4.32E-07 | 6.46E-06 |
| *MS4A14* | 1.792146801 | 0.407420252 | 4.398767103 | 1.09E-05 | 0.000105748 |
| *IGSF23* | 1.791142047 | 0.583077331 | 3.071877352 | 0.002127171 | 0.008924815 |
| *NGEF* | 1.790256966 | 0.584058967 | 3.06519901 | 0.002175252 | 0.009079774 |
| *PTPRJ* | 1.789820482 | 0.319793877 | 5.596794092 | 2.18E-08 | 4.64E-07 |
| *BATF2* | 1.789096719 | 0.366250679 | 4.884896662 | 1.03E-06 | 1.39E-05 |
| *COPZ2* | 1.785710823 | 0.391400582 | 4.56236118 | 5.06E-06 | 5.46E-05 |
| *KLRC2* | 1.785667743 | 0.559312473 | 3.192612051 | 0.001409922 | 0.006359718 |
| *PLA2G2D* | 1.785423727 | 0.550498811 | 3.243283532 | 0.001181606 | 0.005477362 |
| *FAM186B* | 1.785170555 | 0.448671218 | 3.978794452 | 6.93E-05 | 0.000511948 |
| *ARNTL* | 1.784211492 | 0.285089877 | 6.258417556 | 3.89E-10 | 1.29E-08 |
| *C6orf141* | 1.783223236 | 0.732090908 | 2.435794814 | 0.014859111 | 0.043747171 |
| *ODF3B* | 1.782456196 | 0.326653901 | 5.456711802 | 4.85E-08 | 9.37E-07 |
| *SYNGR3* | 1.782133654 | 0.346138834 | 5.148609397 | 2.62E-07 | 4.19E-06 |
| *ZBTB7B* | 1.781765878 | 0.206085238 | 8.645771498 | 5.34E-18 | 8.46E-16 |
| *RASD2* | 1.781604517 | 0.544831799 | 3.270008322 | 0.001075443 | 0.005058578 |
| *DPEP3* | 1.781596691 | 0.289173059 | 6.161005106 | 7.23E-10 | 2.20E-08 |
| *RORB* | 1.780328804 | 0.604607424 | 2.944602951 | 0.003233693 | 0.012603521 |
| *KCNJ15* | 1.780194115 | 0.549838394 | 3.237667893 | 0.00120511 | 0.005561074 |
| *CHST7* | 1.779089831 | 0.306071779 | 5.812655571 | 6.15E-09 | 1.50E-07 |
| *TSPAN14* | 1.777942976 | 0.208350581 | 8.533419829 | 1.42E-17 | 2.02E-15 |
| *EGR4* | 1.776667335 | 0.463572209 | 3.832557904 | 0.000126818 | 0.000855432 |
| *WSCD2* | 1.774486892 | 0.626339238 | 2.833108297 | 0.004609777 | 0.016800263 |
| *NACC2* | 1.772862312 | 0.292355847 | 6.064056294 | 1.33E-09 | 3.82E-08 |
| *CDHR1* | 1.772468825 | 0.389992081 | 4.54488414 | 5.50E-06 | 5.87E-05 |
| *NCKAP1* | 1.76983756 | 0.377146408 | 4.692706919 | 2.70E-06 | 3.19E-05 |
| *NPAS2* | 1.769667985 | 0.346075017 | 5.113538677 | 3.16E-07 | 4.92E-06 |
| *TMEM154* | 1.767917175 | 0.252690966 | 6.996360811 | 2.63E-12 | 1.37E-10 |
| *CEMIP2* | 1.767702247 | 0.216418271 | 8.167989865 | 3.14E-16 | 3.38E-14 |
| *CCDC68* | 1.76731191 | 0.508545096 | 3.475231448 | 0.000510413 | 0.002752108 |
| *GPR17* | 1.767144796 | 0.549777861 | 3.214288753 | 0.001307681 | 0.005969995 |
| *ITLN1* | 1.765014772 | 0.412646286 | 4.277306821 | 1.89E-05 | 0.000168637 |
| *TF* | 1.764311932 | 0.360665707 | 4.89182059 | 9.99E-07 | 1.35E-05 |
| *RIC3* | 1.763097772 | 0.326339479 | 5.402649346 | 6.57E-08 | 1.22E-06 |
| *EYA2* | 1.762967429 | 0.573263344 | 3.07531861 | 0.002102778 | 0.008843568 |
| *RASSF4* | 1.760595184 | 0.360003995 | 4.890487907 | 1.01E-06 | 1.36E-05 |
| *PLA2G2C* | 1.760503648 | 0.577611066 | 3.047904989 | 0.002304428 | 0.009529584 |
| *BRI3* | 1.757737578 | 0.220006093 | 7.989494985 | 1.35E-15 | 1.34E-13 |
| *ITGB2* | 1.757329868 | 0.226483587 | 7.759193012 | 8.55E-15 | 7.08E-13 |
| *GPX3* | 1.755952813 | 0.427238585 | 4.110005219 | 3.96E-05 | 0.000315563 |
| *SERPINB9* | 1.755905401 | 0.31982072 | 5.4902803 | 4.01E-08 | 7.94E-07 |
| *CHL1* | 1.755846893 | 0.636248444 | 2.759687525 | 0.005785667 | 0.020304998 |
| *PRKACG* | 1.755098436 | 0.465301601 | 3.771958728 | 0.000161971 | 0.001052413 |
| *AOAH* | 1.753516614 | 0.280607723 | 6.248996265 | 4.13E-10 | 1.36E-08 |
| *CPE* | 1.751830659 | 0.541714465 | 3.233863543 | 0.001221278 | 0.005619299 |
| *PTPRN2* | 1.748999482 | 0.375820238 | 4.653819316 | 3.26E-06 | 3.75E-05 |
| *PRDM1* | 1.748003643 | 0.35847472 | 4.876225698 | 1.08E-06 | 1.45E-05 |
| *TAGLN* | 1.747772531 | 0.258640363 | 6.757539732 | 1.40E-11 | 6.43E-10 |
| *ECHDC3* | 1.747640576 | 0.280880557 | 6.222006219 | 4.91E-10 | 1.58E-08 |
| *DMWD* | 1.746121549 | 0.266147944 | 6.560717775 | 5.35E-11 | 2.14E-09 |
| *FNIP2* | 1.745095603 | 0.248590702 | 7.019955254 | 2.22E-12 | 1.17E-10 |
| *CAMK4* | 1.74480197 | 0.345754313 | 5.046363571 | 4.50E-07 | 6.70E-06 |
| *S100A6* | 1.743935177 | 0.22790342 | 7.652079874 | 1.98E-14 | 1.51E-12 |
| *C1orf162* | 1.743440825 | 0.229994173 | 7.58036955 | 3.45E-14 | 2.51E-12 |
| *IL1A* | 1.743138886 | 0.600452243 | 2.903043341 | 0.003695555 | 0.014015393 |
| *BCL11B* | 1.742818538 | 0.33633264 | 5.181829926 | 2.20E-07 | 3.57E-06 |
| *IL1R1* | 1.741967143 | 0.444473917 | 3.919166181 | 8.89E-05 | 0.00063309 |
| *ZFPM2* | 1.740983295 | 0.502629613 | 3.463749945 | 0.000532701 | 0.002846109 |
| *CTSZ* | 1.740677894 | 0.200723926 | 8.672000061 | 4.25E-18 | 6.86E-16 |
| *SLC22A18AS* | 1.739554269 | 0.337226167 | 5.158420191 | 2.49E-07 | 4.00E-06 |
| *SHISA7* | 1.738993567 | 0.601816682 | 2.889573553 | 0.003857647 | 0.014518807 |
| *FILIP1L* | 1.738895788 | 0.38616392 | 4.502999104 | 6.70E-06 | 6.96E-05 |
| *PXT1* | 1.73785255 | 0.364491371 | 4.767883932 | 1.86E-06 | 2.31E-05 |
| *NELL2* | 1.737753274 | 0.372918449 | 4.659874776 | 3.16E-06 | 3.67E-05 |
| *MUC13* | 1.735673669 | 0.662164036 | 2.621214041 | 0.008761723 | 0.028567423 |
| *TCIRG1* | 1.73513845 | 0.187546834 | 9.251760824 | 2.21E-20 | 5.18E-18 |
| *ADGRD1* | 1.735069063 | 0.4585926 | 3.783465023 | 0.00015466 | 0.001010399 |
| *PLPPR2* | 1.734866515 | 0.212410898 | 8.167502382 | 3.15E-16 | 3.38E-14 |
| *GPR141* | 1.733485554 | 0.312870863 | 5.540578433 | 3.01E-08 | 6.18E-07 |
| *ZAR1* | 1.732974172 | 0.698734975 | 2.480159478 | 0.013132363 | 0.03956763 |
| *CFAP45* | 1.73203069 | 0.306825302 | 5.645006061 | 1.65E-08 | 3.60E-07 |
| *GDF15* | 1.731808124 | 0.505865552 | 3.423455337 | 0.000618304 | 0.003215019 |
| *PACSIN1* | 1.731065467 | 0.459740146 | 3.765312825 | 0.000166341 | 0.001073752 |
| *CLEC18B* | 1.73105641 | 0.35037357 | 4.940602142 | 7.79E-07 | 1.09E-05 |
| *BCAR1* | 1.729353446 | 0.618910573 | 2.794189535 | 0.005202997 | 0.018602449 |
| *ALOX5* | 1.728717508 | 0.312908088 | 5.524681452 | 3.30E-08 | 6.68E-07 |
| *GP6* | 1.728385131 | 0.418075483 | 4.134146108 | 3.56E-05 | 0.000289261 |
| *GCNT2* | 1.726649525 | 0.278499023 | 6.199840502 | 5.65E-10 | 1.78E-08 |
| *PIM3* | 1.726535679 | 0.281312811 | 6.137422875 | 8.39E-10 | 2.53E-08 |
| *SGPP1* | 1.7260773 | 0.368580452 | 4.683040815 | 2.83E-06 | 3.32E-05 |
| *AATK* | 1.726024479 | 0.357120598 | 4.833169777 | 1.34E-06 | 1.74E-05 |
| *FGD6* | 1.725949224 | 0.314229513 | 5.492638833 | 3.96E-08 | 7.85E-07 |
| *RAI14* | 1.725079458 | 0.580147633 | 2.973518046 | 0.002944071 | 0.011673423 |
| *TLR2* | 1.724733771 | 0.214871275 | 8.026823368 | 1.00E-15 | 1.01E-13 |
| *GABBR1* | 1.723717427 | 0.378215548 | 4.557500177 | 5.18E-06 | 5.57E-05 |
| *FCGR2A* | 1.723178918 | 0.318271338 | 5.414181906 | 6.16E-08 | 1.15E-06 |
| *SYNE2* | 1.720798285 | 0.344462494 | 4.995604206 | 5.87E-07 | 8.46E-06 |
| *HLA-DMB* | 1.720697554 | 0.327784934 | 5.249471155 | 1.53E-07 | 2.59E-06 |
| *GREM2* | 1.719446947 | 0.53590854 | 3.208470881 | 0.001334428 | 0.006071092 |
| *FFAR3* | 1.716976008 | 0.504735879 | 3.401731638 | 0.000669604 | 0.003425496 |
| *FAIM2* | 1.716742463 | 0.641993915 | 2.674079026 | 0.007493479 | 0.025112595 |
| *IZUMO1* | 1.716153213 | 0.64914703 | 2.643704943 | 0.008200411 | 0.027038037 |
| *PDE1A* | 1.715408149 | 0.50238501 | 3.414528923 | 0.000638924 | 0.003298937 |
| *CDKN1A* | 1.714112862 | 0.336761159 | 5.089995727 | 3.58E-07 | 5.47E-06 |
| *IRF8* | 1.713880657 | 0.363358191 | 4.716780025 | 2.40E-06 | 2.88E-05 |
| *COL21A1* | 1.713497647 | 0.558800044 | 3.066387818 | 0.002166621 | 0.00905433 |
| *PLEKHN1* | 1.711629198 | 0.336460861 | 5.087156921 | 3.63E-07 | 5.55E-06 |
| *IL6ST* | 1.710408715 | 0.233699812 | 7.31882794 | 2.50E-13 | 1.56E-11 |
| *MCOLN3* | 1.709774515 | 0.495275477 | 3.452168731 | 0.0005561 | 0.002947739 |
| *PPM1J* | 1.709683878 | 0.411147248 | 4.158324994 | 3.21E-05 | 0.000263121 |
| *LTA4H* | 1.708694412 | 0.186439769 | 9.164860152 | 4.96E-20 | 1.07E-17 |
| *PDE6H* | 1.708578958 | 0.4085817 | 4.181731479 | 2.89E-05 | 0.000241 |
| *FAM153B* | 1.707564906 | 0.448979215 | 3.803215934 | 0.00014283 | 0.000945903 |
| *AMY1C* | 1.706060667 | 0.519656517 | 3.283054502 | 0.001026888 | 0.0048678 |
| *HHIPL1* | 1.703871685 | 0.629728787 | 2.705723034 | 0.006815586 | 0.023196882 |
| *SLC18B1* | 1.702218084 | 0.277728984 | 6.129061719 | 8.84E-10 | 2.65E-08 |
| *CXADR* | 1.701793642 | 0.563332925 | 3.020937652 | 0.002519932 | 0.010267857 |
| *SGTB* | 1.701326686 | 0.200143493 | 8.500534594 | 1.89E-17 | 2.56E-15 |
| *TLR1* | 1.698724551 | 0.215883903 | 7.868694834 | 3.58E-15 | 3.20E-13 |
| *ARHGEF11* | 1.69573736 | 0.319941644 | 5.300145792 | 1.16E-07 | 2.03E-06 |
| *ARAP3* | 1.695063257 | 0.369768538 | 4.584119751 | 4.56E-06 | 4.99E-05 |
| *RXRA* | 1.694045633 | 0.260936444 | 6.492177193 | 8.46E-11 | 3.26E-09 |
| *CIBAR2* | 1.693952476 | 0.709713274 | 2.386812447 | 0.016995159 | 0.048670052 |
| *RNASE6* | 1.693256112 | 0.381066325 | 4.443468239 | 8.85E-06 | 8.83E-05 |
| *AP1M2* | 1.69296194 | 0.497292753 | 3.404356751 | 0.000663201 | 0.003399333 |
| *NR4A1* | 1.691224063 | 0.355223072 | 4.761019753 | 1.93E-06 | 2.38E-05 |
| *ZDHHC19* | 1.690623645 | 0.461320902 | 3.664745381 | 0.000247585 | 0.001500295 |
| *IL27* | 1.687759196 | 0.536178552 | 3.147755891 | 0.00164529 | 0.007217754 |
| *TP53AIP1* | 1.687545546 | 0.536081682 | 3.14792615 | 0.001644332 | 0.007214551 |
| *RNF112* | 1.687443882 | 0.367393397 | 4.593016362 | 4.37E-06 | 4.83E-05 |
| *TAFA1* | 1.686356497 | 0.566878262 | 2.974812427 | 0.002931677 | 0.011632638 |
| *USP2* | 1.684196582 | 0.394324585 | 4.271092003 | 1.95E-05 | 0.000172532 |
| *FMNL2* | 1.683288341 | 0.29465956 | 5.712654761 | 1.11E-08 | 2.53E-07 |
| *GPR137B* | 1.68316148 | 0.271384258 | 6.202133812 | 5.57E-10 | 1.76E-08 |
| *DPYD* | 1.681259888 | 0.254450967 | 6.607402226 | 3.91E-11 | 1.62E-09 |
| *SLC12A3* | 1.679657412 | 0.563685425 | 2.979777972 | 0.002884574 | 0.011471617 |
| *SLC35D3* | 1.677484301 | 0.444178953 | 3.776595652 | 0.000158987 | 0.001035571 |
| *NAIP* | 1.676443692 | 0.254201626 | 6.594936934 | 4.25E-11 | 1.74E-09 |
| *MYBPC1* | 1.676351851 | 0.553849897 | 3.026725941 | 0.00247218 | 0.010111024 |
| *ABCA6* | 1.675860922 | 0.350339415 | 4.783535192 | 1.72E-06 | 2.17E-05 |
| *GAS6* | 1.67468535 | 0.405945827 | 4.12539122 | 3.70E-05 | 0.000298346 |
| *EML4* | 1.673423706 | 0.214907301 | 7.786723382 | 6.88E-15 | 5.88E-13 |
| *TFEB* | 1.671969293 | 0.264997122 | 6.309386604 | 2.80E-10 | 9.73E-09 |
| *SVEP1* | 1.671854844 | 0.687818291 | 2.430663542 | 0.015071203 | 0.044224508 |
| *ACSS3* | 1.67150138 | 0.613671081 | 2.723774074 | 0.006454064 | 0.02221591 |
| *OSGIN1* | 1.670066171 | 0.316295272 | 5.280085792 | 1.29E-07 | 2.23E-06 |
| *SLC10A4* | 1.668441261 | 0.458730725 | 3.637081997 | 0.000275744 | 0.001643562 |
| *CYP3A5* | 1.668382438 | 0.608415661 | 2.742175364 | 0.006103375 | 0.021222367 |
| *AFDN* | 1.667454783 | 0.42208978 | 3.950474192 | 7.80E-05 | 0.000567208 |
| *TNFSF10* | 1.666635118 | 0.310795965 | 5.362473474 | 8.21E-08 | 1.50E-06 |
| *PLEKHO2* | 1.664192169 | 0.219061281 | 7.596925202 | 3.03E-14 | 2.23E-12 |
| *CLRN3* | 1.662771458 | 0.640288278 | 2.596910666 | 0.009406639 | 0.03032039 |
| *GNAZ* | 1.662044991 | 0.394876581 | 4.209023958 | 2.56E-05 | 0.000217726 |
| *PPARGC1A* | 1.66177258 | 0.690591066 | 2.406304775 | 0.016114814 | 0.046646906 |
| *OSCAR* | 1.661204303 | 0.36758959 | 4.519182124 | 6.21E-06 | 6.52E-05 |
| *CDC42EP1* | 1.656894088 | 0.530131356 | 3.125440647 | 0.001775389 | 0.00768623 |
| *SLC16A10* | 1.656557094 | 0.428309475 | 3.867663899 | 0.000109883 | 0.000756679 |
| *UGT8* | 1.656254303 | 0.410616111 | 4.033583337 | 5.49E-05 | 0.000419308 |
| *CLIC6* | 1.654421944 | 0.61547115 | 2.688057669 | 0.007186898 | 0.024267568 |
| *MYBPHL* | 1.653890995 | 0.466410067 | 3.546001927 | 0.000391123 | 0.002207023 |
| *JAZF1* | 1.650637345 | 0.289647221 | 5.698785376 | 1.21E-08 | 2.72E-07 |
| *GPRIN1* | 1.650321176 | 0.333604401 | 4.946940662 | 7.54E-07 | 1.06E-05 |
| *HELZ2* | 1.649380722 | 0.277233685 | 5.949423933 | 2.69E-09 | 7.24E-08 |
| *TBC1D30* | 1.648148583 | 0.246044416 | 6.698581554 | 2.10E-11 | 9.19E-10 |
| *CYRIA* | 1.646990105 | 0.269611001 | 6.108764466 | 1.00E-09 | 2.98E-08 |
| *TNFSF14* | 1.646031864 | 0.357265863 | 4.607302394 | 4.08E-06 | 4.55E-05 |
| *KIF1B* | 1.642629241 | 0.206474064 | 7.95562022 | 1.78E-15 | 1.70E-13 |
| *SLITRK5* | 1.641216961 | 0.600420812 | 2.733444491 | 0.006267569 | 0.021686713 |
| *SNX18* | 1.641137067 | 0.26353697 | 6.227350435 | 4.74E-10 | 1.53E-08 |
| *EPOR* | 1.639511446 | 0.276013241 | 5.939973898 | 2.85E-09 | 7.62E-08 |
| *ITGAX* | 1.638915485 | 0.262195451 | 6.250739586 | 4.09E-10 | 1.35E-08 |
| *POTEM* | 1.638507114 | 0.361641727 | 4.530746847 | 5.88E-06 | 6.22E-05 |
| *NME8* | 1.637290687 | 0.322660599 | 5.074343414 | 3.89E-07 | 5.88E-06 |
| *VASP* | 1.636774827 | 0.159781185 | 10.24385213 | 1.26E-24 | 6.65E-22 |
| *ZCCHC24* | 1.632134797 | 0.271258269 | 6.01690339 | 1.78E-09 | 4.98E-08 |
| *GP9* | 1.631659014 | 0.483176429 | 3.376942492 | 0.000732964 | 0.003687065 |
| *FAM209B* | 1.63143673 | 0.307580715 | 5.304093034 | 1.13E-07 | 1.99E-06 |
| *GIMAP5* | 1.631057194 | 0.345215334 | 4.724753033 | 2.30E-06 | 2.78E-05 |
| *PPFIA4* | 1.630746602 | 0.433833375 | 3.758923811 | 0.000170646 | 0.0010964 |
| *FSTL3* | 1.630630507 | 0.280704565 | 5.809063006 | 6.28E-09 | 1.53E-07 |
| *CFP* | 1.629210367 | 0.253559065 | 6.425368248 | 1.32E-10 | 4.88E-09 |
| *IFIT1B* | 1.628666947 | 0.633568632 | 2.57062434 | 0.010151538 | 0.032229173 |
| *FZD8* | 1.626919078 | 0.417257714 | 3.899074893 | 9.66E-05 | 0.000677317 |
| *ADGRG3* | 1.626533358 | 0.453522014 | 3.586448529 | 0.000335212 | 0.001938523 |
| *SIPA1L2* | 1.625679976 | 0.378199155 | 4.298475959 | 1.72E-05 | 0.00015551 |
| *PTPRO* | 1.624581726 | 0.384472129 | 4.225486338 | 2.38E-05 | 0.000204986 |
| *STPG4* | 1.624537154 | 0.351529201 | 4.6213434 | 3.81E-06 | 4.29E-05 |
| *PLEKHH2* | 1.624151705 | 0.484326599 | 3.35342248 | 0.000798188 | 0.003960412 |
| *CLEC5A* | 1.623558123 | 0.416023466 | 3.902563811 | 9.52E-05 | 0.000669107 |
| *CDH23* | 1.623393276 | 0.321980729 | 5.041895763 | 4.61E-07 | 6.84E-06 |
| *KCTD7* | 1.622106069 | 0.269378188 | 6.021668197 | 1.73E-09 | 4.84E-08 |
| *TBC1D9* | 1.621472458 | 0.44813014 | 3.618307075 | 0.000296536 | 0.001746448 |
| *VSIR* | 1.621338177 | 0.222807831 | 7.276845564 | 3.42E-13 | 2.10E-11 |
| *TSPO* | 1.620272843 | 0.205250807 | 7.894111933 | 2.92E-15 | 2.66E-13 |
| *NTRK2* | 1.619291877 | 0.570598405 | 2.83788364 | 0.004541373 | 0.016592996 |
| *ADAM19* | 1.618602223 | 0.323458426 | 5.004050274 | 5.61E-07 | 8.15E-06 |
| *GPR176* | 1.618527396 | 0.420337353 | 3.850543814 | 0.000117856 | 0.00080285 |
| *UBE2D1* | 1.618398854 | 0.219117483 | 7.385986889 | 1.51E-13 | 9.86E-12 |
| *ALDH1A3* | 1.618289653 | 0.646930152 | 2.501490538 | 0.012367175 | 0.037684684 |
| *OGFRL1* | 1.61824767 | 0.214589714 | 7.54112413 | 4.66E-14 | 3.32E-12 |
| *UNC119* | 1.617204531 | 0.188865858 | 8.562715071 | 1.10E-17 | 1.62E-15 |
| *SAMHD1* | 1.617003154 | 0.353079705 | 4.579711411 | 4.66E-06 | 5.08E-05 |
| *TSHZ2* | 1.616494631 | 0.426973642 | 3.785935415 | 0.000153131 | 0.001002799 |
| *ASAP1* | 1.613421344 | 0.241393085 | 6.683792714 | 2.33E-11 | 1.01E-09 |
| *HLX* | 1.612926663 | 0.22407472 | 7.198164374 | 6.10E-13 | 3.55E-11 |
| *AP1S2* | 1.612653785 | 0.225837393 | 7.140774003 | 9.28E-13 | 5.20E-11 |
| *TBC1D2* | 1.612507933 | 0.240751378 | 6.697813941 | 2.12E-11 | 9.20E-10 |
| *PLK3* | 1.612323063 | 0.269314519 | 5.986766215 | 2.14E-09 | 5.91E-08 |
| *ARHGEF28* | 1.61094628 | 0.44988157 | 3.580823014 | 0.000342514 | 0.001972585 |
| *ABCA9* | 1.610881533 | 0.399368601 | 4.033570813 | 5.49E-05 | 0.000419308 |
| *POTEI* | 1.610495698 | 0.445623319 | 3.61402922 | 0.000301475 | 0.001769608 |
| *GPAT3* | 1.607822757 | 0.314062251 | 5.119439703 | 3.06E-07 | 4.79E-06 |
| *FGF7* | 1.607531432 | 0.560052663 | 2.870321912 | 0.004100541 | 0.015260608 |
| *THBS2* | 1.605638263 | 0.671380327 | 2.391547978 | 0.016777491 | 0.048225575 |
| *VNN1* | 1.603342006 | 0.426024231 | 3.763499557 | 0.000167552 | 0.001079999 |
| *PLA2G4C* | 1.602900346 | 0.405439654 | 3.953486868 | 7.70E-05 | 0.0005614 |
| *CTSD* | 1.602647295 | 0.24465671 | 6.550596115 | 5.73E-11 | 2.28E-09 |
| *RBP4* | 1.600929969 | 0.526249019 | 3.042152881 | 0.002348926 | 0.009681817 |
| *CLEC12B* | 1.600615995 | 0.316158281 | 5.062704641 | 4.13E-07 | 6.21E-06 |
| *KIR2DL1* | 1.600342661 | 0.563321787 | 2.840903188 | 0.004498597 | 0.016461401 |
| *ITGAD* | 1.596878403 | 0.308182105 | 5.181606509 | 2.20E-07 | 3.57E-06 |
| *IGFBP3* | 1.596303283 | 0.404198018 | 3.949310018 | 7.84E-05 | 0.000569843 |
| *CASR* | 1.59629363 | 0.475319045 | 3.358362447 | 0.000784057 | 0.003900082 |
| *CSF1* | 1.59484111 | 0.452400154 | 3.525288609 | 0.000423022 | 0.002356771 |
| *PHEX* | 1.594096882 | 0.407878983 | 3.90825943 | 9.30E-05 | 0.000657217 |
| *LY86* | 1.593784219 | 0.456138965 | 3.494076019 | 0.000475706 | 0.002599954 |
| *ARRB2* | 1.592831443 | 0.17455829 | 9.124925802 | 7.18E-20 | 1.49E-17 |
| *PLXNC1* | 1.59166631 | 0.285665459 | 5.571784263 | 2.52E-08 | 5.26E-07 |
| *HGD* | 1.589649697 | 0.29677277 | 5.356454013 | 8.49E-08 | 1.54E-06 |
| *HTR3A* | 1.589454771 | 0.646410228 | 2.458894835 | 0.013936544 | 0.041569323 |
| *SLC6A8* | 1.588280151 | 0.448705694 | 3.539692433 | 0.000400594 | 0.002250433 |
| *CPNE3* | 1.583394307 | 0.246023236 | 6.435954309 | 1.23E-10 | 4.56E-09 |
| *GRB7* | 1.582857773 | 0.614142261 | 2.577347098 | 0.009956192 | 0.031758687 |
| *KCNS1* | 1.582835063 | 0.604297367 | 2.619298293 | 0.008811086 | 0.028707673 |
| *TNFRSF11A* | 1.581440655 | 0.385763099 | 4.09951252 | 4.14E-05 | 0.000328067 |
| *NPC2* | 1.58104975 | 0.171620382 | 9.212482425 | 3.19E-20 | 7.31E-18 |
| *RASGRP1* | 1.58013743 | 0.314532166 | 5.02377054 | 5.07E-07 | 7.46E-06 |
| *TNFRSF17* | 1.579713492 | 0.472245071 | 3.345113777 | 0.000822488 | 0.004056823 |
| *IRF4* | 1.579669642 | 0.342129957 | 4.617162606 | 3.89E-06 | 4.37E-05 |
| *TNFSF15* | 1.579606408 | 0.399337029 | 3.955572094 | 7.64E-05 | 0.000557167 |
| *SERINC2* | 1.577569906 | 0.418421582 | 3.770288088 | 0.000163059 | 0.00105818 |
| *ANK2* | 1.57551724 | 0.511648006 | 3.079299091 | 0.002074883 | 0.008744839 |
| *EBF1* | 1.574638672 | 0.361068236 | 4.361055651 | 1.29E-05 | 0.000122275 |
| *FAM171A1* | 1.57345017 | 0.590512238 | 2.664551331 | 0.00770911 | 0.025704612 |
| *LFNG* | 1.57325712 | 0.308359639 | 5.102020236 | 3.36E-07 | 5.17E-06 |
| *ATOH8* | 1.570114426 | 0.372804161 | 4.211633318 | 2.54E-05 | 0.000215515 |
| *NADK* | 1.569858329 | 0.177911566 | 8.823812651 | 1.11E-18 | 1.93E-16 |
| *BATF3* | 1.567560855 | 0.291433449 | 5.378795257 | 7.50E-08 | 1.38E-06 |
| *PSAT1* | 1.567484621 | 0.369395748 | 4.243374835 | 2.20E-05 | 0.000191067 |
| *INKA2* | 1.565999067 | 0.219077277 | 7.148158333 | 8.79E-13 | 4.96E-11 |
| *CASP1* | 1.565887296 | 0.236336039 | 6.625681382 | 3.46E-11 | 1.44E-09 |
| *RAB11FIP4* | 1.564734677 | 0.285406454 | 5.482478251 | 4.19E-08 | 8.27E-07 |
| *RHOU* | 1.564067788 | 0.37008544 | 4.226234325 | 2.38E-05 | 0.000204362 |
| *TRABD2A* | 1.563822051 | 0.308037974 | 5.076718409 | 3.84E-07 | 5.82E-06 |
| *BVES* | 1.563292174 | 0.530890966 | 2.944657706 | 0.003233121 | 0.012602843 |
| *HEG1* | 1.562835027 | 0.287548108 | 5.435038464 | 5.48E-08 | 1.04E-06 |
| *ECSCR* | 1.562006663 | 0.422750875 | 3.694863227 | 0.000220005 | 0.001355844 |
| *GAS2L3* | 1.561446115 | 0.30463893 | 5.125563285 | 2.97E-07 | 4.66E-06 |
| *RAB43* | 1.560855994 | 0.293959346 | 5.309768224 | 1.10E-07 | 1.94E-06 |
| *VOPP1* | 1.560130367 | 0.220783169 | 7.066346483 | 1.59E-12 | 8.58E-11 |
| *CCDC170* | 1.55973741 | 0.430103027 | 3.626427416 | 0.00028737 | 0.001699415 |
| *CTTNBP2* | 1.55973057 | 0.646909263 | 2.411049984 | 0.015906668 | 0.046230398 |
| *HNRNPLL* | 1.559414634 | 0.335215158 | 4.651981266 | 3.29E-06 | 3.78E-05 |
| *CDO1* | 1.55736153 | 0.50515047 | 3.082965617 | 0.002049488 | 0.008656251 |
| *PSRC1* | 1.557304113 | 0.273367597 | 5.696739967 | 1.22E-08 | 2.75E-07 |
| *CECR2* | 1.557231302 | 0.563619969 | 2.762910096 | 0.005728854 | 0.020139102 |
| *GPR82* | 1.556892549 | 0.489008823 | 3.183771897 | 0.001453695 | 0.006520002 |
| *NFKBIZ* | 1.554945248 | 0.254327271 | 6.113954036 | 9.72E-10 | 2.89E-08 |
| *AKTIP* | 1.553668479 | 0.208021151 | 7.468800518 | 8.09E-14 | 5.52E-12 |
| *MAK* | 1.552903804 | 0.291889017 | 5.320185809 | 1.04E-07 | 1.84E-06 |
| *MAP1B* | 1.55273238 | 0.623068529 | 2.492073197 | 0.012699986 | 0.038538225 |
| *EFHD2* | 1.552023828 | 0.14914027 | 10.40647054 | 2.32E-25 | 1.38E-22 |
| *SLC49A3* | 1.550429099 | 0.300826651 | 5.153895422 | 2.55E-07 | 4.09E-06 |
| *SPTBN4* | 1.55018343 | 0.521514094 | 2.972466995 | 0.00295417 | 0.01170428 |
| *LST1* | 1.549146625 | 0.223306989 | 6.937295743 | 4.00E-12 | 2.03E-10 |
| *KLHL34* | 1.548903448 | 0.640024485 | 2.420069049 | 0.01551756 | 0.045299141 |
| *CD274* | 1.548836232 | 0.34842615 | 4.445235328 | 8.78E-06 | 8.76E-05 |
| *PIP4P2* | 1.548238907 | 0.198643981 | 7.794038839 | 6.49E-15 | 5.61E-13 |
| *BLVRB* | 1.547776679 | 0.213143232 | 7.261674074 | 3.82E-13 | 2.32E-11 |
| *INHBB* | 1.546493106 | 0.39476726 | 3.917480658 | 8.95E-05 | 0.000636466 |
| *SAMSN1* | 1.546451059 | 0.284586955 | 5.434019489 | 5.51E-08 | 1.05E-06 |
| *DOK2* | 1.54642772 | 0.283142548 | 5.461657858 | 4.72E-08 | 9.18E-07 |
| *MCUB* | 1.545785102 | 0.302951659 | 5.102415043 | 3.35E-07 | 5.17E-06 |
| *MLXIPL* | 1.545668791 | 0.626812811 | 2.465917677 | 0.013666274 | 0.040898049 |
| *SPRED1* | 1.543958391 | 0.465935477 | 3.313674247 | 0.000920787 | 0.00446309 |
| *HEPACAM2* | 1.543767597 | 0.48512269 | 3.182220969 | 0.001461503 | 0.006549452 |
| *SPX* | 1.543018683 | 0.471616627 | 3.271764809 | 0.001068784 | 0.005034738 |
| *EPHB1* | 1.542968633 | 0.383741024 | 4.020859214 | 5.80E-05 | 0.000439457 |
| *GPR42* | 1.54188319 | 0.578337023 | 2.666063433 | 0.007674521 | 0.025616263 |
| *ADTRP* | 1.541167638 | 0.352306648 | 4.374506259 | 1.22E-05 | 0.000115873 |
| *MCU* | 1.541161886 | 0.204483154 | 7.536864793 | 4.81E-14 | 3.42E-12 |
| *CAPN8* | 1.540124578 | 0.604363485 | 2.548341549 | 0.010823644 | 0.033900592 |
| *FKBP9* | 1.540111589 | 0.345519444 | 4.457380371 | 8.30E-06 | 8.36E-05 |
| *ZDHHC18* | 1.539714272 | 0.222257439 | 6.927616355 | 4.28E-12 | 2.16E-10 |
| *IFITM3* | 1.53970261 | 0.343754022 | 4.47908246 | 7.50E-06 | 7.67E-05 |
| *KCNE2* | 1.539105317 | 0.558637265 | 2.755106783 | 0.005867298 | 0.020518691 |
| *GLIPR2* | 1.536156326 | 0.200647407 | 7.655998899 | 1.92E-14 | 1.48E-12 |
| *SERPINE2* | 1.535917258 | 0.404207506 | 3.799823688 | 0.000144799 | 0.000955149 |
| *TVP23A* | 1.535186595 | 0.368822977 | 4.162394134 | 3.15E-05 | 0.000259079 |
| *IFNGR1* | 1.533494565 | 0.24542777 | 6.248252049 | 4.15E-10 | 1.36E-08 |
| *ADAM9* | 1.533300056 | 0.326659359 | 4.693880688 | 2.68E-06 | 3.17E-05 |
| *SIGLEC10* | 1.533106505 | 0.276085967 | 5.553004085 | 2.81E-08 | 5.80E-07 |
| *GNAO1* | 1.532838275 | 0.381710472 | 4.01570926 | 5.93E-05 | 0.000447986 |
| *UBTD1* | 1.532057695 | 0.246582546 | 6.213163572 | 5.19E-10 | 1.66E-08 |
| *IFI6* | 1.53094456 | 0.282520065 | 5.418887884 | 6.00E-08 | 1.13E-06 |
| *WARS1* | 1.530751838 | 0.188517925 | 8.119927251 | 4.66E-16 | 4.83E-14 |
| *TMEM266* | 1.530232064 | 0.439407305 | 3.482491176 | 0.000496772 | 0.002693697 |
| *AOC2* | 1.529519016 | 0.250987577 | 6.094002868 | 1.10E-09 | 3.24E-08 |
| *KDF1* | 1.527611236 | 0.42468253 | 3.597066344 | 0.000321826 | 0.001872058 |
| *GPR35* | 1.527344773 | 0.277909818 | 5.495828767 | 3.89E-08 | 7.73E-07 |
| *CA1* | 1.527278192 | 0.610489822 | 2.501725888 | 0.012358957 | 0.037676729 |
| *GNG12* | 1.525014673 | 0.501125566 | 3.043178748 | 0.002340932 | 0.009655317 |
| *MPZL3* | 1.524987776 | 0.229308453 | 6.650377489 | 2.92E-11 | 1.23E-09 |
| *CD226* | 1.524885936 | 0.305450001 | 4.992260365 | 5.97E-07 | 8.58E-06 |
| *SNCG* | 1.524760821 | 0.521065164 | 2.926238264 | 0.003430881 | 0.013214254 |
| *KMO* | 1.523917069 | 0.321497305 | 4.740061728 | 2.14E-06 | 2.60E-05 |
| *THBS4* | 1.523439059 | 0.451402485 | 3.374901797 | 0.00073842 | 0.003710386 |
| *PRG4* | 1.522753857 | 0.408883773 | 3.724172877 | 0.000195957 | 0.001231527 |
| *SRGAP2B* | 1.521945749 | 0.199995226 | 7.6099104 | 2.74E-14 | 2.04E-12 |
| *PPM1M* | 1.521636668 | 0.221627299 | 6.865745661 | 6.61E-12 | 3.25E-10 |
| *TSPAN15* | 1.520389243 | 0.437010867 | 3.479065074 | 0.000503166 | 0.00272138 |
| *HCAR3* | 1.520067697 | 0.375433796 | 4.048830216 | 5.15E-05 | 0.000396465 |
| *ENPP4* | 1.519932577 | 0.336282541 | 4.519808174 | 6.19E-06 | 6.51E-05 |
| *FUCA1* | 1.519640556 | 0.228419607 | 6.652846375 | 2.87E-11 | 1.21E-09 |
| *SLC41A2* | 1.517582677 | 0.319995006 | 4.742519873 | 2.11E-06 | 2.57E-05 |
| *KL* | 1.516780909 | 0.450676822 | 3.365562275 | 0.000763878 | 0.003818007 |
| *EPAS1* | 1.516651545 | 0.379636452 | 3.995010328 | 6.47E-05 | 0.000482079 |
| *STX3* | 1.515594875 | 0.220359151 | 6.877839512 | 6.08E-12 | 3.00E-10 |
| *DAGLA* | 1.515360957 | 0.388039745 | 3.905169445 | 9.42E-05 | 0.000663117 |
| *BCL2L11* | 1.513443044 | 0.282064049 | 5.36560064 | 8.07E-08 | 1.47E-06 |
| *METRNL* | 1.512331422 | 0.309427224 | 4.887518949 | 1.02E-06 | 1.38E-05 |
| *VSTM1* | 1.511869562 | 0.487142961 | 3.103543896 | 0.001912179 | 0.008170054 |
| *MSRB1* | 1.508901277 | 0.259120471 | 5.823165069 | 5.77E-09 | 1.42E-07 |
| *GGN* | 1.505703515 | 0.388274729 | 3.877933341 | 0.000105348 | 0.000730053 |
| *BMP8A* | 1.504058122 | 0.480877848 | 3.127734262 | 0.001761594 | 0.00763695 |
| *CD40LG* | 1.504049984 | 0.309181583 | 4.864617004 | 1.15E-06 | 1.52E-05 |
| *BICDL1* | 1.503999385 | 0.312301223 | 4.815861339 | 1.47E-06 | 1.88E-05 |
| *GFRA2* | 1.503780162 | 0.426005072 | 3.529958349 | 0.000415625 | 0.002322512 |
| *RGS10* | 1.502950055 | 0.260770857 | 5.763489332 | 8.24E-09 | 1.93E-07 |
| *HES4* | 1.502373084 | 0.463788526 | 3.239349404 | 0.001198027 | 0.005537606 |
| *TUFT1* | 1.50005271 | 0.359811753 | 4.168993082 | 3.06E-05 | 0.000252677 |
| *SLC22A23* | 1.499688152 | 0.404645268 | 3.706179881 | 0.000210409 | 0.00130602 |
| *HLA-DQA2* | 1.499213649 | 0.49554376 | 3.025391036 | 0.002483119 | 0.010147896 |
| *TNFSF9* | 1.499206547 | 0.38867306 | 3.857243276 | 0.000114673 | 0.000785564 |
| *EML5* | 1.498375403 | 0.321148345 | 4.665679977 | 3.08E-06 | 3.58E-05 |
| *NUP62CL* | 1.498021295 | 0.475816511 | 3.148317176 | 0.001642134 | 0.007206904 |
| *TMPRSS4* | 1.498016356 | 0.627143248 | 2.388635069 | 0.016911091 | 0.048486323 |
| *NKAPL* | 1.497436433 | 0.439079484 | 3.410399452 | 0.000648678 | 0.003337722 |
| *JDP2* | 1.496451595 | 0.270792057 | 5.526201959 | 3.27E-08 | 6.63E-07 |
| *MMP17* | 1.496269929 | 0.352999107 | 4.238735741 | 2.25E-05 | 0.000194631 |
| *RARA* | 1.49554956 | 0.181763543 | 8.227995216 | 1.90E-16 | 2.15E-14 |
| *HLA-DRA* | 1.494220722 | 0.330101233 | 4.526552985 | 6.00E-06 | 6.33E-05 |
| *RIPOR2* | 1.493671199 | 0.247746794 | 6.029023314 | 1.65E-09 | 4.65E-08 |
| *HEY2* | 1.493444399 | 0.491976831 | 3.035599046 | 0.002400584 | 0.009860241 |
| *KCNMB1* | 1.492858917 | 0.346117844 | 4.313152127 | 1.61E-05 | 0.000146909 |
| *ZDBF2* | 1.49270155 | 0.410431634 | 3.63690668 | 0.000275932 | 0.001643752 |
| *AMPD1* | 1.490398926 | 0.618484919 | 2.409757911 | 0.015963109 | 0.04634432 |
| *SORCS2* | 1.490221591 | 0.479893027 | 3.105320366 | 0.00190073 | 0.008129918 |
| *SCN1B* | 1.490206302 | 0.291256822 | 5.116468313 | 3.11E-07 | 4.85E-06 |
| *TRIM55* | 1.489872287 | 0.558957257 | 2.665449403 | 0.00768855 | 0.025649567 |
| *CLEC2L* | 1.489513654 | 0.574441091 | 2.592978944 | 0.00951486 | 0.030606889 |
| *DEF8* | 1.488325104 | 0.179059905 | 8.311883713 | 9.42E-17 | 1.16E-14 |
| *SPOCK2* | 1.487784808 | 0.320909695 | 4.636147898 | 3.55E-06 | 4.05E-05 |
| *PDE4B* | 1.486825374 | 0.367334872 | 4.04760203 | 5.17E-05 | 0.000398066 |
| *MAST3* | 1.486203234 | 0.195881178 | 7.587269234 | 3.27E-14 | 2.38E-12 |
| *PLA1A* | 1.486087153 | 0.354388049 | 4.193389581 | 2.75E-05 | 0.000230575 |
| *HID1* | 1.485282497 | 0.333116498 | 4.458747928 | 8.24E-06 | 8.31E-05 |
| *TCF7* | 1.483399734 | 0.331160811 | 4.479393949 | 7.49E-06 | 7.67E-05 |
| *TRIB1* | 1.483280065 | 0.26587927 | 5.578772904 | 2.42E-08 | 5.09E-07 |
| *ZC3H12A* | 1.482424752 | 0.316777039 | 4.679710228 | 2.87E-06 | 3.37E-05 |
| *C20orf27* | 1.481238328 | 0.204303894 | 7.250171773 | 4.16E-13 | 2.51E-11 |
| *SLFN5* | 1.48022082 | 0.304435994 | 4.862174153 | 1.16E-06 | 1.53E-05 |
| *ITK* | 1.47911457 | 0.316140811 | 4.678657478 | 2.89E-06 | 3.38E-05 |
| *RHBDL3* | 1.479107905 | 0.519834248 | 2.845345243 | 0.004436331 | 0.016278718 |
| *ABHD5* | 1.479056282 | 0.247698117 | 5.971205184 | 2.36E-09 | 6.44E-08 |
| *CTSB* | 1.478837077 | 0.244776687 | 6.04157649 | 1.53E-09 | 4.33E-08 |
| *RAPSN* | 1.478636551 | 0.304617628 | 4.854074141 | 1.21E-06 | 1.59E-05 |
| *CYP4F22* | 1.477786928 | 0.411990797 | 3.586941603 | 0.000334579 | 0.001936968 |
| *ZNF365* | 1.475985986 | 0.399502725 | 3.694557998 | 0.00022027 | 0.001356851 |
| *BTNL8* | 1.475303149 | 0.448632075 | 3.288447777 | 0.001007415 | 0.004794155 |
| *EHD4* | 1.474948189 | 0.25721976 | 5.734194719 | 9.80E-09 | 2.26E-07 |
| *FRAT2* | 1.474087398 | 0.210247396 | 7.01120408 | 2.36E-12 | 1.24E-10 |
| *CACNA2D3* | 1.473903756 | 0.553136461 | 2.664629543 | 0.007707318 | 0.025701342 |
| *ENDOU* | 1.472824096 | 0.45487957 | 3.237833027 | 0.001204413 | 0.005558666 |
| *USP3* | 1.472459109 | 0.180828413 | 8.1428526 | 3.86E-16 | 4.06E-14 |
| *BEST1* | 1.47194213 | 0.265703491 | 5.539792207 | 3.03E-08 | 6.19E-07 |
| *CNIH2* | 1.470903703 | 0.241247703 | 6.097068223 | 1.08E-09 | 3.18E-08 |
| *DSCAML1* | 1.470662629 | 0.433007891 | 3.396387593 | 0.000682816 | 0.003481273 |
| *ISM1* | 1.470604364 | 0.469669574 | 3.131146762 | 0.001741251 | 0.007561186 |
| *SPI1* | 1.470568645 | 0.175557578 | 8.376560353 | 5.45E-17 | 6.96E-15 |
| *HBA1* | 1.466752303 | 0.570879873 | 2.569283614 | 0.010190901 | 0.032331676 |
| *PLEKHG3* | 1.465515537 | 0.31857272 | 4.600254335 | 4.22E-06 | 4.68E-05 |
| *CDC42EP2* | 1.462619738 | 0.212420942 | 6.885478081 | 5.76E-12 | 2.85E-10 |
| *PRKACA* | 1.461911204 | 0.177780011 | 8.223147248 | 1.98E-16 | 2.23E-14 |
| *GSDMA* | 1.461869466 | 0.403704237 | 3.621139763 | 0.000293308 | 0.001729365 |
| *PSTPIP1* | 1.46132467 | 0.257480725 | 5.675472088 | 1.38E-08 | 3.06E-07 |
| *KIAA1755* | 1.459717822 | 0.547691878 | 2.665217214 | 0.007693861 | 0.02566458 |
| *HLA-DPA1* | 1.457082203 | 0.349445926 | 4.169692922 | 3.05E-05 | 0.000252099 |
| *OMG* | 1.455523243 | 0.269268962 | 5.405462374 | 6.46E-08 | 1.21E-06 |
| *C17orf107* | 1.454012095 | 0.335286781 | 4.336622193 | 1.45E-05 | 0.000134477 |
| *CTRC* | 1.453468843 | 0.43838746 | 3.315489097 | 0.000914829 | 0.004443055 |
| *SEMA3D* | 1.451258519 | 0.525421064 | 2.762086677 | 0.005743323 | 0.020182466 |
| *HAVCR2* | 1.450907478 | 0.302346996 | 4.798815586 | 1.60E-06 | 2.02E-05 |
| *OAS2* | 1.446576467 | 0.30067751 | 4.811056422 | 1.50E-06 | 1.91E-05 |
| *ARL4C* | 1.446516236 | 0.27498895 | 5.260270406 | 1.44E-07 | 2.46E-06 |
| *AVPR2* | 1.444547477 | 0.479222698 | 3.014355296 | 0.00257526 | 0.010454275 |
| *CAPN3* | 1.443523493 | 0.288362725 | 5.005929569 | 5.56E-07 | 8.09E-06 |
| *CDC42EP4* | 1.44301679 | 0.275233767 | 5.242877007 | 1.58E-07 | 2.67E-06 |
| *IL1B* | 1.442046411 | 0.367351998 | 3.925516724 | 8.65E-05 | 0.000619265 |
| *SH3RF2* | 1.441572875 | 0.47376698 | 3.042788828 | 0.002343968 | 0.009665317 |
| *TMEM150B* | 1.440505454 | 0.382382533 | 3.767184241 | 0.000165099 | 0.001068134 |
| *ST3GAL5* | 1.439880341 | 0.237506945 | 6.062476782 | 1.34E-09 | 3.85E-08 |
| *SLC4A3* | 1.438125821 | 0.491539327 | 2.925759427 | 0.003436165 | 0.01322978 |
| *NLRP1* | 1.437851395 | 0.230689286 | 6.232848594 | 4.58E-10 | 1.48E-08 |
| *TYROBP* | 1.437560193 | 0.214029577 | 6.716642686 | 1.86E-11 | 8.24E-10 |
| *FCGR2B* | 1.437538048 | 0.450189125 | 3.193186969 | 0.001407118 | 0.006347973 |
| *EPS8* | 1.436526717 | 0.376760485 | 3.812838058 | 0.00013738 | 0.000914789 |
| *UNC93B1* | 1.436166863 | 0.20556554 | 6.986418358 | 2.82E-12 | 1.46E-10 |
| *SMAD7* | 1.436114806 | 0.357373598 | 4.018525188 | 5.86E-05 | 0.000443302 |
| *CEACAM4* | 1.435399472 | 0.317997429 | 4.51387131 | 6.37E-06 | 6.66E-05 |
| *FTL* | 1.434865462 | 0.200632668 | 7.151704022 | 8.57E-13 | 4.85E-11 |
| *DEDD2* | 1.43477961 | 0.169745565 | 8.452530753 | 2.85E-17 | 3.81E-15 |
| *CD52* | 1.434355959 | 0.371985892 | 3.85594183 | 0.000115285 | 0.000788561 |
| *KCNG1* | 1.434007886 | 0.539626548 | 2.657407964 | 0.007874409 | 0.026156588 |
| *ANKRD29* | 1.433861911 | 0.429120829 | 3.341394345 | 0.000833587 | 0.004101343 |
| *HLA-B* | 1.432298009 | 0.222283235 | 6.443571898 | 1.17E-10 | 4.37E-09 |
| *MAP2K1* | 1.43164738 | 0.194872298 | 7.346592593 | 2.03E-13 | 1.29E-11 |
| *RASGRF2* | 1.430480577 | 0.339979206 | 4.207553141 | 2.58E-05 | 0.000218972 |
| *PLCL2* | 1.428801712 | 0.214440674 | 6.662923048 | 2.68E-11 | 1.14E-09 |
| *SHTN1* | 1.42843948 | 0.471987342 | 3.026435991 | 0.002474552 | 0.010119419 |
| *TMPRSS13* | 1.427585736 | 0.327834259 | 4.354595945 | 1.33E-05 | 0.000125413 |
| *SLC26A8* | 1.4269106 | 0.345757053 | 4.126916833 | 3.68E-05 | 0.000296677 |
| *APOL3* | 1.426867385 | 0.263224006 | 5.420734257 | 5.94E-08 | 1.12E-06 |
| *FAM153A* | 1.426789336 | 0.433207923 | 3.293543955 | 0.000989329 | 0.004727274 |
| *RIMS2* | 1.425335621 | 0.520368391 | 2.739089548 | 0.006160959 | 0.021380324 |
| *STS* | 1.424108841 | 0.284773971 | 5.000839214 | 5.71E-07 | 8.25E-06 |
| *MICAL2* | 1.423185693 | 0.264774845 | 5.375078936 | 7.65E-08 | 1.40E-06 |
| *MYO1F* | 1.423034568 | 0.17449597 | 8.155114222 | 3.49E-16 | 3.68E-14 |
| *TIGAR* | 1.422557694 | 0.209705617 | 6.783593662 | 1.17E-11 | 5.45E-10 |
| *SLC6A6* | 1.420636595 | 0.214529376 | 6.622107511 | 3.54E-11 | 1.47E-09 |
| *PCLO* | 1.419628966 | 0.542087 | 2.618821268 | 0.008823416 | 0.028741929 |
| *NAGA* | 1.418452253 | 0.241893815 | 5.86394593 | 4.52E-09 | 1.14E-07 |
| *OTUD1* | 1.418137689 | 0.286706035 | 4.946312648 | 7.56E-07 | 1.06E-05 |
| *SYTL3* | 1.417879171 | 0.330397573 | 4.29143337 | 1.78E-05 | 0.000159605 |
| *PLXDC1* | 1.41620046 | 0.31601174 | 4.48148053 | 7.41E-06 | 7.60E-05 |
| *ACE* | 1.414704529 | 0.343798433 | 4.114924307 | 3.87E-05 | 0.000309982 |
| *AREG* | 1.414663049 | 0.576572284 | 2.453574495 | 0.014144424 | 0.042076076 |
| *PLEC* | 1.413913122 | 0.227122039 | 6.225345326 | 4.80E-10 | 1.55E-08 |
| *FAM110D* | 1.413144726 | 0.574184458 | 2.461133713 | 0.013849874 | 0.04134587 |
| *RMDN2* | 1.412606555 | 0.321278597 | 4.396827457 | 1.10E-05 | 0.000106468 |
| *ARHGEF12* | 1.409597365 | 0.290475413 | 4.852725234 | 1.22E-06 | 1.60E-05 |
| *KCNA2* | 1.408731522 | 0.513334748 | 2.74427462 | 0.006064478 | 0.021107404 |
| *HAPLN3* | 1.407460948 | 0.286432026 | 4.913769488 | 8.93E-07 | 1.23E-05 |
| *SAPCD2* | 1.407317076 | 0.247423655 | 5.687884104 | 1.29E-08 | 2.87E-07 |
| *ZNF821* | 1.406510015 | 0.263975068 | 5.328192641 | 9.92E-08 | 1.78E-06 |
| *ANKRD37* | 1.405444676 | 0.288859937 | 4.865488405 | 1.14E-06 | 1.51E-05 |
| *GCHFR* | 1.40365708 | 0.271839707 | 5.163546911 | 2.42E-07 | 3.90E-06 |
| *HSPA1A* | 1.403275295 | 0.331505459 | 4.233038273 | 2.31E-05 | 0.000198976 |
| *EVI2B* | 1.402159887 | 0.199727395 | 7.020368385 | 2.21E-12 | 1.17E-10 |
| *SLAMF1* | 1.402158959 | 0.318861108 | 4.397397246 | 1.10E-05 | 0.000106286 |
| *IGF1* | 1.40108136 | 0.44723473 | 3.132765111 | 0.00173168 | 0.00752685 |
| *ATG2A* | 1.400110456 | 0.23368954 | 5.991327032 | 2.08E-09 | 5.76E-08 |
| *TLE6* | 1.400010901 | 0.541779484 | 2.584097298 | 0.009763425 | 0.031279321 |
| *RGCC* | 1.399231552 | 0.342226705 | 4.088610064 | 4.34E-05 | 0.000342243 |
| *GSAP* | 1.399128934 | 0.200684184 | 6.971794721 | 3.13E-12 | 1.61E-10 |
| *CYP46A1* | 1.398884079 | 0.451559982 | 3.097892048 | 0.001949024 | 0.008299476 |
| *XPNPEP2* | 1.398851982 | 0.361954385 | 3.864718978 | 0.000111217 | 0.000765035 |
| *ACOXL* | 1.398711554 | 0.550843938 | 2.53921566 | 0.011110131 | 0.034634931 |
| *RTP5* | 1.398697119 | 0.470019544 | 2.975827571 | 0.002921991 | 0.011602929 |
| *SRXN1* | 1.398524984 | 0.254440589 | 5.496469693 | 3.87E-08 | 7.70E-07 |
| *CAST* | 1.397055691 | 0.219160104 | 6.374589477 | 1.83E-10 | 6.62E-09 |
| *MAP3K3* | 1.396941508 | 0.166189211 | 8.405729229 | 4.25E-17 | 5.52E-15 |
| *SPATA12* | 1.396827777 | 0.506266708 | 2.759074917 | 0.005796524 | 0.020326949 |
| *PAK1* | 1.396714378 | 0.196712675 | 7.100276456 | 1.25E-12 | 6.77E-11 |
| *IGLL5* | 1.392457234 | 0.530575271 | 2.624429199 | 0.008679434 | 0.028332219 |
| *PPP1R18* | 1.392045834 | 0.182562986 | 7.625016807 | 2.44E-14 | 1.85E-12 |
| *REC8* | 1.391527014 | 0.248416659 | 5.601584932 | 2.12E-08 | 4.52E-07 |
| *CXCL3* | 1.391151101 | 0.45426093 | 3.062449374 | 0.002195336 | 0.009140544 |
| *PIK3IP1* | 1.388634013 | 0.281235994 | 4.937611262 | 7.91E-07 | 1.10E-05 |
| *FHAD1* | 1.388633242 | 0.417851243 | 3.323271772 | 0.000889682 | 0.004335556 |
| *CORO2A* | 1.388330094 | 0.292537248 | 4.745823318 | 2.08E-06 | 2.54E-05 |
| *FBXO2* | 1.388225912 | 0.343100628 | 4.046118834 | 5.21E-05 | 0.000400303 |
| *CRTAM* | 1.386537276 | 0.295356626 | 4.694451225 | 2.67E-06 | 3.16E-05 |
| *ODF3L1* | 1.385202285 | 0.442412105 | 3.131022564 | 0.001741988 | 0.007563347 |
| *JCHAIN* | 1.384690397 | 0.394316623 | 3.511620651 | 0.000445383 | 0.002464438 |
| *CLEC12A* | 1.384612872 | 0.291908768 | 4.743306897 | 2.10E-06 | 2.57E-05 |
| *METTL7B* | 1.382916248 | 0.45153615 | 3.062692211 | 0.002193556 | 0.009137938 |
| *WIPI1* | 1.382806417 | 0.294207532 | 4.70010543 | 2.60E-06 | 3.08E-05 |
| *COL6A3* | 1.379440146 | 0.48349348 | 2.853068767 | 0.004329925 | 0.015964185 |
| *B9D1* | 1.377771819 | 0.288439075 | 4.776647617 | 1.78E-06 | 2.23E-05 |
| *ABCB11* | 1.37725774 | 0.521637002 | 2.640260823 | 0.008284224 | 0.027254771 |
| *LTA* | 1.377002303 | 0.321277832 | 4.28601716 | 1.82E-05 | 0.000163081 |
| *ARHGAP31* | 1.376980079 | 0.344685894 | 3.994883752 | 6.47E-05 | 0.000482223 |
| *WWTR1* | 1.376945143 | 0.338069099 | 4.072969537 | 4.64E-05 | 0.000363082 |
| *CRABP2* | 1.375310815 | 0.308654915 | 4.455820235 | 8.36E-06 | 8.41E-05 |
| *OAS3* | 1.374848085 | 0.258144283 | 5.325890108 | 1.00E-07 | 1.80E-06 |
| *FBN2* | 1.374243555 | 0.460653317 | 2.983249015 | 0.002852058 | 0.011362291 |
| *OSBPL11* | 1.373063574 | 0.197716339 | 6.944613592 | 3.79E-12 | 1.93E-10 |
| *IER5* | 1.371883245 | 0.253043761 | 5.421525667 | 5.91E-08 | 1.12E-06 |
| *NSUN7* | 1.371850731 | 0.47314628 | 2.899421994 | 0.003738514 | 0.014142733 |
| *CPPED1* | 1.371508126 | 0.221325437 | 6.196793937 | 5.76E-10 | 1.81E-08 |
| *SRGAP2C* | 1.371065859 | 0.209882562 | 6.532538238 | 6.47E-11 | 2.55E-09 |
| *S100A4* | 1.370182106 | 0.23530886 | 5.82290912 | 5.78E-09 | 1.42E-07 |
| *LMTK2* | 1.369907923 | 0.250910949 | 5.459737516 | 4.77E-08 | 9.24E-07 |
| *PNOC* | 1.369500782 | 0.382171446 | 3.58347228 | 0.000339057 | 0.001955755 |
| *ACP5* | 1.369288151 | 0.297198029 | 4.607325811 | 4.08E-06 | 4.55E-05 |
| *AGFG1* | 1.36918697 | 0.206605994 | 6.627043803 | 3.42E-11 | 1.43E-09 |
| *PTPRH* | 1.36883744 | 0.491610855 | 2.784392219 | 0.005362814 | 0.019085469 |
| *HLA-DPB1* | 1.368647826 | 0.329503304 | 4.153669509 | 3.27E-05 | 0.000267564 |
| *RELB* | 1.365738215 | 0.262506298 | 5.20268742 | 1.96E-07 | 3.25E-06 |
| *TFCP2L1* | 1.364238493 | 0.45149147 | 3.021626287 | 0.002514207 | 0.010248487 |
| *SH2B3* | 1.364142378 | 0.180166161 | 7.571579299 | 3.69E-14 | 2.67E-12 |
| *SEC14L1* | 1.363711784 | 0.16862131 | 8.087422553 | 6.09E-16 | 6.24E-14 |
| *MBD2* | 1.360446065 | 0.138389736 | 9.830541644 | 8.32E-23 | 2.96E-20 |
| *TJP2* | 1.359478022 | 0.250699427 | 5.422740832 | 5.87E-08 | 1.11E-06 |
| *ANKDD1A* | 1.35859247 | 0.296706759 | 4.578906369 | 4.67E-06 | 5.09E-05 |
| *APOBEC3B* | 1.357783306 | 0.247546652 | 5.48495928 | 4.14E-08 | 8.16E-07 |
| *ULBP2* | 1.356545559 | 0.420008708 | 3.229803416 | 0.001238754 | 0.005688958 |
| *CREB5* | 1.356449195 | 0.35903764 | 3.7780139 | 0.000158084 | 0.001029905 |
| *MYBPC3* | 1.3551623 | 0.214674626 | 6.312633793 | 2.74E-10 | 9.57E-09 |
| *TMEM63B* | 1.355149065 | 0.173980102 | 7.789103741 | 6.75E-15 | 5.79E-13 |
| *EREG* | 1.354978192 | 0.519068153 | 2.610405173 | 0.009043504 | 0.029344113 |
| *PCSK1N* | 1.3546271 | 0.405776679 | 3.338356222 | 0.000842756 | 0.004140021 |
| *LILRB4* | 1.353851916 | 0.524333166 | 2.582045167 | 0.009821673 | 0.031421413 |
| *SLC16A3* | 1.353672755 | 0.24540466 | 5.516084145 | 3.47E-08 | 6.98E-07 |
| *LACTB* | 1.353004555 | 0.178770261 | 7.568398398 | 3.78E-14 | 2.72E-12 |
| *SULT1B1* | 1.352689869 | 0.320661069 | 4.218441215 | 2.46E-05 | 0.00021041 |
| *CYP1A1* | 1.352404785 | 0.542193651 | 2.494320586 | 0.012619851 | 0.038323391 |
| *CEP85L* | 1.352060062 | 0.227758883 | 5.936365875 | 2.91E-09 | 7.76E-08 |
| *HTR7* | 1.351817759 | 0.455121294 | 2.970236237 | 0.002975708 | 0.011768993 |
| *TADA2B* | 1.351456925 | 0.228928427 | 5.903403721 | 3.56E-09 | 9.24E-08 |
| *ARHGAP44* | 1.351109744 | 0.542974142 | 2.48835007 | 0.012833734 | 0.038862127 |
| *LRRC75A* | 1.350216535 | 0.305340346 | 4.422004999 | 9.78E-06 | 9.63E-05 |
| *MXD1* | 1.349872919 | 0.283426771 | 4.76268673 | 1.91E-06 | 2.36E-05 |
| *IL4R* | 1.349584005 | 0.231248517 | 5.836076378 | 5.34E-09 | 1.33E-07 |
| *GPM6A* | 1.348884905 | 0.508493078 | 2.652710457 | 0.007984833 | 0.026459529 |
| *XCL1* | 1.348123844 | 0.424345196 | 3.176950883 | 0.001488322 | 0.006647994 |
| *VSIG1* | 1.348112351 | 0.378840358 | 3.558523594 | 0.000372945 | 0.002120323 |
| *KIF21B* | 1.347790243 | 0.238835671 | 5.643169784 | 1.67E-08 | 3.62E-07 |
| *KIF1C* | 1.346928498 | 0.232242295 | 5.799669242 | 6.64E-09 | 1.61E-07 |
| *MVP* | 1.346885744 | 0.177050644 | 7.607347338 | 2.80E-14 | 2.07E-12 |
| *CLEC18A* | 1.34640447 | 0.345794573 | 3.893654139 | 9.87E-05 | 0.000689888 |
| *SNX9* | 1.345863563 | 0.342649567 | 3.927813402 | 8.57E-05 | 0.000613938 |
| *GRINA* | 1.344520503 | 0.216266426 | 6.21696362 | 5.07E-10 | 1.62E-08 |
| *IL13RA1* | 1.3430755 | 0.290862547 | 4.617560823 | 3.88E-06 | 4.36E-05 |
| *KLRB1* | 1.342729826 | 0.318311082 | 4.218294317 | 2.46E-05 | 0.000210459 |
| *SLC7A11* | 1.342227779 | 0.389103396 | 3.449540132 | 0.000561542 | 0.002970619 |
| *CCDC50* | 1.342186528 | 0.368320773 | 3.644069589 | 0.000268361 | 0.001606511 |
| *C10orf105* | 1.341821714 | 0.354891795 | 3.780931917 | 0.000156242 | 0.001018746 |
| *HERPUD1* | 1.340282731 | 0.1910173 | 7.016551551 | 2.27E-12 | 1.20E-10 |
| *ITSN1* | 1.339888203 | 0.264040216 | 5.074561087 | 3.88E-07 | 5.88E-06 |
| *BAIAP3* | 1.339151882 | 0.31087869 | 4.307634859 | 1.65E-05 | 0.000150059 |
| *TMEM170B* | 1.338968875 | 0.262432484 | 5.102146108 | 3.36E-07 | 5.17E-06 |
| *KLF15* | 1.337800865 | 0.52878059 | 2.529973473 | 0.011407115 | 0.03539537 |
| *CETP* | 1.337770165 | 0.503030325 | 2.659422503 | 0.007827474 | 0.026019794 |
| *ADIRF* | 1.337492245 | 0.49513501 | 2.701267774 | 0.00690757 | 0.023456987 |
| *RORC* | 1.33733025 | 0.313088111 | 4.27141818 | 1.94E-05 | 0.000172376 |
| *ELFN1* | 1.337094987 | 0.467328388 | 2.86114651 | 0.004221119 | 0.01561755 |
| *RASSF2* | 1.335722952 | 0.185510109 | 7.200270434 | 6.01E-13 | 3.51E-11 |
| *STXBP2* | 1.335074826 | 0.183798194 | 7.263808189 | 3.76E-13 | 2.30E-11 |
| *ELF3* | 1.334856941 | 0.261480296 | 5.105000106 | 3.31E-07 | 5.11E-06 |
| *GRPR* | 1.333038971 | 0.240100714 | 5.551999201 | 2.82E-08 | 5.83E-07 |
| *GGT2* | 1.33291582 | 0.423352923 | 3.148474355 | 0.001641251 | 0.007205027 |
| *ABCA1* | 1.332112551 | 0.374639591 | 3.555717506 | 0.000376949 | 0.002139626 |
| *DNAJC5B* | 1.331680678 | 0.424889369 | 3.134182156 | 0.001723338 | 0.007500894 |
| *GRN* | 1.3309526 | 0.279921536 | 4.754734552 | 1.99E-06 | 2.44E-05 |
| *RAB24* | 1.328885429 | 0.210780253 | 6.304601174 | 2.89E-10 | 9.99E-09 |
| *SLC2A3* | 1.328585588 | 0.4054402 | 3.276896537 | 0.001049548 | 0.004954002 |
| *WNT1* | 1.328252052 | 0.472107196 | 2.813454367 | 0.004901234 | 0.017683322 |
| *SLC31A2* | 1.327769764 | 0.250154937 | 5.307789575 | 1.11E-07 | 1.96E-06 |
| *MAPRE3* | 1.327067723 | 0.280210158 | 4.735972928 | 2.18E-06 | 2.65E-05 |
| *RASGEF1C* | 1.326988268 | 0.484534966 | 2.738684226 | 0.006168559 | 0.021404352 |
| *ASAH2* | 1.326660719 | 0.475765373 | 2.788476831 | 0.005295654 | 0.018882721 |
| *SLC26A6* | 1.326385954 | 0.229315807 | 5.784101712 | 7.29E-09 | 1.74E-07 |
| *RADX* | 1.326039917 | 0.355730388 | 3.727654318 | 0.00019327 | 0.001218158 |
| *HCN2* | 1.326031662 | 0.282477408 | 4.694292801 | 2.68E-06 | 3.16E-05 |
| *PTGER2* | 1.326001503 | 0.239436242 | 5.538015021 | 3.06E-08 | 6.24E-07 |
| *TMEM37* | 1.32593525 | 0.449330863 | 2.950910694 | 0.003168385 | 0.012390167 |
| *POTEJ* | 1.325212678 | 0.395508112 | 3.350658653 | 0.000806196 | 0.003991406 |
| *NR4A2* | 1.324164346 | 0.328991278 | 4.024922349 | 5.70E-05 | 0.000433183 |
| *RHBDF2* | 1.324102769 | 0.206762351 | 6.403983918 | 1.51E-10 | 5.57E-09 |
| *LRRC7* | 1.323914262 | 0.460093922 | 2.87748696 | 0.004008565 | 0.014994107 |
| *SESN3* | 1.323729471 | 0.270324995 | 4.896807533 | 9.74E-07 | 1.32E-05 |
| *FGF9* | 1.323470288 | 0.499866622 | 2.647646854 | 0.008105415 | 0.026783404 |
| *CDHR3* | 1.322902921 | 0.367727783 | 3.59750604 | 0.000321283 | 0.00186924 |
| *GFOD1* | 1.32264604 | 0.319989335 | 4.133406631 | 3.57E-05 | 0.000290045 |
| *LPAR6* | 1.322470129 | 0.446375348 | 2.962686301 | 0.003049672 | 0.012000052 |
| *S100A3* | 1.322235769 | 0.454614833 | 2.908474761 | 0.003631965 | 0.013833747 |
| *SMAP2* | 1.321402661 | 0.201360013 | 6.562388644 | 5.30E-11 | 2.12E-09 |
| *FGF2* | 1.317092123 | 0.479471543 | 2.746966201 | 0.006014933 | 0.020970124 |
| *ISG20* | 1.316158303 | 0.289568103 | 4.54524614 | 5.49E-06 | 5.86E-05 |
| *PXDC1* | 1.315694149 | 0.387180925 | 3.398137831 | 0.000678462 | 0.003461864 |
| *SOX6* | 1.315508636 | 0.506315674 | 2.598198522 | 0.00937143 | 0.030234608 |
| *EHD1* | 1.315458552 | 0.208461111 | 6.310330711 | 2.78E-10 | 9.70E-09 |
| *TNFRSF12A* | 1.313987248 | 0.397050708 | 3.309368857 | 0.000935066 | 0.004514326 |
| *MYD88* | 1.313939365 | 0.153677782 | 8.549963115 | 1.23E-17 | 1.79E-15 |
| *PDCD1LG2* | 1.313527507 | 0.397622517 | 3.303453526 | 0.000955018 | 0.004595235 |
| *CD247* | 1.31337241 | 0.312761583 | 4.199276639 | 2.68E-05 | 0.000225799 |
| *PXYLP1* | 1.312108306 | 0.221763071 | 5.91671236 | 3.28E-09 | 8.61E-08 |
| *MPP7* | 1.309651647 | 0.254608036 | 5.1437954 | 2.69E-07 | 4.28E-06 |
| *SCUBE1* | 1.309462607 | 0.52855171 | 2.477454113 | 0.013232342 | 0.039829136 |
| *TMC5* | 1.308821517 | 0.447827441 | 2.922602319 | 0.003471196 | 0.013341934 |
| *TIMP4* | 1.30850791 | 0.546861824 | 2.39275783 | 0.016722274 | 0.04809306 |
| *FERMT2* | 1.306708241 | 0.425938813 | 3.067830876 | 0.002156186 | 0.009023827 |
| *IFITM2* | 1.305723399 | 0.221914044 | 5.883915123 | 4.01E-09 | 1.02E-07 |
| *CLEC9A* | 1.30413271 | 0.537022831 | 2.428449286 | 0.015163546 | 0.044455394 |
| *NLRC5* | 1.303760202 | 0.179786332 | 7.251720325 | 4.12E-13 | 2.49E-11 |
| *SHISAL1* | 1.303662991 | 0.538112522 | 2.422658715 | 0.015407394 | 0.045035684 |
| *RGS16* | 1.30330818 | 0.438612986 | 2.971430899 | 0.002964156 | 0.011735032 |
| *ID2* | 1.302637005 | 0.296816923 | 4.388688456 | 1.14E-05 | 0.000109658 |
| *FTH1* | 1.302583864 | 0.263719819 | 4.939271806 | 7.84E-07 | 1.09E-05 |
| *XCL2* | 1.301917262 | 0.394481198 | 3.300327791 | 0.00096572 | 0.004639675 |
| *EGF* | 1.300720953 | 0.519786376 | 2.502414478 | 0.012334942 | 0.037639766 |
| *SH2D4B* | 1.29892456 | 0.378516843 | 3.43161628 | 0.000599996 | 0.00313527 |
| *CAPN2* | 1.298531561 | 0.26561485 | 4.888776206 | 1.01E-06 | 1.37E-05 |
| *NAP1L3* | 1.29601845 | 0.49062911 | 2.641544142 | 0.008252905 | 0.02717998 |
| *ERMN* | 1.295696273 | 0.383627951 | 3.377481411 | 0.000731529 | 0.003681748 |
| *TCF7L2* | 1.294863037 | 0.219930239 | 5.887608001 | 3.92E-09 | 1.00E-07 |
| *PRKCD* | 1.29485327 | 0.194036661 | 6.673240315 | 2.50E-11 | 1.07E-09 |
| *MTUS1* | 1.294397368 | 0.466214017 | 2.776401656 | 0.005496426 | 0.01945816 |
| *TLE3* | 1.29401071 | 0.241698471 | 5.353822498 | 8.61E-08 | 1.56E-06 |
| *NOTCH2* | 1.293393159 | 0.196191481 | 6.592504172 | 4.32E-11 | 1.77E-09 |
| *RRP12* | 1.29336108 | 0.175638981 | 7.363747335 | 1.79E-13 | 1.15E-11 |
| *OR7D2* | 1.293258699 | 0.435946456 | 2.966553993 | 0.003011575 | 0.011881158 |
| *GNG5P2* | 1.291769333 | 0.273962109 | 4.715138675 | 2.42E-06 | 2.90E-05 |
| *ATP6V0D1* | 1.290984976 | 0.1684525 | 7.663792304 | 1.81E-14 | 1.41E-12 |
| *TMEM117* | 1.29069838 | 0.402946389 | 3.203151625 | 0.001359324 | 0.006167509 |
| *SH2B2* | 1.290150858 | 0.267425398 | 4.824339303 | 1.40E-06 | 1.81E-05 |
| *DUSP2* | 1.290077784 | 0.350592888 | 3.679703229 | 0.000233506 | 0.001429758 |
| *FOXI1* | 1.289929312 | 0.507298015 | 2.542744646 | 0.010998558 | 0.034350011 |
| *CYTIP* | 1.289560442 | 0.26249303 | 4.912741651 | 8.98E-07 | 1.23E-05 |
| *PPIF* | 1.288750859 | 0.201042071 | 6.410354076 | 1.45E-10 | 5.34E-09 |
| *RCBTB2* | 1.288148392 | 0.241618396 | 5.331334098 | 9.75E-08 | 1.75E-06 |
| *CTSK* | 1.288041685 | 0.215924766 | 5.965233664 | 2.44E-09 | 6.65E-08 |
| *FCGR1B* | 1.287952556 | 0.34591782 | 3.723290562 | 0.000196643 | 0.001234615 |
| *PTX3* | 1.287909282 | 0.384229582 | 3.351926407 | 0.000802513 | 0.003978134 |
| *VEGFA* | 1.286590501 | 0.388183302 | 3.314389091 | 0.000918436 | 0.00445374 |
| *CD6* | 1.286486311 | 0.325499978 | 3.952339169 | 7.74E-05 | 0.000563682 |
| *LVRN* | 1.285133473 | 0.371197879 | 3.462125045 | 0.000535928 | 0.00285997 |
| *TP53I11* | 1.285047122 | 0.321476863 | 3.997323826 | 6.41E-05 | 0.000478407 |
| *SMPD3* | 1.284933263 | 0.349970541 | 3.671546925 | 0.000241087 | 0.001467945 |
| *RNF141* | 1.283799206 | 0.155654004 | 8.247775027 | 1.61E-16 | 1.85E-14 |
| *CPEB2* | 1.283291082 | 0.224129678 | 5.725663348 | 1.03E-08 | 2.37E-07 |
| *CDK5R1* | 1.282960556 | 0.263843822 | 4.86257569 | 1.16E-06 | 1.53E-05 |
| *GYG1* | 1.282903473 | 0.226678576 | 5.659570898 | 1.52E-08 | 3.33E-07 |
| *CRB3* | 1.282735678 | 0.359610274 | 3.567016216 | 0.000361069 | 0.002065415 |
| *ZNF300* | 1.282706883 | 0.504955839 | 2.540235764 | 0.011077777 | 0.034566594 |
| *ITIH1* | 1.282573718 | 0.434801132 | 2.949793878 | 0.00317986 | 0.012423807 |
| *CORO7* | 1.281642504 | 0.155048022 | 8.266100319 | 1.38E-16 | 1.62E-14 |
| *SMCO4* | 1.281209859 | 0.326234569 | 3.927265775 | 8.59E-05 | 0.000615059 |
| *SMIM10L2A* | 1.2802406 | 0.422049753 | 3.033387868 | 0.002418246 | 0.009918402 |
| *SLC12A9* | 1.277341126 | 0.240101989 | 5.319993942 | 1.04E-07 | 1.85E-06 |
| *DLGAP3* | 1.27680717 | 0.338098634 | 3.776433983 | 0.00015909 | 0.00103603 |
| *HLA-C* | 1.276704141 | 0.203538658 | 6.272538869 | 3.55E-10 | 1.20E-08 |
| *MIDN* | 1.276622537 | 0.220558903 | 5.78812517 | 7.12E-09 | 1.71E-07 |
| *F12* | 1.275973227 | 0.299760518 | 4.256642047 | 2.08E-05 | 0.000182226 |
| *SLC31A1* | 1.275394531 | 0.22332143 | 5.711026177 | 1.12E-08 | 2.55E-07 |
| *IRS2* | 1.275274792 | 0.306049518 | 4.166890379 | 3.09E-05 | 0.000254885 |
| *KYNU* | 1.273545271 | 0.379100038 | 3.359391042 | 0.000781144 | 0.003888036 |
| *CAMK2N1* | 1.271512095 | 0.337440148 | 3.768111481 | 0.000164487 | 0.001065264 |
| *KCNJ5* | 1.271339483 | 0.388811686 | 3.269807798 | 0.001076206 | 0.005059158 |
| *KLHL2* | 1.270648939 | 0.2274752 | 5.585878993 | 2.33E-08 | 4.92E-07 |
| *HS3ST1* | 1.270378019 | 0.491426714 | 2.585081321 | 0.009735603 | 0.031196503 |
| *TPRG1* | 1.269964275 | 0.439976365 | 2.88643749 | 0.003896301 | 0.014634708 |
| *KCTD17* | 1.26907296 | 0.297285078 | 4.26887542 | 1.96E-05 | 0.000174003 |
| *SEPTIN3* | 1.268188977 | 0.429803018 | 2.950628366 | 0.003171282 | 0.012396902 |
| *GRAMD1B* | 1.267445056 | 0.28068423 | 4.515554926 | 6.32E-06 | 6.62E-05 |
| *FHL2* | 1.266908179 | 0.431640119 | 2.935102935 | 0.003334371 | 0.012913241 |
| *PFKFB3* | 1.266710965 | 0.344463684 | 3.677342556 | 0.000235676 | 0.001440543 |
| *CCDC153* | 1.265953359 | 0.204092249 | 6.202848777 | 5.55E-10 | 1.75E-08 |
| *APBB1* | 1.265329316 | 0.360384726 | 3.511051451 | 0.000446338 | 0.002467996 |
| *HLA-E* | 1.265246094 | 0.188116238 | 6.725873895 | 1.75E-11 | 7.79E-10 |
| *MOB3A* | 1.26452708 | 0.171859413 | 7.357915724 | 1.87E-13 | 1.19E-11 |
| *CALY* | 1.263955094 | 0.498203809 | 2.537024146 | 0.011179924 | 0.034813268 |
| *M1AP* | 1.26344062 | 0.314917635 | 4.011971635 | 6.02E-05 | 0.000454046 |
| *SNX10* | 1.260770797 | 0.2176348 | 5.793056974 | 6.91E-09 | 1.66E-07 |
| *GIMAP6* | 1.26044041 | 0.338926854 | 3.718915735 | 0.00020008 | 0.001253649 |
| *NFASC* | 1.260302708 | 0.514171111 | 2.451134808 | 0.014240661 | 0.042297088 |
| *FAM83D* | 1.257946427 | 0.298287112 | 4.217233589 | 2.47E-05 | 0.000211197 |
| *SRC* | 1.257428164 | 0.306891516 | 4.097305062 | 4.18E-05 | 0.000330878 |
| *SLC2A4* | 1.257066051 | 0.523208451 | 2.40261037 | 0.016278521 | 0.047026179 |
| *RNF152* | 1.25637104 | 0.50461164 | 2.489778158 | 0.012782285 | 0.038743395 |
| *PTPRM* | 1.256135456 | 0.458933883 | 2.737072815 | 0.006198857 | 0.021492296 |
| *TCAF2* | 1.255948664 | 0.304708675 | 4.121801471 | 3.76E-05 | 0.000302495 |
| *ANKRD9* | 1.255020664 | 0.277939697 | 4.515442305 | 6.32E-06 | 6.62E-05 |
| *EDA* | 1.254461602 | 0.431362466 | 2.908138055 | 0.003635878 | 0.013842135 |
| *RGS19* | 1.253987075 | 0.165165432 | 7.592309494 | 3.14E-14 | 2.30E-12 |
| *TICAM1* | 1.253240107 | 0.184597241 | 6.789051134 | 1.13E-11 | 5.26E-10 |
| *FOXP3* | 1.253183303 | 0.311088338 | 4.028384062 | 5.62E-05 | 0.000427779 |
| *CD4* | 1.252860922 | 0.369032811 | 3.394985172 | 0.000686323 | 0.003495689 |
| *ZFP92* | 1.251122813 | 0.361920151 | 3.456902882 | 0.000546422 | 0.002908125 |
| *RNF149* | 1.249460283 | 0.205350361 | 6.084529274 | 1.17E-09 | 3.42E-08 |
| *KCNN3* | 1.249414406 | 0.345361119 | 3.6177043 | 0.000297228 | 0.001749543 |
| *PUDP* | 1.249176258 | 0.255238882 | 4.894145631 | 9.87E-07 | 1.34E-05 |
| *SEMA4A* | 1.249031408 | 0.24692375 | 5.058368851 | 4.23E-07 | 6.34E-06 |
| *CXCR5* | 1.248822906 | 0.357284565 | 3.495317254 | 0.000473499 | 0.002590576 |
| *DDX60L* | 1.247557755 | 0.190703222 | 6.541880852 | 6.07E-11 | 2.41E-09 |
| *CLDN5* | 1.246587854 | 0.459621721 | 2.712203966 | 0.006683745 | 0.022845737 |
| *IFITM1* | 1.246526276 | 0.334790912 | 3.72329783 | 0.000196637 | 0.001234615 |
| *MBOAT2* | 1.246041224 | 0.359265605 | 3.468300907 | 0.00052376 | 0.002809245 |
| *HJURP* | 1.245283545 | 0.32286266 | 3.857007019 | 0.000114784 | 0.000786091 |
| *ARHGAP26* | 1.245118889 | 0.202383293 | 6.152281017 | 7.64E-10 | 2.32E-08 |
| *KLF11* | 1.244796225 | 0.296316829 | 4.200896141 | 2.66E-05 | 0.000224489 |
| *ALOX15* | 1.244154536 | 0.472275735 | 2.634381665 | 0.008429068 | 0.02766804 |
| *SFN* | 1.243547366 | 0.373572305 | 3.328799666 | 0.000872211 | 0.004260256 |
| *TMEM88* | 1.243231682 | 0.210537121 | 5.905047412 | 3.53E-09 | 9.18E-08 |
| *SMOX* | 1.242790113 | 0.333015604 | 3.731927567 | 0.00019002 | 0.001201374 |
| *NEDD9* | 1.241994305 | 0.241813122 | 5.136174143 | 2.80E-07 | 4.44E-06 |
| *IL23A* | 1.241010947 | 0.2587356 | 4.796444502 | 1.62E-06 | 2.04E-05 |
| *PIANP* | 1.240828375 | 0.409796877 | 3.027910769 | 0.002462508 | 0.010074069 |
| *ADAMTS17* | 1.240750311 | 0.317541151 | 3.907368563 | 9.33E-05 | 0.000659018 |
| *PIM1* | 1.240694864 | 0.295543787 | 4.198006923 | 2.69E-05 | 0.000226646 |
| *FBLN2* | 1.239673141 | 0.471066174 | 2.631632688 | 0.008497568 | 0.027861077 |
| *LIF* | 1.239482948 | 0.45235029 | 2.740095395 | 0.006142135 | 0.02132903 |
| *SH3BP2* | 1.239296007 | 0.162922388 | 7.6066649 | 2.81E-14 | 2.08E-12 |
| *GADD45B* | 1.238733396 | 0.317330648 | 3.903604654 | 9.48E-05 | 0.000666679 |
| *PLAUR* | 1.238026293 | 0.379894242 | 3.258870906 | 0.001118566 | 0.005224924 |
| *RAB20* | 1.237918585 | 0.375421385 | 3.297410945 | 0.000975806 | 0.004675362 |
| *CD300LF* | 1.237431847 | 0.278508899 | 4.443060352 | 8.87E-06 | 8.83E-05 |
| *DNMBP* | 1.237109088 | 0.19757326 | 6.261520845 | 3.81E-10 | 1.27E-08 |
| *OR2B6* | 1.236766855 | 0.478910566 | 2.582458903 | 0.009809905 | 0.031397844 |
| *GNS* | 1.23637969 | 0.224392005 | 5.50990973 | 3.59E-08 | 7.20E-07 |
| *FRAT1* | 1.236339511 | 0.19074054 | 6.48178678 | 9.06E-11 | 3.47E-09 |
| *A3GALT2* | 1.235430118 | 0.332577056 | 3.71471842 | 0.00020343 | 0.001270782 |
| *SYNDIG1L* | 1.234440796 | 0.500189395 | 2.467946758 | 0.013589053 | 0.040716996 |
| *GPCPD1* | 1.234150166 | 0.181260285 | 6.808718011 | 9.85E-12 | 4.63E-10 |
| *COL16A1* | 1.232837712 | 0.439660944 | 2.804064654 | 0.005046278 | 0.018146654 |
| *SPINT1* | 1.231644701 | 0.345648622 | 3.563285441 | 0.000366242 | 0.002089719 |
| *INSC* | 1.23037586 | 0.43581053 | 2.823189843 | 0.004754841 | 0.017243583 |
| *SQOR* | 1.229709151 | 0.174472067 | 7.048172094 | 1.81E-12 | 9.71E-11 |
| *ERO1A* | 1.228939925 | 0.186038275 | 6.605844532 | 3.95E-11 | 1.63E-09 |
| *PRKAR2B* | 1.228141422 | 0.29435736 | 4.172280328 | 3.02E-05 | 0.00024984 |
| *TMIGD3* | 1.227539936 | 0.266884358 | 4.599519978 | 4.23E-06 | 4.69E-05 |
| *RNF157* | 1.226929852 | 0.328019025 | 3.74042284 | 0.000183711 | 0.001166611 |
| *CD109* | 1.225492209 | 0.437538504 | 2.800878544 | 0.00509637 | 0.018287313 |
| *SIDT2* | 1.224860264 | 0.191459448 | 6.397491886 | 1.58E-10 | 5.77E-09 |
| *APOE* | 1.224409777 | 0.494413101 | 2.476491368 | 0.013268083 | 0.039925328 |
| *RELT* | 1.223888291 | 0.245456203 | 4.986177892 | 6.16E-07 | 8.80E-06 |
| *GLRX* | 1.223397936 | 0.199116862 | 6.144120224 | 8.04E-10 | 2.43E-08 |
| *COLEC12* | 1.222417258 | 0.445574595 | 2.743462649 | 0.006079496 | 0.021148631 |
| *CCRL2* | 1.222083824 | 0.255817457 | 4.777171335 | 1.78E-06 | 2.22E-05 |
| *PLSCR1* | 1.221707166 | 0.216196553 | 5.650909541 | 1.60E-08 | 3.48E-07 |
| *AGAP1* | 1.220933972 | 0.383759717 | 3.181506339 | 0.001465113 | 0.006561916 |
| *SRPX* | 1.220195806 | 0.437006898 | 2.792166008 | 0.005235649 | 0.018704401 |
| *PDXK* | 1.219534043 | 0.223038195 | 5.467826017 | 4.56E-08 | 8.89E-07 |
| *MTRNR2L10* | 1.217536021 | 0.400234499 | 3.042056651 | 0.002349677 | 0.009681817 |
| *FRAS1* | 1.217251703 | 0.466237256 | 2.610798873 | 0.0090331 | 0.029316365 |
| *OSBP2* | 1.216881995 | 0.43487159 | 2.798255902 | 0.005137939 | 0.018418094 |
| *FKBP15* | 1.216632069 | 0.185873118 | 6.5454977 | 5.93E-11 | 2.36E-09 |
| *DPF3* | 1.215967457 | 0.338703238 | 3.590067415 | 0.000330592 | 0.001916359 |
| *HECW2* | 1.215111911 | 0.291294682 | 4.171418108 | 3.03E-05 | 0.000250591 |
| *EHBP1L1* | 1.214699908 | 0.145231463 | 8.363889487 | 6.07E-17 | 7.65E-15 |
| *ABCA10* | 1.21345294 | 0.265549382 | 4.569594284 | 4.89E-06 | 5.30E-05 |
| *ACOT9* | 1.213007775 | 0.192160033 | 6.312487347 | 2.75E-10 | 9.57E-09 |
| *NFKBID* | 1.212944034 | 0.235659675 | 5.147015647 | 2.65E-07 | 4.22E-06 |
| *ELL* | 1.210194425 | 0.214768492 | 5.634878808 | 1.75E-08 | 3.79E-07 |
| *S100A10* | 1.209924403 | 0.359996123 | 3.360937316 | 0.000776785 | 0.003872424 |
| *LGI4* | 1.209489908 | 0.280246969 | 4.315800137 | 1.59E-05 | 0.000145538 |
| *GPSM3* | 1.20892216 | 0.147254806 | 8.209729729 | 2.22E-16 | 2.44E-14 |
| *SLC44A2* | 1.208512826 | 0.217733948 | 5.550410658 | 2.85E-08 | 5.88E-07 |
| *KCNA3* | 1.208502294 | 0.356721512 | 3.387803236 | 0.000704548 | 0.003566795 |
| *NCEH1* | 1.207780112 | 0.246440719 | 4.900895105 | 9.54E-07 | 1.30E-05 |
| *NUGGC* | 1.207363835 | 0.329983942 | 3.658856332 | 0.000253343 | 0.001529627 |
| *NAAA* | 1.206744483 | 0.247316394 | 4.879355008 | 1.06E-06 | 1.43E-05 |
| *MTF1* | 1.204952544 | 0.175033339 | 6.884131636 | 5.81E-12 | 2.87E-10 |
| *SBF2* | 1.203144244 | 0.226342571 | 5.31558973 | 1.06E-07 | 1.88E-06 |
| *EVI2A* | 1.20126662 | 0.201845947 | 5.951403225 | 2.66E-09 | 7.16E-08 |
| *PLEKHD1* | 1.200908707 | 0.390488367 | 3.075402003 | 0.00210219 | 0.00884227 |
| *MXD3* | 1.19973302 | 0.179566259 | 6.68128315 | 2.37E-11 | 1.02E-09 |
| *RASL11A* | 1.199098613 | 0.291640214 | 4.111568141 | 3.93E-05 | 0.000313909 |
| *MAPK8IP1* | 1.198598808 | 0.282088743 | 4.249013258 | 2.15E-05 | 0.0001874 |
| *SULT1A1* | 1.1979452 | 0.219038253 | 5.469114099 | 4.52E-08 | 8.83E-07 |
| *KALRN* | 1.196866283 | 0.398475833 | 3.003610722 | 0.002667964 | 0.010749279 |
| *ANK1* | 1.196579972 | 0.480922458 | 2.488093357 | 0.012843001 | 0.038882106 |
| *CORO1A* | 1.196253256 | 0.143325955 | 8.346382591 | 7.04E-17 | 8.74E-15 |
| *TAGAP* | 1.195957865 | 0.236591659 | 5.054945174 | 4.31E-07 | 6.44E-06 |
| *OGFR* | 1.194138294 | 0.177010688 | 6.746136681 | 1.52E-11 | 6.86E-10 |
| *PLEKHA1* | 1.193470972 | 0.256303259 | 4.656479894 | 3.22E-06 | 3.71E-05 |
| *VPS37C* | 1.192952746 | 0.19794298 | 6.026749448 | 1.67E-09 | 4.71E-08 |
| *CSGALNACT2* | 1.192888861 | 0.227237083 | 5.249534305 | 1.52E-07 | 2.59E-06 |
| *APOBR* | 1.19192877 | 0.196290574 | 6.072266971 | 1.26E-09 | 3.67E-08 |
| *KCNC3* | 1.191925143 | 0.485210776 | 2.45651004 | 0.014029388 | 0.041797811 |
| *FBXL5* | 1.191824221 | 0.156223376 | 7.628974952 | 2.37E-14 | 1.80E-12 |
| *LOXL3* | 1.191259718 | 0.219245951 | 5.43343999 | 5.53E-08 | 1.05E-06 |
| *GGT1* | 1.19123466 | 0.283851814 | 4.196677987 | 2.71E-05 | 0.00022766 |
| *IL32* | 1.189908587 | 0.3144817 | 3.783713293 | 0.000154506 | 0.001009919 |
| *CELSR2* | 1.189502741 | 0.265556361 | 4.479285438 | 7.49E-06 | 7.67E-05 |
| *TIGIT* | 1.189199796 | 0.342664739 | 3.47044694 | 0.000519593 | 0.002791622 |
| *LTB* | 1.18889706 | 0.275478075 | 4.315759287 | 1.59E-05 | 0.000145538 |
| *PTPRE* | 1.188148354 | 0.2906423 | 4.088009061 | 4.35E-05 | 0.000343045 |
| *HLA-DMA* | 1.187776525 | 0.268722885 | 4.420079544 | 9.87E-06 | 9.70E-05 |
| *GPR18* | 1.187640213 | 0.34360742 | 3.456386977 | 0.000547469 | 0.002912298 |
| *FAM107B* | 1.187588209 | 0.24858086 | 4.777472444 | 1.78E-06 | 2.22E-05 |
| *SOWAHD* | 1.186495171 | 0.264808632 | 4.480575886 | 7.44E-06 | 7.63E-05 |
| *ASB2* | 1.185632089 | 0.337186759 | 3.516247471 | 0.000437693 | 0.002426126 |
| *GNPDA1* | 1.185408487 | 0.281440539 | 4.211932268 | 2.53E-05 | 0.000215288 |
| *RNF19B* | 1.184001199 | 0.238265344 | 4.969254782 | 6.72E-07 | 9.51E-06 |
| *AGPAT3* | 1.183356049 | 0.218956344 | 5.404529638 | 6.50E-08 | 1.21E-06 |
| *ADGRV1* | 1.183328772 | 0.407731158 | 2.902227975 | 0.003705188 | 0.014042397 |
| *PLXNA1* | 1.1831742 | 0.347181138 | 3.407944935 | 0.000654541 | 0.003360388 |
| *IER5L* | 1.183001286 | 0.29188845 | 4.05292256 | 5.06E-05 | 0.000390834 |
| *LDB2* | 1.182043663 | 0.486257842 | 2.430898917 | 0.015061416 | 0.044199887 |
| *MCC* | 1.181433067 | 0.337655936 | 3.498925807 | 0.000467137 | 0.002564633 |
| *CEACAM21* | 1.181207391 | 0.31847379 | 3.708962643 | 0.00020811 | 0.001294773 |
| *PLCB4* | 1.181142164 | 0.478062186 | 2.470687283 | 0.013485369 | 0.040436945 |
| *CSTB* | 1.179972991 | 0.253797848 | 4.649263195 | 3.33E-06 | 3.82E-05 |
| *PDZK1IP1* | 1.179401382 | 0.485162153 | 2.430942675 | 0.015059597 | 0.044198647 |
| *ACVR1C* | 1.178358582 | 0.318013131 | 3.705377128 | 0.000211076 | 0.001309248 |
| *MAP3K9* | 1.177362353 | 0.29468719 | 3.995295326 | 6.46E-05 | 0.000481613 |
| *TNFSF8* | 1.177036996 | 0.310646903 | 3.78898674 | 0.000151263 | 0.000991999 |
| *DYNLT4* | 1.176711402 | 0.266956362 | 4.407879226 | 1.04E-05 | 0.000101834 |
| *CABP7* | 1.176319473 | 0.379245002 | 3.101740215 | 0.001923867 | 0.008213343 |
| *USHBP1* | 1.17607719 | 0.392263576 | 2.998180974 | 0.002715963 | 0.010901606 |
| *SDR42E2* | 1.174523927 | 0.353020518 | 3.327069868 | 0.000877644 | 0.004284146 |
| *NFKBIE* | 1.17413874 | 0.186954632 | 6.280340464 | 3.38E-10 | 1.14E-08 |
| *ALDH1A1* | 1.173940275 | 0.469234005 | 2.501822681 | 0.012355579 | 0.03767427 |
| *FAM241B* | 1.173266953 | 0.393797147 | 2.97936885 | 0.002888428 | 0.011484061 |
| *ZBTB7A* | 1.172661415 | 0.183644858 | 6.385484611 | 1.71E-10 | 6.19E-09 |
| *SIPA1L1* | 1.17256375 | 0.264268978 | 4.437008675 | 9.12E-06 | 9.06E-05 |
| *PLCH2* | 1.172330568 | 0.330776535 | 3.54417694 | 0.000393841 | 0.002219588 |
| *SERTAD3* | 1.171933507 | 0.178589005 | 6.562181742 | 5.30E-11 | 2.12E-09 |
| *AQP8* | 1.171483983 | 0.449732762 | 2.604844658 | 0.009191592 | 0.029763609 |
| *NAV1* | 1.170881802 | 0.334055825 | 3.505048303 | 0.000456524 | 0.002514353 |
| *SYDE2* | 1.170294777 | 0.446965165 | 2.618313165 | 0.008836567 | 0.028778843 |
| *CPNE9* | 1.169719663 | 0.197316505 | 5.928138972 | 3.06E-09 | 8.12E-08 |
| *MCF2* | 1.169632826 | 0.262085417 | 4.462792469 | 8.09E-06 | 8.19E-05 |
| *GLTP* | 1.169333328 | 0.146910434 | 7.959498148 | 1.73E-15 | 1.66E-13 |
| *NOTCH4* | 1.16730379 | 0.287878886 | 4.054843366 | 5.02E-05 | 0.000388387 |
| *BCAS1* | 1.166560508 | 0.473669786 | 2.46281385 | 0.013785147 | 0.041187598 |
| *HSBP1L1* | 1.166219384 | 0.288416546 | 4.043524551 | 5.27E-05 | 0.000404074 |
| *P2RY6* | 1.166138629 | 0.365595743 | 3.189694221 | 0.001424234 | 0.006416046 |
| *H1-0* | 1.165667622 | 0.420845816 | 2.769821102 | 0.005608709 | 0.01978706 |
| *SDS* | 1.163520753 | 0.420944 | 2.76407492 | 0.005708443 | 0.020080728 |
| *DPYS* | 1.16307005 | 0.479099944 | 2.427614662 | 0.015198482 | 0.044531945 |
| *H2AJ* | 1.162257863 | 0.270253872 | 4.300615024 | 1.70E-05 | 0.000154184 |
| *CHMP4B* | 1.161619207 | 0.161912212 | 7.174376754 | 7.26E-13 | 4.17E-11 |
| *GCNT4* | 1.161514199 | 0.368008206 | 3.156218202 | 0.001598292 | 0.007045244 |
| *CFAP58* | 1.160829852 | 0.23926938 | 4.851560404 | 1.22E-06 | 1.61E-05 |
| *RBM44* | 1.160156848 | 0.275875252 | 4.205367605 | 2.61E-05 | 0.000220803 |
| *HCAR2* | 1.159880048 | 0.368757657 | 3.145372106 | 0.001658757 | 0.007268772 |
| *VAV2* | 1.159158005 | 0.266572913 | 4.348371299 | 1.37E-05 | 0.000128567 |
| *ATP6V1C2* | 1.158453929 | 0.39259934 | 2.950728159 | 0.003170258 | 0.012394429 |
| *KLF2* | 1.158446091 | 0.265584114 | 4.361880209 | 1.29E-05 | 0.000121888 |
| *RNF166* | 1.157726237 | 0.140315479 | 8.250880438 | 1.57E-16 | 1.82E-14 |
| *MAP3K7CL* | 1.157659408 | 0.305922241 | 3.784162287 | 0.000154227 | 0.001008514 |
| *PREX1* | 1.157306517 | 0.146484821 | 7.900521766 | 2.78E-15 | 2.55E-13 |
| *IL17RA* | 1.156479551 | 0.280608136 | 4.121332933 | 3.77E-05 | 0.00030288 |
| *ZC3H12D* | 1.15608652 | 0.209080157 | 5.52939377 | 3.21E-08 | 6.52E-07 |
| *TGM1* | 1.155740766 | 0.276053078 | 4.186661395 | 2.83E-05 | 0.000236574 |
| *RASSF3* | 1.154741294 | 0.198015419 | 5.83157261 | 5.49E-09 | 1.36E-07 |
| *SLC9A8* | 1.154445813 | 0.192664124 | 5.992012359 | 2.07E-09 | 5.75E-08 |
| *CNIH4* | 1.153946258 | 0.209000533 | 5.521259884 | 3.37E-08 | 6.80E-07 |
| *ALDH3B1* | 1.153842431 | 0.191929839 | 6.011792839 | 1.83E-09 | 5.12E-08 |
| *DPYSL4* | 1.152846021 | 0.465210212 | 2.478118474 | 0.013207728 | 0.039766392 |
| *TGFBR3* | 1.150967732 | 0.33984233 | 3.386769773 | 0.000707207 | 0.003577968 |
| *CEMP1* | 1.150267879 | 0.359353884 | 3.200933484 | 0.001369832 | 0.006208318 |
| *MYH10* | 1.147228831 | 0.47804891 | 2.39981476 | 0.01640337 | 0.04731776 |
| *NAV2* | 1.146938117 | 0.414207016 | 2.768997321 | 0.00562291 | 0.019826112 |
| *OTX1* | 1.145652538 | 0.476962954 | 2.40197384 | 0.016306875 | 0.047092668 |
| *PITPNC1* | 1.145385472 | 0.186377075 | 6.14552767 | 7.97E-10 | 2.41E-08 |
| *AP5B1* | 1.144845617 | 0.242230223 | 4.726270746 | 2.29E-06 | 2.77E-05 |
| *SLC30A1* | 1.142258775 | 0.229468127 | 4.977853736 | 6.43E-07 | 9.14E-06 |
| *RGMB* | 1.141581863 | 0.402792932 | 2.834165579 | 0.004594552 | 0.016758283 |
| *SMARCD3* | 1.141490433 | 0.390652581 | 2.922009193 | 0.003477813 | 0.0133625 |
| *IRF6* | 1.140540136 | 0.389197231 | 2.930493967 | 0.003384236 | 0.013074367 |
| *ASAH2B* | 1.139620817 | 0.39625456 | 2.875981582 | 0.004027732 | 0.015049798 |
| *GRK6* | 1.138458639 | 0.159234567 | 7.149569713 | 8.71E-13 | 4.92E-11 |
| *ZDHHC7* | 1.137590324 | 0.166358193 | 6.83819836 | 8.02E-12 | 3.83E-10 |
| *CD8A* | 1.137567058 | 0.349383168 | 3.255929768 | 0.001130217 | 0.005271571 |
| *FOSL1* | 1.137548041 | 0.338524205 | 3.360315227 | 0.000778536 | 0.003877491 |
| *TMEM127* | 1.137241608 | 0.154398294 | 7.365635846 | 1.76E-13 | 1.14E-11 |
| *DUSP16* | 1.137193014 | 0.318729029 | 3.567899092 | 0.000359855 | 0.002060704 |
| *HPN* | 1.137189494 | 0.424138183 | 2.681176889 | 0.007336372 | 0.024680149 |
| *SPIRE1* | 1.137076987 | 0.341429419 | 3.330342742 | 0.000867391 | 0.004239985 |
| *SLC45A4* | 1.136720735 | 0.22983176 | 4.945881872 | 7.58E-07 | 1.06E-05 |
| *ACTB* | 1.136299164 | 0.153856242 | 7.385460283 | 1.52E-13 | 9.88E-12 |
| *ERRFI1* | 1.135970058 | 0.452331508 | 2.511366193 | 0.012026487 | 0.036926954 |
| *CPEB4* | 1.135256959 | 0.269441002 | 4.213378619 | 2.52E-05 | 0.000214143 |
| *RFPL2* | 1.134722556 | 0.441247461 | 2.571623989 | 0.010122276 | 0.032155622 |
| *ARAP1* | 1.134071332 | 0.156177827 | 7.261410622 | 3.83E-13 | 2.32E-11 |
| *XKR8* | 1.133937632 | 0.180576468 | 6.279542636 | 3.40E-10 | 1.15E-08 |
| *LYN* | 1.133666743 | 0.186867791 | 6.066678141 | 1.31E-09 | 3.78E-08 |
| *HAVCR1* | 1.133499678 | 0.326775306 | 3.468743375 | 0.000522899 | 0.002805573 |
| *BIRC3* | 1.133410613 | 0.225018388 | 5.036968859 | 4.73E-07 | 7.00E-06 |
| *PRKCA* | 1.132611466 | 0.337589717 | 3.354993971 | 0.000793667 | 0.003942308 |
| *C3orf52* | 1.131724097 | 0.410675567 | 2.755761943 | 0.00585556 | 0.020484429 |
| *PEA15* | 1.131714999 | 0.196629472 | 5.755571582 | 8.63E-09 | 2.02E-07 |
| *MCOLN1* | 1.130411886 | 0.178208537 | 6.343197158 | 2.25E-10 | 8.01E-09 |
| *SEMA4B* | 1.129973598 | 0.209364928 | 5.397148445 | 6.77E-08 | 1.25E-06 |
| *NUMB* | 1.128404289 | 0.153341046 | 7.358788246 | 1.86E-13 | 1.19E-11 |
| *GPR183* | 1.127970621 | 0.386641281 | 2.917356932 | 0.003530116 | 0.013515885 |
| *EVA1C* | 1.127931122 | 0.300762929 | 3.750233197 | 0.00017667 | 0.001128465 |
| *RENBP* | 1.127288561 | 0.195087538 | 5.778372998 | 7.54E-09 | 1.79E-07 |
| *SLC5A10* | 1.127276188 | 0.474186894 | 2.377282465 | 0.017440727 | 0.049671943 |
| *B4GALT5* | 1.126612374 | 0.244339801 | 4.610842637 | 4.01E-06 | 4.48E-05 |
| *CD3E* | 1.126174968 | 0.29204522 | 3.856166414 | 0.000115179 | 0.000788007 |
| *ASAH1* | 1.126002406 | 0.184450617 | 6.104628035 | 1.03E-09 | 3.04E-08 |
| *LIMK2* | 1.125371484 | 0.24291599 | 4.632760001 | 3.61E-06 | 4.10E-05 |
| *STRIP2* | 1.125074239 | 0.352692823 | 3.189955016 | 0.001422949 | 0.006411171 |
| *MAML2* | 1.122648857 | 0.293974751 | 3.818861498 | 0.000134069 | 0.000895188 |
| *CSF2RA* | 1.122367517 | 0.2504306 | 4.481750695 | 7.40E-06 | 7.60E-05 |
| *TMEM120A* | 1.122243363 | 0.218602308 | 5.133721471 | 2.84E-07 | 4.48E-06 |
| *CPNE5* | 1.122204479 | 0.330050345 | 3.400100912 | 0.00067361 | 0.003443765 |
| *SLC25A34* | 1.12192613 | 0.24333937 | 4.610540951 | 4.02E-06 | 4.49E-05 |
| *SLC16A11* | 1.121020461 | 0.431619875 | 2.597240129 | 0.00939762 | 0.030300577 |
| *C20orf144* | 1.119142896 | 0.367623919 | 3.044260281 | 0.002332532 | 0.009625687 |
| *GPT* | 1.118161622 | 0.357020351 | 3.131926847 | 0.001736631 | 0.00754423 |
| *CD276* | 1.118064583 | 0.457485828 | 2.443932717 | 0.014528136 | 0.042973525 |
| *GRAP2* | 1.116335591 | 0.343137227 | 3.253321127 | 0.001140645 | 0.005306917 |
| *IL10RB* | 1.115221351 | 0.184305251 | 6.050947238 | 1.44E-09 | 4.10E-08 |
| *RNF13* | 1.112289563 | 0.158612172 | 7.012636852 | 2.34E-12 | 1.23E-10 |
| *DAPP1* | 1.111891594 | 0.20994283 | 5.2961637 | 1.18E-07 | 2.07E-06 |
| *P4HA3* | 1.111469823 | 0.378712263 | 2.93486621 | 0.003336915 | 0.012921516 |
| *FCRLA* | 1.111282915 | 0.446918176 | 2.486546698 | 0.012898964 | 0.038999962 |
| *TP53INP2* | 1.111179654 | 0.332790852 | 3.338972959 | 0.000840887 | 0.004132123 |
| *LRP10* | 1.110440487 | 0.184540821 | 6.017316281 | 1.77E-09 | 4.97E-08 |
| *ACTA1* | 1.109416083 | 0.290091975 | 3.82435979 | 0.000131112 | 0.000878659 |
| *SDCBP* | 1.107744915 | 0.200826731 | 5.515923651 | 3.47E-08 | 6.98E-07 |
| *ADGRA2* | 1.106523866 | 0.423203897 | 2.614635344 | 0.008932276 | 0.029039355 |
| *CD27* | 1.106425355 | 0.34140252 | 3.240823631 | 0.001191849 | 0.005516075 |
| *GBP2* | 1.105878014 | 0.235812342 | 4.689652815 | 2.74E-06 | 3.22E-05 |
| *EAF1* | 1.105867791 | 0.250101156 | 4.421682052 | 9.79E-06 | 9.64E-05 |
| *CLCN5* | 1.105146557 | 0.219864165 | 5.02649697 | 5.00E-07 | 7.37E-06 |
| *AHR* | 1.105102087 | 0.292196736 | 3.782048016 | 0.000155543 | 0.001014605 |
| *STARD8* | 1.104477056 | 0.293664116 | 3.761021511 | 0.000169221 | 0.001088571 |
| *DOK3* | 1.104182516 | 0.208229799 | 5.302711328 | 1.14E-07 | 2.00E-06 |
| *MAP2K6* | 1.10390991 | 0.227800086 | 4.845959154 | 1.26E-06 | 1.65E-05 |
| *FLT4* | 1.103563861 | 0.381744312 | 2.890845588 | 0.003842068 | 0.014473608 |
| *KIAA0040* | 1.102969114 | 0.236970804 | 4.654451501 | 3.25E-06 | 3.74E-05 |
| *ITPK1* | 1.101174198 | 0.131823371 | 8.35340643 | 6.63E-17 | 8.30E-15 |
| *INAVA* | 1.099677681 | 0.445885998 | 2.46627543 | 0.013652631 | 0.040868811 |
| *ARHGAP27* | 1.099528971 | 0.163899804 | 6.70854353 | 1.97E-11 | 8.65E-10 |
| *DDR1* | 1.099349655 | 0.355349036 | 3.093717846 | 0.001976654 | 0.008403566 |
| *MAFF* | 1.098560104 | 0.385398133 | 2.850455175 | 0.00436567 | 0.016069728 |
| *TSPAN16* | 1.098369254 | 0.382616608 | 2.870678457 | 0.004095919 | 0.015250579 |
| *LPCAT1* | 1.097692057 | 0.198138108 | 5.540035021 | 3.02E-08 | 6.19E-07 |
| *PI16* | 1.097160371 | 0.393833814 | 2.785846038 | 0.005338822 | 0.019010771 |
| *SH3TC1* | 1.09631574 | 0.249446938 | 4.394985763 | 1.11E-05 | 0.000107178 |
| *SGMS1* | 1.096268206 | 0.16126138 | 6.798082735 | 1.06E-11 | 4.96E-10 |
| *RBM38* | 1.09596822 | 0.269577447 | 4.06550412 | 4.79E-05 | 0.000373059 |
| *MERTK* | 1.0958894 | 0.293625745 | 3.732266051 | 0.000189765 | 0.001200239 |
| *CBX6* | 1.095805545 | 0.195932269 | 5.5927773 | 2.23E-08 | 4.73E-07 |
| *TPD52L2* | 1.095728442 | 0.160226352 | 6.838628137 | 8.00E-12 | 3.83E-10 |
| *PLD3* | 1.095124395 | 0.251751692 | 4.350018013 | 1.36E-05 | 0.000127756 |
| *SLC49A4* | 1.094691134 | 0.232204317 | 4.714344455 | 2.42E-06 | 2.91E-05 |
| *RASGRP4* | 1.093206043 | 0.201502994 | 5.42525955 | 5.79E-08 | 1.10E-06 |
| *EVI5* | 1.092761186 | 0.22227215 | 4.916320767 | 8.82E-07 | 1.21E-05 |
| *GNG10* | 1.090585786 | 0.278208239 | 3.920034102 | 8.85E-05 | 0.000630957 |
| *MAP3K14* | 1.090505927 | 0.178361029 | 6.114036979 | 9.71E-10 | 2.89E-08 |
| *PDLIM5* | 1.088522848 | 0.199313391 | 5.461363347 | 4.72E-08 | 9.18E-07 |
| *GPR156* | 1.088007475 | 0.373934097 | 2.909623602 | 0.003618643 | 0.013793107 |
| *AIF1* | 1.087419599 | 0.192669697 | 5.643957604 | 1.66E-08 | 3.61E-07 |
| *SIRPG* | 1.087264627 | 0.33107324 | 3.284060735 | 0.001023229 | 0.004855111 |
| *TMSB4X* | 1.085859655 | 0.174475606 | 6.223561455 | 4.86E-10 | 1.56E-08 |
| *HHLA2* | 1.08531692 | 0.334979896 | 3.239946436 | 0.001195522 | 0.005528108 |
| *DIPK1A* | 1.084549751 | 0.316564678 | 3.425997363 | 0.000612546 | 0.003189795 |
| *HECA* | 1.084327421 | 0.187686636 | 5.777328876 | 7.59E-09 | 1.80E-07 |
| *GPR132* | 1.083140456 | 0.242724071 | 4.462435273 | 8.10E-06 | 8.20E-05 |
| *RHOG* | 1.082595905 | 0.15795379 | 6.853877355 | 7.19E-12 | 3.48E-10 |
| *CLCN4* | 1.080273966 | 0.417645271 | 2.586582541 | 0.009693295 | 0.031086106 |
| *CYSRT1* | 1.079578134 | 0.291486231 | 3.703701984 | 0.000212476 | 0.001316269 |
| *CXCR6* | 1.078923608 | 0.373700401 | 2.887135272 | 0.00388767 | 0.014607489 |
| *OAT* | 1.078641395 | 0.178221915 | 6.052237711 | 1.43E-09 | 4.07E-08 |
| *BCAR3* | 1.078167804 | 0.401729537 | 2.683815117 | 0.007278735 | 0.024522735 |
| *GAA* | 1.077955184 | 0.251070807 | 4.293430984 | 1.76E-05 | 0.0001584 |
| *PAG1* | 1.07746126 | 0.24595391 | 4.380744587 | 1.18E-05 | 0.000113183 |
| *IL2RB* | 1.076402049 | 0.327323832 | 3.288492754 | 0.001007254 | 0.00479411 |
| *ARPC1B* | 1.07577451 | 0.166755909 | 6.451192756 | 1.11E-10 | 4.17E-09 |
| *CLIP4* | 1.075360305 | 0.305584061 | 3.519032712 | 0.000433123 | 0.002404586 |
| *NXPH4* | 1.074734082 | 0.395071699 | 2.72035199 | 0.006521246 | 0.02239737 |
| *C14orf132* | 1.07381445 | 0.405338816 | 2.649177445 | 0.008068795 | 0.026693049 |
| *HBEGF* | 1.072180177 | 0.450056584 | 2.382323059 | 0.017203797 | 0.049130849 |
| *KLF10* | 1.071673218 | 0.240941023 | 4.447865301 | 8.67E-06 | 8.67E-05 |
| *PRNP* | 1.071392012 | 0.204724413 | 5.233337809 | 1.66E-07 | 2.80E-06 |
| *AMPD3* | 1.070812474 | 0.237462902 | 4.509388472 | 6.50E-06 | 6.79E-05 |
| *ARPIN* | 1.070663285 | 0.398595381 | 2.686090544 | 0.00722935 | 0.024387499 |
| *LRP3* | 1.069667246 | 0.296261456 | 3.6105515 | 0.000305547 | 0.001790851 |
| *FAM177B* | 1.069493275 | 0.259210838 | 4.125958943 | 3.69E-05 | 0.000297687 |
| *G6PD* | 1.069360183 | 0.179423487 | 5.959978819 | 2.52E-09 | 6.84E-08 |
| *COL4A3* | 1.068973004 | 0.388797761 | 2.749432 | 0.005969865 | 0.020826762 |
| *SRD5A1* | 1.067884184 | 0.212690504 | 5.020836202 | 5.14E-07 | 7.55E-06 |
| *MTCH1* | 1.066441169 | 0.148639432 | 7.17468542 | 7.25E-13 | 4.17E-11 |
| *CSF1R* | 1.065835726 | 0.335725197 | 3.174726636 | 0.001499777 | 0.006689721 |
| *DDIT4* | 1.065440178 | 0.399900274 | 2.664264685 | 0.007715683 | 0.025721109 |
| *COL18A1* | 1.065388108 | 0.285575519 | 3.730670298 | 0.000190971 | 0.0012057 |
| *ZNF831* | 1.065016144 | 0.277818481 | 3.833496385 | 0.000126335 | 0.000852719 |
| *CYRIB* | 1.063186308 | 0.144682502 | 7.348409726 | 2.01E-13 | 1.28E-11 |
| *BLOC1S1* | 1.062448507 | 0.142841548 | 7.437951493 | 1.02E-13 | 6.87E-12 |
| *SAMD8* | 1.062276415 | 0.196482957 | 5.406455762 | 6.43E-08 | 1.20E-06 |
| *OPTN* | 1.061960153 | 0.295832615 | 3.589733173 | 0.000331017 | 0.001918466 |
| *LONRF3* | 1.061366756 | 0.370122722 | 2.867607667 | 0.004135881 | 0.015363229 |
| *PARP12* | 1.06135698 | 0.168483622 | 6.299466788 | 2.99E-10 | 1.02E-08 |
| *ADAM15* | 1.061147623 | 0.187344557 | 5.664149739 | 1.48E-08 | 3.26E-07 |
| *CTRL* | 1.05974593 | 0.308446443 | 3.435753445 | 0.000590908 | 0.003094425 |
| *PIM2* | 1.058114421 | 0.245701302 | 4.306507187 | 1.66E-05 | 0.00015068 |
| *ZNF318* | 1.057832887 | 0.167180714 | 6.327481579 | 2.49E-10 | 8.77E-09 |
| *NXN* | 1.057789183 | 0.441788223 | 2.394335401 | 0.016650515 | 0.047924782 |
| *CPQ* | 1.057752828 | 0.193673905 | 5.461514442 | 4.72E-08 | 9.18E-07 |
| *TJP3* | 1.057693874 | 0.306509402 | 3.450771384 | 0.000558987 | 0.002960346 |
| *CBX7* | 1.057216125 | 0.210043189 | 5.033327335 | 4.82E-07 | 7.13E-06 |
| *SLC35F6* | 1.056903832 | 0.200668503 | 5.266914406 | 1.39E-07 | 2.39E-06 |
| *DNAJC5* | 1.05618495 | 0.164091422 | 6.436564069 | 1.22E-10 | 4.55E-09 |
| *TOX2* | 1.055830811 | 0.408843327 | 2.582482682 | 0.009809229 | 0.031397844 |
| *MYBPC2* | 1.055458259 | 0.338045707 | 3.122235358 | 0.001794834 | 0.007760815 |
| *WDFY3* | 1.054468754 | 0.284203893 | 3.71025444 | 0.000207051 | 0.00128973 |
| *ASMT* | 1.053172919 | 0.353920272 | 2.975734937 | 0.002922874 | 0.011604978 |
| *MCL1* | 1.052887244 | 0.186539758 | 5.644304752 | 1.66E-08 | 3.61E-07 |
| *RRM2* | 1.052625622 | 0.300626069 | 3.501444922 | 0.000462743 | 0.002543828 |
| *FOS* | 1.052258085 | 0.243329727 | 4.324412386 | 1.53E-05 | 0.000140739 |
| *YOD1* | 1.051405883 | 0.274175702 | 3.834788704 | 0.000125672 | 0.000848972 |
| *JAG2* | 1.051211048 | 0.306724102 | 3.427220232 | 0.000609794 | 0.003178602 |
| *IL21R* | 1.050553744 | 0.310494815 | 3.383482409 | 0.000715728 | 0.003612994 |
| *RIN1* | 1.04980576 | 0.323750245 | 3.242640819 | 0.001184274 | 0.005486518 |
| *NQO2* | 1.049453905 | 0.183075441 | 5.732357662 | 9.90E-09 | 2.29E-07 |
| *EIF5A2* | 1.0489994 | 0.313625785 | 3.344748584 | 0.000823572 | 0.004061535 |
| *CCDC126* | 1.046358415 | 0.177886103 | 5.882181901 | 4.05E-09 | 1.03E-07 |
| *ABTB1* | 1.045835187 | 0.167469951 | 6.244912482 | 4.24E-10 | 1.39E-08 |
| *SSH2* | 1.045178806 | 0.173917303 | 6.009630962 | 1.86E-09 | 5.19E-08 |
| *SPATC1* | 1.044773873 | 0.402297624 | 2.597017259 | 0.00940372 | 0.030314069 |
| *FYN* | 1.044709225 | 0.203080596 | 5.144308441 | 2.69E-07 | 4.27E-06 |
| *ME1* | 1.043559284 | 0.392264642 | 2.660345014 | 0.007806064 | 0.025964986 |
| *TIMP1* | 1.04306161 | 0.298387783 | 3.495657896 | 0.000472895 | 0.002588613 |
| *RABGEF1* | 1.04271563 | 0.333563483 | 3.125988556 | 0.001772084 | 0.007674023 |
| *SETD7* | 1.042516894 | 0.277163109 | 3.761384033 | 0.000168976 | 0.001087215 |
| *FCGR1A* | 1.040984987 | 0.329587548 | 3.158447565 | 0.001586118 | 0.006999883 |
| *MARVELD2* | 1.039957176 | 0.391997275 | 2.652970422 | 0.007978686 | 0.026441927 |
| *TAF7L* | 1.038728768 | 0.404780066 | 2.566155937 | 0.010283259 | 0.032578747 |
| *CABYR* | 1.038436719 | 0.271873518 | 3.81955818 | 0.000133691 | 0.000893229 |
| *ADAM8* | 1.038389052 | 0.277011731 | 3.748538184 | 0.000177868 | 0.001135201 |
| *NAA60* | 1.037195281 | 0.192413803 | 5.390441138 | 7.03E-08 | 1.30E-06 |
| *MGAT1* | 1.037006498 | 0.17313997 | 5.989411314 | 2.11E-09 | 5.83E-08 |
| *BMF* | 1.036538856 | 0.23993397 | 4.320100468 | 1.56E-05 | 0.000143143 |
| *PAK3* | 1.036043778 | 0.4007535 | 2.585239502 | 0.009731138 | 0.03118535 |
| *HK1* | 1.033967057 | 0.161040744 | 6.42053081 | 1.36E-10 | 5.02E-09 |
| *CCNE1* | 1.033840559 | 0.237109402 | 4.360183735 | 1.30E-05 | 0.000122727 |
| *UBL3* | 1.033458052 | 0.140161303 | 7.373347893 | 1.66E-13 | 1.08E-11 |
| *RAB30* | 1.03323654 | 0.316506768 | 3.264500621 | 0.001096572 | 0.005142156 |
| *PPM1N* | 1.033062947 | 0.270177725 | 3.823642191 | 0.000131495 | 0.000880228 |
| *MTARC1* | 1.032402741 | 0.404738063 | 2.550792315 | 0.010747835 | 0.033699828 |
| *FNDC7* | 1.032119668 | 0.428987516 | 2.405943364 | 0.016130765 | 0.046676006 |
| *ZBTB43* | 1.031825454 | 0.287844149 | 3.584667109 | 0.000337508 | 0.00194931 |
| *GAS7* | 1.031757076 | 0.207099584 | 4.981936974 | 6.30E-07 | 8.98E-06 |
| *PLB1* | 1.03128337 | 0.226019587 | 4.562805295 | 5.05E-06 | 5.45E-05 |
| *NPTN* | 1.030648738 | 0.154572624 | 6.667731381 | 2.60E-11 | 1.11E-09 |
| *MINK1* | 1.030321145 | 0.153667726 | 6.704863606 | 2.02E-11 | 8.84E-10 |
| *ANXA2* | 1.02926747 | 0.315514284 | 3.26218977 | 0.001105551 | 0.005176353 |
| *ZCCHC2* | 1.028700987 | 0.165919036 | 6.200017847 | 5.65E-10 | 1.78E-08 |
| *ARHGAP20* | 1.027945146 | 0.331674611 | 3.099257858 | 0.001940061 | 0.008266869 |
| *LTB4R* | 1.02648487 | 0.203039093 | 5.055602112 | 4.29E-07 | 6.42E-06 |
| *GRPEL1* | 1.026454856 | 0.172309813 | 5.957030753 | 2.57E-09 | 6.94E-08 |
| *FKBP1A* | 1.026300502 | 0.119340118 | 8.599794599 | 7.99E-18 | 1.23E-15 |
| *GNB2* | 1.02607371 | 0.133614719 | 7.679346366 | 1.60E-14 | 1.26E-12 |
| *ZFP37* | 1.025616532 | 0.428312663 | 2.394551038 | 0.016640727 | 0.047902052 |
| *FZD5* | 1.025498187 | 0.246795223 | 4.155259465 | 3.25E-05 | 0.000266053 |
| *BTG2* | 1.02545397 | 0.223548122 | 4.587173262 | 4.49E-06 | 4.92E-05 |
| *PHF13* | 1.025433073 | 0.258018055 | 3.974268672 | 7.06E-05 | 0.000520928 |
| *PLEKHG2* | 1.025117323 | 0.233017574 | 4.399313343 | 1.09E-05 | 0.000105539 |
| *RIOK3* | 1.024976736 | 0.250915753 | 4.084943744 | 4.41E-05 | 0.000347173 |
| *FAM72A* | 1.024489475 | 0.214437853 | 4.777558909 | 1.77E-06 | 2.22E-05 |
| *SAT1* | 1.024246662 | 0.286804477 | 3.571236655 | 0.0003553 | 0.002037936 |
| *SLC12A8* | 1.023688175 | 0.327426595 | 3.126466179 | 0.001769209 | 0.007663665 |
| *SPRY3* | 1.020900777 | 0.192673087 | 5.298616404 | 1.17E-07 | 2.04E-06 |
| *PKMYT1* | 1.020890046 | 0.272381088 | 3.748021031 | 0.000178235 | 0.001137048 |
| *PHC2* | 1.020019126 | 0.202395857 | 5.039723341 | 4.66E-07 | 6.91E-06 |
| *MAP3K8* | 1.019865474 | 0.28103675 | 3.628939891 | 0.000284587 | 0.001686225 |
| *TCP11L2* | 1.019213121 | 0.183765798 | 5.546261223 | 2.92E-08 | 6.00E-07 |
| *NEDD4L* | 1.01893522 | 0.369696188 | 2.756142077 | 0.005848759 | 0.020462898 |
| *LIPE* | 1.018018761 | 0.306821485 | 3.317951347 | 0.000906803 | 0.004408131 |
| *VPS9D1* | 1.017921837 | 0.224204362 | 4.540151798 | 5.62E-06 | 5.98E-05 |
| *ADCY4* | 1.017420561 | 0.281292489 | 3.61694891 | 0.000298096 | 0.00175335 |
| *STBD1* | 1.017315731 | 0.265305578 | 3.83450562 | 0.000125817 | 0.000849587 |
| *ZNFX1* | 1.016076023 | 0.180396432 | 5.632461865 | 1.78E-08 | 3.84E-07 |
| *TUBB2A* | 1.015271241 | 0.326888365 | 3.105865339 | 0.001897231 | 0.008120436 |
| *MICAL1* | 1.01493618 | 0.241089911 | 4.209782882 | 2.56E-05 | 0.000217113 |
| *FAM110A* | 1.014009715 | 0.170603337 | 5.943668722 | 2.79E-09 | 7.46E-08 |
| *ADGRE1* | 1.013973865 | 0.308147254 | 3.290549734 | 0.000999918 | 0.004762802 |
| *WSB2* | 1.012509386 | 0.182841685 | 5.537628832 | 3.07E-08 | 6.25E-07 |
| *MYBL2* | 1.012326691 | 0.274299898 | 3.690583554 | 0.00022374 | 0.001375552 |
| *REL* | 1.010512752 | 0.208133788 | 4.855111513 | 1.20E-06 | 1.58E-05 |
| *LAP3* | 1.010224698 | 0.132987541 | 7.596386006 | 3.05E-14 | 2.24E-12 |
| *GRK7* | 1.009724688 | 0.296041012 | 3.410759483 | 0.000647822 | 0.003334004 |
| *AOX1* | 1.009497802 | 0.415726194 | 2.428275671 | 0.015170807 | 0.044459088 |
| *LPAR2* | 1.008962657 | 0.221803893 | 4.548895166 | 5.39E-06 | 5.76E-05 |
| *EIF4E3* | 1.008149588 | 0.259207969 | 3.889346421 | 0.000100515 | 0.000700548 |
| *SIRPB2* | 1.008058839 | 0.336532727 | 2.995425873 | 0.002740619 | 0.010977735 |
| *MREG* | 1.007857199 | 0.347206466 | 2.90276045 | 0.003698894 | 0.014024698 |
| *MTMR3* | 1.007405819 | 0.206003612 | 4.890233774 | 1.01E-06 | 1.36E-05 |
| *ARHGAP9* | 1.007110113 | 0.148586342 | 6.77794541 | 1.22E-11 | 5.65E-10 |
| *CAP1* | 1.003612368 | 0.143732176 | 6.982517022 | 2.90E-12 | 1.49E-10 |
| *SRA1* | 1.003431716 | 0.149806744 | 6.698174532 | 2.11E-11 | 9.19E-10 |
| *SUCLG2* | 1.003287626 | 0.269940925 | 3.716693297 | 0.000201847 | 0.001262283 |
| *MTHFS* | 1.003061234 | 0.161324186 | 6.217674244 | 5.05E-10 | 1.62E-08 |
| *PITPNM1* | 1.003015406 | 0.158200751 | 6.340143148 | 2.30E-10 | 8.15E-09 |
| *DUSP1* | 1.002416291 | 0.237178146 | 4.226427712 | 2.37E-05 | 0.000204242 |
| *SYNM* | 1.00092001 | 0.410743647 | 2.436848425 | 0.014815888 | 0.043649303 |
| *POLE4* | 1.00041824 | 0.172166219 | 5.810769641 | 6.22E-09 | 1.51E-07 |
| *TREX1* | 1.000112921 | 0.155367277 | 6.43708857 | 1.22E-10 | 4.54E-09 |
| *LSR* | 1.000014743 | 0.347604865 | 2.876872114 | 0.004016383 | 0.015014482 |
| *VANGL1* | -1.001325021 | 0.290108998 | -3.451547624 | 0.000557381 | 0.002954037 |
| *DNAL1* | -1.002580176 | 0.139253996 | -7.199651016 | 6.04E-13 | 3.52E-11 |
| *ALDH7A1* | -1.003118842 | 0.339752872 | -2.952495545 | 0.003152166 | 0.012338936 |
| *HHAT* | -1.004533346 | 0.269252739 | -3.730819411 | 0.000190858 | 0.001205467 |
| *A1BG* | -1.005899301 | 0.26495441 | -3.796499565 | 0.000146754 | 0.00096623 |
| *MYOM2* | -1.006091409 | 0.331747836 | -3.03269924 | 0.00242377 | 0.009937427 |
| *TMEM182* | -1.006744319 | 0.222418032 | -4.526361051 | 6.00E-06 | 6.34E-05 |
| *UBE2F-SCLY* | -1.007240743 | 0.414758137 | -2.428501465 | 0.015161364 | 0.044455394 |
| *FSCN1* | -1.008400472 | 0.301582357 | -3.343698487 | 0.000826695 | 0.004072497 |
| *SPACA9* | -1.008410166 | 0.247951606 | -4.066963636 | 4.76E-05 | 0.000371188 |
| *ZNF594* | -1.010435771 | 0.299567193 | -3.372985408 | 0.000743579 | 0.003733344 |
| *SLC22A16* | -1.010812291 | 0.227342022 | -4.446218435 | 8.74E-06 | 8.73E-05 |
| *PCDHGA6* | -1.011694493 | 0.352162689 | -2.872804317 | 0.00406846 | 0.015162606 |
| *B4GAT1* | -1.011804443 | 0.221249765 | -4.573132283 | 4.80E-06 | 5.22E-05 |
| *SLC26A1* | -1.01276442 | 0.21300908 | -4.754559848 | 1.99E-06 | 2.44E-05 |
| *NTNG2* | -1.01314435 | 0.407707481 | -2.484978564 | 0.012955924 | 0.039127334 |
| *GOLGA8Q* | -1.013945266 | 0.203083841 | -4.992742215 | 5.95E-07 | 8.56E-06 |
| *TP53BP1* | -1.014751569 | 0.13744575 | -7.382924321 | 1.55E-13 | 1.00E-11 |
| *CPXM1* | -1.016003487 | 0.33677272 | -3.016881787 | 0.002553894 | 0.010382618 |
| *TNS2* | -1.017087497 | 0.276456196 | -3.679018633 | 0.000234133 | 0.00143277 |
| *APOLD1* | -1.018593722 | 0.290181355 | -3.510197002 | 0.000447775 | 0.002475076 |
| *PLTP* | -1.018610391 | 0.264972033 | -3.844218501 | 0.000120937 | 0.000822072 |
| *CPLANE1* | -1.018973809 | 0.161563101 | -6.306971085 | 2.85E-10 | 9.87E-09 |
| *PM20D2* | -1.019990631 | 0.195200357 | -5.225352281 | 1.74E-07 | 2.91E-06 |
| *ZNF74* | -1.020084776 | 0.224078235 | -4.552359913 | 5.30E-06 | 5.69E-05 |
| *LAPTM4B* | -1.02012921 | 0.392591696 | -2.598448257 | 0.009364616 | 0.030221865 |
| *CFAP43* | -1.022697294 | 0.378484695 | -2.702083618 | 0.006890643 | 0.023419605 |
| *ZNF248* | -1.023580874 | 0.196291271 | -5.214602103 | 1.84E-07 | 3.07E-06 |
| *ARHGAP22* | -1.024774934 | 0.364179956 | -2.813924594 | 0.004894071 | 0.017663516 |
| *EFHC2* | -1.028388842 | 0.308285249 | -3.335835381 | 0.000850435 | 0.004173107 |
| *TIE1* | -1.028916038 | 0.372654701 | -2.76104403 | 0.005761691 | 0.020231063 |
| *DNAAF4* | -1.029274219 | 0.340584225 | -3.022084241 | 0.002510407 | 0.010234312 |
| *MORN1* | -1.029866609 | 0.174996577 | -5.885067172 | 3.98E-09 | 1.02E-07 |
| *C19orf44* | -1.030094593 | 0.235741552 | -4.369592821 | 1.24E-05 | 0.00011812 |
| *AFAP1L2* | -1.031008291 | 0.376255146 | -2.740183897 | 0.006140482 | 0.021325627 |
| *ZNF382* | -1.031909018 | 0.25416955 | -4.059923852 | 4.91E-05 | 0.000380777 |
| *TEX30* | -1.032616796 | 0.215615194 | -4.789165252 | 1.67E-06 | 2.11E-05 |
| *GFI1B* | -1.032847411 | 0.393637658 | -2.623853152 | 0.008694126 | 0.028364553 |
| *NTN1* | -1.034862704 | 0.411353928 | -2.515747715 | 0.011878017 | 0.036555563 |
| *LRP11* | -1.037444004 | 0.365963308 | -2.834830654 | 0.004584998 | 0.016735006 |
| *PLPP1* | -1.037709094 | 0.29927787 | -3.467376639 | 0.000525565 | 0.002816537 |
| *ITM2C* | -1.039074447 | 0.277154991 | -3.749073549 | 0.000177489 | 0.001133009 |
| *SPN* | -1.039482238 | 0.196004529 | -5.303358271 | 1.14E-07 | 2.00E-06 |
| *CALR* | -1.039705171 | 0.222436544 | -4.674165282 | 2.95E-06 | 3.45E-05 |
| *B3GALNT1* | -1.040256909 | 0.303003275 | -3.433154012 | 0.000596603 | 0.003120117 |
| *CLDN12* | -1.040988012 | 0.237248352 | -4.387756551 | 1.15E-05 | 0.000110028 |
| *ZNF737* | -1.041655806 | 0.256175007 | -4.066188261 | 4.78E-05 | 0.000372241 |
| *NLGN2* | -1.043126697 | 0.268147419 | -3.890123951 | 0.000100193 | 0.000698615 |
| *TTC30A* | -1.044311268 | 0.215553633 | -4.844786202 | 1.27E-06 | 1.66E-05 |
| *NHLRC4* | -1.04479747 | 0.248452448 | -4.205221081 | 2.61E-05 | 0.000220887 |
| *CAPN14* | -1.045335857 | 0.306589182 | -3.409565364 | 0.000650665 | 0.003345531 |
| *MEGF6* | -1.04547523 | 0.218023323 | -4.795244915 | 1.62E-06 | 2.05E-05 |
| *TMEM220* | -1.045760373 | 0.203190113 | -5.146708948 | 2.65E-07 | 4.22E-06 |
| *FNBP1L* | -1.045788663 | 0.33448192 | -3.126592498 | 0.001768449 | 0.007662214 |
| *ZNF107* | -1.048051155 | 0.187719956 | -5.583056678 | 2.36E-08 | 4.99E-07 |
| *FAM161A* | -1.049580293 | 0.23681817 | -4.432009127 | 9.34E-06 | 9.24E-05 |
| *SAXO2* | -1.051357133 | 0.302009464 | -3.48120591 | 0.000499162 | 0.002705266 |
| *TMIGD2* | -1.052155299 | 0.406304586 | -2.589572784 | 0.009609511 | 0.030870577 |
| *ALS2* | -1.052191499 | 0.219653042 | -4.790243241 | 1.67E-06 | 2.10E-05 |
| *ARMC9* | -1.054433657 | 0.281843277 | -3.741205636 | 0.00018314 | 0.001163449 |
| *AK9* | -1.05560357 | 0.180211881 | -5.857569218 | 4.70E-09 | 1.18E-07 |
| *ECM1* | -1.055814146 | 0.4372079 | -2.414901804 | 0.015739452 | 0.045817443 |
| *STPG2* | -1.056362374 | 0.303178609 | -3.484290591 | 0.000493443 | 0.002678862 |
| *LZTFL1* | -1.056852172 | 0.145839735 | -7.2466682 | 4.27E-13 | 2.57E-11 |
| *PHLDB1* | -1.058379135 | 0.352891162 | -2.999165891 | 0.002707199 | 0.010878279 |
| *INHA* | -1.059067907 | 0.426645985 | -2.482310734 | 0.013053339 | 0.039375144 |
| *PTGES3L* | -1.059819218 | 0.240768282 | -4.401822405 | 1.07E-05 | 0.000104429 |
| *P3H4* | -1.059850995 | 0.247836549 | -4.276411196 | 1.90E-05 | 0.000169174 |
| *PROB1* | -1.060019641 | 0.184632341 | -5.741245727 | 9.40E-09 | 2.18E-07 |
| *TM7SF3* | -1.061294552 | 0.203913975 | -5.204619011 | 1.94E-07 | 3.22E-06 |
| *SLC14A1* | -1.062291779 | 0.384841233 | -2.760337739 | 0.005774163 | 0.020269119 |
| *LBX2* | -1.062964811 | 0.294643029 | -3.607636039 | 0.000309 | 0.001806073 |
| *C17orf113* | -1.06369254 | 0.211657218 | -5.025543428 | 5.02E-07 | 7.39E-06 |
| *ABCA2* | -1.063820878 | 0.181243888 | -5.869554508 | 4.37E-09 | 1.11E-07 |
| *GOLGA8T* | -1.06418412 | 0.209056715 | -5.090408702 | 3.57E-07 | 5.46E-06 |
| *KCTD16* | -1.064377384 | 0.400674762 | -2.65646226 | 0.007896529 | 0.026213561 |
| *ZNF334* | -1.064446586 | 0.435177938 | -2.446003101 | 0.014444976 | 0.042763505 |
| *CUBN* | -1.064965755 | 0.290280503 | -3.668747103 | 0.000243742 | 0.001481547 |
| *C8orf37* | -1.067084246 | 0.156734646 | -6.808221892 | 9.88E-12 | 4.64E-10 |
| *RASGRF1* | -1.068612086 | 0.415447499 | -2.572195255 | 0.010105588 | 0.032109053 |
| *ARMCX1* | -1.070049983 | 0.223401138 | -4.789814388 | 1.67E-06 | 2.10E-05 |
| *ADAM22* | -1.070189021 | 0.337819243 | -3.167933864 | 0.001535264 | 0.006806769 |
| *RBMS2* | -1.070756787 | 0.238492698 | -4.489683738 | 7.13E-06 | 7.35E-05 |
| *WDR35* | -1.070795306 | 0.214118881 | -5.000938274 | 5.71E-07 | 8.25E-06 |
| *ADCY6* | -1.072799996 | 0.342484648 | -3.132403165 | 0.001733816 | 0.007534068 |
| *MGST1* | -1.074056199 | 0.347370616 | -3.091960425 | 0.001988394 | 0.008443271 |
| *ZDHHC9* | -1.074292251 | 0.166096214 | -6.467891278 | 9.94E-11 | 3.76E-09 |
| *PCDHGB4* | -1.075409998 | 0.415962981 | -2.585350251 | 0.009728012 | 0.031179315 |
| *SOX12* | -1.07630385 | 0.231921492 | -4.640811177 | 3.47E-06 | 3.97E-05 |
| *LRRIQ3* | -1.076682188 | 0.296412607 | -3.632376505 | 0.000280823 | 0.001667554 |
| *ADA* | -1.077251115 | 0.25587858 | -4.210008969 | 2.55E-05 | 0.000216954 |
| *ERMP1* | -1.078787491 | 0.231976956 | -4.650408004 | 3.31E-06 | 3.80E-05 |
| *PROX2* | -1.078996403 | 0.263210437 | -4.099367845 | 4.14E-05 | 0.000328189 |
| *ST3GAL4* | -1.079069275 | 0.220989788 | -4.882892038 | 1.05E-06 | 1.41E-05 |
| *EVA1B* | -1.081161794 | 0.2523945 | -4.28361868 | 1.84E-05 | 0.000164477 |
| *PACSIN3* | -1.082231574 | 0.31884552 | -3.394219161 | 0.000688246 | 0.003503878 |
| *RGPD1* | -1.082647325 | 0.409009607 | -2.646997297 | 0.008121 | 0.026812514 |
| *GUCY1B1* | -1.082735737 | 0.360416068 | -3.004127262 | 0.002663439 | 0.010735144 |
| *GRIK5* | -1.083609365 | 0.347595876 | -3.117440226 | 0.001824289 | 0.00786088 |
| *SPNS2* | -1.083675986 | 0.308739749 | -3.509998269 | 0.00044811 | 0.002476494 |
| *KLHL41* | -1.083798783 | 0.431655816 | -2.510793887 | 0.012046001 | 0.036964734 |
| *ZNF501* | -1.083882811 | 0.249272741 | -4.348180261 | 1.37E-05 | 0.000128629 |
| *TTC23* | -1.084349832 | 0.334323424 | -3.243415664 | 0.001181058 | 0.005475624 |
| *CLIP2* | -1.085002448 | 0.253703257 | -4.276659513 | 1.90E-05 | 0.000169048 |
| *PAPPA2* | -1.085135073 | 0.414979651 | -2.614911525 | 0.008925057 | 0.029026607 |
| *FTCD* | -1.086381948 | 0.426540906 | -2.546958408 | 0.010866638 | 0.03401506 |
| *LRRC34* | -1.086596093 | 0.282275374 | -3.849418662 | 0.000118398 | 0.0008062 |
| *ZNF70* | -1.087616521 | 0.201589113 | -5.395214567 | 6.84E-08 | 1.27E-06 |
| *BMP1* | -1.087619426 | 0.182730414 | -5.952043793 | 2.65E-09 | 7.15E-08 |
| *CHST12* | -1.087832636 | 0.20746186 | -5.243530709 | 1.58E-07 | 2.67E-06 |
| *PLEKHG4* | -1.089358162 | 0.289426235 | -3.763854239 | 0.000167314 | 0.001078718 |
| *ZNF781* | -1.090146471 | 0.401328624 | -2.716343677 | 0.006600736 | 0.022618845 |
| *USP54* | -1.091190515 | 0.257290658 | -4.241080974 | 2.22E-05 | 0.000192819 |
| *LRRIQ4* | -1.092386031 | 0.391183882 | -2.792512885 | 0.005230038 | 0.018686467 |
| *ZNF608* | -1.092386656 | 0.376795498 | -2.899149967 | 0.003741759 | 0.014149936 |
| *TMSB15B* | -1.094092096 | 0.276732148 | -3.953614008 | 7.70E-05 | 0.000561231 |
| *PHYHD1* | -1.095641181 | 0.327411681 | -3.346371689 | 0.000818766 | 0.00404161 |
| *MPP4* | -1.095832515 | 0.431876583 | -2.537374236 | 0.011168749 | 0.03478189 |
| *GATM* | -1.098065359 | 0.44410927 | -2.472511683 | 0.013416733 | 0.040280737 |
| *DNM1* | -1.098272216 | 0.326448209 | -3.364307681 | 0.000767359 | 0.003833286 |
| *SH2D1A* | -1.098375985 | 0.394854484 | -2.781723467 | 0.005407109 | 0.019199938 |
| *CEL* | -1.099012545 | 0.302535496 | -3.63267306 | 0.0002805 | 0.001666264 |
| *DNAAF3* | -1.099691163 | 0.458695147 | -2.397433612 | 0.016510373 | 0.04757873 |
| *DNAI4* | -1.099935996 | 0.294103117 | -3.73996715 | 0.000184044 | 0.001168493 |
| *TWNK* | -1.100354543 | 0.209025273 | -5.264217716 | 1.41E-07 | 2.41E-06 |
| *SMO* | -1.10069432 | 0.38650509 | -2.847813259 | 0.004402075 | 0.016179273 |
| *FAM216A* | -1.10152226 | 0.211080749 | -5.21848756 | 1.80E-07 | 3.02E-06 |
| *PTCH1* | -1.101896006 | 0.428271204 | -2.572893054 | 0.010085237 | 0.032076585 |
| *NPB* | -1.104533123 | 0.261908905 | -4.217241575 | 2.47E-05 | 0.000211197 |
| *RADIL* | -1.107729472 | 0.452386453 | -2.448635375 | 0.014339853 | 0.04251193 |
| *AVEN* | -1.108708589 | 0.18678505 | -5.935745879 | 2.93E-09 | 7.78E-08 |
| *ZNF425* | -1.113366743 | 0.21974304 | -5.06667581 | 4.05E-07 | 6.09E-06 |
| *SORBS1* | -1.114096376 | 0.306051317 | -3.640227351 | 0.000272397 | 0.001625757 |
| *HACD1* | -1.114882255 | 0.254087973 | -4.387780504 | 1.15E-05 | 0.000110028 |
| *FKBP7* | -1.115172322 | 0.222636583 | -5.008935666 | 5.47E-07 | 7.98E-06 |
| *CCDC168* | -1.115520131 | 0.322258478 | -3.461569542 | 0.000537035 | 0.002865395 |
| *COL24A1* | -1.115653877 | 0.36437034 | -3.06186798 | 0.002199604 | 0.009152297 |
| *CCDC62* | -1.116630389 | 0.204784359 | -5.45271325 | 4.96E-08 | 9.56E-07 |
| *AQP3* | -1.118583806 | 0.335712554 | -3.331968952 | 0.000862339 | 0.004221806 |
| *PDGFD* | -1.119997087 | 0.404873878 | -2.766286363 | 0.005669872 | 0.019965014 |
| *FAM71E1* | -1.121336002 | 0.249710257 | -4.490548436 | 7.10E-06 | 7.33E-05 |
| *ZKSCAN7* | -1.121678819 | 0.264773518 | -4.236370869 | 2.27E-05 | 0.000196315 |
| *IFT81* | -1.121872684 | 0.218364705 | -5.137609962 | 2.78E-07 | 4.41E-06 |
| *LPAR5* | -1.121877046 | 0.275566373 | -4.071168169 | 4.68E-05 | 0.00036536 |
| *DNMT3B* | -1.123039057 | 0.316309419 | -3.550444566 | 0.000384581 | 0.002176314 |
| *PCYT1B* | -1.123945128 | 0.470060386 | -2.391065407 | 0.01679956 | 0.048262716 |
| *ESPNL* | -1.125551714 | 0.40164923 | -2.802325086 | 0.005073572 | 0.018221635 |
| *GOLGA8N* | -1.126458677 | 0.221658235 | -5.081961775 | 3.74E-07 | 5.69E-06 |
| *PCDHGA7* | -1.128882449 | 0.407466782 | -2.770489521 | 0.00559721 | 0.019754734 |
| *TBC1D16* | -1.131010982 | 0.329330431 | -3.434274132 | 0.000594143 | 0.00310982 |
| *TCP11* | -1.132397666 | 0.312144995 | -3.627793765 | 0.000285853 | 0.001692346 |
| *CCDC40* | -1.13425447 | 0.411141001 | -2.75879678 | 0.00580146 | 0.020340128 |
| *MIP* | -1.135875097 | 0.363859736 | -3.121738911 | 0.001797863 | 0.007767593 |
| *CCDC169* | -1.138074138 | 0.306848029 | -3.708917864 | 0.000208147 | 0.001294773 |
| *TANC1* | -1.138888873 | 0.415105202 | -2.743615031 | 0.006076675 | 0.021141636 |
| *NT5M* | -1.139131346 | 0.23459289 | -4.855779503 | 1.20E-06 | 1.58E-05 |
| *DLL1* | -1.139363223 | 0.423429553 | -2.690797597 | 0.007128143 | 0.024094873 |
| *TEC* | -1.143877338 | 0.204255391 | -5.600230823 | 2.14E-08 | 4.55E-07 |
| *LAX1* | -1.144141796 | 0.301098192 | -3.799895935 | 0.000144757 | 0.000955069 |
| *ZSCAN2* | -1.14491907 | 0.158373378 | -7.229239441 | 4.86E-13 | 2.88E-11 |
| *DUSP19* | -1.145725726 | 0.286402972 | -4.000397472 | 6.32E-05 | 0.000473016 |
| *MAP7* | -1.145740753 | 0.340566402 | -3.364221328 | 0.0007676 | 0.00383388 |
| *CERKL* | -1.146288511 | 0.383986867 | -2.985228427 | 0.002833666 | 0.011301819 |
| *ZNF665* | -1.146803921 | 0.181446898 | -6.320328043 | 2.61E-10 | 9.15E-09 |
| *TP63* | -1.147097726 | 0.470757936 | -2.436703959 | 0.014821808 | 0.04366268 |
| *FAM166B* | -1.147139242 | 0.409948986 | -2.798248758 | 0.005138052 | 0.018418094 |
| *MIA* | -1.148563561 | 0.458662765 | -2.504156971 | 0.012274356 | 0.037505504 |
| *TRIM16* | -1.150302533 | 0.26764919 | -4.297799416 | 1.73E-05 | 0.000155754 |
| *CREB3L4* | -1.150382533 | 0.182590359 | -6.300346519 | 2.97E-10 | 1.02E-08 |
| *PLEKHA5* | -1.152067138 | 0.220942902 | -5.214320657 | 1.84E-07 | 3.07E-06 |
| *TXLNB* | -1.152995367 | 0.325287411 | -3.544543464 | 0.000393294 | 0.002216899 |
| *ZNF804A* | -1.153175046 | 0.369347317 | -3.122196893 | 0.001795068 | 0.007760815 |
| *LANCL3* | -1.153326013 | 0.382817094 | -3.01273384 | 0.002589059 | 0.010497059 |
| *ZKSCAN3* | -1.15425945 | 0.212647025 | -5.428053602 | 5.70E-08 | 1.08E-06 |
| *C16orf46* | -1.154838737 | 0.214323363 | -5.388300754 | 7.11E-08 | 1.31E-06 |
| *PCDHGA9* | -1.155175895 | 0.406408253 | -2.84240265 | 0.00447749 | 0.016397436 |
| *GUCY2F* | -1.156381495 | 0.471615535 | -2.451958024 | 0.014208124 | 0.042220253 |
| *P2RX6* | -1.156386519 | 0.31741974 | -3.643083187 | 0.000269392 | 0.001610855 |
| *CHODL* | -1.157900416 | 0.375549078 | -3.083219965 | 0.002047737 | 0.00865001 |
| *ZC3HAV1L* | -1.159676527 | 0.270782915 | -4.282679816 | 1.85E-05 | 0.000165079 |
| *ARL10* | -1.159878829 | 0.294950846 | -3.93244788 | 8.41E-05 | 0.000604131 |
| *SYNPO2L* | -1.160543046 | 0.444117159 | -2.613146154 | 0.008971293 | 0.029145653 |
| *ZNF320* | -1.162540727 | 0.195828518 | -5.936524155 | 2.91E-09 | 7.76E-08 |
| *TMEM232* | -1.165699331 | 0.286177209 | -4.073347897 | 4.63E-05 | 0.000362671 |
| *KRBA2* | -1.166001955 | 0.248733606 | -4.687753998 | 2.76E-06 | 3.25E-05 |
| *ZNF404* | -1.166728823 | 0.374437194 | -3.115953335 | 0.001833513 | 0.007891459 |
| *GPR174* | -1.166860464 | 0.305399182 | -3.820771408 | 0.000133035 | 0.000889222 |
| *IQSEC3* | -1.168144754 | 0.422314906 | -2.76605144 | 0.005673958 | 0.019972343 |
| *SARDH* | -1.170444576 | 0.333340064 | -3.511262832 | 0.000445983 | 0.002466465 |
| *CMBL* | -1.171854732 | 0.318303741 | -3.681561292 | 0.00023181 | 0.001420751 |
| *MMP11* | -1.17236592 | 0.356027156 | -3.292911508 | 0.000991557 | 0.004732919 |
| *MPL* | -1.172854152 | 0.424789551 | -2.761024019 | 0.005762044 | 0.020231063 |
| *TMEM67* | -1.172856342 | 0.243199641 | -4.822607203 | 1.42E-06 | 1.82E-05 |
| *NPW* | -1.175259005 | 0.313633831 | -3.747232884 | 0.000178796 | 0.001139056 |
| *SNORC* | -1.176513002 | 0.299568136 | -3.927363631 | 8.59E-05 | 0.000614948 |
| *SPATA17* | -1.176760008 | 0.384348709 | -3.061698873 | 0.002200847 | 0.009153859 |
| *LGR4* | -1.177437401 | 0.432568123 | -2.72196988 | 0.006489406 | 0.022312222 |
| *ZNF112* | -1.178849116 | 0.472048507 | -2.497305038 | 0.012514126 | 0.038058139 |
| *ZDHHC23* | -1.18005437 | 0.274019161 | -4.306466617 | 1.66E-05 | 0.00015068 |
| *CLBA1* | -1.180599066 | 0.259413596 | -4.551030028 | 5.34E-06 | 5.71E-05 |
| *SCG3* | -1.181859285 | 0.467633737 | -2.527318268 | 0.011493729 | 0.035585453 |
| *PCDHGA8* | -1.182039359 | 0.468108323 | -2.525140661 | 0.011565199 | 0.035766699 |
| *TMEM17* | -1.183584165 | 0.269643233 | -4.389445084 | 1.14E-05 | 0.000109472 |
| *NIPA1* | -1.184733508 | 0.215243826 | -5.504146296 | 3.71E-08 | 7.41E-07 |
| *ICAM5* | -1.184933656 | 0.388381988 | -3.050949045 | 0.002281193 | 0.009438576 |
| *MSI2* | -1.186506001 | 0.26853497 | -4.418441302 | 9.94E-06 | 9.76E-05 |
| *CEP41* | -1.187095562 | 0.217474557 | -5.458549165 | 4.80E-08 | 9.29E-07 |
| *FUT2* | -1.187603334 | 0.478347386 | -2.482721486 | 0.013038298 | 0.039342329 |
| *GSTA4* | -1.188160804 | 0.387013226 | -3.070078037 | 0.002140028 | 0.008970437 |
| *CARNS1* | -1.188546474 | 0.302807006 | -3.925095693 | 8.67E-05 | 0.000619951 |
| *AFF2* | -1.189966728 | 0.429588736 | -2.7700138 | 0.005605392 | 0.019777562 |
| *CNIH3* | -1.192549679 | 0.194799819 | -6.12192397 | 9.25E-10 | 2.76E-08 |
| *PHLDA3* | -1.195489794 | 0.360245016 | -3.318546383 | 0.000904873 | 0.004400431 |
| *RHOBTB1* | -1.197212667 | 0.366049302 | -3.270632293 | 0.001073073 | 0.005048181 |
| *GPR88* | -1.197764106 | 0.470116028 | -2.547805294 | 0.010840296 | 0.03394267 |
| *DIXDC1* | -1.19781957 | 0.295766911 | -4.049876867 | 5.12E-05 | 0.000395176 |
| *ST8SIA6* | -1.198839741 | 0.421669887 | -2.843076488 | 0.004468035 | 0.016372277 |
| *IFT22* | -1.198872446 | 0.20365146 | -5.88688362 | 3.94E-09 | 1.01E-07 |
| *KCNA6* | -1.19889399 | 0.453306351 | -2.644776511 | 0.008174489 | 0.026963199 |
| *THNSL1* | -1.20217573 | 0.299994957 | -4.007319795 | 6.14E-05 | 0.000461112 |
| *C21orf91* | -1.202654362 | 0.212823698 | -5.650941955 | 1.60E-08 | 3.48E-07 |
| *RSPH1* | -1.203879169 | 0.378965055 | -3.176755096 | 0.001489327 | 0.006651545 |
| *RAB40A* | -1.204121985 | 0.314083055 | -3.833769341 | 0.000126194 | 0.000851954 |
| *ADAMTS6* | -1.206820966 | 0.325161636 | -3.711449418 | 0.000206076 | 0.001285033 |
| *SPATA25* | -1.216748715 | 0.212297377 | -5.731341262 | 9.96E-09 | 2.30E-07 |
| *ANKRD63* | -1.216760147 | 0.404425806 | -3.008611542 | 0.002624444 | 0.01060498 |
| *SMAD9* | -1.218147285 | 0.448543977 | -2.715781164 | 0.006611961 | 0.022649954 |
| *TCTN1* | -1.218694537 | 0.212821651 | -5.726365388 | 1.03E-08 | 2.36E-07 |
| *PRR36* | -1.218884894 | 0.391694867 | -3.11182248 | 0.001859363 | 0.007990767 |
| *SRSF12* | -1.220718057 | 0.399860582 | -3.052859198 | 0.002266723 | 0.009384694 |
| *C11orf95* | -1.221431655 | 0.228320383 | -5.349639133 | 8.81E-08 | 1.60E-06 |
| *ZNRF1* | -1.224686146 | 0.186894039 | -6.552836839 | 5.65E-11 | 2.25E-09 |
| *ITPKA* | -1.225136371 | 0.286700305 | -4.273230097 | 1.93E-05 | 0.000171221 |
| *GAL3ST4* | -1.225151617 | 0.320650954 | -3.820826364 | 0.000133005 | 0.000889211 |
| *BEND5* | -1.227864743 | 0.22785009 | -5.388914895 | 7.09E-08 | 1.31E-06 |
| *AGBL4* | -1.2279988 | 0.351653224 | -3.492073205 | 0.000479287 | 0.002615464 |
| *PTGDR2* | -1.228507561 | 0.367891291 | -3.339322212 | 0.000839831 | 0.004128212 |
| *KANK1* | -1.229301235 | 0.221076548 | -5.560523036 | 2.69E-08 | 5.58E-07 |
| *RLN2* | -1.230822506 | 0.391705699 | -3.142212407 | 0.001676763 | 0.007329415 |
| *STOX1* | -1.231898701 | 0.313927411 | -3.924151432 | 8.70E-05 | 0.000621944 |
| *C3orf80* | -1.232224837 | 0.488312443 | -2.523435263 | 0.011621446 | 0.035905571 |
| *AKNAD1* | -1.23271321 | 0.405820841 | -3.037579853 | 0.002384862 | 0.009804571 |
| *DEPDC7* | -1.235518223 | 0.341188864 | -3.621214973 | 0.000293223 | 0.001729184 |
| *GOLGA8J* | -1.235728609 | 0.209782543 | -5.890521643 | 3.85E-09 | 9.90E-08 |
| *EXTL2* | -1.238816242 | 0.224333066 | -5.522218667 | 3.35E-08 | 6.77E-07 |
| *ZNF497* | -1.239726365 | 0.198830606 | -6.235088208 | 4.52E-10 | 1.46E-08 |
| *MAPK12* | -1.240248271 | 0.328729565 | -3.772852832 | 0.000161391 | 0.001049078 |
| *SMIM24* | -1.241348807 | 0.340226148 | -3.648599068 | 0.000263674 | 0.001582045 |
| *PARD6G* | -1.241942637 | 0.45010472 | -2.759230422 | 0.005793767 | 0.02032536 |
| *MYB* | -1.242894706 | 0.184313819 | -6.743361457 | 1.55E-11 | 6.97E-10 |
| *SLA2* | -1.243464387 | 0.34397909 | -3.614941787 | 0.000300415 | 0.001764368 |
| *REP15* | -1.243816952 | 0.343088356 | -3.625354611 | 0.000288565 | 0.001705212 |
| *KRT8* | -1.245273914 | 0.374151811 | -3.328258415 | 0.000873908 | 0.004267883 |
| *LNP1* | -1.245539388 | 0.218157981 | -5.709345961 | 1.13E-08 | 2.58E-07 |
| *KIAA0895* | -1.246233588 | 0.314927156 | -3.957212215 | 7.58E-05 | 0.000553815 |
| *ADGRA3* | -1.246328321 | 0.308991396 | -4.033537303 | 5.49E-05 | 0.000419308 |
| *PTPRF* | -1.246357542 | 0.404580648 | -3.080615814 | 0.00206573 | 0.008709743 |
| *ASPHD1* | -1.249129718 | 0.42888927 | -2.912476028 | 0.003585757 | 0.013690837 |
| *SPINK2* | -1.25034846 | 0.473428503 | -2.641050235 | 0.008264946 | 0.027202656 |
| *AMH* | -1.251478245 | 0.320543756 | -3.904235293 | 9.45E-05 | 0.000665239 |
| *NREP* | -1.251500093 | 0.220786945 | -5.668360922 | 1.44E-08 | 3.18E-07 |
| *PPFIBP1* | -1.251957348 | 0.230004769 | -5.443179964 | 5.23E-08 | 1.00E-06 |
| *LRRC43* | -1.253191674 | 0.274024239 | -4.573287665 | 4.80E-06 | 5.22E-05 |
| *LPO* | -1.253776416 | 0.429523498 | -2.918993775 | 0.003511632 | 0.013463029 |
| *EPHA10* | -1.253931822 | 0.36208046 | -3.463130328 | 0.00053393 | 0.002850747 |
| *PCDHGA5* | -1.254125278 | 0.451886784 | -2.7753086 | 0.005514935 | 0.019519318 |
| *MLC1* | -1.254307986 | 0.300598795 | -4.172697977 | 3.01E-05 | 0.000249471 |
| *WDR93* | -1.254489959 | 0.373101965 | -3.362324722 | 0.000772892 | 0.003854839 |
| *REEP2* | -1.255082141 | 0.369296676 | -3.39857416 | 0.000677381 | 0.003458018 |
| *DNAJC12* | -1.255191256 | 0.408974253 | -3.069120484 | 0.0021469 | 0.008993286 |
| *TTLL10* | -1.257330144 | 0.514747202 | -2.44261676 | 0.014581212 | 0.043090259 |
| *TMIE* | -1.257983395 | 0.375171086 | -3.353092608 | 0.00079914 | 0.003963893 |
| *QPRT* | -1.261872865 | 0.291883747 | -4.323203599 | 1.54E-05 | 0.00014139 |
| *KCNIP3* | -1.262370586 | 0.48694135 | -2.592448941 | 0.009529533 | 0.030650974 |
| *GPC2* | -1.262903585 | 0.290647615 | -4.345136575 | 1.39E-05 | 0.000130206 |
| *UNC13A* | -1.263063758 | 0.333682895 | -3.785221764 | 0.000153572 | 0.001005265 |
| *LAMB2* | -1.265316393 | 0.510529728 | -2.478438227 | 0.013195896 | 0.039738326 |
| *ACSM3* | -1.265588651 | 0.26797327 | -4.722816761 | 2.33E-06 | 2.81E-05 |
| *GOLGA8O* | -1.267874887 | 0.209460876 | -6.053039167 | 1.42E-09 | 4.06E-08 |
| *GRID2IP* | -1.269534133 | 0.309431674 | -4.102793088 | 4.08E-05 | 0.000324178 |
| *PIH1D2* | -1.27076093 | 0.250475271 | -5.073398769 | 3.91E-07 | 5.91E-06 |
| *PHYHIP* | -1.271453837 | 0.491622563 | -2.586239796 | 0.00970294 | 0.031113885 |
| *RPGRIP1L* | -1.273947202 | 0.208353573 | -6.114352556 | 9.69E-10 | 2.88E-08 |
| *RFX8* | -1.274681715 | 0.354393271 | -3.596799996 | 0.000322156 | 0.00187363 |
| *ZNF660* | -1.275194288 | 0.204971802 | -6.221315694 | 4.93E-10 | 1.58E-08 |
| *WDR31* | -1.275245242 | 0.225669514 | -5.65094159 | 1.60E-08 | 3.48E-07 |
| *GUCY1A1* | -1.276780309 | 0.424399378 | -3.008440578 | 0.002625921 | 0.01060824 |
| *CTSF* | -1.277160492 | 0.275518084 | -4.635486977 | 3.56E-06 | 4.05E-05 |
| *OCLN* | -1.277195643 | 0.385984152 | -3.308932858 | 0.000936523 | 0.004519294 |
| *POLR1F* | -1.278303927 | 0.231978335 | -5.510445303 | 3.58E-08 | 7.18E-07 |
| *TTC39A* | -1.278444129 | 0.364633669 | -3.506105545 | 0.000454715 | 0.002507308 |
| *NPAS1* | -1.278478158 | 0.362421124 | -3.527603862 | 0.000419339 | 0.0023412 |
| *MEST* | -1.280310587 | 0.368479138 | -3.474580933 | 0.000511652 | 0.002757381 |
| *CORO2B* | -1.281558476 | 0.431545253 | -2.969696615 | 0.00298094 | 0.011780853 |
| *SIGLEC12* | -1.281930678 | 0.409951921 | -3.127026886 | 0.001765838 | 0.007652205 |
| *NEK5* | -1.282349352 | 0.400618731 | -3.200922104 | 0.001369886 | 0.006208318 |
| *ZNF559-ZNF177* | -1.282736168 | 0.38149694 | -3.362376036 | 0.000772748 | 0.00385473 |
| *HTR4* | -1.283001746 | 0.537494979 | -2.387002292 | 0.016986385 | 0.048649327 |
| *PTK7* | -1.285274128 | 0.320392791 | -4.011557579 | 6.03E-05 | 0.000454473 |
| *KIT* | -1.286282845 | 0.369457849 | -3.481541528 | 0.000498536 | 0.002702804 |
| *SHF* | -1.28839961 | 0.278074991 | -4.633281135 | 3.60E-06 | 4.09E-05 |
| *MDGA1* | -1.288681184 | 0.406403222 | -3.170942345 | 0.001519453 | 0.0067486 |
| *DLC1* | -1.289114694 | 0.435224343 | -2.961954483 | 0.00305693 | 0.012024126 |
| *TRO* | -1.292284668 | 0.497851338 | -2.595724004 | 0.009439185 | 0.030388167 |
| *PLEKHA6* | -1.292512102 | 0.426269578 | -3.032147183 | 0.002428208 | 0.009953042 |
| *DCHS1* | -1.295245303 | 0.403087518 | -3.213310373 | 0.001312144 | 0.005987781 |
| *SSBP2* | -1.296260571 | 0.19720706 | -6.573094128 | 4.93E-11 | 1.99E-09 |
| *GPRASP2* | -1.297038534 | 0.282626763 | -4.589227575 | 4.45E-06 | 4.89E-05 |
| *C9orf43* | -1.297550597 | 0.326173863 | -3.978094953 | 6.95E-05 | 0.000513216 |
| *RAB9B* | -1.298937427 | 0.300533528 | -4.322104872 | 1.55E-05 | 0.000142013 |
| *CCDC148* | -1.299545057 | 0.369912431 | -3.513115403 | 0.000442885 | 0.002453188 |
| *MLLT11* | -1.300047167 | 0.250837044 | -5.182835631 | 2.19E-07 | 3.55E-06 |
| *NAT8L* | -1.301638063 | 0.344277825 | -3.780778107 | 0.000156339 | 0.001019165 |
| *LRRC28* | -1.303948787 | 0.227658183 | -5.727660509 | 1.02E-08 | 2.34E-07 |
| *DEPTOR* | -1.30731187 | 0.316096645 | -4.135797994 | 3.54E-05 | 0.000287483 |
| *NAALADL2* | -1.308815759 | 0.440532587 | -2.970985119 | 0.002968462 | 0.011747671 |
| *POGLUT2* | -1.309297197 | 0.409229936 | -3.19941696 | 0.001377059 | 0.00623083 |
| *RAB38* | -1.309484673 | 0.312142801 | -4.195146165 | 2.73E-05 | 0.000228917 |
| *KLF17* | -1.309564358 | 0.494713323 | -2.647117627 | 0.008118111 | 0.026808567 |
| *ZNF285* | -1.313156775 | 0.335429772 | -3.914848605 | 9.05E-05 | 0.00064207 |
| *RASD1* | -1.314884716 | 0.479697122 | -2.741072765 | 0.006123894 | 0.021279693 |
| *RNASE10* | -1.315392812 | 0.379659638 | -3.46466329 | 0.000530896 | 0.002838378 |
| *SLC35F2* | -1.316075638 | 0.279179034 | -4.71409195 | 2.43E-06 | 2.91E-05 |
| *PROCR* | -1.316160303 | 0.330209505 | -3.985834091 | 6.72E-05 | 0.000498632 |
| *AJUBA* | -1.323668142 | 0.329819577 | -4.013309809 | 5.99E-05 | 0.000451811 |
| *MMRN1* | -1.332305892 | 0.465263115 | -2.863553652 | 0.004189179 | 0.015515694 |
| *VPREB1* | -1.333736015 | 0.546681867 | -2.439693166 | 0.014699741 | 0.043366568 |
| *SV2A* | -1.334231292 | 0.319997047 | -4.169511262 | 3.05E-05 | 0.000252235 |
| *TPBG* | -1.335266628 | 0.467429308 | -2.856617259 | 0.004281818 | 0.015810712 |
| *ASIC1* | -1.337796641 | 0.409932747 | -3.263453948 | 0.001100631 | 0.005156366 |
| *VWCE* | -1.339940845 | 0.30522347 | -4.390032142 | 1.13E-05 | 0.000109215 |
| *CCDC188* | -1.339953556 | 0.451377704 | -2.968586049 | 0.002991733 | 0.011817608 |
| *ENPP3* | -1.340283372 | 0.422275389 | -3.173955685 | 0.001503766 | 0.006700961 |
| *TSPOAP1* | -1.341265206 | 0.298288911 | -4.496530573 | 6.91E-06 | 7.15E-05 |
| *SFRP5* | -1.343810799 | 0.55946425 | -2.40196009 | 0.016307488 | 0.047092668 |
| *CFAP61* | -1.346977987 | 0.439396423 | -3.06551878 | 0.002172927 | 0.009073498 |
| *SLC16A9* | -1.348004597 | 0.548551137 | -2.457390946 | 0.013995029 | 0.04171233 |
| *COL23A1* | -1.3490177 | 0.436366181 | -3.091480871 | 0.001991608 | 0.008453622 |
| *KLHL23* | -1.353194829 | 0.289042748 | -4.681642549 | 2.85E-06 | 3.34E-05 |
| *CENPJ* | -1.356130236 | 0.17520418 | -7.740284729 | 9.92E-15 | 8.16E-13 |
| *DOC2A* | -1.358406559 | 0.340214107 | -3.992799043 | 6.53E-05 | 0.00048557 |
| *WASF1* | -1.358985214 | 0.306172725 | -4.438622722 | 9.05E-06 | 9.00E-05 |
| *CIB3* | -1.360760646 | 0.363511522 | -3.743376926 | 0.000181564 | 0.001154827 |
| *SMYD3* | -1.36152473 | 0.261092302 | -5.214725672 | 1.84E-07 | 3.07E-06 |
| *CLEC11A* | -1.361955729 | 0.389974134 | -3.492425808 | 0.000478655 | 0.002613815 |
| *LCA5L* | -1.363315252 | 0.278899004 | -4.888204096 | 1.02E-06 | 1.37E-05 |
| *RIMBP3C* | -1.363416022 | 0.425257921 | -3.206092008 | 0.00134551 | 0.006117112 |
| *ALDH5A1* | -1.363573934 | 0.273462374 | -4.986331081 | 6.15E-07 | 8.80E-06 |
| *HDC* | -1.367630111 | 0.498688586 | -2.742453207 | 0.006098214 | 0.021206751 |
| *KCNMB4* | -1.367958649 | 0.391083012 | -3.497872847 | 0.000468985 | 0.002572103 |
| *RNF183* | -1.368205558 | 0.352911166 | -3.876912062 | 0.000105791 | 0.000732802 |
| *ZNF114* | -1.373070211 | 0.321188927 | -4.274961225 | 1.91E-05 | 0.000170135 |
| *RSPO1* | -1.374297556 | 0.472230089 | -2.910228693 | 0.003611644 | 0.013773063 |
| *CDR2L* | -1.374633637 | 0.399640999 | -3.439671209 | 0.000582421 | 0.003060612 |
| *PLCH1* | -1.37516964 | 0.446731739 | -3.078289539 | 0.002081925 | 0.008768684 |
| *TBX1* | -1.376517416 | 0.435438739 | -3.161219463 | 0.001571101 | 0.006941349 |
| *NRXN2* | -1.376704219 | 0.412278118 | -3.339260952 | 0.000840016 | 0.004128482 |
| *ENPP7* | -1.377886853 | 0.52891039 | -2.605142346 | 0.00918361 | 0.029743846 |
| *NDFIP2* | -1.378788034 | 0.372759786 | -3.698864754 | 0.000216566 | 0.001336898 |
| *PRRT2* | -1.379248856 | 0.38061838 | -3.623705344 | 0.000290412 | 0.00171504 |
| *MAST1* | -1.379639702 | 0.297827239 | -4.632348968 | 3.62E-06 | 4.10E-05 |
| *SPSB4* | -1.384208914 | 0.556361346 | -2.487967441 | 0.012847549 | 0.038882106 |
| *ANKRD13B* | -1.384586116 | 0.277628167 | -4.987196108 | 6.13E-07 | 8.77E-06 |
| *TNFRSF4* | -1.388657452 | 0.403854002 | -3.438513535 | 0.000584917 | 0.003069518 |
| *ADGRG5* | -1.389886555 | 0.328533206 | -4.230581662 | 2.33E-05 | 0.000200779 |
| *FAM171A2* | -1.390370203 | 0.30775003 | -4.517855628 | 6.25E-06 | 6.56E-05 |
| *INKA1* | -1.39116061 | 0.273147928 | -5.093066685 | 3.52E-07 | 5.39E-06 |
| *KCNJ12* | -1.393779758 | 0.558030062 | -2.497678625 | 0.012500947 | 0.038032674 |
| *GYG2* | -1.394456636 | 0.4344189 | -3.209935474 | 0.001327648 | 0.006045457 |
| *DDC* | -1.396357053 | 0.482123298 | -2.896265455 | 0.003776328 | 0.014260222 |
| *UROC1* | -1.401644794 | 0.430113136 | -3.258781651 | 0.001118917 | 0.005225797 |
| *ZNF454* | -1.402498282 | 0.543615347 | -2.579946076 | 0.009881574 | 0.031577945 |
| *CEP70* | -1.40481735 | 0.286495286 | -4.903457113 | 9.42E-07 | 1.29E-05 |
| *MAMDC2* | -1.409653206 | 0.487082249 | -2.894076325 | 0.003802757 | 0.014344623 |
| *AMN* | -1.409673243 | 0.385602014 | -3.65577251 | 0.000256409 | 0.001545482 |
| *TMPRSS9* | -1.409699158 | 0.341371904 | -4.129511369 | 3.64E-05 | 0.000294023 |
| *DST* | -1.411113111 | 0.352162022 | -4.006999689 | 6.15E-05 | 0.000461628 |
| *GPR135* | -1.419332706 | 0.287164412 | -4.942578703 | 7.71E-07 | 1.08E-05 |
| *ZNF711* | -1.419422076 | 0.497788763 | -2.851454637 | 0.00435197 | 0.016028615 |
| *DPY19L2* | -1.419589899 | 0.376113094 | -3.774369793 | 0.000160413 | 0.001043572 |
| *C1QTNF4* | -1.421221996 | 0.454452444 | -3.127328315 | 0.001764028 | 0.00764541 |
| *SFTPB* | -1.421275197 | 0.511485675 | -2.778719455 | 0.005457364 | 0.019352336 |
| *ZNF221* | -1.421877063 | 0.21588542 | -6.586257943 | 4.51E-11 | 1.84E-09 |
| *DCLK2* | -1.421937922 | 0.501842837 | -2.833432735 | 0.0046051 | 0.016788935 |
| *TAS2R3* | -1.422278147 | 0.343510388 | -4.140422517 | 3.47E-05 | 0.000281893 |
| *CRABP1* | -1.423767287 | 0.516494837 | -2.756595393 | 0.005840657 | 0.020439897 |
| *C2orf66* | -1.424119883 | 0.569118435 | -2.502326046 | 0.012338024 | 0.037643741 |
| *DUOXA1* | -1.424608408 | 0.589542958 | -2.416462431 | 0.015672143 | 0.045674588 |
| *FST* | -1.431636566 | 0.596126695 | -2.401564263 | 0.016325141 | 0.047126452 |
| *DYTN* | -1.432157393 | 0.41075901 | -3.486612247 | 0.00048918 | 0.002660736 |
| *SLC29A4* | -1.434235749 | 0.412187319 | -3.479572718 | 0.000502214 | 0.002718087 |
| *LTC4S* | -1.434467464 | 0.278475785 | -5.151138949 | 2.59E-07 | 4.14E-06 |
| *TSPAN10* | -1.435604301 | 0.283919559 | -5.056376905 | 4.27E-07 | 6.40E-06 |
| *AGAP9* | -1.437654302 | 0.268533397 | -5.353726263 | 8.62E-08 | 1.56E-06 |
| *KRT18* | -1.437751739 | 0.437514532 | -3.286180538 | 0.001015559 | 0.004825658 |
| *MICU3* | -1.438146575 | 0.429271499 | -3.350202794 | 0.000807524 | 0.003995465 |
| *SLC35F3* | -1.438554189 | 0.554137178 | -2.5960254 | 0.009430909 | 0.03037079 |
| *SVOPL* | -1.439406444 | 0.453667651 | -3.172821428 | 0.001509653 | 0.006720943 |
| *BCL2* | -1.441716243 | 0.183666328 | -7.849649202 | 4.17E-15 | 3.70E-13 |
| *SLC22A11* | -1.442964067 | 0.543171843 | -2.656551672 | 0.007894435 | 0.026212108 |
| *PDZD2* | -1.445191796 | 0.539923235 | -2.676661609 | 0.007435969 | 0.024948919 |
| *AVPR1B* | -1.452976625 | 0.543218511 | -2.674755364 | 0.00747838 | 0.025069956 |
| *ZBTB12* | -1.454927237 | 0.262276426 | -5.547304649 | 2.90E-08 | 5.98E-07 |
| *CFAP69* | -1.460693146 | 0.391595752 | -3.730104679 | 0.0001914 | 0.001207928 |
| *ZC3H12B* | -1.461137424 | 0.497099931 | -2.939323325 | 0.003289297 | 0.012776802 |
| *LRRC17* | -1.464530705 | 0.384926208 | -3.804705098 | 0.000141973 | 0.00094161 |
| *CD8B2* | -1.465254348 | 0.450939014 | -3.249340385 | 0.00115673 | 0.005373853 |
| *LTBP1* | -1.467977716 | 0.478759194 | -3.066213109 | 0.002167887 | 0.009058427 |
| *FBXW10* | -1.471045717 | 0.534654408 | -2.751395472 | 0.005934195 | 0.020720593 |
| *CYSLTR2* | -1.47110681 | 0.352224253 | -4.17661986 | 2.96E-05 | 0.000245637 |
| *COCH* | -1.472738175 | 0.329140544 | -4.474496383 | 7.66E-06 | 7.81E-05 |
| *RLN1* | -1.473036639 | 0.355578269 | -4.142650909 | 3.43E-05 | 0.000279454 |
| *MANEAL* | -1.475616576 | 0.345041192 | -4.276638877 | 1.90E-05 | 0.000169048 |
| *ARMH4* | -1.477213895 | 0.479654939 | -3.079742904 | 0.002071794 | 0.008732982 |
| *GXYLT2* | -1.478341424 | 0.587958743 | -2.514362515 | 0.011924779 | 0.036674516 |
| *DIO2* | -1.483333326 | 0.552616128 | -2.68420202 | 0.007270316 | 0.024502196 |
| *ALOX12B* | -1.485173551 | 0.380298736 | -3.905281321 | 9.41E-05 | 0.000663105 |
| *SCN11A* | -1.48605262 | 0.352688302 | -4.213501307 | 2.51E-05 | 0.000214141 |
| *EPDR1* | -1.487235842 | 0.308839548 | -4.815561522 | 1.47E-06 | 1.88E-05 |
| *CD96* | -1.487505535 | 0.431763072 | -3.445189343 | 0.00057066 | 0.0030108 |
| *XKR9* | -1.487734773 | 0.399092854 | -3.727791056 | 0.000193165 | 0.001217854 |
| *GPR21* | -1.490444877 | 0.454430697 | -3.279806775 | 0.001038782 | 0.004915349 |
| *NYNRIN* | -1.491178652 | 0.368288507 | -4.048941588 | 5.14E-05 | 0.000396465 |
| *CBLN1* | -1.491188235 | 0.554812171 | -2.687735263 | 0.007193841 | 0.024278801 |
| *PIFO* | -1.492443172 | 0.353804788 | -4.21826731 | 2.46E-05 | 0.000210459 |
| *KIAA1549* | -1.49297654 | 0.337738573 | -4.420509405 | 9.85E-06 | 9.69E-05 |
| *CCDC87* | -1.493674443 | 0.288854039 | -5.171035344 | 2.33E-07 | 3.76E-06 |
| *SLCO2A1* | -1.495120949 | 0.617539134 | -2.421095065 | 0.01547383 | 0.045204832 |
| *SPINK5* | -1.49768296 | 0.554294127 | -2.701964329 | 0.006893116 | 0.023425493 |
| *CASKIN2* | -1.510502039 | 0.305911867 | -4.937703317 | 7.90E-07 | 1.10E-05 |
| *FGF11* | -1.516171721 | 0.297296453 | -5.099864822 | 3.40E-07 | 5.22E-06 |
| *EBF4* | -1.518552377 | 0.368677775 | -4.118914891 | 3.81E-05 | 0.000305687 |
| *RTKN* | -1.519587994 | 0.298072128 | -5.098054627 | 3.43E-07 | 5.27E-06 |
| *GPR63* | -1.522064334 | 0.538928098 | -2.824243788 | 0.004739232 | 0.017194858 |
| *SPTLC3* | -1.524666114 | 0.393686523 | -3.872792242 | 0.000107596 | 0.000743515 |
| *IRX5* | -1.527519005 | 0.633417728 | -2.411550762 | 0.01588484 | 0.046183919 |
| *COL27A1* | -1.532767511 | 0.451283693 | -3.396461101 | 0.000682633 | 0.003480898 |
| *PRSS12* | -1.53350587 | 0.62596439 | -2.449829246 | 0.014292398 | 0.042418914 |
| *ANGPT1* | -1.535548557 | 0.457415175 | -3.357012711 | 0.000787895 | 0.003916094 |
| *BICC1* | -1.53773831 | 0.627448354 | -2.450780691 | 0.014254677 | 0.042330776 |
| *ADCY1* | -1.541557056 | 0.528283536 | -2.918048647 | 0.003522294 | 0.013494099 |
| *STYK1* | -1.542313352 | 0.366123044 | -4.212554707 | 2.52E-05 | 0.00021481 |
| *PCDHGA3* | -1.544634182 | 0.581470893 | -2.656425626 | 0.007897387 | 0.02621366 |
| *MST1* | -1.546109092 | 0.296928788 | -5.207003009 | 1.92E-07 | 3.18E-06 |
| *FGF17* | -1.548653041 | 0.406968899 | -3.805335114 | 0.000141612 | 0.000939827 |
| *DNAH3* | -1.550806732 | 0.486601292 | -3.187017292 | 0.001437482 | 0.006459657 |
| *IRAK1BP1* | -1.550945678 | 0.236214597 | -6.56583335 | 5.17E-11 | 2.08E-09 |
| *SPINK4* | -1.551706165 | 0.572060223 | -2.712487431 | 0.006678031 | 0.022831814 |
| *ZNF610* | -1.552931482 | 0.299033243 | -5.193173387 | 2.07E-07 | 3.39E-06 |
| *NDST3* | -1.556199251 | 0.623115447 | -2.497449323 | 0.012509034 | 0.038049965 |
| *NKD1* | -1.564683401 | 0.383426177 | -4.080794418 | 4.49E-05 | 0.000352903 |
| *ZNF521* | -1.56562918 | 0.538201595 | -2.909001373 | 0.003625853 | 0.013817262 |
| *FUT6* | -1.566854622 | 0.505539767 | -3.099369669 | 0.001939329 | 0.008265341 |
| *CTSW* | -1.567735108 | 0.274055577 | -5.720500649 | 1.06E-08 | 2.43E-07 |
| *EMID1* | -1.57174341 | 0.333826049 | -4.708270712 | 2.50E-06 | 2.98E-05 |
| *PLXNB1* | -1.578196439 | 0.340443817 | -4.635703039 | 3.56E-06 | 4.05E-05 |
| *IGSF10* | -1.578267837 | 0.575206751 | -2.743827042 | 0.006072752 | 0.021132137 |
| *FAM81B* | -1.579264581 | 0.355795872 | -4.438681574 | 9.05E-06 | 9.00E-05 |
| *OBSL1* | -1.579782143 | 0.377360015 | -4.186405771 | 2.83E-05 | 0.000236778 |
| *CBX2* | -1.580258589 | 0.327544062 | -4.824567969 | 1.40E-06 | 1.81E-05 |
| *CDK6* | -1.584667957 | 0.234333564 | -6.762445505 | 1.36E-11 | 6.24E-10 |
| *ZCCHC18* | -1.586174183 | 0.329389721 | -4.815493879 | 1.47E-06 | 1.88E-05 |
| *RCOR2* | -1.586954339 | 0.393040472 | -4.037635952 | 5.40E-05 | 0.000413347 |
| *NANOS1* | -1.587172754 | 0.395623495 | -4.011826327 | 6.03E-05 | 0.000454119 |
| *CNNM1* | -1.589052092 | 0.535787663 | -2.965824341 | 0.003018729 | 0.011900482 |
| *NFATC4* | -1.593824319 | 0.553912307 | -2.877394667 | 0.004009738 | 0.014996722 |
| *PCDHGA10* | -1.596387188 | 0.483806524 | -3.299639648 | 0.00096809 | 0.004646834 |
| *KIAA1614* | -1.602235971 | 0.481293039 | -3.329023776 | 0.00087151 | 0.004258143 |
| *SLC45A3* | -1.605809943 | 0.309494486 | -5.188492902 | 2.12E-07 | 3.47E-06 |
| *SKIDA1* | -1.607861707 | 0.510113176 | -3.151970549 | 0.001621726 | 0.007132171 |
| *GOLGA8S* | -1.611283162 | 0.317411328 | -5.076325316 | 3.85E-07 | 5.83E-06 |
| *DBNDD1* | -1.615895159 | 0.372591004 | -4.336914047 | 1.44E-05 | 0.000134377 |
| *TGM5* | -1.61801125 | 0.386376457 | -4.187654861 | 2.82E-05 | 0.00023579 |
| *ZSCAN31* | -1.618605137 | 0.274259238 | -5.901734235 | 3.60E-09 | 9.30E-08 |
| *NPTX1* | -1.621913544 | 0.539367633 | -3.007065024 | 0.002637833 | 0.010648209 |
| *GUCY2D* | -1.621989213 | 0.379668316 | -4.272121597 | 1.94E-05 | 0.00017193 |
| *TMEM74* | -1.636329973 | 0.606943289 | -2.696017903 | 0.00701739 | 0.02377381 |
| *MED12L* | -1.636930164 | 0.447322609 | -3.6593951 | 0.000252811 | 0.001526706 |
| *IGFBP2* | -1.639583364 | 0.483840423 | -3.388686195 | 0.000702283 | 0.003558752 |
| *SULT1C4* | -1.639618466 | 0.414951795 | -3.951346844 | 7.77E-05 | 0.000565607 |
| *ENPP6* | -1.640255737 | 0.661035739 | -2.481341991 | 0.013088873 | 0.039457323 |
| *GRIP1* | -1.640318555 | 0.428668803 | -3.826540542 | 0.000129957 | 0.000871961 |
| *EMILIN1* | -1.640485035 | 0.411955555 | -3.982189375 | 6.83E-05 | 0.000505396 |
| *CDH24* | -1.64235748 | 0.26573802 | -6.180363197 | 6.40E-10 | 2.00E-08 |
| *TMEM221* | -1.642459759 | 0.551516753 | -2.97807773 | 0.002900624 | 0.011529653 |
| *NPM2* | -1.643645876 | 0.363668433 | -4.519627564 | 6.19E-06 | 6.51E-05 |
| *PCDHGC5* | -1.644499412 | 0.500547132 | -3.285403724 | 0.001018363 | 0.004836806 |
| *SEMA3G* | -1.653950822 | 0.377722301 | -4.378748135 | 1.19E-05 | 0.000114052 |
| *MPO* | -1.6586661 | 0.482284055 | -3.439189171 | 0.000583459 | 0.003064033 |
| *PPP1R27* | -1.659692941 | 0.505225739 | -3.28505223 | 0.001019635 | 0.004841597 |
| *RARB* | -1.66036973 | 0.680663525 | -2.439339953 | 0.014714119 | 0.043397945 |
| *PTPN14* | -1.662629 | 0.490953673 | -3.386529302 | 0.000707827 | 0.003579961 |
| *CAPSL* | -1.662772224 | 0.592426206 | -2.80671619 | 0.005004932 | 0.018012291 |
| *SERPINE1* | -1.665427038 | 0.369603804 | -4.505979157 | 6.61E-06 | 6.88E-05 |
| *ERVFRD-1* | -1.666290642 | 0.454850134 | -3.663383862 | 0.000248905 | 0.001505702 |
| *NAV3* | -1.666961539 | 0.656183449 | -2.540389491 | 0.011072908 | 0.034554808 |
| *PRPH* | -1.672749014 | 0.485883853 | -3.442693157 | 0.000575952 | 0.003033164 |
| *PALM* | -1.673088367 | 0.358962171 | -4.660904415 | 3.15E-06 | 3.66E-05 |
| *ADRA2A* | -1.675000873 | 0.419358712 | -3.994195959 | 6.49E-05 | 0.000483284 |
| *CYP2E1* | -1.682352388 | 0.407171409 | -4.131803832 | 3.60E-05 | 0.000291552 |
| *IRGM* | -1.683592937 | 0.33141812 | -5.079966476 | 3.78E-07 | 5.73E-06 |
| *IRX3* | -1.686487905 | 0.664055336 | -2.539679775 | 0.011095401 | 0.034604532 |
| *PRSS57* | -1.688333045 | 0.335194845 | -5.036870562 | 4.73E-07 | 7.00E-06 |
| *GNB3* | -1.689367322 | 0.396866116 | -4.256768852 | 2.07E-05 | 0.000182226 |
| *VASH2* | -1.690157263 | 0.43158683 | -3.916146522 | 9.00E-05 | 0.000638784 |
| *APOC2* | -1.695300604 | 0.580706344 | -2.919376757 | 0.00350732 | 0.013451386 |
| *PPP1R1C* | -1.698239459 | 0.592433688 | -2.866547758 | 0.004149755 | 0.015409344 |
| *CYP26C1* | -1.700572338 | 0.657275353 | -2.587305807 | 0.00967297 | 0.0310335 |
| *FTO* | -1.701370709 | 0.326147493 | -5.216568409 | 1.82E-07 | 3.05E-06 |
| *FAM227A* | -1.703132217 | 0.397630501 | -4.283203155 | 1.84E-05 | 0.000164738 |
| *CFAP46* | -1.708183116 | 0.64077144 | -2.665822802 | 0.007680016 | 0.0256292 |
| *SPATA9* | -1.709975318 | 0.415491523 | -4.11554803 | 3.86E-05 | 0.000309478 |
| *UNC79* | -1.71376616 | 0.321932976 | -5.323363205 | 1.02E-07 | 1.82E-06 |
| *SRPK3* | -1.714523417 | 0.486743897 | -3.522434339 | 0.000427603 | 0.002376439 |
| *LRRC74B* | -1.716774135 | 0.660126304 | -2.600675241 | 0.009304049 | 0.030050909 |
| *ZSCAN23* | -1.717381713 | 0.463312974 | -3.706742118 | 0.000209943 | 0.001303569 |
| *FRMD4A* | -1.721070987 | 0.462680317 | -3.719784316 | 0.000199393 | 0.001250393 |
| *NOG* | -1.724040691 | 0.470227832 | -3.666394407 | 0.000245994 | 0.001492372 |
| *COL4A5* | -1.724187788 | 0.610775812 | -2.822947067 | 0.004758443 | 0.017254669 |
| *PCDHGC4* | -1.724460594 | 0.554412115 | -3.110430934 | 0.001868146 | 0.00802089 |
| *GLIS3* | -1.72671313 | 0.57168174 | -3.020409799 | 0.002524329 | 0.010284447 |
| *RTN4R* | -1.730201837 | 0.372062688 | -4.650296562 | 3.31E-06 | 3.80E-05 |
| *TMEM125* | -1.733221322 | 0.705569275 | -2.456486391 | 0.014030312 | 0.041797811 |
| *TPSD1* | -1.733593099 | 0.53562293 | -3.236592391 | 0.001209661 | 0.005578007 |
| *FILIP1* | -1.734041221 | 0.49117872 | -3.530367156 | 0.000414983 | 0.002320564 |
| *IL5RA* | -1.74994847 | 0.374518132 | -4.67253337 | 2.98E-06 | 3.47E-05 |
| *FER1L6* | -1.749970893 | 0.450586787 | -3.883759895 | 0.000102853 | 0.000714647 |
| *AADAT* | -1.750106017 | 0.615637415 | -2.842754475 | 0.004472551 | 0.016383224 |
| *KRT2* | -1.750763184 | 0.417212376 | -4.196335692 | 2.71E-05 | 0.000227839 |
| *KCTD19* | -1.752707284 | 0.444353425 | -3.94439918 | 8.00E-05 | 0.00058058 |
| *GJC1* | -1.755127552 | 0.449046912 | -3.908561675 | 9.28E-05 | 0.000656945 |
| *TRH* | -1.760754827 | 0.633907272 | -2.777622065 | 0.005475827 | 0.019402594 |
| *SHROOM3* | -1.767882133 | 0.734238235 | -2.407777269 | 0.01604997 | 0.046499922 |
| *S100B* | -1.772895623 | 0.453018169 | -3.913519909 | 9.10E-05 | 0.000645325 |
| *COL14A1* | -1.776222407 | 0.423082667 | -4.19828687 | 2.69E-05 | 0.000226486 |
| *TNFSF4* | -1.777322597 | 0.318428543 | -5.581542973 | 2.38E-08 | 5.02E-07 |
| *MMP2* | -1.778972746 | 0.52368194 | -3.397048115 | 0.00068117 | 0.003474559 |
| *FLNC* | -1.780808439 | 0.565890906 | -3.146911214 | 0.001650051 | 0.007234626 |
| *SLC16A2* | -1.790755093 | 0.68543616 | -2.612577503 | 0.008986231 | 0.0291852 |
| *GPT2* | -1.793922485 | 0.289034487 | -6.206603592 | 5.41E-10 | 1.72E-08 |
| *STXBP1* | -1.79436482 | 0.367377194 | -4.884257511 | 1.04E-06 | 1.40E-05 |
| *SLC24A3* | -1.804461292 | 0.483294169 | -3.733670731 | 0.000188709 | 0.001194039 |
| *AGRN* | -1.808983979 | 0.357961731 | -5.053568085 | 4.34E-07 | 6.48E-06 |
| *ITGB8* | -1.81287052 | 0.489973342 | -3.699937049 | 0.000215653 | 0.001332041 |
| *TEKT1* | -1.815631307 | 0.577498554 | -3.143958189 | 0.001666793 | 0.00729288 |
| *ACY3* | -1.8172591 | 0.511249545 | -3.554544189 | 0.000378635 | 0.002147656 |
| *PCDHGA2* | -1.819304884 | 0.516401247 | -3.523045106 | 0.000426619 | 0.002373053 |
| *KIAA1217* | -1.825877809 | 0.662149027 | -2.757502822 | 0.005824471 | 0.020393702 |
| *GCSAML* | -1.8262398 | 0.425522742 | -4.29175605 | 1.77E-05 | 0.000159418 |
| *COLGALT2* | -1.827618544 | 0.429353031 | -4.256680198 | 2.07E-05 | 0.000182226 |
| *NEGR1* | -1.833077474 | 0.451324923 | -4.061547195 | 4.87E-05 | 0.000378696 |
| *USP44* | -1.83428745 | 0.602979334 | -3.042040327 | 0.002349804 | 0.009681817 |
| *DPYSL3* | -1.838906172 | 0.492784007 | -3.731667722 | 0.000190216 | 0.001202134 |
| *MAP1A* | -1.83911626 | 0.392882914 | -4.681079769 | 2.85E-06 | 3.35E-05 |
| *MEX3B* | -1.842019877 | 0.309109167 | -5.959124068 | 2.54E-09 | 6.87E-08 |
| *CAV2* | -1.847287342 | 0.544343773 | -3.393604251 | 0.000689793 | 0.003508932 |
| *MYOZ3* | -1.851399081 | 0.347925435 | -5.321252476 | 1.03E-07 | 1.84E-06 |
| *MKRN3* | -1.852774148 | 0.587308651 | -3.15468561 | 0.001606711 | 0.007076975 |
| *RTL3* | -1.862698319 | 0.445379062 | -4.18227635 | 2.89E-05 | 0.000240486 |
| *ITM2A* | -1.86624192 | 0.385483001 | -4.841307956 | 1.29E-06 | 1.68E-05 |
| *PRODH* | -1.870583455 | 0.572085029 | -3.269764739 | 0.00107637 | 0.005059177 |
| *STEAP1* | -1.877175451 | 0.691031452 | -2.716483377 | 0.006597951 | 0.022618845 |
| *ONECUT2* | -1.88963703 | 0.716344379 | -2.637889101 | 0.008342386 | 0.027426168 |
| *ZNF474* | -1.892536002 | 0.393307483 | -4.811848451 | 1.50E-06 | 1.91E-05 |
| *S100A1* | -1.895154083 | 0.492036871 | -3.851650542 | 0.000117324 | 0.000800263 |
| *PLPPR3* | -1.896997789 | 0.400495286 | -4.73662951 | 2.17E-06 | 2.64E-05 |
| *MGAT5B* | -1.897707031 | 0.543165281 | -3.493792952 | 0.00047621 | 0.002602263 |
| *IL9R* | -1.900281866 | 0.370596758 | -5.127626792 | 2.93E-07 | 4.61E-06 |
| *WT1* | -1.900592298 | 0.427643636 | -4.444336682 | 8.82E-06 | 8.80E-05 |
| *GLYATL2* | -1.902931181 | 0.597017979 | -3.187393424 | 0.001435614 | 0.00645445 |
| *P3H3* | -1.908466099 | 0.379984746 | -5.022480817 | 5.10E-07 | 7.50E-06 |
| *AR* | -1.909754854 | 0.576831211 | -3.310768937 | 0.0009304 | 0.004497975 |
| *NME5* | -1.9109801 | 0.619446876 | -3.084978186 | 0.002035671 | 0.008610529 |
| *GATA2* | -1.913147307 | 0.332698322 | -5.750396619 | 8.90E-09 | 2.07E-07 |
| *SERPING1* | -1.919502142 | 0.359591432 | -5.338008564 | 9.40E-08 | 1.69E-06 |
| *DCDC1* | -1.927317111 | 0.682222278 | -2.825057422 | 0.004727214 | 0.017165308 |
| *GLP1R* | -1.932085889 | 0.796880157 | -2.424562679 | 0.015326837 | 0.044858206 |
| *F2* | -1.932707788 | 0.576814483 | -3.350657527 | 0.000806199 | 0.003991406 |
| *SEZ6L2* | -1.93499536 | 0.555214997 | -3.48512805 | 0.000491902 | 0.002672783 |
| *IL12RB2* | -1.938944455 | 0.58100062 | -3.337250233 | 0.000846117 | 0.004153953 |
| *MUC4* | -1.942908997 | 0.753323322 | -2.579117016 | 0.009905323 | 0.031641059 |
| *RGS9BP* | -1.943817072 | 0.353376577 | -5.500695859 | 3.78E-08 | 7.55E-07 |
| *HSPG2* | -1.944371068 | 0.306631162 | -6.341074592 | 2.28E-10 | 8.11E-09 |
| *CA5A* | -1.954009404 | 0.661741102 | -2.952830642 | 0.003148747 | 0.012328312 |
| *LTK* | -1.960970322 | 0.402522781 | -4.87170022 | 1.11E-06 | 1.47E-05 |
| *CHRNA6* | -1.968267049 | 0.620506835 | -3.172031217 | 0.001513767 | 0.006735041 |
| *TTLL6* | -1.973496785 | 0.565906409 | -3.487320081 | 0.000487887 | 0.002655528 |
| *OR1J4* | -1.98042926 | 0.779386391 | -2.54101083 | 0.01105325 | 0.034496862 |
| *KRT17* | -1.983202575 | 0.633994541 | -3.128106705 | 0.001759363 | 0.007628324 |
| *SSPN* | -1.985165527 | 0.377830844 | -5.254111881 | 1.49E-07 | 2.53E-06 |
| *ARHGEF25* | -1.988283577 | 0.491211545 | -4.047713448 | 5.17E-05 | 0.000397973 |
| *DAPL1* | -2.001822781 | 0.608203672 | -3.291369113 | 0.00099701 | 0.004751784 |
| *WDR86* | -2.004545281 | 0.516320305 | -3.882367709 | 0.000103444 | 0.000718121 |
| *WNT8A* | -2.008474368 | 0.679370826 | -2.956374177 | 0.003112792 | 0.012210558 |
| *ARSL* | -2.009799559 | 0.583357388 | -3.445228605 | 0.000570577 | 0.0030108 |
| *OR1Q1* | -2.019773359 | 0.783003296 | -2.57952089 | 0.009893747 | 0.031610463 |
| *PCDHB13* | -2.024479436 | 0.593250012 | -3.412523214 | 0.000643644 | 0.003317898 |
| *SCN2A* | -2.036388036 | 0.763882692 | -2.66583869 | 0.007679653 | 0.0256292 |
| *NTN3* | -2.037849364 | 0.572183368 | -3.561531978 | 0.000368697 | 0.002100698 |
| *MMP15* | -2.045772536 | 0.430867694 | -4.74802953 | 2.05E-06 | 2.52E-05 |
| *GPR153* | -2.048233629 | 0.396571305 | -5.164855861 | 2.41E-07 | 3.87E-06 |
| *SP7* | -2.049391303 | 0.52763012 | -3.884143883 | 0.000102691 | 0.000713832 |
| *PCDHB14* | -2.050977504 | 0.591615803 | -3.466738878 | 0.000526813 | 0.002822272 |
| *CHD5* | -2.053597011 | 0.372431327 | -5.514028653 | 3.51E-08 | 7.04E-07 |
| *OR14L1P* | -2.055903948 | 0.520176825 | -3.952317457 | 7.74E-05 | 0.000563682 |
| *CYTL1* | -2.05834349 | 0.45384634 | -4.535331249 | 5.75E-06 | 6.10E-05 |
| *BCL2L10* | -2.059300971 | 0.487762118 | -4.22193708 | 2.42E-05 | 0.000207666 |
| *WNT9A* | -2.059920569 | 0.556153231 | -3.703872338 | 0.000212333 | 0.001315643 |
| *PAGE5* | -2.062868874 | 0.834802004 | -2.471087592 | 0.013470282 | 0.040407017 |
| *AEBP1* | -2.066486184 | 0.412371895 | -5.011219744 | 5.41E-07 | 7.89E-06 |
| *SFRP4* | -2.071767548 | 0.434870116 | -4.764106503 | 1.90E-06 | 2.35E-05 |
| *SYDE1* | -2.071868341 | 0.574313244 | -3.607558006 | 0.000309092 | 0.001806282 |
| *KLHL13* | -2.072048222 | 0.438747127 | -4.722647958 | 2.33E-06 | 2.81E-05 |
| *PCDHGA1* | -2.072300876 | 0.472530729 | -4.385536751 | 1.16E-05 | 0.000110988 |
| *BHLHE41* | -2.074296449 | 0.391083671 | -5.303971002 | 1.13E-07 | 1.99E-06 |
| *ADAMTSL2* | -2.075399638 | 0.575046431 | -3.609099239 | 0.000307262 | 0.001798907 |
| *PRSS45P* | -2.083349397 | 0.460863283 | -4.520536726 | 6.17E-06 | 6.49E-05 |
| *IGLL1* | -2.094584306 | 0.487921038 | -4.292875577 | 1.76E-05 | 0.000158752 |
| *EDA2R* | -2.095391969 | 0.387685823 | -5.404871273 | 6.49E-08 | 1.21E-06 |
| *SRGAP3* | -2.103165343 | 0.346823512 | -6.064079488 | 1.33E-09 | 3.82E-08 |
| *GRM7* | -2.110849584 | 0.678728824 | -3.110004333 | 0.001870846 | 0.008028128 |
| *PTGER1* | -2.110884798 | 0.551400203 | -3.828226372 | 0.00012907 | 0.000867851 |
| *SAMD11* | -2.111929506 | 0.497456083 | -4.245459207 | 2.18E-05 | 0.000189715 |
| *GAREM1* | -2.117016883 | 0.506832471 | -4.176955906 | 2.95E-05 | 0.000245404 |
| *CAMSAP3* | -2.121953327 | 0.471203956 | -4.503258731 | 6.69E-06 | 6.95E-05 |
| *TEKT2* | -2.125018213 | 0.401392803 | -5.294111398 | 1.20E-07 | 2.09E-06 |
| *SERTM2* | -2.128131799 | 0.607670653 | -3.502113829 | 0.000461582 | 0.002538543 |
| *SERTM1* | -2.128946623 | 0.627022478 | -3.395327438 | 0.000685466 | 0.00349197 |
| *KCNJ11* | -2.134723508 | 0.456217965 | -4.679174588 | 2.88E-06 | 3.38E-05 |
| *ADAMTS7* | -2.136642511 | 0.554927818 | -3.85030709 | 0.00011797 | 0.000803454 |
| *FGF5* | -2.137544387 | 0.716301114 | -2.984142205 | 0.002843746 | 0.01133488 |
| *TEX101* | -2.141309032 | 0.81440375 | -2.629296627 | 0.008556169 | 0.027987134 |
| *METTL24* | -2.163858128 | 0.563157786 | -3.842365643 | 0.000121854 | 0.00082653 |
| *ARHGEF10* | -2.164733886 | 0.40582426 | -5.334165792 | 9.60E-08 | 1.72E-06 |
| *ZNF385C* | -2.167688595 | 0.333562259 | -6.498602688 | 8.11E-11 | 3.14E-09 |
| *BEND6* | -2.180137779 | 0.438119391 | -4.976127106 | 6.49E-07 | 9.22E-06 |
| *IGDCC4* | -2.180392681 | 0.448639017 | -4.860015737 | 1.17E-06 | 1.55E-05 |
| *CTXN1* | -2.184830664 | 0.403417399 | -5.415806724 | 6.10E-08 | 1.15E-06 |
| *RASGRP3* | -2.194233673 | 0.3191014 | -6.876289712 | 6.14E-12 | 3.02E-10 |
| *TNFRSF11B* | -2.194451338 | 0.618244247 | -3.5494893 | 0.000385979 | 0.002183054 |
| *SIGLEC6* | -2.199311937 | 0.453551083 | -4.849094228 | 1.24E-06 | 1.62E-05 |
| *CYP2F1* | -2.203362129 | 0.519615391 | -4.240371177 | 2.23E-05 | 0.000193377 |
| *KCNH1* | -2.212890261 | 0.915975439 | -2.41588384 | 0.015697068 | 0.045734596 |
| *DUOX1* | -2.21612723 | 0.648146112 | -3.419178467 | 0.000628105 | 0.003254755 |
| *GASK1A* | -2.221067837 | 0.518084047 | -4.287080159 | 1.81E-05 | 0.000162395 |
| *SLC7A3* | -2.225006466 | 0.860085985 | -2.58695817 | 0.009682735 | 0.031058531 |
| *PDE6A* | -2.230710563 | 0.543930887 | -4.101091918 | 4.11E-05 | 0.000325998 |
| *GPC5* | -2.241515361 | 0.597289268 | -3.752813724 | 0.000174861 | 0.001119618 |
| *GABRD* | -2.2442623 | 0.470495589 | -4.769996476 | 1.84E-06 | 2.29E-05 |
| *TSLP* | -2.247601922 | 0.493556701 | -4.553887968 | 5.27E-06 | 5.65E-05 |
| *IL12B* | -2.262846769 | 0.72515024 | -3.120521297 | 0.001805312 | 0.007793396 |
| *GOLGA8M* | -2.273749444 | 0.396651927 | -5.732354469 | 9.90E-09 | 2.29E-07 |
| *SALL4* | -2.280542306 | 0.504355519 | -4.521695947 | 6.13E-06 | 6.46E-05 |
| *MTUS2* | -2.294951482 | 0.635303389 | -3.61237091 | 0.00030341 | 0.001779976 |
| *STAR* | -2.2967794 | 0.332028629 | -6.917413729 | 4.60E-12 | 2.30E-10 |
| *PCDHB12* | -2.305512027 | 0.664081745 | -3.471729264 | 0.000517118 | 0.0027821 |
| *VANGL2* | -2.310508621 | 0.411540265 | -5.614295415 | 1.97E-08 | 4.24E-07 |
| *ADAMTS14* | -2.319572011 | 0.423438546 | -5.477942512 | 4.30E-08 | 8.46E-07 |
| *SLC9C2* | -2.330371834 | 0.65740203 | -3.544819956 | 0.000392881 | 0.002214969 |
| *ANKRD18B* | -2.330483979 | 0.509781108 | -4.571538529 | 4.84E-06 | 5.26E-05 |
| *SLC7A4* | -2.331411271 | 0.654602509 | -3.561567884 | 0.000368647 | 0.002100698 |
| *LPAR4* | -2.341590259 | 0.377017057 | -6.210833735 | 5.27E-10 | 1.68E-08 |
| *ITGB6* | -2.342559762 | 0.411395229 | -5.694183099 | 1.24E-08 | 2.79E-07 |
| *NPTX2* | -2.344460734 | 0.68345462 | -3.430309293 | 0.000602894 | 0.003147296 |
| *KIRREL3* | -2.361271742 | 0.550307201 | -4.290824721 | 1.78E-05 | 0.000159952 |
| *CYP3A7* | -2.37781155 | 0.898206139 | -2.647289355 | 0.008113989 | 0.026803344 |
| *GSG1* | -2.379756506 | 0.529936235 | -4.490646888 | 7.10E-06 | 7.32E-05 |
| *SHANK1* | -2.383149172 | 0.394278407 | -6.044330936 | 1.50E-09 | 4.26E-08 |
| *PGBD5* | -2.383955404 | 0.598345791 | -3.984243625 | 6.77E-05 | 0.000501513 |
| *GABRR1* | -2.387080709 | 0.726411732 | -3.286126317 | 0.001015754 | 0.004825863 |
| *PCDHGB2* | -2.387264735 | 0.466969993 | -5.112244411 | 3.18E-07 | 4.94E-06 |
| *RXFP1* | -2.391151619 | 0.581530732 | -4.111823308 | 3.93E-05 | 0.000313641 |
| *ZPBP* | -2.394004862 | 0.618805459 | -3.86875201 | 0.000109394 | 0.000754131 |
| *NAP1L2* | -2.403127134 | 0.536740643 | -4.477259482 | 7.56E-06 | 7.72E-05 |
| *SHD* | -2.404723916 | 0.671559315 | -3.580806432 | 0.000342535 | 0.001972585 |
| *PXDN* | -2.405974643 | 0.733852223 | -3.278554685 | 0.001043401 | 0.004934344 |
| *GRID1* | -2.409121531 | 0.462599235 | -5.207794021 | 1.91E-07 | 3.17E-06 |
| *LAMC3* | -2.410285001 | 0.422000141 | -5.711573928 | 1.12E-08 | 2.55E-07 |
| *TUBB4A* | -2.41068724 | 0.471045604 | -5.117736411 | 3.09E-07 | 4.83E-06 |
| *UNC5C* | -2.438097434 | 0.732034763 | -3.330576029 | 0.000866665 | 0.004237088 |
| *ZNF556* | -2.43872408 | 0.927258951 | -2.63003563 | 0.008537592 | 0.027947666 |
| *TTC29* | -2.461653066 | 0.953629589 | -2.58135139 | 0.009841435 | 0.031468735 |
| *MYCN* | -2.465811591 | 0.455574257 | -5.412534958 | 6.21E-08 | 1.16E-06 |
| *UGT3A2* | -2.466566739 | 0.574829543 | -4.290953322 | 1.78E-05 | 0.000159905 |
| *BMP10* | -2.480009993 | 0.626388222 | -3.959221943 | 7.52E-05 | 0.000550116 |
| *ASPG* | -2.485207051 | 0.723425886 | -3.435330554 | 0.000591831 | 0.003098746 |
| *GDF11* | -2.491378774 | 0.291545922 | -8.545407735 | 1.28E-17 | 1.85E-15 |
| *ACP4* | -2.496123161 | 0.824022284 | -3.02919376 | 0.002452074 | 0.010036571 |
| *SHH* | -2.504936032 | 0.476499326 | -5.25695609 | 1.46E-07 | 2.50E-06 |
| *PCDH9* | -2.520264059 | 0.569452418 | -4.425767594 | 9.61E-06 | 9.48E-05 |
| *PRR15* | -2.528161551 | 0.575775501 | -4.390880727 | 1.13E-05 | 0.000108905 |
| *PURG* | -2.530495334 | 0.786857806 | -3.215949964 | 0.001300135 | 0.005938115 |
| *OR2G3* | -2.531874759 | 0.797763516 | -3.173715907 | 0.001505009 | 0.006704555 |
| *CERS1* | -2.535576969 | 0.522651509 | -4.851372143 | 1.23E-06 | 1.61E-05 |
| *TPSB2* | -2.543976586 | 0.490433719 | -5.187197553 | 2.13E-07 | 3.48E-06 |
| *PRSS3* | -2.559625654 | 0.763063438 | -3.354407416 | 0.000795352 | 0.003947578 |
| *SPATA18* | -2.570502241 | 0.527336283 | -4.874502899 | 1.09E-06 | 1.46E-05 |
| *LOXL4* | -2.577182773 | 0.57560479 | -4.47734768 | 7.56E-06 | 7.72E-05 |
| *USP41* | -2.579085236 | 0.387960232 | -6.647808262 | 2.97E-11 | 1.25E-09 |
| *SMIM2* | -2.582621601 | 0.863997838 | -2.989152849 | 0.002797521 | 0.011168917 |
| *GSG1L* | -2.587548223 | 0.65469491 | -3.952296229 | 7.74E-05 | 0.000563682 |
| *JAM2* | -2.597274568 | 0.445883682 | -5.825004759 | 5.71E-09 | 1.41E-07 |
| *DCHS2* | -2.612437071 | 0.944474566 | -2.766021623 | 0.005674477 | 0.019972343 |
| *POU4F1* | -2.620857622 | 0.534928884 | -4.899450565 | 9.61E-07 | 1.31E-05 |
| *RAET1G* | -2.620937456 | 0.774780567 | -3.382812591 | 0.000717476 | 0.003619034 |
| *THSD4* | -2.632188738 | 0.856275107 | -3.0739989 | 0.002112102 | 0.008874529 |
| *MRC2* | -2.636078853 | 0.386903659 | -6.813269385 | 9.54E-12 | 4.51E-10 |
| *MPPED2* | -2.643507904 | 0.773845488 | -3.416066832 | 0.000635327 | 0.003286258 |
| *PTPRG* | -2.650971681 | 0.663306409 | -3.996601942 | 6.43E-05 | 0.000479641 |
| *NLGN1* | -2.654485067 | 0.825194733 | -3.216798364 | 0.001296297 | 0.005922293 |
| *SLC7A2* | -2.662876863 | 0.660053834 | -4.034332845 | 5.48E-05 | 0.000418394 |
| *ATP12A* | -2.663550449 | 0.45699355 | -5.828420225 | 5.60E-09 | 1.38E-07 |
| *CERCAM* | -2.664805098 | 0.31380608 | -8.49188486 | 2.03E-17 | 2.74E-15 |
| *PIEZO2* | -2.698626856 | 0.621968373 | -4.338849002 | 1.43E-05 | 0.000133474 |
| *OR1J2* | -2.707750006 | 0.836052113 | -3.238733524 | 0.001200617 | 0.005545999 |
| *PCDHGC3* | -2.707969719 | 0.479296747 | -5.649881281 | 1.61E-08 | 3.50E-07 |
| *SLC26A9* | -2.708786648 | 0.737028589 | -3.675280295 | 0.000237588 | 0.001449431 |
| *CHRNB4* | -2.718943653 | 0.575530001 | -4.724243131 | 2.31E-06 | 2.79E-05 |
| *F10* | -2.742793111 | 0.859597184 | -3.19078885 | 0.001418849 | 0.006396339 |
| *CAMK2N2* | -2.744199076 | 0.582908661 | -4.707768577 | 2.50E-06 | 2.99E-05 |
| *FAT3* | -2.751133039 | 0.768849702 | -3.578245566 | 0.000345908 | 0.001990255 |
| *CCNA1* | -2.755419085 | 0.420069345 | -6.559438621 | 5.40E-11 | 2.15E-09 |
| *MMP16* | -2.782428578 | 0.803345159 | -3.463553054 | 0.000533091 | 0.002847712 |
| *CYP2A6* | -2.78605734 | 0.634043544 | -4.394110416 | 1.11E-05 | 0.000107512 |
| *ADAMTS18* | -2.817118474 | 0.864685985 | -3.257967082 | 0.001122134 | 0.005239274 |
| *TEX15* | -2.860847816 | 0.940426341 | -3.042075377 | 0.002349531 | 0.009681817 |
| *GFRA3* | -2.865480224 | 0.659745749 | -4.343309871 | 1.40E-05 | 0.000131139 |
| *KLRF2* | -2.867717301 | 1.015702345 | -2.82338356 | 0.004751968 | 0.017235141 |
| *OLIG2* | -2.867901747 | 0.709967688 | -4.03948207 | 5.36E-05 | 0.000410305 |
| *DLL3* | -2.870743339 | 0.562550038 | -5.103089763 | 3.34E-07 | 5.15E-06 |
| *CLSTN2* | -2.918377075 | 0.72549732 | -4.022588361 | 5.76E-05 | 0.000436975 |
| *CDH9* | -2.925372322 | 1.141040537 | -2.563775981 | 0.010354035 | 0.032730909 |
| *ERBB4* | -2.929972535 | 0.709404849 | -4.130183968 | 3.62E-05 | 0.000293239 |
| *HGF* | -2.942446214 | 0.440262667 | -6.683387976 | 2.33E-11 | 1.01E-09 |
| *LRRC18* | -2.950370839 | 1.025015876 | -2.878365992 | 0.003997411 | 0.014959456 |
| *ASS1* | -2.955375606 | 0.707224384 | -4.178837258 | 2.93E-05 | 0.000243766 |
| *GCGR* | -2.963305332 | 0.736563528 | -4.023149694 | 5.74E-05 | 0.000436143 |
| *CPA3* | -2.974866912 | 0.490592749 | -6.063821604 | 1.33E-09 | 3.82E-08 |
| *OGDHL* | -2.982541639 | 0.530892787 | -5.617973561 | 1.93E-08 | 4.16E-07 |
| *SLC6A13* | -2.991330198 | 0.52618704 | -5.684918041 | 1.31E-08 | 2.91E-07 |
| *SNCAIP* | -3.013204116 | 0.901255757 | -3.343339659 | 0.000827765 | 0.004075865 |
| *CCL1* | -3.020225061 | 0.786447987 | -3.840336691 | 0.000122866 | 0.000832677 |
| *PCDHB7* | -3.033598946 | 0.910340367 | -3.332378806 | 0.00086107 | 0.004217549 |
| *PCDHB11* | -3.043694395 | 0.76758651 | -3.965278642 | 7.33E-05 | 0.000538323 |
| *IL17RE* | -3.059308561 | 0.445669564 | -6.864522068 | 6.67E-12 | 3.26E-10 |
| *NTRK3* | -3.073079702 | 0.764154451 | -4.02154263 | 5.78E-05 | 0.000438604 |
| *ABCC8* | -3.086235764 | 1.074725167 | -2.871651151 | 0.004083335 | 0.015209088 |
| *CNTN1* | -3.12690434 | 0.922039487 | -3.391291136 | 0.000695642 | 0.00353244 |
| *RAET1E* | -3.128703986 | 0.599836844 | -5.215924995 | 1.83E-07 | 3.05E-06 |
| *OR10H1* | -3.141040238 | 0.886284493 | -3.544054153 | 0.000394024 | 0.002219832 |
| *ADAMTS1* | -3.154110592 | 0.548051191 | -5.755138648 | 8.66E-09 | 2.02E-07 |
| *ARPP21* | -3.187556463 | 0.820655128 | -3.884160782 | 0.000102684 | 0.000713832 |
| *TAFA5* | -3.20546877 | 0.748902011 | -4.280224547 | 1.87E-05 | 0.000166629 |
| *PTGDS* | -3.247461692 | 0.518259504 | -6.266091925 | 3.70E-10 | 1.24E-08 |
| *TMPRSS11F* | -3.347168943 | 1.008211341 | -3.319908044 | 0.000900471 | 0.004382735 |
| *ADAMTS15* | -3.435628899 | 0.673087596 | -5.104281997 | 3.32E-07 | 5.12E-06 |
| *TWIST1* | -3.486083235 | 0.595901429 | -5.850100478 | 4.91E-09 | 1.24E-07 |
| *LYPD6B* | -3.48928551 | 0.603612898 | -5.780667578 | 7.44E-09 | 1.77E-07 |
| *PCDHB10* | -3.493694951 | 0.698303097 | -5.003121086 | 5.64E-07 | 8.17E-06 |
| *CBLN2* | -3.496882673 | 0.9323598 | -3.75057212 | 0.000176432 | 0.001127623 |
| *HHIP* | -3.518415819 | 0.655048455 | -5.371229858 | 7.82E-08 | 1.43E-06 |
| *TBX18* | -3.5459224 | 0.932801978 | -3.801366725 | 0.0001439 | 0.000951003 |
| *PSKH2* | -3.546747205 | 0.853087522 | -4.157542003 | 3.22E-05 | 0.000263888 |
| *GSC* | -3.570101059 | 0.671274847 | -5.31838944 | 1.05E-07 | 1.86E-06 |
| *C2CD4B* | -3.597232394 | 0.942453164 | -3.816881867 | 0.000135149 | 0.000901448 |
| *PLAAT5* | -3.617019493 | 0.583569702 | -6.198093356 | 5.72E-10 | 1.80E-08 |
| *NRXN3* | -3.62207215 | 0.781302296 | -4.635942031 | 3.55E-06 | 4.05E-05 |
| *ADGRG6* | -3.632102003 | 0.706576603 | -5.140422127 | 2.74E-07 | 4.35E-06 |
| *PCDHB9* | -3.63474088 | 0.665598909 | -5.460857626 | 4.74E-08 | 9.19E-07 |
| *GPR32* | -3.642422661 | 0.91939408 | -3.961764319 | 7.44E-05 | 0.000545172 |
| *RBFOX3* | -3.67554298 | 0.763535422 | -4.813847365 | 1.48E-06 | 1.89E-05 |
| *NEK10* | -3.685553308 | 0.465098224 | -7.924247221 | 2.30E-15 | 2.15E-13 |
| *BHLHE23* | -3.775751544 | 0.659483532 | -5.725315885 | 1.03E-08 | 2.37E-07 |
| *HMX3* | -3.786576337 | 1.083903316 | -3.493463191 | 0.000476799 | 0.002605029 |
| *CACNA1C* | -3.818280806 | 0.450300365 | -8.479408644 | 2.26E-17 | 3.04E-15 |
| *ROBO2* | -3.836407063 | 1.035382004 | -3.705305915 | 0.000211136 | 0.001309248 |
| *CT45A5* | -3.879850601 | 1.396468897 | -2.778329406 | 0.00546392 | 0.019371244 |
| *BTC* | -3.90800379 | 0.822871087 | -4.749229681 | 2.04E-06 | 2.50E-05 |
| *COL2A1* | -3.96020878 | 0.518243075 | -7.641604825 | 2.15E-14 | 1.63E-12 |
| *NCR2* | -3.97571502 | 0.946927585 | -4.198541771 | 2.69E-05 | 0.000226352 |
| *SSTR2* | -4.141858789 | 0.724224311 | -5.71902755 | 1.07E-08 | 2.45E-07 |
| *KCNJ6* | -4.218343914 | 1.163554699 | -3.625393732 | 0.000288522 | 0.001705212 |
| *NETO1* | -4.265960835 | 0.706825704 | -6.03537875 | 1.59E-09 | 4.48E-08 |
| *FBLN1* | -4.272137349 | 0.724623044 | -5.895668627 | 3.73E-09 | 9.62E-08 |
| *LEP* | -4.322880912 | 1.29260956 | -3.344305229 | 0.000824889 | 0.004065498 |
| *POSTN* | -4.40101936 | 1.109943729 | -3.965083315 | 7.34E-05 | 0.000538389 |
| *EFNB3* | -4.697097337 | 0.685619787 | -6.850877741 | 7.34E-12 | 3.55E-10 |
| *CHRM5* | -4.873112688 | 0.681983717 | -7.145497124 | 8.97E-13 | 5.05E-11 |
| *KCNE5* | -4.904535622 | 0.618319739 | -7.932037928 | 2.16E-15 | 2.03E-13 |
| *UNCX* | -4.92594126 | 1.285827385 | -3.830950653 | 0.000127649 | 0.000859757 |
| *ASTN1* | -5.040869575 | 1.151647682 | -4.377093494 | 1.20E-05 | 0.000114748 |
| *EBF3* | -5.241253245 | 0.907510349 | -5.775419809 | 7.68E-09 | 1.81E-07 |
| *SIM1* | -5.367098042 | 1.456461025 | -3.68502689 | 0.000228678 | 0.00140416 |
| *MYT1L* | -5.507907139 | 1.269275284 | -4.339411008 | 1.43E-05 | 0.000133188 |
| *IRX1* | -5.791659885 | 0.8880827 | -6.521532154 | 6.96E-11 | 2.72E-09 |
| *AGR2* | -6.276036299 | 0.878433922 | -7.144574158 | 9.03E-13 | 5.08E-11 |
| *GABRE* | -6.410249348 | 0.81265326 | -7.888049758 | 3.07E-15 | 2.78E-13 |
| *MYO18B* | -6.791586499 | 0.766379692 | -8.861908227 | 7.87E-19 | 1.40E-16 |
| *NKX3-2* | -7.061533498 | 1.18388713 | -5.964701634 | 2.45E-09 | 6.67E-08 |
| *PDPN* | -7.477452041 | 1.562282894 | -4.786234345 | 1.70E-06 | 2.14E-05 |
| *RASL12* | -7.586638109 | 1.15355851 | -6.576725884 | 4.81E-11 | 1.95E-09 |
| *SIX3* | -8.959849913 | 0.970428362 | -9.232881336 | 2.63E-20 | 6.13E-18 |
| *BRINP2* | -9.017786332 | 1.14534293 | -7.873437814 | 3.45E-15 | 3.09E-13 |

**Supplementary Table S3. Univariate Cox analysis of 51 VRSGs in TCGA AML.**

| **Genes** | **Hazard Ratio (HR)** | **95% CI Down** | **95% CI UP** | **P value** |
| --- | --- | --- | --- | --- |
| *ITPK1* | 2.217 | 1.445 | 3.403 | **<0.001** |
| *G6PD* | 2.197 | 1.427 | 3.383 | **<0.001** |
| *GRK6* | 2.041 | 1.334 | 3.123 | **0.001** |
| *TNFSF15* | 2.003 | 1.299 | 3.088 | **0.002** |
| *SRC* | 1.927 | 1.251 | 2.968 | **0.003** |
| *PIK3R5* | 1.918 | 1.250 | 2.942 | **0.003** |
| *NINJ1* | 1.863 | 1.209 | 2.871 | **0.005** |
| *ABI3* | 1.795 | 1.169 | 2.755 | **0.007** |
| *MAP2K1* | 1.679 | 1.097 | 2.570 | **0.017** |
| *MOB3A* | 1.641 | 1.072 | 2.512 | **0.023** |
| *PRKCD* | 1.636 | 1.068 | 2.506 | **0.024** |
| *ARPC1B* | 1.618 | 1.059 | 2.474 | **0.026** |
| *BAG3* | 1.617 | 1.055 | 2.480 | **0.028** |
| *PIM1* | 1.609 | 1.053 | 2.457 | **0.028** |
| *HK3* | 1.514 | 0.992 | 2.309 | 0.055 |
| *CEBPB* | 1.501 | 0.982 | 2.297 | 0.061 |
| *VENTX* | 1.370 | 0.898 | 2.091 | 0.144 |
| *BCL6* | 1.334 | 0.874 | 2.037 | 0.182 |
| *NADK* | 1.325 | 0.869 | 2.021 | 0.192 |
| *LGALS3* | 1.314 | 0.859 | 2.009 | 0.208 |
| *CDKN2B* | 1.311 | 0.859 | 1.999 | 0.210 |
| *KCNJ12* | 1.273 | 0.832 | 1.946 | 0.266 |
| *MAP2K6* | 1.220 | 0.799 | 1.863 | 0.357 |
| *TP63* | 1.207 | 0.791 | 1.840 | 0.382 |
| *ZFP36* | 1.173 | 0.770 | 1.788 | 0.458 |
| *CXCL1* | 1.163 | 0.763 | 1.773 | 0.483 |
| *PDZD2* | 1.128 | 0.737 | 1.726 | 0.579 |
| *SFN* | 1.125 | 0.738 | 1.716 | 0.583 |
| *CDKN1A* | 1.116 | 0.732 | 1.699 | 0.611 |
| *MCL1* | 1.115 | 0.732 | 1.699 | 0.611 |
| *IL1A* | 1.114 | 0.730 | 1.699 | 0.618 |
| *DUSP16* | 1.095 | 0.718 | 1.670 | 0.673 |
| *CBX7* | 1.071 | 0.703 | 1.634 | 0.748 |
| *UBTD1* | 1.060 | 0.696 | 1.616 | 0.786 |
| *BLVRA* | 0.996 | 0.653 | 1.520 | 0.985 |
| *SGK1* | 0.937 | 0.616 | 1.428 | 0.763 |
| *TMSB4X* | 0.933 | 0.612 | 1.421 | 0.746 |
| *KL* | 0.852 | 0.558 | 1.300 | 0.457 |
| *HJURP* | 0.847 | 0.555 | 1.292 | 0.441 |
| *VEGFA* | 0.835 | 0.547 | 1.274 | 0.402 |
| *WT1* | 0.828 | 0.542 | 1.265 | 0.382 |
| *MAPK12* | 0.792 | 0.517 | 1.216 | 0.287 |
| *ERRFI1* | 0.783 | 0.513 | 1.196 | 0.258 |
| *SERPINE1* | 0.751 | 0.491 | 1.148 | 0.186 |
| *MAST1* | 0.748 | 0.490 | 1.141 | 0.178 |
| *MMP9* | 0.715 | 0.467 | 1.093 | 0.121 |
| *FOS* | 0.664 | 0.435 | 1.012 | 0.057 |
| *IGFBP3* | 0.623 | 0.407 | 0.953 | **0.029** |
| *AR* | 0.596 | 0.389 | 0.912 | **0.017** |
| *CDK6* | 0.570 | 0.368 | 0.882 | **0.012** |
| *ID4* | 0.537 | 0.350 | 0.823 | **0.004** |

**Supplementary Table S4. Monocytic differentiation gene set.**

| **Gene Effects** | **Genes** |
| --- | --- |
| Monocytic differentiation marker | *CTSC* |
| Monocytic differentiation marker | *SELL* |
| Monocytic differentiation marker | *CD68* |
| Monocytic differentiation marker | *CD163* |
| Monocytic differentiation marker | *CD9* |
| Monocytic differentiation marker | *CD36* |
| Monocytic differentiation marker | *LILRB1* |
| Monocytic differentiation marker | *ITGB1* |
| Monocytic differentiation marker | *PECAM1* |
| Monocytic differentiation marker | *LILRB2* |
| Monocytic differentiation marker | *ITGAX* |
| Monocytic differentiation marker | *CD93* |
| Monocytic differentiation marker | *CTSA* |
| Monocytic differentiation marker | *FCGR1A* |
| Monocytic differentiation marker | *CD4* |
| Monocytic differentiation marker | *CD14* |
| Monocytic differentiation marker | *LILRB3* |
| Monocytic differentiation marker | *CTSD* |
| Monocytic differentiation marker | *LYZ* |
| Monocytic differentiation marker | *ITGB2* |
| Monocytic differentiation marker | *ITGAM* |
| Monocytic differentiation marker | *CTSB* |
| Monocytic differentiation marker | *LILRB4* |

**Supplementary Table S5. SenMayo gene set.**

| **Genes (human)** | **Classification** | **State** |
| --- | --- | --- |
| *ACVR1B* | Transmembrane signal receptors | Transmembrane |
| *ANG* | Miscellaneous | Secreted |
| *ANGPT1* | Intercellular signal molecule | Secreted |
| *ANGPTL4* | Intercellular signal molecule | Secreted |
| *AREG* | Growth factor | Intracellular |
| *AXL* | Transmembrane signal receptors | Transmembrane |
| *BEX3* | Miscellaneous | Intracellular |
| *BMP2* | Growth factor | Secreted |
| *BMP6* | Growth factor | Secreted |
| *C3* | Protease inhibitors | Secreted |
| *CCL1* | Cytokine/Chemokine | Secreted |
| *CCL13* | Cytokine/Chemokine | Secreted |
| *CCL16* | Cytokine/Chemokine | Secreted |
| *CCL2* | Cytokine/Chemokine | Secreted |
| *CCL20* | Cytokine/Chemokine | Secreted |
| *CCL24* | Cytokine/Chemokine | Secreted |
| *CCL26* | Cytokine/Chemokine | Secreted |
| *CCL3* | Cytokine/Chemokine | Secreted |
| *CCL3L1* | Cytokine/Chemokine | Secreted |
| *CCL4* | Cytokine/Chemokine | Secreted |
| *CCL5* | Cytokine/Chemokine | Secreted |
| *CCL7* | Cytokine/Chemokine | Secreted |
| *CCL8* | Cytokine/Chemokine | Secreted |
| *CD55* | Miscellaneous | Secreted |
| *CD9* | Transmembrane signal receptors | Transmembrane |
| *CSF1* | Cytokine/Chemokine | Secreted |
| *CSF2* | Cytokine/Chemokine | Secreted |
| *CSF2RB* | Transmembrane signal receptors | Transmembrane |
| *CST4* | Protease inhibitors | Secreted |
| *CTNNB1* | Transcription factors and regulators | Transmembrane |
| *CTSB* | (Metallo-)proteases | Secreted |
| *CXCL1* | Cytokine/Chemokine | Secreted |
| *CXCL10* | Cytokine/Chemokine | Secreted |
| *CXCL12* | Cytokine/Chemokine | Secreted |
| *CXCL16* | Cytokine/Chemokine | Secreted |
| *CXCL2* | Cytokine/Chemokine | Secreted |
| *CXCL3* | Cytokine/Chemokine | Secreted |
| *CXCL8* | Cytokine/Chemokine | Secreted |
| *CXCR2* | Cytokine/Chemokine | Transmembrane |
| *DKK1* | Intercellular signal molecule | Secreted |
| *EDN1* | Intercellular signal molecule | Secreted |
| *EGF* | Transmembrane signal receptors | Transmembrane |
| *EGFR* | Transmembrane signal receptors | Transmembrane |
| *EREG* | Growth factor | Secreted |
| *ESM1* | Intercellular signal molecule | Secreted |
| *ETS2* | Transcription factors and regulators | Intracellular |
| *FAS* | Transmembrane signal receptors | Transmembrane |
| *FGF1* | Growth factor | Secreted |
| *FGF2* | Growth factor | Secreted |
| *FGF7* | Growth factor | Secreted |
| *GDF15* | Growth factor | Secreted |
| *GEM* | Miscellaneous | Intracellular |
| *GMFG* | Intercellular signal molecule | Intracellular |
| *HGF* | (Metallo-) proteases | Secreted |
| *HMGB1* | Transcription factors and regulators | Intracellular |
| *ICAM1* | Miscellaneous | Intracellular |
| *ICAM3* | Miscellaneous | Intracellular |
| *IGF1* | Growth factor | Secreted |
| *IGFBP1* | Protease inhibitors | Secreted |
| *IGFBP2* | Protease inhibitors | Secreted |
| *IGFBP3* | Protease inhibitors | Secreted |
| *IGFBP4* | Protease inhibitors | Secreted |
| *IGFBP5* | Protease inhibitors | Secreted |
| *IGFBP6* | Protease inhibitors | Secreted |
| *IGFBP7* | Miscellaneous | Secreted |
| *IL10* | Cytokine/Chemokine | Secreted |
| *IL13* | Cytokine/Chemokine | Secreted |
| *IL15* | Cytokine/Chemokine | Secreted |
| *IL18* | Cytokine/Chemokine | Secreted |
| *IL1A* | Cytokine/Chemokine | Secreted |
| *IL1B* | Cytokine/Chemokine | Secreted |
| *IL2* | Cytokine/Chemokine | Secreted |
| *IL32* | Cytokine/Chemokine | Secreted |
| *IL6* | Cytokine/Chemokine | Secreted |
| *IL6ST* | Transmembrane signal receptors | Transmembrane |
| *IL7* | Cytokine/Chemokine | Secreted |
| *INHA* | Growth factor | Secreted |
| *IQGAP2* | Miscellaneous | Intracellular |
| *ITGA2* | Transmembrane signal receptors | Transmembrane |
| *ITPKA* | Protein modifying enzymes | Intracellular |
| *JUN* | Transcription factors and regulators | Intracellular |
| *KITLG* | Growth factor | Intracellular |
| *LCP1* | Miscellaneous | Intracellular |
| *MIF* | Protein modifying enzymes | Secreted |
| *MMP1* | (Metallo-)proteases | Secreted |
| *MMP10* | (Metallo-)proteases | Secreted |
| *MMP12* | (Metallo-)proteases | Secreted |
| *MMP13* | (Metallo-)proteases | Secreted |
| *MMP14* | (Metallo-)proteases | Intracellular |
| *MMP2* | (Metallo-)proteases | Secreted |
| *MMP3* | (Metallo-)proteases | Secreted |
| *MMP9* | (Metallo-)proteases | Secreted |
| *NAP1L4* | Miscellaneous | Intracellular |
| *NRG1* | Growth factor | Secreted |
| *PAPPA* | (Metallo-)proteases | Secreted |
| *PECAM1* | Miscellaneous | Intracellular |
| *PGF* | Growth factor | Secreted |
| *PIGF* | Protein modifying enzymes | Transmembrane |
| *PLAT* | (Metallo-)proteases | Secreted |
| *PLAU* | (Metallo-)proteases | Secreted |
| *PLAUR* | Transmembrane signal receptors | Transmembrane |
| *PTBP1* | Miscellaneous | Intracellular |
| *PTGER2* | Transmembrane signal receptors | Transmembrane |
| *PTGES* | Protein modifying enzymes | Intracellular |
| *RPS6KA5* | Protein modifying enzymes | Intracellular |
| *SCAMP4* | Miscellaneous | Intracellular |
| *SELPLG* | Transmembrane signal receptors | Transmembrane |
| *SEMA3F* | Intercellular signal molecule | Secreted |
| *SERPINB4* | Protease inhibitors | Intracellular |
| *SERPINE1* | Protease inhibitors | Secreted |
| *SERPINE2* | Protease inhibitors | Secreted |
| *SPP1* | Cytokine/Chemokine | Secreted |
| *SPX* | Intercellular signal molecule | Secreted |
| *TIMP2* | Protease inhibitors | Secreted |
| *TNF* | Cytokine/Chemokine | Secreted |
| *TNFRSF10C* | Transmembrane signal receptors | Transmembrane |
| *TNFRSF11B* | Transmembrane signal receptors | Transmembrane |
| *TNFRSF1A* | Transmembrane signal receptors | Transmembrane |
| *TNFRSF1B* | Transmembrane signal receptors | Transmembrane |
| *TUBGCP2* | Miscellaneous | Intracellular |
| *VEGFA* | Growth factor | Secreted |
| *VEGFC* | Growth factor | Secreted |
| *VGF* | Intercellular signal molecule | Secreted |
| *WNT16* | Intercellular signal molecule | Secreted |
| *WNT2* | Intercellular signal molecule | Transmembrane |

**Supplementary Table S6. Primers for quantitative RT-PCR.**

| *GAPDH* human-F | GTCTCCTCTGACTTCAACAGCG |
| --- | --- |
| *GAPDH* human-R | ACCACCCTGTTGCTGTAGCCAA |
| G6PD human-F | CTGTTCCGTGAGGACCAGATCT |
| G6PD human-R | TGAAGGTGAGGATAACGCAGGC |
| BAG3 human-F | TGCCAGAAACCACTCAGCCAGA |
| BAG3 human-R | TGAGGATGAGCAGTCAGAGGCA |

**Supplementary Table S7. Validating the robustness of the 6-gene signature for OS in the combined dataset of Beat AML and GSE106291 by re-sampling analysis.**

| **Test** | **Univariate Cox analysis** | | **Kaplan-Meier analysis** | **AUC of ROC curve** | | |
| --- | --- | --- | --- | --- | --- | --- |
|  | **HR** | **Cox P** | **Log Rank P** | **1-Years** | **3-Years** | **5-Years** |
| 1 | 2.428449331 | 0.000510302 | 0.000810387 | 0.595 | 0.594 | 0.586 |
| 2 | 1.980881564 | 0.007254187 | 0.01377779 | 0.583 | 0.566 | 0.579 |
| 3 | 1.99297359 | 0.00625565 | 0.002288938 | 0.577 | 0.593 | 0.562 |
| 4 | 2.22156381 | 0.002190414 | 0.003259083 | 0.589 | 0.596 | 0.592 |
| 5 | 2.130477143 | 0.002571436 | 0.000686221 | 0.591 | 0.582 | 0.607 |
| 6 | 2.017207976 | 0.00699624 | 0.009892836 | 0.571 | 0.578 | 0.596 |
| 7 | 2.215187389 | 0.001194694 | 3.44E-05 | 0.595 | 0.597 | 0.577 |
| 8 | 2.234903506 | 0.001416923 | 0.001002983 | 0.593 | 0.608 | 0.613 |
| 9 | 1.938280993 | 0.009062201 | 0.00746163 | 0.591 | 0.589 | 0.589 |
| 10 | 2.195504664 | 0.002001351 | 0.000807519 | 0.594 | 0.6 | 0.593 |
| 11 | 2.99106304 | 1.96E-05 | 5.37E-05 | 0.625 | 0.621 | 0.615 |
| 12 | 2.189734031 | 0.002294857 | 0.001350631 | 0.6 | 0.609 | 0.552 |
| 13 | 2.286676051 | 0.001550786 | 0.000252044 | 0.601 | 0.596 | 0.627 |
| 14 | 1.714703271 | 0.02962767 | 0.003755333 | 0.579 | 0.582 | 0.568 |
| 15 | 1.956423158 | 0.008041993 | 0.000274068 | 0.569 | 0.596 | 0.591 |
| 16 | 2.15275247 | 0.001702024 | 0.000460061 | 0.596 | 0.582 | 0.587 |
| 17 | 1.786776522 | 0.021179623 | 0.012843934 | 0.554 | 0.585 | 0.548 |
| 18 | 2.146976033 | 0.002829463 | 0.000152286 | 0.599 | 0.597 | 0.597 |
| 19 | 2.577846539 | 0.000291535 | 7.37E-06 | 0.617 | 0.603 | 0.599 |
| 20 | 2.059631004 | 0.003456377 | 0.000264521 | 0.594 | 0.584 | 0.571 |
| 21 | 2.145608538 | 0.002380298 | 0.002862472 | 0.601 | 0.578 | 0.581 |
| 22 | 2.18371657 | 0.002285636 | 0.000173467 | 0.591 | 0.591 | 0.582 |
| 23 | 3.03457461 | 4.28E-05 | 3.33E-05 | 0.607 | 0.622 | 0.651 |
| 24 | 2.297794003 | 0.001160263 | 0.00062896 | 0.586 | 0.598 | 0.597 |
| 25 | 2.026661054 | 0.005908661 | 0.001086356 | 0.58 | 0.584 | 0.586 |
| 26 | 2.204029907 | 0.002224009 | 0.003405302 | 0.598 | 0.586 | 0.567 |
| 27 | 2.420649809 | 0.000488067 | 0.000265654 | 0.596 | 0.613 | 0.585 |
| 28 | 2.841620193 | 6.47E-05 | 0.000467289 | 0.596 | 0.609 | 0.606 |
| 29 | 2.022449312 | 0.005858867 | 0.000728994 | 0.595 | 0.59 | 0.568 |
| 30 | 2.522504639 | 0.000383537 | 0.000169059 | 0.589 | 0.596 | 0.584 |
| 31 | 1.879617347 | 0.010811165 | 0.0020309 | 0.588 | 0.57 | 0.592 |
| 32 | 2.273204811 | 0.002100766 | 0.002031058 | 0.591 | 0.595 | 0.589 |
| 33 | 2.526686435 | 0.000280181 | 1.92E-05 | 0.606 | 0.613 | 0.615 |
| 34 | 2.243517828 | 0.001652944 | 0.001628574 | 0.597 | 0.6 | 0.556 |
| 35 | 2.000537375 | 0.005436452 | 0.0007629 | 0.582 | 0.611 | 0.612 |
| 36 | 2.226972073 | 0.001456613 | 0.000242313 | 0.599 | 0.58 | 0.571 |
| 37 | 2.040780694 | 0.004710332 | 0.000673108 | 0.587 | 0.576 | 0.594 |
| 38 | 2.058953167 | 0.003707863 | 0.00477989 | 0.577 | 0.59 | 0.571 |
| 39 | 2.384000961 | 0.000575611 | 0.000404839 | 0.599 | 0.599 | 0.615 |
| 40 | 2.523136533 | 0.000448236 | 0.00019646 | 0.608 | 0.609 | 0.574 |
| 41 | 2.031383716 | 0.004846435 | 0.001673245 | 0.59 | 0.602 | 0.561 |
| 42 | 2.024237712 | 0.005450353 | 0.00438967 | 0.57 | 0.577 | 0.604 |
| 43 | 1.901927086 | 0.010626918 | 0.002772028 | 0.589 | 0.575 | 0.565 |
| 44 | 1.693499199 | 0.039157881 | 0.001493267 | 0.577 | 0.576 | 0.557 |
| 45 | 2.852548456 | 5.30E-05 | 2.31E-05 | 0.614 | 0.627 | 0.607 |
| 46 | 2.135063384 | 0.003660495 | 0.000754862 | 0.594 | 0.586 | 0.549 |
| 47 | 2.300076886 | 0.001671697 | 0.002357614 | 0.595 | 0.599 | 0.57 |
| 48 | 2.573369379 | 0.0001521 | 1.25E-05 | 0.61 | 0.611 | 0.616 |
| 49 | 2.400264337 | 0.000484658 | 0.000326329 | 0.611 | 0.599 | 0.578 |
| 50 | 2.238486276 | 0.001490126 | 0.001320203 | 0.59 | 0.587 | 0.553 |
| 51 | 2.278974944 | 0.001346452 | 0.000463282 | 0.589 | 0.594 | 0.561 |
| 52 | 2.092746673 | 0.002408797 | 5.71E-05 | 0.599 | 0.599 | 0.557 |
| 53 | 2.018729782 | 0.004972117 | 0.000834468 | 0.596 | 0.577 | 0.548 |
| 54 | 2.085435162 | 0.004780706 | 0.003719025 | 0.603 | 0.581 | 0.571 |
| 55 | 2.036044403 | 0.005157689 | 0.000227178 | 0.572 | 0.596 | 0.666 |
| 56 | 2.002933031 | 0.005739156 | 0.002322678 | 0.583 | 0.58 | 0.545 |
| 57 | 2.180056102 | 0.002660659 | 0.000429172 | 0.583 | 0.606 | 0.564 |
| 58 | 2.183707652 | 0.002717172 | 0.000675333 | 0.589 | 0.603 | 0.64 |
| 59 | 2.225576961 | 0.00141916 | 0.000267993 | 0.596 | 0.611 | 0.563 |
| 60 | 1.954819705 | 0.008084137 | 0.008207511 | 0.591 | 0.587 | 0.527 |
| 61 | 1.949230247 | 0.009137086 | 0.001270886 | 0.582 | 0.574 | 0.61 |
| 62 | 2.41112037 | 0.000650622 | 0.001024271 | 0.601 | 0.597 | 0.604 |
| 63 | 2.356637129 | 0.000540477 | 0.000384948 | 0.605 | 0.597 | 0.635 |
| 64 | 1.853915995 | 0.014112983 | 0.003357605 | 0.586 | 0.581 | 0.541 |
| 65 | 2.222641019 | 0.00124141 | 0.003969845 | 0.588 | 0.606 | 0.605 |
| 66 | 1.991205087 | 0.004589302 | 8.54E-05 | 0.593 | 0.602 | 0.573 |
| 67 | 2.274377941 | 0.000995954 | 0.000173625 | 0.596 | 0.617 | 0.621 |
| 68 | 1.810650926 | 0.020505839 | 0.004745815 | 0.588 | 0.567 | 0.527 |
| 69 | 1.765016642 | 0.02098469 | 0.002920362 | 0.584 | 0.561 | 0.576 |
| 70 | 2.77357647 | 6.63E-05 | 1.56E-05 | 0.606 | 0.602 | 0.631 |
| 71 | 2.165974974 | 0.002534898 | 0.000438375 | 0.597 | 0.589 | 0.542 |
| 72 | 2.167410716 | 0.002275835 | 0.000596851 | 0.584 | 0.586 | 0.634 |
| 73 | 2.145570223 | 0.002912076 | 0.002587035 | 0.58 | 0.591 | 0.566 |
| 74 | 1.875984987 | 0.011980815 | 0.003946741 | 0.576 | 0.572 | 0.568 |
| 75 | 1.723502218 | 0.040857154 | 0.006660178 | 0.578 | 0.569 | 0.614 |
| 76 | 2.853554217 | 5.00E-05 | 3.66E-05 | 0.615 | 0.603 | 0.6 |
| 77 | 2.565914303 | 0.000337772 | 0.000435976 | 0.607 | 0.604 | 0.552 |
| 78 | 1.724545473 | 0.033658766 | 0.002120656 | 0.569 | 0.577 | 0.576 |
| 79 | 1.91065082 | 0.013186775 | 0.001210069 | 0.585 | 0.576 | 0.531 |
| 80 | 2.1707803 | 0.001799242 | 0.000876245 | 0.601 | 0.597 | 0.558 |
| 81 | 2.484070641 | 0.000560708 | 0.001040207 | 0.58 | 0.581 | 0.614 |
| 82 | 1.84045217 | 0.018358773 | 0.007806552 | 0.578 | 0.577 | 0.559 |
| 83 | 2.533177945 | 0.000165478 | 3.24E-05 | 0.626 | 0.608 | 0.603 |
| 84 | 1.982351371 | 0.006648774 | 0.000307143 | 0.581 | 0.579 | 0.591 |
| 85 | 2.456407106 | 0.000509261 | 8.20E-05 | 0.619 | 0.612 | 0.602 |
| 86 | 1.778747721 | 0.021683528 | 0.005101792 | 0.57 | 0.579 | 0.598 |
| 87 | 1.910834236 | 0.010509023 | 0.004077361 | 0.568 | 0.594 | 0.587 |
| 88 | 2.335294753 | 0.000675109 | 0.000484726 | 0.594 | 0.59 | 0.619 |
| 89 | 2.069474725 | 0.004111856 | 0.00285072 | 0.589 | 0.594 | 0.591 |
| 90 | 1.915035186 | 0.01005059 | 0.005630369 | 0.577 | 0.589 | 0.601 |
| 91 | 2.208427407 | 0.001820404 | 0.001572557 | 0.604 | 0.582 | 0.593 |
| 92 | 2.378075235 | 0.000954156 | 1.79E-05 | 0.608 | 0.605 | 0.576 |
| 93 | 2.185661847 | 0.001760544 | 6.89E-05 | 0.611 | 0.59 | 0.59 |
| 94 | 2.035349707 | 0.004657196 | 0.005081111 | 0.576 | 0.588 | 0.55 |
| 95 | 1.726430506 | 0.032029216 | 0.009475589 | 0.575 | 0.58 | 0.55 |
| 96 | 2.504168721 | 0.000328877 | 0.000207168 | 0.612 | 0.601 | 0.634 |
| 97 | 2.184902814 | 0.001761474 | 0.000101705 | 0.585 | 0.597 | 0.618 |
| 98 | 2.181273605 | 0.002388479 | 0.000434252 | 0.586 | 0.585 | 0.57 |
| 99 | 2.35995125 | 0.001287565 | 0.001607864 | 0.589 | 0.579 | 0.589 |
| 100 | 2.41704554 | 0.000631389 | 8.70E-05 | 0.598 | 0.598 | 0.587 |
